# Protein mass spectrometry extends temporal blood meal detection over polymerase chain reaction in mouse-fed Chagas disease vectors

**DOI:** 10.1590/0074-02760180160

**Published:** 2018-08-27

**Authors:** Judith I Keller, Justin O Schmidt, Anna M Schmoker, Bryan A Ballif, Lori Stevens

**Affiliations:** 1 University of Vermont, Department of Biology, Burlington, VT, United States of America; 2 Southwestern Biological Institute, Tucson, AZ, United States of America

**Keywords:** LC-MS/MS, Chagas disease, blood meals, hemoglobin, albumin, SINE-PCR

## Abstract

**BACKGROUND:**

Chagas disease is highly prevalent in Latin America, and vector control is the most effective control strategy to date. We have previously shown that liquid chromatography tandem mass spectrometry (LC-MS/MS) is a valuable tool for identifying triatomine vector blood meals.

**OBJECTIVES:**

The purpose of this study was to determine blood meal detection ability as a function of method [polymerase chain reaction (PCR) vs. LC-MS/MS], time since feeding, and the effect of molting in mouse-fed triatomine insect vectors targeting hemoglobin and albumin proteins with LC-MS/MS and short interspersed nuclear elements (SINE)-based PCR.

**METHODS:**

We experimentally fed *Triatoma protracta* on mice and used LC-MS/MS to detect hemoglobin and albumin peptides over time post-feeding and post-molting (≤ 12 weeks). We compared LC-MS/MS results with those of a standard PCR method based on SINEs.

**FINDINGS:**

Hemoglobin-based LC-MS/MS detected blood meals most robustly at all time points post-feeding. Post-molting, no blood meals were detected with PCR, whereas LC-MS/MS detected mouse hemoglobin and albumin up to 12 weeks.

**MAIN CONCLUSIONS:**

In our study, the hemoglobin signature in the insect abdomen lasted longer than that of albumin and DNA. LC-MS/MS using hemoglobin shows promise for identifying triatomine blood meals over long temporal scales and even post-molting. Clarifying the frequency of blood-feeding on different hosts can foster our understanding of vector behavior and may help devise sounder disease-control strategies, including Ecohealth (community based ecosystem management) approaches.

Chagas disease is a major neglected tropical disease, with high endemic prevalence in Latin America where most transmission is by insect vectors in the subfamily Triatominae, also known as kissing bugs. Although autochthonous cases of Chagas disease in the United States are thought to be rare, Chagas incidence is likely, for a variety of reasons, to be underreported.^1^ ^-^ ^3^ Chagas disease is caused by the protozoan parasite *Trypanosoma cruzi*, withthe most common mode of transmission via a triatomine insect defecating while taking a blood meal or through the oral transmission route. The parasite is subsequently introduced into the new host’s blood stream through a break in the skin or mucous membrane.

Identifying the blood meal sources and feeding patterns of native insect vectors provides valuable data for understanding the ecology and behavior of the vector, presenting the need for research aimed at developing better blood meal identification methods. In addition, blood meal source prevalence can help elucidate local transmission cycles and can provide data for evidence-based vector control strategies.^4^ ^,^ ^5^ Insecticide spraying is often effective in the short term, but reinfestation of insect vectors and the financial burden of frequent large-scale spraying, as well as pyrethroid resistance make this not the most strategic option for battling Chagas disease.^6^ ^-^ ^8^ While massive campaigns of indoor residual insecticide spraying have effectively reduced introduced vectors in many regions, such as *Rhodnius prolixus* in Central America and *Triatoma infestans* in parts of South America,^7^ ^,^ ^8^ for native vectors such as *Triatoma dimidiata* in Central America, a recent study suggests a control strategy for Chagas disease includes a holistic Ecohealth approach that focuses on community participation, education, and vector control.^5^ ^,^ ^9^ ^-^ ^12^ As such, within the Ecohealth framework, vector control policies need to consider domestic vectors and perhaps underestimate sylvatic vector influence.^13^ To develop effective native vector control strategies, a knowledge of the vector ecology, including blood meal sources of vectors collected in various ecotopes, such as sylvatic, peridomestic, or domestic environments is important.^5^

However, blood meal source detection can be challenging. Triatomine insects are hematophagous and their digestive systems evolved to digest blood.^14^ Therefore, blood meal detection ability rapidly decays as the blood is digested. Few studies examined detection ability over time and have shown all detection methods work best with high quality material from recently-fed vectors.^15^ ^-^ ^19^ Often fieldwork conditions are not ideal for the storage and transportation of insect samples to maintain high quality of DNA or antigens commonly used for blood meal identification. Thus, time since feeding and storage of material could be two of the major reasons why in many studies upwards of 50% of samples do not have a blood meal detected.^19^ ^-^ ^23^

Blood meal sources have traditionally been detected by a number of methods including immunological approaches such as precipitin tests and enzyme-linked immunosorbent assay (ELISA)^24^ which are dependent on protein antibodies of blood meal sources present in an area, and DNA-based methods often focusing on polymerase chain reaction (PCR) amplification of mitochondrial or nuclear DNA. In addition, PCR amplification of a species-specific repetitive sequences of nuclear DNA, such as short interspersed nuclear elements (SINEs) using standard PCR,^17^ ^,^ ^18^ PCR amplification followed by sequencing of vertebrate mitochondrial DNA^25^ or ribosomal subunits such as 12 S,^26^ genomics,^27^ and other next-generation sequencing tools^28^ are emerging. Recently, we have shown the usefulness of a protein-based method based on liquid chromatography tandem mass spectrometry (LC-MS/MS) for blood meal source identification.^29^ A few studies have compared different methodologies directly. For example, Lucero et al. compared 12 S sequencing and qPCR,^30^ while Stevens et al. compared 12 S sequencing and cytochrome b.^25^ We compared LC-MS/MS with 12 S-based DNA sequencing and found LC-MS/MS identified blood meals from insects collected alive and dead; however, blood meals were not detected with DNA sequencing methods for three of the four samples.^29^ Blood meal detection ability can also vary depending on the species of host blood. A study examining the effect of both blood meal host species and time elapsed since a recent feeding, reported detection ability can drop off as early as 1-2 weeks for some species.^18^

Blood is a complex fluid with components that decay at different rates. Because iron stabilises molecules, blood proteins such as hemoglobin can be remarkably stable and have been detected in a 46-million-year-old fossilised mosquito.^16^ ^,^ ^31^ ^,^ ^32^ Peptides from hemoglobin, the most abundant protein in red blood cells, have been detected 309 days post-molt in ticks; and peptides from albumin, the most abundant blood serum protein, have been detected 85 days post-molt in ticks.^16^ Other components such as transferrin and immunoglobulins are known to degrade rapidly, while keratin, actin, histones, and tubulins were too conserved across the animal kingdom to be informative when evaluated for mass-spectrometry-based detection in ticks.^16^ Abundance, stability, and species-specific sequence variation make hemoglobin and albumin molecules great targets for LC-MS/MS-based techniques.^16^ ^,^ ^29^ ^,^ ^32^ LC-MS/MS has led to promising results when compared with DNA-based methods.^29^ A major benefit of using protein-based techniques is the quantity and quality of data gained from a single LC-MS/MS run.^16^

Information about blood meal perseverance in the insect gut is limited. Nevertheless, the longevity of blood meal detection ability in Chagas vectors is critical information to aid in the knowledge of the parasite transmission and overall feeding habits of the insect. For example, the average time for a blood meal digestion has been estimated to be approximately 14 days in adult female *T. infestans*,^33^ but different components of blood may vary and blood meals have been detected up to 10 weeks post-feeding in adult male and female *T. pallidipennis*, *T. barberi*, *T. dimidiata*, *T. phyllosoma*, and *T. longipennis.* ^15^ Molting behavior of insects can also affect blood meal detection. Kissing bugs are hemimetabolous, emerging wingless from an egg, and successively molt through five nymphal instars into winged adults.^34^ Experimentally evaluating blood meal detection post-molt would indicate whether or not we can detect feeding across molts and if the decrease in albumin and hemoglobin peptides over time can provide temporal information about the last blood meal.

In this study we determine how the ability to detect and identify a blood meal declines over time and is affected by molting. We assess Triatominae insect vector blood meals for the first time comparing two identification methods, protein LC-MS/MS and PCR of SINE-DNA in two experiments: (1) recently molted adult *Triatoma protracta* (Hemiptera: Reduviidae) fed once on mouse and assayed 0-4 weeks post-feeding; and (2) recently molted (fed approximately 1 week prior) *T. protracta* not fed after eclosion and assayed 0-12 weeks after molting. In addition to comparing LC-MS/MS with PCR, within LC-MS/MS we compare the ability of hemoglobin and albumin peptides to identify the source of a blood meal.

## MATERIALS AND METHODS

*Ethics* - White inbred ICR (CD-1) *Mus musculus* (house mouse) (Harlan Laboratories, Madison, WI) were used for feeding experiments. All procedures using mice were first approved by the Southwestern Biological Institute, Tucson, Arizona, USA Animal Care and Use Committee and follow international standards.^35^ Mice were immobilised in small mesh cages and placed in the enclosure containing the insect vectors. Insects were allowed to feed until satiated, or approximately for 30-60 minutes, at which point mice were removed.

*Experimental feeding and insect collection* - The parent colony of *T. protracta* vectors used in this study was established in 2009 from wild caught vectors collected in the Tucson, Arizona, USA basin with approximately one dozen individuals, and no additions have been made since then. As a result, we consider this population to be relatively inbred and individuals have been in colony for seven generations. The insects were housed in 30 x 25 x 28 cm polypropylene containers containing a paper lining at the base and egg cartons (composed of wood pulp fiber) for refugia. They were provided a continuous source of water, which consisted of 1.5 mL microtubes filled with distilled water and plugged with absorbent cotton. The typical temperature in the rearing facility ranged from approximately 10-20ºC in the winter and 20-32ºC in the summer, with a relative humidity of approximately 30% in the winter, and 10-40% in the summer. Spring and fall temperatures and humidity were intermediate between those of winter and summer. In March-June 2016, the lab-reared *T. protracta* were established into two groups representing the two experiments: (1) post-feeding or (2) post-molting. After the experimental mouse feeding, the two experimental groups were kept separately in equivalent aforementioned containers. No non-experimental insects were housed in the experimental groups.

The post-feeding (F) insects were collected after eclosing as adults and allowed a single blood meal on *M. musculus* within 19-37 days. Within an hour of feeding, the adult individuals (hereafter referred to as F0wk) were preserved in 95% ethanol and 5% glycerol and stored at 4ºC. Additional individuals were collected at each of the following time points: one week (F1wk), two weeks (F2wk), and four weeks (F4wk) post-feeding without access to an additional blood meal. Insects that died between sampling times were not analysed (see Supplementary data I, Table II for details of sample sizes and longevity). None of the insects in the F group survived past four weeks, except one specimen that was not analysed due to a mishap in the preparation. We analysed four insects from each time period, except F0wk where we analysed three.

Adult post-molting (M) insects were collected from the colony after feeding on *M. musculus* as 5th instar nymphs, and most molted to adults within one week of feeding. After molting, insects in this group were not fed, but were collected and preserved at the same time intervals (M0wk, etc) as the fed insects. These post-molt insects survived longer than the fed insects and sampling was extended to eight and 12 weeks (Table I). The longevity of post-molt bugs was a little longer, of the nine remaining at eight weeks, eight died before week 12 and we were able to analyse the one specimen alive at 12 weeks (Supplementary data I, Table II). Although most adult *Triatoma* species live much longer than 4-8 weeks, starvation has been shown to reduce adult longevity in *Rhodnius prolixus*,^36^ and the difference between the post-molt and post-fed specimen may be an artifact of small sample size. We sampled two insects from each time period, 0, 1, 2, 4, 8 and one surviving individual at 12 weeks. Samples were stored at 4ºC and within 1-4 weeks of collection, were shipped to the University of Vermont by priority mail in insulated containers, where they were stored at -20ºC until dissection in August 2016 and May 2017.

The 15 post-feeding and 11 post-molting *T. protracta* were evaluated using methods similar to Keller et al.^29^ Results were visualised with graphs made using JMP®, Version 13 (SAS Institute Inc., Cary, NC, 1989-2016). Below we highlight important aspects of the methods and indicate changes from our previous study.^29^

*Dissection of insect vectors* - For each insect, the abdomen was cut into left and right halves. Abdomen halves were randomly assigned to LC-MS/MS protein analysis or mouse-specific SINE-DNA PCR.

*Hemoglobin and albumin protein extraction, sodium dodecyl sulfate polyacrylamide gel electrophoresis (SDS-PAGE), and mass spectrometry* - We extracted protein from *T. protracta* insect abdomen halves as previously described^29^ except that 200 µL of denaturing sampling buffer was added per 0.1 g of insect tissue. For samples weighing 0.05 g and below, 100 µL of 95ºC denaturing sampling buffer was added. Denaturing SDS-PAGE using gel regions surrounding the molecular weight of hemoglobin (~16 kDa) and albumin (~65 kDa) were excised and prepared for mass spectrometry analysis as previously described.^29^ In brief, because LC-MS/MS works on peptides smaller than the hemoglobin and albumin proteins, following in-gel digestion with trypsin and peptide extraction, LC-MS/MS was performed using a linear ion trap-orbitrap (LTQ-Orbitrap; Thermo Electron, Waltham, Massachusetts, USA) where spectra all were collected in the orbitrap.^29^ ^,^ ^37^ Samples were subjected to 15 min of isocratic loading in 2.5% MeCN, 0.15% FA (Solvent A), and peptides were subsequently eluded with a 0-50% gradient of 99% MeCN, 0.15% FA (Solvent B) over 45 min (400 mL/min flow rate average across a flow splitter), followed by 10 min 100% Solvent B, and a 15 min equilibration with Solvent A.

LC-MS/MS does not directly sequence peptides, but rather infers amino acid sequences of short peptides based on theoretical peptide masses present in an underlying database, in our case, GenBank^38^ hemoglobin and albumin entries. We searched these mass spectra using the SEQUEST algorithm (Thermo Electron V26.12) against a custom forward and reverse concatenated database containing vertebrate hemoglobin sequences (20 January 2016, 17,000+ entries) extracted from GenBank as previously described^29^ and “serum albumin” (26 October 2016, 1600+ entries) in any curated field. Peptide identification and stringent filtering of peptides was as described in Keller et al.,^29^ where no reverse database matches resulted and false discovery of peptides was below 0.01%.

**Fig. 1:**
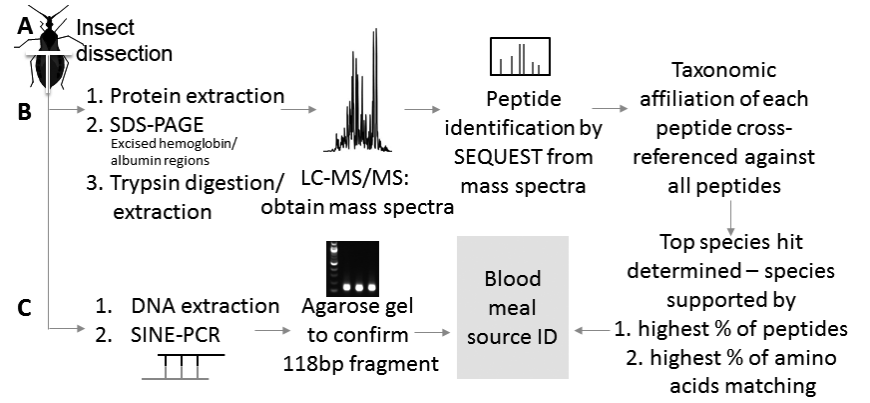
workflow describing liquid chromatography tandem mass spectrometry (LC-MS/MS) and DNA-based detection of blood meals. Insect abdomens were dissected into left and right halves (A) and subsequently were processed for blood meal species identification by protein (B) (see Keller et al.^29^ for additional details) and by DNA (C).

Blood meal sources were identified as previously described with a pipeline to infer the most likely blood source^29^ (Fig. 1). The pipeline considered the potential taxa represented by the peptide inferred from the mass spectra and cross-referenced the most likely blood meal source based on individual peptides in a sample. We subsequently quantified the identified protein coverage at the peptide and amino acid level. This provided the percentage support for a particular blood source (see Keller et al.^29^ for details).

*DNA extraction and SINE-based PCR* - DNA extraction used the DNeasy Blood and Tissue Kit (Qiagen, Valencia, CA) as previously described.^17^ ^,^ ^30^ Briefly, the manufacturer’s instructions for extracting tissue were followed using insect abdomens chopped finely with scissors. DNA was eluted with two separate sequential elutions of 100 µL each. DNA concentration was measured using a Nanodrop ND-1000 instrument (Thermo Scientific, Waltham, MA, USA), and the instrument was calibrated using the elution buffer (Qiagen, Valencia, CA) DNA was stored in.

DNA extracts were subjected to PCR amplification after optimising previously published methods for our equipment and reagents.^18^ ^,^ ^39^ ^,^ ^40^ The 12 µL PCR reaction contained 1 µL of DNA template, 0.2 µM each of forward (5’AGATGGCTCAGTGGGTAAAGG3’) and reverse (5’GTGGAGGTCAGAGGACAAACTT3’) primers, and 6 µL 1X EconoTaq PLUS GREEN (Lucigen, Middleton, WI, USA). PCR conditions were as follows: initial denaturisation for 5 min at 95ºC, 30 cycles of 95ºC for 30 s, annealing for 30 s at 55ºC, and extension for 30 s at 72ºC, followed by a final extension of 5 min at 72ºC. Nancy-520 stained 1.5 % agarose gels (Sigma-Aldrich, Milwaukee, WI, USA) were used to verify the 118 bp PCR fragments. Positive (DNA extracted from mouse tissue) and negative (PCR-grade water) controls were included in each set of PCR amplifications.

## RESULTS

**Fig. 2:**
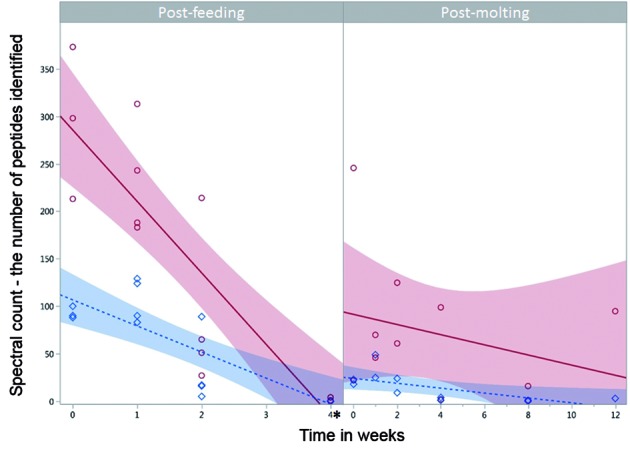
detection of hemoglobin and albumin peptides over time from a known *Mus musculus* blood meal source. The number of hemoglobin (red open circle/solid line) and albumin (blue diamond/dotted line) spectral counts decreased over time post-feeding (left) and post-molting (right). There were more peptides at 0 and 2 wk in the post-feeding specimens, but more at 4 wk for the post-molt. Linear regression lines were fit and 95% confidence intervals are shown (shading) with an alpha level of 0.05. Hemoglobin and albumin peptide abundance varied significantly between each time point post-feeding (Least Squares Regression, p < 0.001), and albumin was significantly lower in abundance than hemoglobin (Least Squares Regression, p < 0.001). Note the difference in time scales on the x-axis (*), none of the analysed post-feeding specimen lived longer than four weeks.

**Fig. 3:**
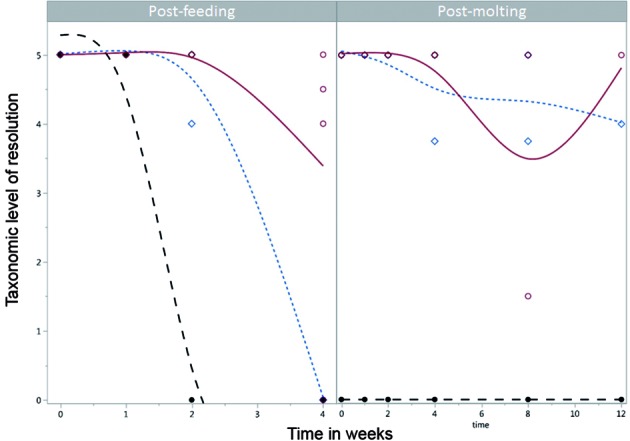
taxonomic level of resolution for *Mus musculus* blood meals over time. Taxonomic level of resolution by liquid chromatography tandem mass spectrometry (LC-MS/MS) varied between hemoglobin (red open circle/solid line) and albumin (blue diamond/dotted line) proteins and from short interspersed nuclear elements-based polymerase chain reaction (SINE-based PCR) (black closed circle/lined line) post-feeding (left). DNA, albumin, and hemoglobin provided species-specific blood meal identification up to one, two, and four weeks post-feeding, respectively. Post-molting (right), the taxonomic level of resolution for *M. musculus* blood meals by LC-MS/MS was stronger for hemoglobin and albumin than SINE DNA, which never detected a blood meal at any post-molting time point. A best-fit line (cubic smoothing spline, lambda of 0.0855, standardised X values) and 95% confidence intervals are shown.

The purpose of this study was to determine blood meal detection ability as a function of method (PCR vs. LC-MS/MS), time since feeding, and the effect of molting in Triatominae insect vectors targeting hemoglobin and albumin proteins with LC-MS/MS and SINE-based PCR. This is the first study to compare protein-based and DNA-based detection methods of experimentally-fed arthropod vectors. Briefly, hemoglobin-based LC-MS/MS yielded the most robust mouse blood meal detection and identification over time. In our experiments none of the post-feeding specimens lived longer than four weeks; however, we were able to identify the *M. musculus* blood meals based on hemoglobin and albumin peptides four and two weeks post-feeding, respectively (Figs 2-3, Supplementary data I, Fig. 2, Table III). With SINE-based PCR we were able to detect *M. musculus* blood meals up to one week post-feeding. The post-molting triatomines lived longer, and we were able to identify *M. musculus* hemoglobin and albumin peptides both up to 12 weeks post-molting, while SINE-based PCR detected no *M. musculus* blood meals at any time post-molting (Fig. 3, Supplementary data I, Fig. 2).

Post feeding

*LC-MS/MS - hemoglobin* - Using LC-MS/MS we were able to identify hemoglobin peptides throughout the entire sampling time of four weeks post-feeding, except for one F4wk sample; however the number of hemoglobin peptides decreased over time from an average of over 300 at F0wk to less than 10 at F4wk (Fig. 2, Table I, Supplementary data II, Table IV). For all four of the biological replicates at the 0, 1, and 2 wk time points and one of the F4wk samples the combination of peptides unambiguously identified the blood meal to the species level, *M. musculus*. With the other three F4wk samples, for two we were able to identify 2-6 species as the most probable blood source (which in both cases included the correct *M. musculus* blood meal - Sample ID 49 equally supported six species: *M. musculus*, *Otospermophilus beecheyi*, *Mus spretus*, *Mus minutoides*, *Jaculus jaculus*, *Callospermophilus lateralis*; sample ID 51 equally supported *M. musculus* and *M. spretus*), while as stated above, one replicate did not contain hemoglobin peptides (Table I).

*LC-MS/MS - albumin* - We were less successful detecting albumin peptides over time. We were only able to identify albumin peptides up to two weeks post-feeding and like hemoglobin, the number decreased over time from an average of over 100 at F0wk to 0 at F4wk (Fig. 2, Table II, Supplementary data III, Table V). For all the replicates at F0wk and F1wk, and three of the four replicates at F2wk, we identified the blood meal to the species level (Fig. 3). For the other F2wk sample, there was equally strong support for *Rattus norvegicus* and *M. musculus* as the blood meal source (Table II). Overall, hemoglobin and albumin peptide abundance varied significantly between each time point post-feeding (Least Squares Regression, p < 0.001), and albumin was significantly lower in abundance than hemoglobin (Least Squares Regression, p < 0.001) (Table III).

*SINE-DNA* - The *M. musculus*-specific-SINE based PCR was the least successful in detecting the *M. musculus* blood meal, we were only able to detect the blood meal in the F0wk and F1wk samples (Fig. 3).

Post molting

*LC-MS/MS - hemoglobin* - Using LC-MS/MS, we were able to identify hemoglobin peptides throughout the entire sampling time of 12 weeks post-molting, except for one M8wk samples. As expected, for the M0, 1, 2wk specimens, the number of hemoglobin peptides was lower than for the post-feeding experiment and generally decreased over time from an average of over 100 at M0wk to less than 10 at M8wk (Fig. 2, Table I). Spectral counts were more variable in post-molting individuals. For example, M8wk averaged around eight hemoglobin peptides while the single replicate M12wk contained 95 hemoglobin peptides. For all replicates except one M8wk sample we identified the blood meal to the species level. One M8wk sample contained a single hemoglobin peptide matching 261 species (Table I).

*LC-MS/MS - albumin* - As with the post-feeding specimens, we were less successful detecting albumin peptides post-molting. We were able to detect albumin peptides over all time points, but the number decreased from a range of 18-49 at the early times to 0-3 at the later (Fig. 2, Table II). For all the replicates at 0, 1, and 2 wk and one of the two M4wk, we identified the blood meal to the species level. For the other samples, we narrowed the likely blood meal sources to 2-7 species, again, all including known blood meal source *M. musculus* (sample ID 54 equally supported *M. musculus* and *R. norvegicus*; sample ID 23 and 30 equally supported *M. musculus*, *R. norvegicus*, *Sorex araneus*, *Octodon degus*, *Ochotona princeps*, *Dipodomys ordii*, and *Cricetulus griseus*). Albumin peptides appeared to decrease significantly after the 2 week timepoint (Table II). Overall, hemoglobin and albumin peptide abundance did not vary significantly post-molting (Least Squares Regression, p > 0.05) (Table III).

*SINE-DNA* - Our *M. musculus*-specific SINE-based PCR did not detect *M. musculus* blood meals at any time points post-molting (Fig. 3).

## DISCUSSION

Ideally, a combination of assays gives researchers a diverse toolbox for identification of blood meal sources. For blood-feeding arthropod disease vectors, including the triatomine vectors of Chagas disease, increasing our understanding of vector blood meal sources facilitates the design of evidence-based control strategies. Few papers have compared different blood meal detection techniques directly, e.g.,^30^ ^,^ ^31^ and this is the first paper to compare a LC-MS/MS protein-based approach to a well-established DNA-based approach. The assay based on protein identified the blood meal source for a longer time post-feeding as well as post-molting.

This study shows variation in detection of blood meals by LC-MS/MS and mouse-specific SINE-based PCR as a function of time since last feeding and time since molting. Overall, LC-MS/MS based on hemoglobin gave precise blood meal identification for the longest amount of time post-feeding and post-molting. Albumin peptides were also present for the majority of time points in both experiments, however, detection of albumin peptides dropped off sharply two weeks post-feeding. DNA-based detection using mouse-specific SINE-DNA was only successful in detecting fresh blood meals up to one week post-feeding and did not detect a blood meal at any time points for the post-molt experiment. To our knowledge, this study is the first to explore post-molt blood meal detection in triatomines, as only incidental findings have been reported previously.^19^

Using hemoglobin peptides opens the door to exploring blood sources of early stage nymphs that have molted into later stages and possibly adults. Many studies of blood meal sources, e.g.,^41^ focus on later stage triatomines, which are more likely to be encountered and are more mobile. Because we were able to detect peptides from a previous life stage up to 12 weeks post-molting with LC-MS/MS but not DNA, we suggest further studies that explore blood sources of early stage nymphs. Such future studies could include examining the nymphs themselves, or through experimental feeding to determine if blood sources of early stage nymphs could be detected in later nymphs or even adults. The SINE-based PCR did not detect any blood meals post-molt. Albumin did have a lingering signature post-molt, but recovered peptides were significantly lower in number. While protein analyses do not have the luxury of amplification technologies that exist for DNA, LC-MS/MS is becoming more sensitive. Because of the low fmol (femtomole = 10^-15^) amount of material needed for LC-MS/MS, future studies should examine the ability to detect blood proteins over the life cycle of the insect. The development and standardisation of methods for LC-MS/MS detection will increase our ability to detect blood sources in field-collected samples, and can boost the design of control strategies for the distinct transmission cycles maintained by different vector species in different localities.

An increasing number of studies are trying mass spectrometry-based techniques for blood meal identification in insect vectors.^16^ ^,^ ^42^ ^,^ ^43^ Our mass spectrometry-based technique differs from DNA sequencing in that the molecule is not sequenced per se, but rather highly accurate mass measurements are used to match observed mass spectra with theoretical ones based on protein sequences in GenBank.^29^ ^,^ ^32^ An alternative approach, matching spectral libraries using measurements made by Matrix Assisted Laser Desorption/Ionisation time of flight mass spectrometry (MALDI-TOF) has also been used^42^ ^,^ ^43^ but requires blood controls from animal species likely encountered in the field to make the matching spectral libraries. However, obtaining such material can be problematic and precludes identifying unanticipated taxa. LC-MS/MS matching theoretical to observed spectra provides a practical approach using sequence searching with data readily available in GenBank,^29^ which we only expect to grow in sequence information over time. In addition, as management decisions are often based at higher taxonomic levels (e.g. rodents in general, birds in general), identifying closely related species through conservation of hemoglobin sequences of related species allows for comprehensive vector management using LC-MS/MS data. In our study, the hemoglobin signature in the blood lasted longer than that of albumin, which has also been previously shown.^16^ We show that hemoglobin is detected in *T. protracta* even several weeks after the insect had fed, and was detected even longer, notably from fewer peptides, in insects that had molted but not subsequently fed (four and 12 weeks, respectively).

**TABLE I:** Summary statistics for hemoglobin peptides identified in adult *Triatoma protracta* abdomen halves in post-feeding and post-molting specimens

*Mus musculus* match									
	Sample ID		Unique peptides match/total unique^*a*^ (nº)	Peptides matching *M. musculus* (%)	Total AA identified based on no. of unique peptides^*b*^	AA mis- matches (nº)	Amino acid matching to *M. musculus* (%)	Spectral count^*c*^	Average spectral count
1. Post-feeding	F0wk	33	16/20	80.00	299	8	97.3	298	294.7
	F0wk	34	15/18	83.33	292	9	96.9	373	
	F0wk	35	18/24	75.00	399	16	96.0	213	
	F1wk	38	16/20	85.00	311	12	96.1	188	231.8
	F1wk	40	20/26	76.92	390	12	96.9	183	
	F1wk	41	15/19	78.95	300	8	97.3	243	
	F1wk	42	18/21	85.71	340	9	97.4	313	
	F2wk	43	14/15	93.33	213	1	99.5	65	89.3
	F2wk	44	13/14	92.86	200	1	99.5	51	
	F2wk	45	17/22	77.27	314	9	97.1	214	
	F2wk	46	4/4	100.00	56	0	100.0	27	
	F4wk	49	1/1^d^	100.00	13	0	100.0	1	2.3
	F4wk	50	4/4	100.00	56	0	100.0	4	
	F4wk	51	4/4^d^	100.00	53	0	100.0	4	
	F4wk	52	no peptides identified				0	
2. Post-molting	M0wk	15	23/28	82.14	422	15	96.4	246	134.0
	M0wk	4	10/12	83.33	186	6	96.8	22	
	M1wk	11	19/22	86.36	320	9	97.2	70	58.0
	M1wk	10	19/21	90.48	326	9	97.2	46	
	M2wk	17	20/22	90.91	293	6	98.0	126	93.5
	M2wk	19	10/12	83.33	197	5	97.5	61	
	M4wk	24	17/21	80.95	295	14	95.3	99	50.5
	M4wk	23	2/2	100.00	31	0	100.0	2	
	M8wk	27	1/1^*d*^	100.00	0	0	100.0	1	8.5
	M8wk	30	8/10	80.00	151	6	96.0	16	
	M12wk	54	18/21	85.71	317	9	97.2	95	95.0

AA: amino acid; *a*: the number of unique peptides identified in a sample that match the known blood meal source, *Mus musculus,* of the approximately 23 detectable peptides from trypsin digestion of 142 aa alpha and 147 aa beta hemoglobin (depending on the amino acid variation; based on GenBank entries NP_032244.2, BAG16710.1); *b*: number of amino acids of the unique peptides identified that match mouse; *c*: spectral count, or number of hemoglobin peptides identified in each liquid chromatography tandem mass spectrometry (LC-MS/MS) run; *d*: *M. musculus* was not uniquely identified as the most likely blood meal source. Sample ID 49 equally supported six species: *M. musculus*, *Otospermophilus beecheyi, Mus spretus, Mus minutoides, Jaculus jaculus, Callospermophilus lateralis*; Sample ID 51 equally supported *M. musculus* and *M. spretus*; The single peptide from sample ID 27 was a non-specific that had been reported in *M. musculus* and 260 other species.

**TABLE II:** Summary statistics for albumin peptides identified in adult *Triatoma protracta* abdomen halves in post-feeding and post-molting specimen

*Mus musculus* match									
	Sample ID		Unique peptides match/total unique^*a*^ (nº)	Peptides matching *M. musculus* (%)	Total AA identified based on no. of unique peptides^*b*^	AA mis- matches (nº)	Amino acid matching to *M. musculus* (%)	Spectral count^*c*^	Average spectral count
1. Post-feeding	F0wk	33	14/14	100.00	194	0	100.0	90	95
	F0wk	34	14/14	100.00	176	0	100.0	102	
	F0wk	35	14/14	100.00	166	0	100.0	93	
	F1wk	38	8/8	100.00	100	0	100.0	90	108.25
	F1wk	40	5/5	100.00	66	0	100.0	83	
	F1wk	41	15/15	100.00	182	0	100.0	129	
	F1wk	42	15/15	100.00	189	0	100.0	131	
	F2wk	43	9/9	100.00	116	0	100.0	17	30.25
	F2wk	44	9/9	100.00	116	0	100.0	17	
	F2wk	45	12/12	100.00	181	0	100.0	81	
	F2wk	46	3/3^*d*^	100.00	40	0	100.0	6	
	F4wk	49	no peptides identified					0
	F4wk	50	no peptides identified					
	F4wk	51	no peptides identified					
	F4wk	52	no peptides identified					
2. Post-molting	M0wk	15	14/14	100.00	172	0	100.0	23	20.5
	M0wk	4	11/11	100.00	138	0	100.0	18	
	M1wk	11	19/19	100.00	248	0	100.0	49	37
	M1wk	10	13/13	100.00	160	0	100.0	25	
	M2wk	17	10/10	100.00	134	0	100.0	25	17
	M2wk	19	7/7	100.00	83	0	100.0	9	
	M4wk	24	2/2	100.00	22	0	100.0	4	2.5
	M4wk	23	1/1^*d*^	100.00	13	0	100.0	1	
	M8wk	27	no peptides identified					0.5
	M8wk	30	1/1^*d*^	100.00	13	0	100.0	1	
	M12wk	54	3/3^*d*^	100.00	40	0	100.0	3	3

AA: amino acid; *a*: of the approximately 58 detectable peptides from trypsin digestion of the 609 AA albumin protein (depending on AA variation, based on GenBank sequence CAD29888.1), shown are the number of unique peptides identified in a sample that match the known blood meal source, *Mus musculus*; b: number of amino acids of the unique peptides identified that match mouse; *c*: spectral count, or number of albumin peptides identified in each liquid chromatography tandem mass spectrometry (LC-MS/MS) run; *d*: *M. musculus* was not uniquely identified as the most likely blood meal source. Sample ID 46 and 54 equally supported *M. musculus* and *Rattus norvegicus*; Sample ID 23 and 30 equally supported *M. musculus, R. norvegicus, Sorex araneus, Octodon degus, Ochotona princeps, Dipodomys ordii,* and *Cricetulus griseus*.

**TABLE III:** Least square regression of albumin and hemoglobin peptide abundance in post-feeding and post-molting experiment of *Triatoma protracta*

	Term	Estimate	t Ratio	Prob > |t|
1. Post-feeding	Time	-51.33298	-7.97	< 0.0001*
	Molecule (Albumin, Hemoglobin)	-44.86667	-4.79	< 0.0001*
	Time x Molecule interaction	23.879202	3.71	0.0010*
2. Post-molting	Time	-3.960947	-1.35	0.1938
	Molecule (Albumin, Hemoglobin)	-28.45455	-2.59	0.0183*
	Time x Molecule interaction	1.3869822	0.47	0.6421

Given hemoglobin is more abundant in blood and is highly stable, it is not necessarily surprising that it is detectable longer than albumin. However, this might not have been the case, particularly as albumin is also stable and as a larger molecule compared to hemoglobin (~608 vs. ~289 amino acids) offers more total tryptic peptides for identification by LC-MS/MS.^38^ Hemoglobin has a second advantage regarding peptide identification in that the size of the underlying database, in this case all hemoglobin entries currently in GenBank, is larger than that for albumin. Albumin has an order of magnitude fewer entries (> 17,000 for hemoglobin (20 January 2016) vs. < 1,700 for albumin (26 October 2016)), which could be a problem when examining samples from sylvatic vectors collected in regions with little molecular data on vertebrate biodiversity. Even if less useful for species identification, the presence or absence of albumin peptides could provide an estimate of the time window in which the insect vector fed, although this would require more controlled experiments to develop a range for time window estimates.

Blood meals have only occasionally been detected post-molting in triatomine insect vectors,^19^ and there is a gap in literature on this subject. Although we used positive controls (DNA extracted from mouse tissue) and checked for PCR inhibition with our samples (internal, same-tube controls), we did not detect a blood meal post-molt. Theoretically PCR can amplify from a single molecule, but it is likely that in the insect vector, DNA from mouse blood was not of sufficient quality or too low in abundance, and PCR requires an intact DNA strand for the sequence between the primers. With DNA we targeted a 118 bp fragment with PCR, however, this SINE transposable element has an estimated 2000 copies in the mouse genome and a detection limit of 0.01 ng 10^-5^ g using qPCR.^39^ In contrast, LC-MS/MS easily analyses small peptides and surveys all peptides extracted from the insect digestive system for a match to hemoglobin peptides reported in GenBank. Experimental feeding studies looking at blood meal detection at times post-feeding by Pinto et al.^18^ had similar results. They were able to detect a mouse blood meal in *T. infestans* 14, but not 21, days after feeding and reported a detection limit of 10 ng with ethidium bromide stained agarose gels, using the same mouse-specific PCR assay used in this study. Hemoglobin-based LC-MS/MS can potentially fill the gap in knowledge of an insect vector’s previous blood meal after the insect has molted or not fed for long periods of time. Triatomine nymphs need at least one blood meal to molt to the subsequent life stage and female vectors generally need a blood meal before egg laying, although autogeny has been recorded in some species of kissing bugs.^44^

Triatomine vector life spans vary, but have been recorded to last from several months to over a year^45^ ^,^ ^46^ and feeding patterns can change from various nymphal stages to adult stages, as well as over the life span of an adult.^47^ In addition, triatomine species can exhibit opportunistic feeding behaviors related to the relative abundance and proximity of animal blood sources.^48^ Therefore, detection of blood meals at various times post-feeding as well as post-molting, and elucidating blood meal sources from previous life stages, is an important aspect of making Ecohealth-based management decisions especially for native vectors.

In this study we show that LC-MS/MS allows correct identification of blood meal sources to the species level but the taxonomic level of resolution decreases with time and as a function of molting and the molecule examined. Although > 95% of amino acids identified were previously reported in GenBank in mouse, not all peptides identified matched the known blood source. Indeed, even with sequencing, a 100% match with DNA is also not always possible because of previously unidentified DNA polymorphisms. We chose to use mouse for our controlled blood meal as mice are easily available and have been used in previous feeding studies.^15^ ^,^ ^18^ However, they also come with challenges such as heterozygosity and various chromosome locations of hemoglobin genes.^49^

As we are critically examining the strengths and weaknesses of this LC-MS/MS technique, we spent considerable energy investigating the few instances when the peptide identified was not known to match to the known blood meal source. Two likely explanations for these mis-matches are: previously unknown polymorphisms, and, misidentification by the SEQUEST program. When our sample pool is corrected for likely cases of misidentification by SEQUEST, our lowest identify of 95.3% amino acids matching mouse increases to 99.7% (294/295 amino acids). If the same approach is applied to all samples, all blood source identification confidence based on amino acids increase to greater than 99.5%, and it may be a fair assumption that the 0.5% represent additional unknown polymorphisms. We detail in the supplementary material specific examples of misidentification by SEQUEST (See Supplementary data I, Table I, Fig. 1 ^54^). Furthermore, for a field study if several species match as the most likely blood meal source (e.g., Samples 26, 30, Table II), one could easily rule out those whose biogeography does not overlap with the species of Chagas vector examined, e.g., the Eurasian shrew, *Sorex araneus*. For others, e.g., American pika, *Ochotona princeps*, there is equal support from a single specimen for both pika and mouse. One would be able to comment on the likelihood of each based on other specimens examined in the same study. In addition, published host records, e.g.,^26^ would indicate if the host had been previously reported for Chagas vectors, although novel blood meal sources are regularly detected and need to be considered.

The use of a blood meal detection technique known to accurately detect blood meals across long temporal scales such as LC-MS/MS can lead to a better understanding of vector biology and for developing informed strategies for vector control. Chagas and other arthropod disease vectors have often not fed recently, but may contain remnants of a blood meal from some time ago. Indeed, in some studies field-collected triatomines were mostly found unfed (*Rhodnius prolixus*)^50^ and while 5th instar nymphs feed most frequently (*Meccus pallidipennis*),^51^ nymphs especially are capable of surviving long period of starvation.^50^ In addition to time since feeding, the quantity of a blood meal is likely to affect detection. Although in our experiment vectors were allowed to feed until satiated, this is not always the case in the wild.

In addition to enhancing our ability to detect blood meals for longer times after feeding, hemoglobin-based LC-MS/MS might also be able to detect multiple blood meals. Wild vectors often have multiple blood meal sources, a topic we have yet to address with our detection technique. As suggested previously^29^ further studies could examine the possibility of using synthetic peptides such as AQUA (*A*bsolute *QUA*ntification) peptides for quantification of a blood meal. Spiking a synthetic AQUA peptide into a blood meal sample could aid in quantification of a blood meal and allow more detailed detection of multiple blood meals. Our previous study showed the feasibility^29^ of this approach.

Freshly fed triatomine specimens are ideal for blood meal analysis. Storage conditions may affect detection ability and fieldwork conditions are not perfect for the storage and transportation of insect samples to maintain high quality of DNA or antigens commonly used for blood meal identification,^52^ however, we showed previously^29^ and in this study that LC-MS/MS seem less sensitive to the storage condition of the vectors. In addition, detection of non-recent blood meals is important to developing Ecohealth management decisions and developing vector control strategies. Therefore, using a technique such as hemoglobin-based LC-MS/MS has strong advantages in some situations for identifying blood meal sources, such as the ability of a single LC-MS/MS run to identify all blood meals at the same time and is also reasonably priced (see Keller et al.,^29^ Supplementary data I, Table III, and Önder et al.,^42^ for review on cost analysis) and proteomics resources are available in many areas with endemic triatomine populations.^53^ LC-MS/MS assays based on hemoglobin and potentially other proteins are a powerful tool for evaluating blood meals in Chagas disease vectors, and could be applied to other vector disease systems. The ability to detect blood proteins over long temporal scales and in molted individuals opens the door to using LC-MS/MS hemoglobin-sequence-based techniques in field-collected specimens and is a valuable part of the diverse toolbox for identification of blood meal sources.
