# Supplementary material for: Protein mass spectrometry extends temporal blood meal detection over polymerase chain reaction in mouse-fed Chagas disease vectors
Source: Mem Inst Oswaldo Cruz. 2018 Aug 27;113(10):e180160. doi: 10.1590/0074-02760180160 (PMC6167943; doi:10.1590/0074-02760180160)
Supplement: Supplementary file 1 [file 1678-8060-mioc-113-10-e180160-s1.pdf]

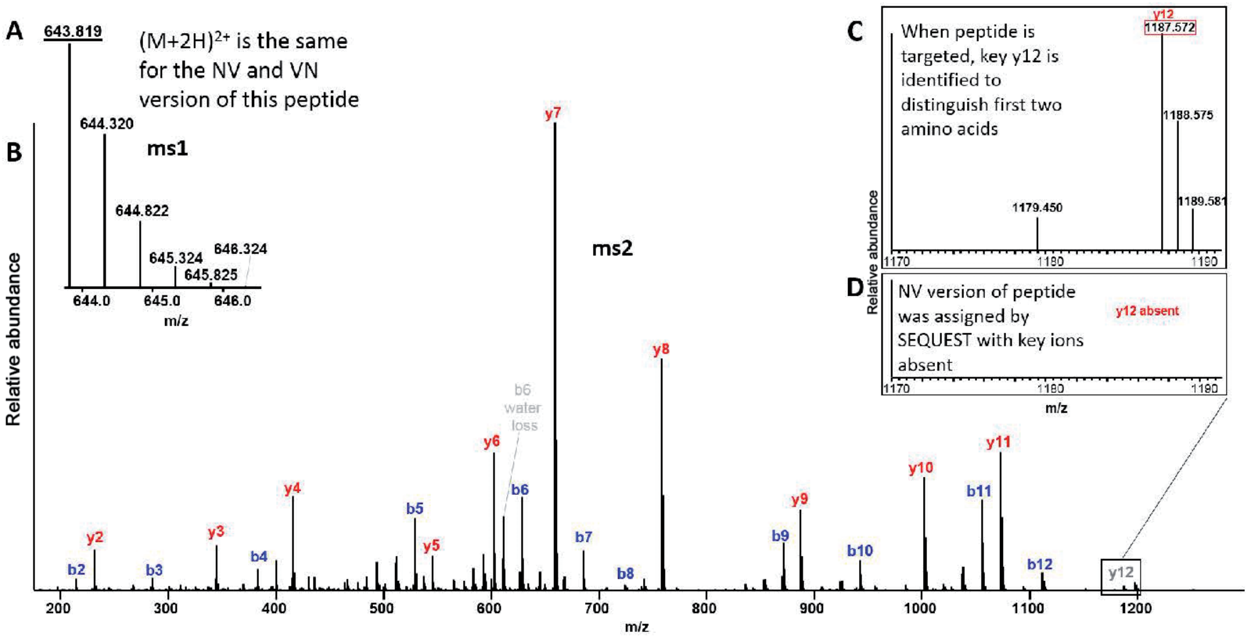

E NVADEVGGEALGR $(M+2H)^{2+}$ 643.820

Seq		b	y	
N	1	115.0503	1286.634	13
V	2	214.1187	~~1172.591~~	12
A	3	285.1558	1073.522	11

VNADEVGGEALGR $(M+2H)^{2+}$ 643.820

Seq		b	y	
V	1	100.0757	1286.634	13
N	2	214.1187	**1187.565**	12
A	3	285.1558	1073.522	11

Fig. 1: critical ions in the fragmentation spectra are needed to distinguish VNADEVGGEALGR from NVADEVGGEALGR. A peptide which shows frequent mismatching by SEQUEST in mouse blood is VNADEVGGEALGR, as it is often identified with the first two amino acids inverted to NVADEVGGEALGR.[29] We identify this peptide in our mouse-fed Triatominae and found that the critical b1, b2, and y12 ions that distinguish the first two amino acids are generally absent in the MS/MS spectra. When this is the case, the precursor peptide ions are often in low abundance.[54] However, when we perform targeted MS/MS analysis for the precursor mass of this peptide we regularly observe the y12 ion which allows one to appropriately determine the sequence as VNADEVGGEALGR. Indeed, we never observed fragment ions suggesting this peptide started with NV. This is strong evidence that VN and not NV is the proper solution for the N-terminal amino acids found in our samples. (A) The isotopic envelope for the precursor doubly-charged peptide with a theoretical monoisotopic mass of 643.820 m/z. The resulting MS/MS fragmentation spectrum (B) frequently includes some of the key diagnostic y12 fragment ion (C). When SEQUEST assigns the NV version of this peptide (D), key ions for determining the sequence of the first two amino acids is absent. The required y12 fragment ion of the NVADEVGGEALGR with a mass of 1172.591 is never identified (D-E), while the 1187.565 y12 fragment ion is identified when then peptide is targeted, allowing us to determine that VNADEVGGEALGR, which matches mouse, is indeed the correct sequence.

CC BY

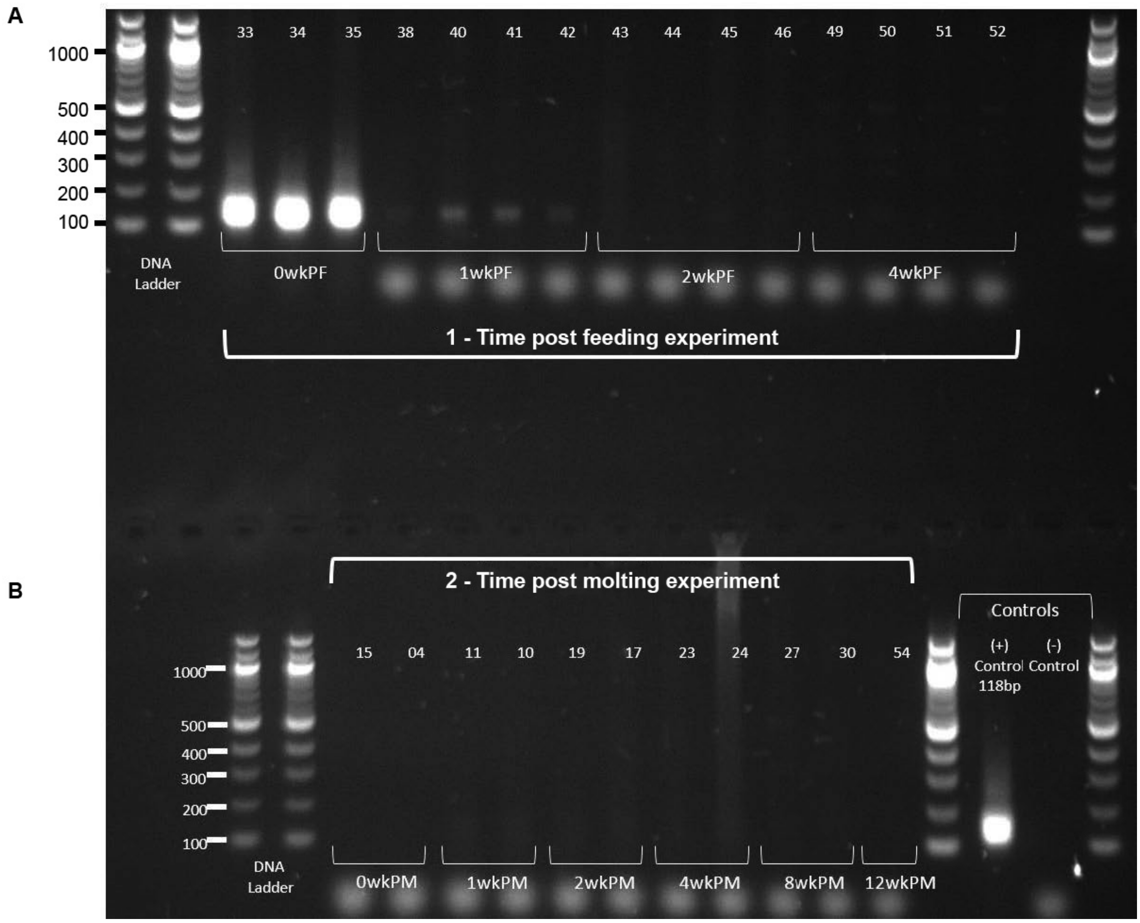

Fig. 2: agarose gel of short interspersed element-polymerase chain reaction (SINE-PCR) product from (A) time post-feeding experiment and (B) time post-molting experiment.

TABLE I

Instances when the peptide identified was not known to match to the known blood meal source. All spectra that did not match the known blood meal source, *Mus musculus*, were consistent with one of the two possible explanations: polymorphisms not previously reported for *M. musculus*, or peptides misidentified by SEQUEST. Manual examination of spectra was performed for these instances. For SEQUEST errors, the likely correct sequence is shown

Peptide	Molecule	Experiment	Samples	Type of mismatch	Correct sequence
IGGHAAEYGAEALER	HBA	PF	35,40,45	Previously identified polymorphism detected in mouse blood in Keller et al.[29]	-
VGSHAGEYGAEALER	HBA	PF<br>PM	38<br>04,10,11,15	Previously identified polymorphism detected in mouse blood in Keller et al.[29]	-
LLGDILIIVLAAHFSK	HBB	PM	24	Previously unidentified polymorphism	-
MFLSFPTTK	HBA	PM	30	Previously unidentified polymorphism	-
VADALATAADNLDDLPGALSALSDLHAHK	HBA	PF	38	Previously unidentified polymorphism	-
VADALATAAGHLDDLPGALSALSDLHAHK	HBA	PF<br>PM	33,34,35,38,40,41,42,43,44,45<br>04,10,11,15,24,54	Previously unidentified polymorphism	-
VADALTTAVAHLDDLPGALSALSDLHAYK	HBA	PM	30	Previously unidentified polymorphism	-
VNPDDVGGEALGR	HBB	PF	45	Previously unidentified polymorphism	-
VNVDDVGGEALGR	HBB	PF<br>PM	33,35,40,41,45<br>15,17	Previously unidentified polymorphism	-
VVAGVASALAHK	HBB	PF	40	Previously unidentified polymorphism	-
VVAGVASALAHKYH	HBB	PF	33	Previously unidentified polymorphism	-
YFDSFGDLSSACAIMGNAK	HBB	PM	19	Previously unidentified polymorphism	-
VAEALATAAGHLDDLPGALSALSDLHAHK	HBA	PF	41	SEQUEST error: Incorrect monoisotopic mass selection	VADALANAAGHLDDLPGALSALSDLHAHK
VGDALGNAVAHLDDLPGALSALSDLHAHK	HBA	PF<br>PM	34,35,38,40,42,45<br>11,15,17,19,54	SEQUEST error: incorrect assignment of peptide sequence, detecting a previously unidentified polymorphism	VADALANAAGHLDDLPAALSALSDLHAHK
VGDALGNAVAHLDDLPGALSALSDLHAHKLR	HBA	PF	35		VADALANAAGHLDDLPAALSALSDLHAHKLR
KVGDALGNAVAHLDDLPGALSALSDLHAHK	HBA	PF<br>PM	33,34,35,40,41,42<br>10,15,24,54		KVADALANAAGHLDDLPAALSALSDLHAHK
LLGNMIIIALSHHLGK	HBB	PM	24	SEQUEST error: incorrect assignment of peptide sequence	LLGNMIVIVLGHHLGK with Histidine oxidized
NVADEVGGEALGR	HBB	PF<br>PM	33,34,35,38,40,41,42,43,45,46,49,50,51<br>15,17,19,24,30	SEQUEST error: see Supplementary data I (Fig. 1)<br>Previously detected in mouse blood in Keller et al.[29]	VNADEVGGEALGR
LGEYGFQNALLVR	ALB	PF<br>PM	33,34,35,38,40,41,42,43,44,45<br>10,11,15,19,54	Isobaric nature of I and L<br>The mass spectrometer cannot distinguish isoleucine and leucine due to their identical m/z ratio. The mouse version of this peptide contains an I instead of an L at residue 10 and was corrected for.	LGEYGFQNAILVR

ALB: albumin; HBA: hemoglobin alpha; HBB: hemoglobin beta; PF: post-feed; PM: post-molt.

TABLE II

Lab-reared *Triatoma protracta* were established into two groups representing the two experiments: (1) post-feeding or (2) post-molting. The two experimental groups were kept separately in equivalent containers. No non-experimental insects were housed in the experimental groups. The number of specimens present in the container is recorded at each time point, along with the number of specimens that died, and the number used for analysis

	Time	No. of specimens	No. specimen(s) died	No. specimen(s) processed
1. Post-feeding	F0wk	35	0	3
	F1wk	32	0	4
	F2wk	28	0	4
	F4wk	24	0	4
	F8wk	20	19	0
2. Post-molting	M0wk	23	0	2
	M1wk	21	0	2
	M2wk	19	0	2
	M4wk	17	0	2
	M8wk	15	4	2
	M12wk	9	8	1

TABLE III

Distribution of detection of hemoglobin and albumin by liquid chromatography tandem mass spectrometry (LC-MS/MS) and DNA by short interspersed nuclear elements-polymerase chain reaction (SINE-PCR) in post-feeding and post-molting *Triatoma protracta*. X/X is the number of positive blood meal vectors/total number of vectors at each time point

		Time					
	Detection	0wk	1wk	2wk	4wk	8wk	12wk
1. Post-feeding	DNA	3/3	4/4	0/4	0/4		
	Albumin (total)	3/3	4/4	4/4	0/4		
	Albumin (to species level)	3/3	4/4	3/4	0/4		
	Hemoglobin (total)	3/3	4/4	4/4	3/4		
	Hemoglobin (to species level)	3/3	4/4	4/4	1/4		
2. Post-molting	DNA	0/2	0/2	0/2	0/2	0/2	0/1
	Albumin (total)	2/2	2/2	2/2	2/2	1/2	1/1
	Albumin (to species level)	2/2	2/2	2/2	1/2	0/2	0/1
	Hemoglobin (total)	2/2	2/2	2/2	2/2	2/2	1/1
	Hemoglobin (to species level)	2/2	2/2	2/2	2/2	1/2	1/1
