# Supplementary material for: Protein mass spectrometry extends temporal blood meal detection over polymerase chain reaction in mouse-fed Chagas disease vectors
Source: Mem Inst Oswaldo Cruz. 2018 Aug 27;113(10):e180160. doi: 10.1590/0074-02760180160 (PMC6167943; doi:10.1590/0074-02760180160)
Supplement: Supplementary file 2 [file 1678-8060-mioc-113-10-e180160-s2.pdf]

TABLE IV
SEQUEST identification of peptides.
Here we show the hemoglobin peptides identified in both experiments, post-feeding and post-molting

Scan	z	Sample ID	Band	PPM	XCorr	Delta correlation	Unique delta correlation	Reference	Redundancy		Peptides	
2149	2	33	B	3.4	3.2	0.92	0.92	gi|554577472|ref|XP_005880462.1|	74	K	AAVSGLWGK	V
3630	2	33	B	3.74	2.55	0.981	0.981	gi|554577472|ref|XP_005880462.1|	74	K	AAVSGLWGK	V
3632	2	33	B	4.66	2.84	0.954	0.954	gi|554577472|ref|XP_005880462.1|	74	K	AAVSGLWGK	V
3754	2	33	B	3.17	2.79	0.951	0.951	gi|554577472|ref|XP_005880462.1|	74	K	AAVSGLWGK	V
3762	2	33	B	3.49	2.75	0.953	0.953	gi|554577472|ref|XP_005880462.1|	74	K	AAVSGLWGK	V
3887	2	33	B	4.31	2.87	0.945	0.945	gi|554577472|ref|XP_005880462.1|	74	K	AAVSGLWGK	V
3889	2	33	B	3.86	2.73	0.981	0.981	gi|554577472|ref|XP_005880462.1|	74	K	AAVSGLWGK	V
4015	2	33	B	2.48	2.89	0.97	0.97	gi|554577472|ref|XP_005880462.1|	74	K	AAVSGLWGK	V
4024	2	33	B	0.24	2.67	0.943	0.943	gi|554577472|ref|XP_005880462.1|	74	K	AAVSGLWGK	V
4133	2	33	B	0.17	2.71	0.984	0.984	gi|554577472|ref|XP_005880462.1|	74	K	AAVSGLWGK	V
4259	2	33	B	2.49	2.84	0.963	0.963	gi|554577472|ref|XP_005880462.1|	74	K	AAVSGLWGK	V
4375	2	33	B	0.07	2.75	0.892	0.892	gi|554577472|ref|XP_005880462.1|	74	K	AAVSGLWGK	V
4385	2	33	B	0.09	2.53	0.977	0.977	gi|554577472|ref|XP_005880462.1|	74	K	AAVSGLWGK	V
4491	2	33	B	1.27	2.66	0.979	0.979	gi|554577472|ref|XP_005880462.1|	74	K	AAVSGLWGK	V
4498	2	33	B	1.33	2.6	0.984	0.984	gi|554577472|ref|XP_005880462.1|	74	K	AAVSGLWGK	V
2220	2	33	A	2.38	3.16	0.922	0.922	gi|554577472|ref|XP_005880462.1|	74	K	AAVSGLWGK	V
2226	2	33	A	2.38	3.11	0.918	0.918	gi|554577472|ref|XP_005880462.1|	74	K	AAVSGLWGK	V
4069	2	33	A	3.91	2.8	0.98	0.98	gi|554577472|ref|XP_005880462.1|	74	K	AAVSGLWGK	V
4193	2	33	A	-0.2	2.54	0.984	0.984	gi|554577472|ref|XP_005880462.1|	74	K	AAVSGLWGK	V
4311	2	33	A	0.19	2.52	0.983	0.983	gi|554577472|ref|XP_005880462.1|	74	K	AAVSGLWGK	V
4319	2	33	A	0.18	2.51	0.989	0.989	gi|554577472|ref|XP_005880462.1|	74	K	AAVSGLWGK	V
4433	2	33	A	1.63	2.7	0.984	0.984	gi|554577472|ref|XP_005880462.1|	74	K	AAVSGLWGK	V
4435	2	33	A	2.48	2.77	0.986	0.986	gi|554577472|ref|XP_005880462.1|	74	K	AAVSGLWGK	V
4589	2	33	A	0.26	2.7	0.92	0.92	gi|554577472|ref|XP_005880462.1|	74	K	AAVSGLWGK	V
2173	2	33	B	2.46	3.94	0.955	0.955	gi|532054982|ref|XP_005370208.1|	94	K	DFTPAAQAAFQK	V
2175	2	33	B	2.67	3.61	0.952	0.952	gi|532054982|ref|XP_005370208.1|	94	K	DFTPAAQAAFQK	V
2295	2	33	B	2.7	3.52	0.994	0.994	gi|532054982|ref|XP_005370208.1|	94	K	DFTPAAQAAFQK	V
2304	2	33	B	2.74	2.6	0.987	0.987	gi|532054982|ref|XP_005370208.1|	94	K	DFTPAAQAAFQK	V
3777	2	33	B	2.93	2.59	0.99	0.99	gi|532054982|ref|XP_005370208.1|	94	K	DFTPAAQAAFQK	V
4144	2	33	B	-0.2	2.66	0.952	0.952	gi|532054982|ref|XP_005370208.1|	94	K	DFTPAAQAAFQK	V
4443	2	33	B	0.27	2.67	0.99	0.99	gi|532054982|ref|XP_005370208.1|	94	K	DFTPAAQAAFQK	V
4453	2	33	B	0.25	2.77	0.984	0.984	gi|532054982|ref|XP_005370208.1|	94	K	DFTPAAQAAFQK	V
2263	2	33	A	3.33	2.95	0.959	0.959	gi|532054982|ref|XP_005370208.1|	94	K	DFTPAAQAAFQK	V

Scan	z	Sample ID	Band	PPM	XCorr	Delta correlation	Unique delta correlation	Reference	Redundancy		Peptides	
2388	2	33	A	3.34	2.55	0.971	0.971	gi|532054982|ref|XP_005370208.1|	94	K	DFTPAAQAAFQK	V
4640	2	33	A	-0.8	2.7	0.979	0.979	gi|532054982|ref|XP_005370208.1|	94	K	DFTPAAQAAFQK	V
2375	2	33	B	3.38	3.19	0	0.311	gi|27574244|pdb|1O1N|A	0	K	FLASVSTVLTSK	Y
2383	2	33	B	3.41	2.8	0	0.287	gi|27574244|pdb|1O1N|A	0	K	FLASVSTVLTSK	Y
2756	2	33	B	3.3	2.6	0	0.298	gi|27574244|pdb|1O1N|A	0	K	FLASVSTVLTSK	Y
2881	2	33	B	3.83	3.11	0	0.29	gi|27574244|pdb|1O1N|A	0	K	FLASVSTVLTSK	Y
2886	2	33	B	3.81	3.22	0	0.302	gi|27574244|pdb|1O1N|A	0	K	FLASVSTVLTSK	Y
3016	2	33	B	4.92	2.56	0	0.342	gi|27574244|pdb|1O1N|A	0	K	FLASVSTVLTSK	Y
3566	2	33	B	3.26	2.84	0	0.364	gi|27574244|pdb|1O1N|A	0	K	FLASVSTVLTSK	Y
3569	2	33	B	3.34	2.9	0	0.375	gi|27574244|pdb|1O1N|A	0	K	FLASVSTVLTSK	Y
3693	2	33	B	3.74	2.77	0	0.285	gi|27574244|pdb|1O1N|A	0	K	FLASVSTVLTSK	Y
3695	2	33	B	3.7	2.98	0	0.347	gi|27574244|pdb|1O1N|A	0	K	FLASVSTVLTSK	Y
3814	2	33	B	4.12	2.86	0	0.368	gi|27574244|pdb|1O1N|A	0	K	FLASVSTVLTSK	Y
3816	2	33	B	4.25	3.06	0	0.33	gi|27574244|pdb|1O1N|A	0	K	FLASVSTVLTSK	Y
3942	2	33	B	3.36	2.8	0	0.364	gi|27574244|pdb|1O1N|A	0	K	FLASVSTVLTSK	Y
3944	2	33	B	3.53	2.75	0	0.374	gi|27574244|pdb|1O1N|A	0	K	FLASVSTVLTSK	Y
4064	2	33	B	-0.2	2.69	0	0.383	gi|27574244|pdb|1O1N|A	0	K	FLASVSTVLTSK	Y
4072	2	33	B	-0.2	2.98	0	0.335	gi|27574244|pdb|1O1N|A	0	K	FLASVSTVLTSK	Y
4181	2	33	B	0.14	2.7	0	0.376	gi|27574244|pdb|1O1N|A	0	K	FLASVSTVLTSK	Y
4193	2	33	B	0.15	2.93	0	0.356	gi|27574244|pdb|1O1N|A	0	K	FLASVSTVLTSK	Y
4304	2	33	B	4.02	2.84	0	0.361	gi|27574244|pdb|1O1N|A	0	K	FLASVSTVLTSK	Y
4308	2	33	B	0.38	2.99	0	0.343	gi|27574244|pdb|1O1N|A	0	K	FLASVSTVLTSK	Y
4417	2	33	B	0.12	3.18	0	0.346	gi|27574244|pdb|1O1N|A	0	K	FLASVSTVLTSK	Y
4421	2	33	B	0.19	3.28	0	0.323	gi|27574244|pdb|1O1N|A	0	K	FLASVSTVLTSK	Y
2457	2	33	A	3.07	3.3	0	0.304	gi|27574244|pdb|1O1N|A	0	K	FLASVSTVLTSK	Y
2466	2	33	A	3.08	3.18	0	0.291	gi|27574244|pdb|1O1N|A	0	K	FLASVSTVLTSK	Y
4132	2	33	A	3.19	2.9	0	0.378	gi|27574244|pdb|1O1N|A	0	K	FLASVSTVLTSK	Y
4248	2	33	A	-0.1	2.72	0	0.351	gi|27574244|pdb|1O1N|A	0	K	FLASVSTVLTSK	Y
4500	2	33	A	0.2	3.04	0	0.376	gi|27574244|pdb|1O1N|A	0	K	FLASVSTVLTSK	Y
4511	2	33	A	0.23	2.99	0	0.344	gi|27574244|pdb|1O1N|A	0	K	FLASVSTVLTSK	Y
2151	2	33	B	4.53	3.54	0.114	0.114	gi|534467043|gb|AGU26656.1|	99	K	GTFASLSELHC#DK	L
2181	2	33	B	2.85	4.07	0.105	0.105	gi|344254270|gb|EGW10374.1|	99	K	IGGHGAEYGAEALER	M
2183	2	33	B	2.65	4.08	0.102	0.102	gi|344254270|gb|EGW10374.1|	99	K	IGGHGAEYGAEALER	M
2300	2	33	B	4.35	4.51	0.101	0.101	gi|344254270|gb|EGW10374.1|	99	K	IGGHGAEYGAEALER	M
2316	2	33	B	4.33	4.15	0.103	0.103	gi|344254270|gb|EGW10374.1|	99	K	IGGHGAEYGAEALER	M
4034	2	33	B	-0.2	3.97	0.103	0.103	gi|344254270|gb|EGW10374.1|	99	K	IGGHGAEYGAEALER	M
2270	2	33	A	3.47	3.6	0.102	0.102	gi|344254270|gb|EGW10374.1|	99	K	IGGHGAEYGAEALER	M
4168	2	33	A	2.09	3.46	0.102	0.102	gi|344254270|gb|EGW10374.1|	99	K	IGGHGAEYGAEALER	M

Scan	z	Sample ID	Band	PPM	XCorr	Delta correlation	Unique delta correlation	Reference	Redundancy		Peptides	
4316	2	33	A	0.2	2.74	0.101	0.101	gi|344254270|gb|EGW10374.1|	99	K	IGGHGAEYGAEALER	M
4184	3	33	B	0.16	3.48	0.523	0.523	gi|122513|sp|P02088.2|HBB1_MOUSE	65	K	KVITAFNDGLNHLDSLK	G
4310	3	33	B	0.27	3.14	0.514	0.514	gi|122513|sp|P02088.2|HBB1_MOUSE	65	K	KVITAFNDGLNHLDSLK	G
4322	3	33	B	0.17	3.08	0.494	0.494	gi|122513|sp|P02088.2|HBB1_MOUSE	65	K	KVITAFNDGLNHLDSLK	G
4430	3	33	B	0.13	3.82	0.562	0.562	gi|122513|sp|P02088.2|HBB1_MOUSE	65	K	KVITAFNDGLNHLDSLK	G
2351	2	33	A	3.27	3.57	0.742	0.742	gi|122513|sp|P02088.2|HBB1_MOUSE	65	K	KVITAFNDGLNHLDSLK	G
2540	2	33	B	4.73	3.7	0.226	0.226	gi|122513|sp|P02088.2|HBB1_MOUSE	88	R	LLGNMIVIVLGHHLGK	D
2547	2	33	B	4.68	3.32	0.19	0.19	gi|122513|sp|P02088.2|HBB1_MOUSE	88	R	LLGNMIVIVLGHHLGK	D
4035	2	33	B	-0.4	3.16	0.229	0.229	gi|122513|sp|P02088.2|HBB1_MOUSE	88	R	LLGNMIVIVLGHHLGK	D
4051	2	33	B	0.3	3.38	0.226	0.226	gi|122513|sp|P02088.2|HBB1_MOUSE	88	R	LLGNMIVIVLGHHLGK	D
4153	2	33	B	0.21	3.16	0.231	0.231	gi|122513|sp|P02088.2|HBB1_MOUSE	88	R	LLGNMIVIVLGHHLGK	D
2119	2	33	B	3.71	2.66	0.488	0.488	gi|27574244|pdb|1O1N|A	99	K	LRVDPVNFK	L
2188	2	33	A	4.04	2.53	0.513	0.513	gi|27574244|pdb|1O1N|A	99	K	LRVDPVNFK	L
2120	2	33	B	3.47	3.05	0.278	0.278	gi|12833511|dbj|BAB22552.1|	31	R	M*FASFPTTK	T
2189	2	33	A	4.02	2.92	0.341	0.341	gi|12833511|dbj|BAB22552.1|	31	R	M*FASFPTTK	T
2197	2	33	A	4.02	2.71	0.352	0.352	gi|12833511|dbj|BAB22552.1|	31	R	M*FASFPTTK	T
4718	2	33	A	-0.9	2.86	0.336	0.336	gi|12833511|dbj|BAB22552.1|	31	R	M*FASFPTTK	T
2169	2	33	B	2.22	2.56	0.385	0.385	gi|12833511|dbj|BAB22552.1|	31	R	MFASFPTTK	T
4001	2	33	B	0.12	2.5	0.382	0.382	gi|12833511|dbj|BAB22552.1|	31	R	MFASFPTTK	T
2160	2	33	B	3.56	2.99	0.16	0.16	gi|344254270|gb|EGW10374.1|	99	K	TYFPHFDVSHGSAQVK	A
2168	2	33	B	3.5	3.7	0.135	0.135	gi|344254270|gb|EGW10374.1|	99	K	TYFPHFDVSHGSAQVK	A
3729	3	33	B	3.32	3.17	0.135	0.135	gi|344254270|gb|EGW10374.1|	99	K	TYFPHFDVSHGSAQVK	A
4118	2	33	B	0.62	2.68	0.131	0.131	gi|344254270|gb|EGW10374.1|	99	K	TYFPHFDVSHGSAQVK	A
4461	3	33	B	0.73	3.03	0.142	0.142	gi|344254270|gb|EGW10374.1|	99	K	TYFPHFDVSHGSAQVK	A
2236	2	33	A	4.31	2.65	0.146	0.146	gi|344254270|gb|EGW10374.1|	99	K	TYFPHFDVSHGSAQVK	A
2355	2	33	A	4.6	3.1	0.139	0.139	gi|344254270|gb|EGW10374.1|	99	K	TYFPHFDVSHGSAQVK	A
2359	2	33	A	4.59	3	0.162	0.162	gi|344254270|gb|EGW10374.1|	99	K	TYFPHFDVSHGSAQVK	A
4047	3	33	B	-0.1	4.25	0.942	0.942	gi|159137400|gb|ABW88847.1|	2	K	VADALATAAGHLDDLPGALSALSDLHAHK	L
4087	3	33	B	0.07	5.24	0.957	0.957	gi|159137400|gb|ABW88847.1|	2	K	VADALATAAGHLDDLPGALSALSDLHAHK	L
4094	3	33	B	-0.2	4.7	0.954	0.954	gi|159137400|gb|ABW88847.1|	2	K	VADALATAAGHLDDLPGALSALSDLHAHK	L
4229	3	33	B	1.57	4.41	0.944	0.944	gi|159137400|gb|ABW88847.1|	2	K	VADALATAAGHLDDLPGALSALSDLHAHK	L
2669	3	33	A	4.88	3.08	0.874	0.874	gi|159137400|gb|ABW88847.1|	2	K	VADALATAAGHLDDLPGALSALSDLHAHK	L
2677	3	33	A	4.92	3.65	0.913	0.913	gi|159137400|gb|ABW88847.1|	2	K	VADALATAAGHLDDLPGALSALSDLHAHK	L
4209	3	33	A	-0.8	4.28	0.922	0.922	gi|159137400|gb|ABW88847.1|	2	K	VADALATAAGHLDDLPGALSALSDLHAHK	L
4363	3	33	A	0.91	6.41	0.964	0.964	gi|159137400|gb|ABW88847.1|	2	K	VADALATAAGHLDDLPGALSALSDLHAHK	L
4368	3	33	A	0.63	6.03	0.941	0.941	gi|159137400|gb|ABW88847.1|	2	K	VADALATAAGHLDDLPGALSALSDLHAHK	L
2505	2	33	B	3.26	2.51	0.81	0.81	gi|122513|sp|P02088.2|HBB1_MOUSE	65	K	VITAFNDGLNHLDSLK	G
4048	2	33	B	-0.1	4.25	0.594	0.594	gi|122513|sp|P02088.2|HBB1_MOUSE	65	K	VITAFNDGLNHLDSLK	G

Scan	z	Sample ID	Band	PPM	XCorr	Delta correlation	Unique delta correlation	Reference	Redundancy		Peptides	
4055	2	33	B	-0.2	3.64	0.588	0.588	gi|122513|sp|P02088.2|HBB1_MOUSE	65	K	VITAFNDGLNHLDSLK	G
4164	2	33	B	0.02	3.02	0.701	0.701	gi|122513|sp|P02088.2|HBB1_MOUSE	65	K	VITAFNDGLNHLDSLK	G
4174	2	33	B	0.02	3.16	0.613	0.613	gi|122513|sp|P02088.2|HBB1_MOUSE	65	K	VITAFNDGLNHLDSLK	G
4364	2	33	B	1.71	3.69	0.708	0.708	gi|122513|sp|P02088.2|HBB1_MOUSE	65	K	VITAFNDGLNHLDSLK	G
2582	2	33	A	3.78	2.61	0.577	0.577	gi|122513|sp|P02088.2|HBB1_MOUSE	65	K	VITAFNDGLNHLDSLK	G
4163	2	33	A	3.81	3.65	0.567	0.567	gi|122513|sp|P02088.2|HBB1_MOUSE	65	K	VITAFNDGLNHLDSLK	G
4195	2	33	A	0.51	3.58	0.611	0.611	gi|122513|sp|P02088.2|HBB1_MOUSE	65	K	VITAFNDGLNHLDSLK	G
4207	2	33	A	-0.2	3.12	0.618	0.618	gi|122513|sp|P02088.2|HBB1_MOUSE	65	K	VITAFNDGLNHLDSLK	G
4330	2	33	A	0.37	2.84	0.703	0.703	gi|122513|sp|P02088.2|HBB1_MOUSE	65	K	VITAFNDGLNHLDSLK	G
625	2	33	B	-0.5	3.21	0.541	0.541	gi|241913510|pdb|3HRW|C	2	-	VLSGEDKSNIK	A
629	2	33	B	-0.5	2.99	0.549	0.549	gi|241913510|pdb|3HRW|C	2	-	VLSGEDKSNIK	A
1851	2	33	A	-0.2	2.87	0.475	0.475	gi|241913510|pdb|3HRW|C	2	-	VLSGEDKSNIK	A
1970	2	33	A	1.46	3.55	0.514	0.514	gi|241913510|pdb|3HRW|C	2	-	VLSGEDKSNIK	A
1976	2	33	A	1.55	3.46	0.514	0.514	gi|241913510|pdb|3HRW|C	2	-	VLSGEDKSNIK	A
2126	2	33	A	4.22	2.68	0.435	0.435	gi|241913510|pdb|3HRW|C	2	-	VLSGEDKSNIK	A
4717	2	33	A	-0.5	2.81	0.48	0.48	gi|241913510|pdb|3HRW|C	2	-	VLSGEDKSNIK	A
2214	2	33	B	3.05	3.38	0	0.236	gi|378548370|sp|B3EWD8.1|HBB_TAMHU	0	K	NVADEVGGEALGR^	L
2347	2	33	B	2.86	3.93	0	0.249	gi|378548370|sp|B3EWD8.1|HBB_TAMHU	0	K	NVADEVGGEALGR^	L
3250	2	33	B	4.73	3.26	0	0.326	gi|378548370|sp|B3EWD8.1|HBB_TAMHU	0	K	NVADEVGGEALGR^	L
3681	2	33	B	3.34	3.89	0	0.237	gi|378548370|sp|B3EWD8.1|HBB_TAMHU	0	K	NVADEVGGEALGR^	L
4054	2	33	B	-0.5	2.58	0	0.281	gi|378548370|sp|B3EWD8.1|HBB_TAMHU	0	K	NVADEVGGEALGR^	L
4173	2	33	B	-0.3	4.01	0	0.221	gi|378548370|sp|B3EWD8.1|HBB_TAMHU	0	K	NVADEVGGEALGR^	L
4632	2	33	B	-0.7	3.22	0	0.221	gi|378548370|sp|B3EWD8.1|HBB_TAMHU	0	K	NVADEVGGEALGR^	L
4636	2	33	B	-0.7	3.04	0	0.319	gi|378548370|sp|B3EWD8.1|HBB_TAMHU	0	K	NVADEVGGEALGR^	L
2157	2	33	A	2.42	3.25	0	0.217	gi|378548370|sp|B3EWD8.1|HBB_TAMHU	0	K	NVADEVGGEALGR^	L
2267	2	33	A	2.56	3.27	0	0.356	gi|378548370|sp|B3EWD8.1|HBB_TAMHU	0	K	NVADEVGGEALGR^	L
2273	2	33	A	3.11	3.88	0	0.352	gi|378548370|sp|B3EWD8.1|HBB_TAMHU	0	K	NVADEVGGEALGR^	L
2412	2	33	A	3.51	3.1	0	0.276	gi|378548370|sp|B3EWD8.1|HBB_TAMHU	0	K	NVADEVGGEALGR^	L
3647	2	33	A	4.97	3.94	0	0.294	gi|378548370|sp|B3EWD8.1|HBB_TAMHU	0	K	NVADEVGGEALGR^	L
3814	2	33	A	4.54	4.18	0	0.297	gi|378548370|sp|B3EWD8.1|HBB_TAMHU	0	K	NVADEVGGEALGR^	L
4462	2	33	A	-0.3	4.14	0	0.287	gi|378548370|sp|B3EWD8.1|HBB_TAMHU	0	K	NVADEVGGEALGR^	L
4466	2	33	A	-0.2	3.87	0	0.263	gi|378548370|sp|B3EWD8.1|HBB_TAMHU	0	K	NVADEVGGEALGR^	L
4691	2	33	A	-0.4	3.79	0	0.214	gi|378548370|sp|B3EWD8.1|HBB_TAMHU	0	K	NVADEVGGEALGR^	L
2122	2	33	B	2.14	3.41	0.149	0.149	gi|667267074|ref|XP_008569536.1|	4	K	VNVDDVGGEALGR	L
2204	2	33	A	3.48	3.25	0.146	0.146	gi|667267074|ref|XP_008569536.1|	4	K	VNVDDVGGEALGR	L
2065	2	33	B	4.62	2.63	0.895	0.895	gi|12847007|dbj|BAB27399.1|	60	K	VVAGVAAALAHK	Y
2191	2	33	B	2.41	3.04	0.871	0.871	gi|12847007|dbj|BAB27399.1|	60	K	VVAGVAAALAHK	Y
2193	2	33	B	2.47	3.06	0.857	0.857	gi|12847007|dbj|BAB27399.1|	60	K	VVAGVAAALAHK	Y

Scan	z	Sample ID	Band	PPM	XCorr	Delta correlation	Unique delta correlation	Reference	Redundancy		Peptides	
2315	2	33	B	3.02	3.58	0.831	0.831	gi|12847007|dbj|BAB27399.1|	60	K	VVAGVAAALAHK	Y
2327	2	33	B	3	3.21	0.854	0.854	gi|12847007|dbj|BAB27399.1|	60	K	VVAGVAAALAHK	Y
3597	2	33	B	3.07	3.24	0.895	0.895	gi|12847007|dbj|BAB27399.1|	60	K	VVAGVAAALAHK	Y
3607	2	33	B	2.85	3.61	0.896	0.896	gi|12847007|dbj|BAB27399.1|	60	K	VVAGVAAALAHK	Y
3731	2	33	B	4.48	3.47	0.913	0.913	gi|12847007|dbj|BAB27399.1|	60	K	VVAGVAAALAHK	Y
3734	2	33	B	2.24	3.63	0.911	0.911	gi|12847007|dbj|BAB27399.1|	60	K	VVAGVAAALAHK	Y
3859	2	33	B	3.75	3.42	0.904	0.904	gi|12847007|dbj|BAB27399.1|	60	K	VVAGVAAALAHK	Y
3862	2	33	B	3.89	3.45	0.905	0.905	gi|12847007|dbj|BAB27399.1|	60	K	VVAGVAAALAHK	Y
3984	2	33	B	2.58	3.5	0.91	0.91	gi|12847007|dbj|BAB27399.1|	60	K	VVAGVAAALAHK	Y
3988	2	33	B	2.2	3.29	0.911	0.911	gi|12847007|dbj|BAB27399.1|	60	K	VVAGVAAALAHK	Y
4098	2	33	B	-0.1	3.95	0.868	0.868	gi|12847007|dbj|BAB27399.1|	60	K	VVAGVAAALAHK	Y
4106	2	33	B	-0.1	3.65	0.898	0.898	gi|12847007|dbj|BAB27399.1|	60	K	VVAGVAAALAHK	Y
4216	2	33	B	-0.2	4.04	0.925	0.925	gi|12847007|dbj|BAB27399.1|	60	K	VVAGVAAALAHK	Y
4220	2	33	B	-0.2	3.96	0.89	0.89	gi|12847007|dbj|BAB27399.1|	60	K	VVAGVAAALAHK	Y
4336	2	33	B	-0.1	3.79	0.906	0.906	gi|12847007|dbj|BAB27399.1|	60	K	VVAGVAAALAHK	Y
4343	2	33	B	-0.1	3.48	0.893	0.893	gi|12847007|dbj|BAB27399.1|	60	K	VVAGVAAALAHK	Y
4452	2	33	B	-0.1	3.55	0.884	0.884	gi|12847007|dbj|BAB27399.1|	60	K	VVAGVAAALAHK	Y
4456	2	33	B	-0.1	3.83	0.895	0.895	gi|12847007|dbj|BAB27399.1|	60	K	VVAGVAAALAHK	Y
4566	2	33	B	-0.6	3.03	0.942	0.942	gi|12847007|dbj|BAB27399.1|	60	K	VVAGVAAALAHK	Y
4576	2	33	B	-0.6	3.36	0.889	0.889	gi|12847007|dbj|BAB27399.1|	60	K	VVAGVAAALAHK	Y
2158	2	33	A	4.21	3.09	0.814	0.814	gi|12847007|dbj|BAB27399.1|	60	K	VVAGVAAALAHK	Y
2161	2	33	A	4.21	3.1	0.859	0.859	gi|12847007|dbj|BAB27399.1|	60	K	VVAGVAAALAHK	Y
2275	2	33	A	3.03	3.45	0.908	0.908	gi|12847007|dbj|BAB27399.1|	60	K	VVAGVAAALAHK	Y
2277	2	33	A	3.06	3.25	0.898	0.898	gi|12847007|dbj|BAB27399.1|	60	K	VVAGVAAALAHK	Y
2423	2	33	A	3.7	3.95	0.88	0.88	gi|12847007|dbj|BAB27399.1|	60	K	VVAGVAAALAHK	Y
2442	2	33	A	3.63	3.73	0.962	0.962	gi|12847007|dbj|BAB27399.1|	60	K	VVAGVAAALAHK	Y
4199	2	33	A	-0.3	3.05	0.948	0.948	gi|12847007|dbj|BAB27399.1|	60	K	VVAGVAAALAHK	Y
4211	2	33	A	-0.6	3.4	0.923	0.923	gi|12847007|dbj|BAB27399.1|	60	K	VVAGVAAALAHK	Y
4332	2	33	A	0	2.62	0.947	0.947	gi|12847007|dbj|BAB27399.1|	60	K	VVAGVAAALAHK	Y
4474	2	33	A	0.68	3.15	0.939	0.939	gi|12847007|dbj|BAB27399.1|	60	K	VVAGVAAALAHK	Y
4484	2	33	A	0.04	3.12	0.957	0.957	gi|12847007|dbj|BAB27399.1|	60	K	VVAGVAAALAHK	Y
4590	2	33	A	-0.6	3.36	0.887	0.887	gi|12847007|dbj|BAB27399.1|	60	K	VVAGVAAALAHK	Y
4598	2	33	A	-0.6	3.19	0.864	0.864	gi|12847007|dbj|BAB27399.1|	60	K	VVAGVAAALAHK	Y
2108	2	33	B	4.36	4.46	0.919	0.919	gi|12847007|dbj|BAB27399.1|	60	K	VVAGVAAALAHKYH	-
2262	2	33	B	2.48	4.05	0.951	0.951	gi|12847007|dbj|BAB27399.1|	60	K	VVAGVAAALAHKYH	-
2273	2	33	B	2.39	3.59	0.949	0.949	gi|12847007|dbj|BAB27399.1|	60	K	VVAGVAAALAHKYH	-
4069	2	33	B	-0.1	3.37	0.954	0.954	gi|12847007|dbj|BAB27399.1|	60	K	VVAGVAAALAHKYH	-
4079	2	33	B	-0	3.1	0.96	0.96	gi|12847007|dbj|BAB27399.1|	60	K	VVAGVAAALAHKYH	-

Scan	z	Sample ID	Band	PPM	XCorr	Delta correlation	Unique delta correlation	Reference	Redundancy		Peptides	
4349	2	33	B	0.26	2.94	0.923	0.923	gi|12847007|dbj|BAB27399.1|	60	K	VVAGVAAALAHKYH	-
4378	2	33	B	-0.2	3.16	0.943	0.943	gi|12847007|dbj|BAB27399.1|	60	K	VVAGVAAALAHKYH	-
4488	2	33	B	-0.3	3	0.953	0.953	gi|12847007|dbj|BAB27399.1|	60	K	VVAGVAAALAHKYH	-
4492	2	33	B	-0.4	3.14	0.957	0.957	gi|12847007|dbj|BAB27399.1|	60	K	VVAGVAAALAHKYH	-
2172	2	33	A	4.9	4.56	0.913	0.913	gi|12847007|dbj|BAB27399.1|	60	K	VVAGVAAALAHKYH	-
2178	2	33	A	4.88	4.07	0.866	0.866	gi|12847007|dbj|BAB27399.1|	60	K	VVAGVAAALAHKYH	-
2294	2	33	A	3.55	4.34	0.915	0.915	gi|12847007|dbj|BAB27399.1|	60	K	VVAGVAAALAHKYH	-
2299	2	33	A	3.68	4.29	0.93	0.93	gi|12847007|dbj|BAB27399.1|	60	K	VVAGVAAALAHKYH	-
2420	2	33	A	4.03	4.39	0.945	0.945	gi|12847007|dbj|BAB27399.1|	60	K	VVAGVAAALAHKYH	-
2429	2	33	A	3.93	4.15	0.949	0.949	gi|12847007|dbj|BAB27399.1|	60	K	VVAGVAAALAHKYH	-
2557	2	33	A	2.91	2.53	0.886	0.886	gi|12847007|dbj|BAB27399.1|	60	K	VVAGVAAALAHKYH	-
4183	2	33	A	0.31	2.66	0.909	0.909	gi|12847007|dbj|BAB27399.1|	60	K	VVAGVAAALAHKYH	-
4196	2	33	A	0	3.15	0.949	0.949	gi|12847007|dbj|BAB27399.1|	60	K	VVAGVAAALAHKYH	-
4303	2	33	A	-0	2.81	0.949	0.949	gi|12847007|dbj|BAB27399.1|	60	K	VVAGVAAALAHKYH	-
4339	2	33	A	0.58	3.16	0.927	0.927	gi|12847007|dbj|BAB27399.1|	60	K	VVAGVAAALAHKYH	-
4358	2	33	A	0.68	3.18	0.944	0.944	gi|12847007|dbj|BAB27399.1|	60	K	VVAGVAAALAHKYH	-
4494	2	33	A	0.22	3.49	0.953	0.953	gi|12847007|dbj|BAB27399.1|	60	K	VVAGVAAALAHKYH	-
4513	2	33	A	0.18	3.61	0.961	0.961	gi|12847007|dbj|BAB27399.1|	60	K	VVAGVAAALAHKYH	-
4622	2	33	A	-0.3	3.29	0.944	0.944	gi|12847007|dbj|BAB27399.1|	60	K	VVAGVAAALAHKYH	-
4629	2	33	A	-0.4	3.28	0.944	0.944	gi|12847007|dbj|BAB27399.1|	60	K	VVAGVAAALAHKYH	-
2327	2	33	A	3.2	2.55	0.889	0.889	gi|591333109|ref|XP_007092291.1|	23	K	VVAGVASALAHKYH	-
2438	3	33	B	2.88	5.95	0.406	0.406	gi|187369324|dbj|BAG31393.1|	73	R	YFDSFGDLSSASAIM*GNAK	V
2765	2	33	B	3.47	3.78	0.588	0.588	gi|187369324|dbj|BAG31393.1|	73	R	YFDSFGDLSSASAIM*GNAK	V
2802	2	33	B	3.49	3.91	0.572	0.572	gi|187369324|dbj|BAG31393.1|	73	R	YFDSFGDLSSASAIM*GNAK	V
4003	2	33	B	-0	3.88	0.572	0.572	gi|187369324|dbj|BAG31393.1|	73	R	YFDSFGDLSSASAIM*GNAK	V
4033	2	33	B	0.18	3.09	0.571	0.571	gi|187369324|dbj|BAG31393.1|	73	R	YFDSFGDLSSASAIM*GNAK	V
4049	2	33	B	-0	4.53	0.544	0.544	gi|187369324|dbj|BAG31393.1|	73	R	YFDSFGDLSSASAIM*GNAK	V
4152	2	33	B	0.48	2.84	0.702	0.702	gi|187369324|dbj|BAG31393.1|	73	R	YFDSFGDLSSASAIM*GNAK	V
4158	2	33	B	0.44	4.05	0.585	0.585	gi|187369324|dbj|BAG31393.1|	73	R	YFDSFGDLSSASAIM*GNAK	V
4353	2	33	B	-0	4.19	0.587	0.587	gi|187369324|dbj|BAG31393.1|	73	R	YFDSFGDLSSASAIM*GNAK	V
4363	2	33	B	-0.1	5	0.586	0.586	gi|187369324|dbj|BAG31393.1|	73	R	YFDSFGDLSSASAIM*GNAK	V
4370	3	33	B	-0.4	3.7	0.445	0.445	gi|187369324|dbj|BAG31393.1|	73	R	YFDSFGDLSSASAIM*GNAK	V
4392	3	33	B	-0.8	4.2	0.485	0.485	gi|187369324|dbj|BAG31393.1|	73	R	YFDSFGDLSSASAIM*GNAK	V
2509	2	33	A	4.2	4.45	0.574	0.574	gi|187369324|dbj|BAG31393.1|	73	R	YFDSFGDLSSASAIM*GNAK	V
2511	2	33	A	4.41	4.78	0.553	0.553	gi|187369324|dbj|BAG31393.1|	73	R	YFDSFGDLSSASAIM*GNAK	V
2845	2	33	A	3.96	4.52	0.622	0.622	gi|187369324|dbj|BAG31393.1|	73	R	YFDSFGDLSSASAIM*GNAK	V
2877	2	33	A	4.2	3.71	0.635	0.635	gi|187369324|dbj|BAG31393.1|	73	R	YFDSFGDLSSASAIM*GNAK	V
2904	2	33	A	4.73	3.84	0.673	0.673	gi|187369324|dbj|BAG31393.1|	73	R	YFDSFGDLSSASAIM*GNAK	V

Scan	z	Sample ID	Band	PPM	XCorr	Delta correlation	Unique delta correlation	Reference	Redundancy		Peptides	
2914	2	33	A	4.79	3.64	0.638	0.638	gi|187369324|dbj|BAG31393.1|	73	R	YFDSFGDLSSASAIM*GNAK	V
4131	2	33	A	2.97	4.43	0.559	0.559	gi|187369324|dbj|BAG31393.1|	73	R	YFDSFGDLSSASAIM*GNAK	V
4133	3	33	A	1.82	3.24	0.364	0.364	gi|187369324|dbj|BAG31393.1|	73	R	YFDSFGDLSSASAIM*GNAK	V
4272	2	33	A	0.14	3.38	0.697	0.697	gi|187369324|dbj|BAG31393.1|	73	R	YFDSFGDLSSASAIM*GNAK	V
4284	2	33	A	0.06	4.15	0.563	0.563	gi|187369324|dbj|BAG31393.1|	73	R	YFDSFGDLSSASAIM*GNAK	V
4297	3	33	A	-0.6	3.63	0.399	0.399	gi|187369324|dbj|BAG31393.1|	73	R	YFDSFGDLSSASAIM*GNAK	V
4454	2	33	A	0.54	4.13	0.651	0.651	gi|187369324|dbj|BAG31393.1|	73	R	YFDSFGDLSSASAIM*GNAK	V
4468	2	33	A	0.04	4.26	0.594	0.594	gi|187369324|dbj|BAG31393.1|	73	R	YFDSFGDLSSASAIM*GNAK	V
4504	3	33	A	-1	3.46	0.356	0.356	gi|187369324|dbj|BAG31393.1|	73	R	YFDSFGDLSSASAIM*GNAK	V
4516	3	33	A	-1	4.98	0.493	0.493	gi|187369324|dbj|BAG31393.1|	73	R	YFDSFGDLSSASAIM*GNAK	V
4578	2	33	A	0.3	3.38	0.59	0.59	gi|187369324|dbj|BAG31393.1|	73	R	YFDSFGDLSSASAIM*GNAK	V
4584	2	33	A	0.33	4.34	0.619	0.619	gi|187369324|dbj|BAG31393.1|	73	R	YFDSFGDLSSASAIM*GNAK	V
2522	2	33	B	4.23	5.06	0.583	0.583	gi|187369324|dbj|BAG31393.1|	73	R	YFDSFGDLSSASAIMGNAK	V
2542	2	33	B	4.26	5.49	0.536	0.536	gi|187369324|dbj|BAG31393.1|	73	R	YFDSFGDLSSASAIMGNAK	V
2653	2	33	B	3.8	5.81	0.549	0.549	gi|187369324|dbj|BAG31393.1|	73	R	YFDSFGDLSSASAIMGNAK	V
2655	2	33	B	3.95	5.95	0.562	0.562	gi|187369324|dbj|BAG31393.1|	73	R	YFDSFGDLSSASAIMGNAK	V
2777	2	33	B	3.92	5.72	0.568	0.568	gi|187369324|dbj|BAG31393.1|	73	R	YFDSFGDLSSASAIMGNAK	V
2797	2	33	B	3.62	5.64	0.59	0.59	gi|187369324|dbj|BAG31393.1|	73	R	YFDSFGDLSSASAIMGNAK	V
2916	2	33	B	4.5	5.58	0.598	0.598	gi|187369324|dbj|BAG31393.1|	73	R	YFDSFGDLSSASAIMGNAK	V
2918	2	33	B	4.52	5.37	0.626	0.626	gi|187369324|dbj|BAG31393.1|	73	R	YFDSFGDLSSASAIMGNAK	V
3594	2	33	B	4.17	4.05	0.632	0.632	gi|187369324|dbj|BAG31393.1|	73	R	YFDSFGDLSSASAIMGNAK	V
3596	2	33	B	3.92	4.18	0.628	0.628	gi|187369324|dbj|BAG31393.1|	73	R	YFDSFGDLSSASAIMGNAK	V
3652	3	33	B	3.77	4.09	0.294	0.294	gi|187369324|dbj|BAG31393.1|	73	R	YFDSFGDLSSASAIMGNAK	V
3659	3	33	B	3.65	4.15	0.333	0.333	gi|187369324|dbj|BAG31393.1|	73	R	YFDSFGDLSSASAIMGNAK	V
3719	2	33	B	4.82	5.61	0.578	0.578	gi|187369324|dbj|BAG31393.1|	73	R	YFDSFGDLSSASAIMGNAK	V
3723	2	33	B	4.72	3.54	0.615	0.615	gi|187369324|dbj|BAG31393.1|	73	R	YFDSFGDLSSASAIMGNAK	V
3781	3	33	B	3.86	4.14	0.303	0.303	gi|187369324|dbj|BAG31393.1|	73	R	YFDSFGDLSSASAIMGNAK	V
3797	3	33	B	4.77	4.33	0.342	0.342	gi|187369324|dbj|BAG31393.1|	73	R	YFDSFGDLSSASAIMGNAK	V
3922	3	33	B	4.43	4.24	0.332	0.332	gi|187369324|dbj|BAG31393.1|	73	R	YFDSFGDLSSASAIMGNAK	V
3927	3	33	B	3.79	3.81	0.319	0.319	gi|187369324|dbj|BAG31393.1|	73	R	YFDSFGDLSSASAIMGNAK	V
3969	2	33	B	4.07	5.41	0.534	0.534	gi|187369324|dbj|BAG31393.1|	73	R	YFDSFGDLSSASAIMGNAK	V
3971	2	33	B	3.75	5.39	0.552	0.552	gi|187369324|dbj|BAG31393.1|	73	R	YFDSFGDLSSASAIMGNAK	V
4039	3	33	B	-0.5	4.4	0.331	0.331	gi|187369324|dbj|BAG31393.1|	73	R	YFDSFGDLSSASAIMGNAK	V
4082	2	33	B	0.27	6.38	0.518	0.518	gi|187369324|dbj|BAG31393.1|	73	R	YFDSFGDLSSASAIMGNAK	V
4091	2	33	B	0.3	6.17	0.573	0.573	gi|187369324|dbj|BAG31393.1|	73	R	YFDSFGDLSSASAIMGNAK	V
4151	3	33	B	-0.4	3.29	0.331	0.331	gi|187369324|dbj|BAG31393.1|	73	R	YFDSFGDLSSASAIMGNAK	V
4156	3	33	B	-0.5	4.33	0.361	0.361	gi|187369324|dbj|BAG31393.1|	73	R	YFDSFGDLSSASAIMGNAK	V
4202	2	33	B	0.22	6.54	0.522	0.522	gi|187369324|dbj|BAG31393.1|	73	R	YFDSFGDLSSASAIMGNAK	V

Scan	z	Sample ID	Band	PPM	XCorr	Delta correlation	Unique delta correlation	Reference	Redundancy		Peptides	
4208	2	33	B	0.17	6.23	0.54	0.54	gi|187369324|dbj|BAG31393.1|	73	R	YFDSFGDLSSASAIMGNAK	V
4268	3	33	B	4.07	3.14	0.362	0.362	gi|187369324|dbj|BAG31393.1|	73	R	YFDSFGDLSSASAIMGNAK	V
4273	3	33	B	3.88	3.83	0.328	0.328	gi|187369324|dbj|BAG31393.1|	73	R	YFDSFGDLSSASAIMGNAK	V
4325	2	33	B	0.4	3.64	0.692	0.692	gi|187369324|dbj|BAG31393.1|	73	R	YFDSFGDLSSASAIMGNAK	V
4329	2	33	B	0.38	4.19	0.646	0.646	gi|187369324|dbj|BAG31393.1|	73	R	YFDSFGDLSSASAIMGNAK	V
4397	3	33	B	-0.3	4.09	0.322	0.322	gi|187369324|dbj|BAG31393.1|	73	R	YFDSFGDLSSASAIMGNAK	V
4407	3	33	B	-0.4	3.56	0.32	0.32	gi|187369324|dbj|BAG31393.1|	73	R	YFDSFGDLSSASAIMGNAK	V
4437	2	33	B	0.23	5.06	0.618	0.618	gi|187369324|dbj|BAG31393.1|	73	R	YFDSFGDLSSASAIMGNAK	V
4445	2	33	B	0.23	3.75	0.611	0.611	gi|187369324|dbj|BAG31393.1|	73	R	YFDSFGDLSSASAIMGNAK	V
2608	2	33	A	3.93	2.7	0.745	0.745	gi|187369324|dbj|BAG31393.1|	73	R	YFDSFGDLSSASAIMGNAK	V
2634	2	33	A	4.04	4.93	0.644	0.644	gi|187369324|dbj|BAG31393.1|	73	R	YFDSFGDLSSASAIMGNAK	V
2643	2	33	A	3.68	4.54	0.631	0.631	gi|187369324|dbj|BAG31393.1|	73	R	YFDSFGDLSSASAIMGNAK	V
2871	2	33	A	4.98	4.96	0.627	0.627	gi|187369324|dbj|BAG31393.1|	73	R	YFDSFGDLSSASAIMGNAK	V
2884	2	33	A	4.85	5.13	0.575	0.575	gi|187369324|dbj|BAG31393.1|	73	R	YFDSFGDLSSASAIMGNAK	V
3541	3	33	A	0.14	3.73	0.352	0.352	gi|187369324|dbj|BAG31393.1|	73	R	YFDSFGDLSSASAIMGNAK	V
3564	3	33	A	4.76	3.71	0.322	0.322	gi|187369324|dbj|BAG31393.1|	73	R	YFDSFGDLSSASAIMGNAK	V
3702	3	33	A	4.83	3.59	0.3	0.3	gi|187369324|dbj|BAG31393.1|	73	R	YFDSFGDLSSASAIMGNAK	V
3839	3	33	A	4.83	4.21	0.355	0.355	gi|187369324|dbj|BAG31393.1|	73	R	YFDSFGDLSSASAIMGNAK	V
3983	3	33	A	4.2	4.55	0.281	0.281	gi|187369324|dbj|BAG31393.1|	73	R	YFDSFGDLSSASAIMGNAK	V
4111	3	33	A	3.47	3.53	0.293	0.293	gi|187369324|dbj|BAG31393.1|	73	R	YFDSFGDLSSASAIMGNAK	V
4114	3	33	A	3.43	3.42	0.283	0.283	gi|187369324|dbj|BAG31393.1|	73	R	YFDSFGDLSSASAIMGNAK	V
4159	2	33	A	4.46	5.33	0.549	0.549	gi|187369324|dbj|BAG31393.1|	73	R	YFDSFGDLSSASAIMGNAK	V
4161	2	33	A	4.48	5.64	0.595	0.595	gi|187369324|dbj|BAG31393.1|	73	R	YFDSFGDLSSASAIMGNAK	V
4233	3	33	A	-0.3	3.66	0.328	0.328	gi|187369324|dbj|BAG31393.1|	73	R	YFDSFGDLSSASAIMGNAK	V
4239	3	33	A	-0.3	5.07	0.349	0.349	gi|187369324|dbj|BAG31393.1|	73	R	YFDSFGDLSSASAIMGNAK	V
4271	2	33	A	0.3	5.61	0.54	0.54	gi|187369324|dbj|BAG31393.1|	73	R	YFDSFGDLSSASAIMGNAK	V
4281	2	33	A	0.29	6.08	0.552	0.552	gi|187369324|dbj|BAG31393.1|	73	R	YFDSFGDLSSASAIMGNAK	V
4355	3	33	A	0.31	4.24	0.353	0.353	gi|187369324|dbj|BAG31393.1|	73	R	YFDSFGDLSSASAIMGNAK	V
4361	3	33	A	1.27	3.82	0.312	0.312	gi|187369324|dbj|BAG31393.1|	73	R	YFDSFGDLSSASAIMGNAK	V
4389	2	33	A	4.01	5.23	0.577	0.577	gi|187369324|dbj|BAG31393.1|	73	R	YFDSFGDLSSASAIMGNAK	V
4391	2	33	A	4.59	5.33	0.604	0.604	gi|187369324|dbj|BAG31393.1|	73	R	YFDSFGDLSSASAIMGNAK	V
4488	3	33	A	-0.1	5.21	0.337	0.337	gi|187369324|dbj|BAG31393.1|	73	R	YFDSFGDLSSASAIMGNAK	V
4499	3	33	A	-0.2	5.44	0.346	0.346	gi|187369324|dbj|BAG31393.1|	73	R	YFDSFGDLSSASAIMGNAK	V
4510	2	33	A	0.43	6.37	0.536	0.536	gi|187369324|dbj|BAG31393.1|	73	R	YFDSFGDLSSASAIMGNAK	V
4521	2	33	A	0.55	5.95	0.586	0.586	gi|187369324|dbj|BAG31393.1|	73	R	YFDSFGDLSSASAIMGNAK	V
4185	2	34	B	-0.3	2.69	0.984	0.984	gi|554577472|ref|XP_005880462.1|	74	K	AAVSGLWGK	V
4165	2	34	A	-0.3	2.75	0.969	0.969	gi|554577472|ref|XP_005880462.1|	74	K	AAVSGLWGK	V
4283	2	34	A	-0.2	2.78	0.985	0.985	gi|554577472|ref|XP_005880462.1|	74	K	AAVSGLWGK	V

↳

Scan	z	Sample ID	Band	PPM	XCorr	Delta correlation	Unique delta correlation	Reference	Redundancy		Peptides	
4289	2	34	A	-0.1	2.72	0.981	0.981	gi|554577472|ref|XP_005880462.1|	74	K	AAVSGLWGK	V
4403	2	34	A	-0.4	2.69	0.972	0.972	gi|554577472|ref|XP_005880462.1|	74	K	AAVSGLWGK	V
4406	2	34	A	-0.3	2.77	0.983	0.983	gi|554577472|ref|XP_005880462.1|	74	K	AAVSGLWGK	V
4521	2	34	A	0.4	2.53	0.902	0.902	gi|554577472|ref|XP_005880462.1|	74	K	AAVSGLWGK	V
2311	2	34	B	-0.5	3.79	0.951	0.951	gi|532054982|ref|XP_005370208.1|	94	K	DFTPAAQAAFQK	V
4546	2	34	A	-0.5	2.56	0.9	0.9	gi|532054982|ref|XP_005370208.1|	94	K	DFTPAAQAAFQK	V
4549	2	34	A	-0.5	2.67	0.904	0.904	gi|532054982|ref|XP_005370208.1|	94	K	DFTPAAQAAFQK	V
2519	2	34	B	3.38	3.5	0	0.355	gi|27574244|pdb|1O1N|A	0	K	FLASVSTVLTSK	Y
2523	2	34	B	0.34	4.05	0	0.312	gi|27574244|pdb|1O1N|A	0	K	FLASVSTVLTSK	Y
2898	2	34	B	3.19	3.44	0	0.376	gi|27574244|pdb|1O1N|A	0	K	FLASVSTVLTSK	Y
2906	2	34	B	3.19	2.85	0	0.325	gi|27574244|pdb|1O1N|A	0	K	FLASVSTVLTSK	Y
3020	2	34	B	3.66	3.25	0	0.286	gi|27574244|pdb|1O1N|A	0	K	FLASVSTVLTSK	Y
3027	2	34	B	3.63	3.2	0	0.307	gi|27574244|pdb|1O1N|A	0	K	FLASVSTVLTSK	Y
3160	2	34	B	3.94	2.93	0	0.306	gi|27574244|pdb|1O1N|A	0	K	FLASVSTVLTSK	Y
3162	2	34	B	4.11	2.66	0	0.306	gi|27574244|pdb|1O1N|A	0	K	FLASVSTVLTSK	Y
3296	2	34	B	3.78	2.7	0	0.333	gi|27574244|pdb|1O1N|A	0	K	FLASVSTVLTSK	Y
3299	2	34	B	3.89	2.6	0	0.384	gi|27574244|pdb|1O1N|A	0	K	FLASVSTVLTSK	Y
3429	2	34	B	3.78	2.91	0	0.27	gi|27574244|pdb|1O1N|A	0	K	FLASVSTVLTSK	Y
3431	2	34	B	3.9	2.64	0	0.34	gi|27574244|pdb|1O1N|A	0	K	FLASVSTVLTSK	Y
3558	2	34	B	4.18	2.73	0	0.302	gi|27574244|pdb|1O1N|A	0	K	FLASVSTVLTSK	Y
3560	2	34	B	4	3.15	0	0.293	gi|27574244|pdb|1O1N|A	0	K	FLASVSTVLTSK	Y
3685	2	34	B	3.79	3.17	0	0.327	gi|27574244|pdb|1O1N|A	0	K	FLASVSTVLTSK	Y
3688	2	34	B	3.8	3.09	0	0.333	gi|27574244|pdb|1O1N|A	0	K	FLASVSTVLTSK	Y
3812	2	34	B	4.83	2.63	0	0.281	gi|27574244|pdb|1O1N|A	0	K	FLASVSTVLTSK	Y
3814	2	34	B	4.78	2.73	0	0.289	gi|27574244|pdb|1O1N|A	0	K	FLASVSTVLTSK	Y
3937	2	34	B	4.89	2.56	0	0.256	gi|27574244|pdb|1O1N|A	0	K	FLASVSTVLTSK	Y
3939	2	34	B	4.84	2.96	0	0.296	gi|27574244|pdb|1O1N|A	0	K	FLASVSTVLTSK	Y
4061	2	34	B	3.39	2.95	0	0.287	gi|27574244|pdb|1O1N|A	0	K	FLASVSTVLTSK	Y
4066	2	34	B	3.38	3.13	0	0.277	gi|27574244|pdb|1O1N|A	0	K	FLASVSTVLTSK	Y
4175	2	34	B	0.11	2.97	0	0.346	gi|27574244|pdb|1O1N|A	0	K	FLASVSTVLTSK	Y
4181	2	34	B	0.04	3.04	0	0.338	gi|27574244|pdb|1O1N|A	0	K	FLASVSTVLTSK	Y
4290	2	34	B	3.25	3.08	0	0.337	gi|27574244|pdb|1O1N|A	0	K	FLASVSTVLTSK	Y
4294	2	34	B	2.49	3.05	0	0.331	gi|27574244|pdb|1O1N|A	0	K	FLASVSTVLTSK	Y
4408	2	34	B	0.13	3.13	0	0.319	gi|27574244|pdb|1O1N|A	0	K	FLASVSTVLTSK	Y
4414	2	34	B	0.21	3.71	0	0.297	gi|27574244|pdb|1O1N|A	0	K	FLASVSTVLTSK	Y
4530	2	34	B	-0	2.88	0	0.368	gi|27574244|pdb|1O1N|A	0	K	FLASVSTVLTSK	Y
3498	2	34	A	3.43	2.63	0	0.295	gi|27574244|pdb|1O1N|A	0	K	FLASVSTVLTSK	Y
3631	2	34	A	4.02	2.52	0	0.308	gi|27574244|pdb|1O1N|A	0	K	FLASVSTVLTSK	Y

Scan	z	Sample ID	Band	PPM	XCorr	Delta correlation	Unique delta correlation	Reference	Redundancy		Peptides	
3761	2	34	A	4.67	2.68	0	0.304	gi|27574244|pdb|1O1N|A	0	K	FLASVSTVLTSK	Y
3763	2	34	A	4.89	2.52	0	0.325	gi|27574244|pdb|1O1N|A	0	K	FLASVSTVLTSK	Y
3892	2	34	A	4.15	2.68	0	0.387	gi|27574244|pdb|1O1N|A	0	K	FLASVSTVLTSK	Y
3894	2	34	A	4.1	2.63	0	0.306	gi|27574244|pdb|1O1N|A	0	K	FLASVSTVLTSK	Y
4021	2	34	A	3.9	2.83	0	0.289	gi|27574244|pdb|1O1N|A	0	K	FLASVSTVLTSK	Y
4023	2	34	A	3.86	2.66	0	0.303	gi|27574244|pdb|1O1N|A	0	K	FLASVSTVLTSK	Y
4139	2	34	A	0.03	3.05	0	0.351	gi|27574244|pdb|1O1N|A	0	K	FLASVSTVLTSK	Y
4142	2	34	A	-0.1	2.83	0	0.322	gi|27574244|pdb|1O1N|A	0	K	FLASVSTVLTSK	Y
4253	2	34	A	0.18	2.94	0	0.372	gi|27574244|pdb|1O1N|A	0	K	FLASVSTVLTSK	Y
4255	2	34	A	0.23	3.15	0	0.333	gi|27574244|pdb|1O1N|A	0	K	FLASVSTVLTSK	Y
4371	2	34	A	2.52	3.12	0	0.366	gi|27574244|pdb|1O1N|A	0	K	FLASVSTVLTSK	Y
4374	2	34	A	3.13	2.78	0	0.368	gi|27574244|pdb|1O1N|A	0	K	FLASVSTVLTSK	Y
4485	2	34	A	0.48	3.14	0	0.359	gi|27574244|pdb|1O1N|A	0	K	FLASVSTVLTSK	Y
4494	2	34	A	0.49	3.47	0	0.315	gi|27574244|pdb|1O1N|A	0	K	FLASVSTVLTSK	Y
2646	2	34	B	2.67	3.59	0.921	0.921	gi|56967333|pdb|1Y0D|C	4	K	FLASVSTVLTSKY	-
2655	2	34	B	2.67	2.7	0.899	0.899	gi|56967333|pdb|1Y0D|C	4	K	FLASVSTVLTSKY	-
3524	2	34	B	3.66	3.75	0.108	0.108	gi|344254270|gb|EGW10374.1|	99	K	IGGHGAEYGAEALER	M
3680	2	34	B	4.24	4.16	0.102	0.102	gi|344254270|gb|EGW10374.1|	99	K	IGGHGAEYGAEALER	M
4055	2	34	B	3.63	3.96	0.1	0.1	gi|344254270|gb|EGW10374.1|	99	K	IGGHGAEYGAEALER	M
4059	2	34	B	3.56	3.91	0.11	0.11	gi|344254270|gb|EGW10374.1|	99	K	IGGHGAEYGAEALER	M
4169	2	34	B	-0.1	3.83	0.104	0.104	gi|344254270|gb|EGW10374.1|	99	K	IGGHGAEYGAEALER	M
4178	2	34	B	-0.1	4.07	0.104	0.104	gi|344254270|gb|EGW10374.1|	99	K	IGGHGAEYGAEALER	M
4286	2	34	B	0.32	3.92	0.105	0.105	gi|344254270|gb|EGW10374.1|	99	K	IGGHGAEYGAEALER	M
4076	2	34	A	4.31	3.36	0.121	0.121	gi|344254270|gb|EGW10374.1|	99	K	IGGHGAEYGAEALER	M
4089	2	34	A	4.49	3.1	0.105	0.105	gi|344254270|gb|EGW10374.1|	99	K	IGGHGAEYGAEALER	M
4335	2	34	A	3.92	3.46	0.114	0.114	gi|344254270|gb|EGW10374.1|	99	K	IGGHGAEYGAEALER	M
4357	2	34	A	4.35	3.81	0.108	0.108	gi|344254270|gb|EGW10374.1|	99	K	IGGHGAEYGAEALER	M
4119	2	34	B	0.98	3.07	0.725	0.725	gi|122513|sp|P02088.2|HBB1_MOUSE	65	K	KVITAFNDGLNHLDSLK	G
4139	2	34	B	-2.1	2.58	0.694	0.694	gi|122513|sp|P02088.2|HBB1_MOUSE	65	K	KVITAFNDGLNHLDSLK	G
4247	2	34	B	-0.2	2.77	0.623	0.623	gi|122513|sp|P02088.2|HBB1_MOUSE	65	K	KVITAFNDGLNHLDSLK	G
4250	2	34	B	0.08	3.14	0.657	0.657	gi|122513|sp|P02088.2|HBB1_MOUSE	65	K	KVITAFNDGLNHLDSLK	G
4426	2	34	B	0.49	2.5	0.677	0.677	gi|122513|sp|P02088.2|HBB1_MOUSE	65	K	KVITAFNDGLNHLDSLK	G
2407	3	34	A	3.87	3.5	0.573	0.573	gi|122513|sp|P02088.2|HBB1_MOUSE	65	K	KVITAFNDGLNHLDSLK	G
4479	3	34	A	0.75	3.1	0.499	0.499	gi|122513|sp|P02088.2|HBB1_MOUSE	65	K	KVITAFNDGLNHLDSLK	G
4499	3	34	A	0.86	3.23	0.503	0.503	gi|122513|sp|P02088.2|HBB1_MOUSE	65	K	KVITAFNDGLNHLDSLK	G
2688	2	34	B	4.25	4.5	0.167	0.167	gi|122513|sp|P02088.2|HBB1_MOUSE	88	R	LLGNMIVIVLGHHLGK	D
2690	2	34	B	4.29	4.16	0.188	0.188	gi|122513|sp|P02088.2|HBB1_MOUSE	88	R	LLGNMIVIVLGHHLGK	D
2807	2	34	B	3.92	3.57	0.187	0.187	gi|122513|sp|P02088.2|HBB1_MOUSE	88	R	LLGNMIVIVLGHHLGK	D

Scan	z	Sample ID	Band	PPM	XCorr	Delta correlation	Unique delta correlation	Reference	Redundancy		Peptides	
2810	2	34	B	3.89	3.88	0.212	0.212	gi|122513|sp|P02088.2|HBB1_MOUSE	88	R	LLGNMIVIVLGHHLGK	D
2986	2	34	B	3.98	3.55	0.219	0.219	gi|122513|sp|P02088.2|HBB1_MOUSE	88	R	LLGNMIVIVLGHHLGK	D
3324	2	34	B	4.73	3.43	0.207	0.207	gi|122513|sp|P02088.2|HBB1_MOUSE	88	R	LLGNMIVIVLGHHLGK	D
3327	2	34	B	4.71	3.36	0.203	0.203	gi|122513|sp|P02088.2|HBB1_MOUSE	88	R	LLGNMIVIVLGHHLGK	D
3457	2	34	B	4.8	2.69	0.21	0.21	gi|122513|sp|P02088.2|HBB1_MOUSE	88	R	LLGNMIVIVLGHHLGK	D
3461	2	34	B	4.96	3.38	0.191	0.191	gi|122513|sp|P02088.2|HBB1_MOUSE	88	R	LLGNMIVIVLGHHLGK	D
3589	2	34	B	4.61	4.01	0.218	0.218	gi|122513|sp|P02088.2|HBB1_MOUSE	88	R	LLGNMIVIVLGHHLGK	D
3592	2	34	B	4.61	3.59	0.208	0.208	gi|122513|sp|P02088.2|HBB1_MOUSE	88	R	LLGNMIVIVLGHHLGK	D
3717	2	34	B	4.4	3.58	0.207	0.207	gi|122513|sp|P02088.2|HBB1_MOUSE	88	R	LLGNMIVIVLGHHLGK	D
3720	2	34	B	4.71	3.99	0.159	0.159	gi|122513|sp|P02088.2|HBB1_MOUSE	88	R	LLGNMIVIVLGHHLGK	D
4091	2	34	B	2.74	3.57	0.212	0.212	gi|122513|sp|P02088.2|HBB1_MOUSE	88	R	LLGNMIVIVLGHHLGK	D
4095	2	34	B	3.58	3.4	0.197	0.197	gi|122513|sp|P02088.2|HBB1_MOUSE	88	R	LLGNMIVIVLGHHLGK	D
4203	2	34	B	0.04	3.85	0.235	0.235	gi|122513|sp|P02088.2|HBB1_MOUSE	88	R	LLGNMIVIVLGHHLGK	D
4210	2	34	B	0.06	3.54	0.222	0.222	gi|122513|sp|P02088.2|HBB1_MOUSE	88	R	LLGNMIVIVLGHHLGK	D
4322	2	34	B	3.55	4.25	0.166	0.166	gi|122513|sp|P02088.2|HBB1_MOUSE	88	R	LLGNMIVIVLGHHLGK	D
4324	2	34	B	3.42	3.77	0.191	0.191	gi|122513|sp|P02088.2|HBB1_MOUSE	88	R	LLGNMIVIVLGHHLGK	D
2625	2	34	A	2.6	3.44	0.206	0.206	gi|122513|sp|P02088.2|HBB1_MOUSE	88	R	LLGNMIVIVLGHHLGK	D
4156	2	34	A	0.23	3.04	0.236	0.236	gi|122513|sp|P02088.2|HBB1_MOUSE	88	R	LLGNMIVIVLGHHLGK	D
4169	2	34	A	0.05	3	0.234	0.234	gi|122513|sp|P02088.2|HBB1_MOUSE	88	R	LLGNMIVIVLGHHLGK	D
4275	2	34	A	1.23	2.69	0.187	0.187	gi|122513|sp|P02088.2|HBB1_MOUSE	88	R	LLGNMIVIVLGHHLGK	D
4287	2	34	A	-1.2	2.89	0.278	0.278	gi|122513|sp|P02088.2|HBB1_MOUSE	88	R	LLGNMIVIVLGHHLGK	D
4186	2	34	B	-0.6	2.54	0.488	0.488	gi|27574244|pdb|1O1N|A	99	K	LRVDPVNFK	L
2183	2	34	A	3.21	2.57	0.487	0.487	gi|27574244|pdb|1O1N|A	99	K	LRVDPVNFK	L
2185	2	34	A	3.21	2.5	0.492	0.492	gi|27574244|pdb|1O1N|A	99	K	LRVDPVNFK	L
2314	2	34	B	1.51	2.59	0.127	0.127	gi|344254270|gb|EGW10374.1|	99	K	TYFPHFDVSHGSAQVK	A
4024	2	34	B	4.9	2.91	0.145	0.145	gi|344254270|gb|EGW10374.1|	99	K	TYFPHFDVSHGSAQVK	A
4148	2	34	B	0.3	3.33	0.138	0.138	gi|344254270|gb|EGW10374.1|	99	K	TYFPHFDVSHGSAQVK	A
4252	2	34	B	0.18	2.81	0.16	0.16	gi|344254270|gb|EGW10374.1|	99	K	TYFPHFDVSHGSAQVK	A
4259	2	34	B	0.32	2.9	0.16	0.16	gi|344254270|gb|EGW10374.1|	99	K	TYFPHFDVSHGSAQVK	A
4378	2	34	B	1.32	3.13	0.131	0.131	gi|344254270|gb|EGW10374.1|	99	K	TYFPHFDVSHGSAQVK	A
4385	2	34	B	0.83	3.11	0.138	0.138	gi|344254270|gb|EGW10374.1|	99	K	TYFPHFDVSHGSAQVK	A
4501	2	34	B	0.25	2.83	0.137	0.137	gi|344254270|gb|EGW10374.1|	99	K	TYFPHFDVSHGSAQVK	A
2231	2	34	A	3.13	3.09	0.156	0.156	gi|344254270|gb|EGW10374.1|	99	K	TYFPHFDVSHGSAQVK	A
4172	2	34	A	0.37	2.7	0.124	0.124	gi|344254270|gb|EGW10374.1|	99	K	TYFPHFDVSHGSAQVK	A
4285	2	34	A	0.41	2.66	0.125	0.125	gi|344254270|gb|EGW10374.1|	99	K	TYFPHFDVSHGSAQVK	A
4290	2	34	A	0.42	2.82	0.144	0.144	gi|344254270|gb|EGW10374.1|	99	K	TYFPHFDVSHGSAQVK	A
4393	3	34	A	0.01	3.12	0.14	0.14	gi|344254270|gb|EGW10374.1|	99	K	TYFPHFDVSHGSAQVK	A
4398	3	34	A	-0.2	3.04	0.15	0.15	gi|344254270|gb|EGW10374.1|	99	K	TYFPHFDVSHGSAQVK	A

Scan	z	Sample ID	Band	PPM	XCorr	Delta correlation	Unique delta correlation	Reference	Redundancy		Peptides	
4444	2	34	A	0.26	2.66	0.132	0.132	gi|344254270|gb|EGW10374.1|	99	K	TYFPHFDVSHGSAQVK	A
4507	3	34	A	0.4	3.03	0.15	0.15	gi|344254270|gb|EGW10374.1|	99	K	TYFPHFDVSHGSAQVK	A
4513	3	34	A	0.42	3.06	0.146	0.146	gi|344254270|gb|EGW10374.1|	99	K	TYFPHFDVSHGSAQVK	A
2675	3	34	B	3.32	8.73	0.927	0.927	gi|159137400|gb|ABW88847.1|	2	K	VADALATAAGHLDDLPGALSALSDLHAHK	L
2871	3	34	B	4.52	7.71	0.958	0.958	gi|159137400|gb|ABW88847.1|	2	K	VADALATAAGHLDDLPGALSALSDLHAHK	L
2875	3	34	B	4.4	7.31	0.923	0.923	gi|159137400|gb|ABW88847.1|	2	K	VADALATAAGHLDDLPGALSALSDLHAHK	L
2992	3	34	B	3.62	4.76	0.937	0.937	gi|159137400|gb|ABW88847.1|	2	K	VADALATAAGHLDDLPGALSALSDLHAHK	L
3030	3	34	B	2.79	5.04	0.944	0.944	gi|159137400|gb|ABW88847.1|	2	K	VADALATAAGHLDDLPGALSALSDLHAHK	L
3367	3	34	B	4.11	6.73	0.943	0.943	gi|159137400|gb|ABW88847.1|	2	K	VADALATAAGHLDDLPGALSALSDLHAHK	L
3369	3	34	B	3.99	5.91	0.947	0.947	gi|159137400|gb|ABW88847.1|	2	K	VADALATAAGHLDDLPGALSALSDLHAHK	L
3499	3	34	B	3.59	5.07	0.957	0.957	gi|159137400|gb|ABW88847.1|	2	K	VADALATAAGHLDDLPGALSALSDLHAHK	L
3502	3	34	B	2.27	5.21	0.933	0.933	gi|159137400|gb|ABW88847.1|	2	K	VADALATAAGHLDDLPGALSALSDLHAHK	L
3628	3	34	B	4.71	6.63	0.948	0.948	gi|159137400|gb|ABW88847.1|	2	K	VADALATAAGHLDDLPGALSALSDLHAHK	L
3633	3	34	B	4.14	6.21	0.94	0.94	gi|159137400|gb|ABW88847.1|	2	K	VADALATAAGHLDDLPGALSALSDLHAHK	L
4022	3	34	B	4.62	7.21	0.972	0.972	gi|159137400|gb|ABW88847.1|	2	K	VADALATAAGHLDDLPGALSALSDLHAHK	L
4030	3	34	B	4.48	5.38	0.931	0.931	gi|159137400|gb|ABW88847.1|	2	K	VADALATAAGHLDDLPGALSALSDLHAHK	L
4149	3	34	B	0	7.24	0.937	0.937	gi|159137400|gb|ABW88847.1|	2	K	VADALATAAGHLDDLPGALSALSDLHAHK	L
4155	3	34	B	-0.3	6.87	0.933	0.933	gi|159137400|gb|ABW88847.1|	2	K	VADALATAAGHLDDLPGALSALSDLHAHK	L
4266	3	34	B	0.16	7.67	0.962	0.962	gi|159137400|gb|ABW88847.1|	2	K	VADALATAAGHLDDLPGALSALSDLHAHK	L
4270	3	34	B	0.16	6.86	0.921	0.921	gi|159137400|gb|ABW88847.1|	2	K	VADALATAAGHLDDLPGALSALSDLHAHK	L
4389	3	34	B	0.68	7.54	0.955	0.955	gi|159137400|gb|ABW88847.1|	2	K	VADALATAAGHLDDLPGALSALSDLHAHK	L
4395	3	34	B	0	7.54	0.952	0.952	gi|159137400|gb|ABW88847.1|	2	K	VADALATAAGHLDDLPGALSALSDLHAHK	L
4043	3	34	A	2.83	5.83	0.957	0.957	gi|159137400|gb|ABW88847.1|	2	K	VADALATAAGHLDDLPGALSALSDLHAHK	L
4070	3	34	A	3.26	5.8	0.94	0.94	gi|159137400|gb|ABW88847.1|	2	K	VADALATAAGHLDDLPGALSALSDLHAHK	L
4186	3	34	A	-0	6.3	0.964	0.964	gi|159137400|gb|ABW88847.1|	2	K	VADALATAAGHLDDLPGALSALSDLHAHK	L
4192	3	34	A	-0.3	5.11	0.939	0.939	gi|159137400|gb|ABW88847.1|	2	K	VADALATAAGHLDDLPGALSALSDLHAHK	L
4301	3	34	A	0.87	7.18	0.934	0.934	gi|159137400|gb|ABW88847.1|	2	K	VADALATAAGHLDDLPGALSALSDLHAHK	L
4305	3	34	A	0.65	7.75	0.941	0.941	gi|159137400|gb|ABW88847.1|	2	K	VADALATAAGHLDDLPGALSALSDLHAHK	L
4426	3	34	A	0.31	6.64	0.952	0.952	gi|159137400|gb|ABW88847.1|	2	K	VADALATAAGHLDDLPGALSALSDLHAHK	L
4430	3	34	A	0.48	5.62	0.937	0.937	gi|159137400|gb|ABW88847.1|	2	K	VADALATAAGHLDDLPGALSALSDLHAHK	L
2522	2	34	B	0.95	5.67	0.579	0.579	gi|122513|sp|P02088.2|HBB1_MOUSE	65	K	VITAFNDGLNHLDSLK	G
3544	2	34	B	3.15	3.24	0.565	0.565	gi|122513|sp|P02088.2|HBB1_MOUSE	65	K	VITAFNDGLNHLDSLK	G
3553	2	34	B	3.56	3.89	0.63	0.63	gi|122513|sp|P02088.2|HBB1_MOUSE	65	K	VITAFNDGLNHLDSLK	G
3677	2	34	B	3.67	4.11	0.564	0.564	gi|122513|sp|P02088.2|HBB1_MOUSE	65	K	VITAFNDGLNHLDSLK	G
3686	2	34	B	3.93	4.52	0.595	0.595	gi|122513|sp|P02088.2|HBB1_MOUSE	65	K	VITAFNDGLNHLDSLK	G
4070	2	34	B	3.3	4.32	0.576	0.576	gi|122513|sp|P02088.2|HBB1_MOUSE	65	K	VITAFNDGLNHLDSLK	G
4077	2	34	B	3.68	4.38	0.643	0.643	gi|122513|sp|P02088.2|HBB1_MOUSE	65	K	VITAFNDGLNHLDSLK	G
4184	2	34	B	-0.1	3.75	0.589	0.589	gi|122513|sp|P02088.2|HBB1_MOUSE	65	K	VITAFNDGLNHLDSLK	G

Scan	z	Sample ID	Band	PPM	XCorr	Delta correlation	Unique delta correlation	Reference	Redundancy		Peptides	
4190	2	34	B	-0.2	4.68	0.548	0.548	gi|122513|sp|P02088.2|HBB1_MOUSE	65	K	VITAFNDGLNHLDSLK	G
4319	2	34	B	2.83	3.15	0.689	0.689	gi|122513|sp|P02088.2|HBB1_MOUSE	65	K	VITAFNDGLNHLDSLK	G
4330	2	34	B	2.7	4.52	0.617	0.617	gi|122513|sp|P02088.2|HBB1_MOUSE	65	K	VITAFNDGLNHLDSLK	G
4444	2	34	B	-0.4	3.33	0.642	0.642	gi|122513|sp|P02088.2|HBB1_MOUSE	65	K	VITAFNDGLNHLDSLK	G
4452	2	34	B	-0.4	3.37	0.629	0.629	gi|122513|sp|P02088.2|HBB1_MOUSE	65	K	VITAFNDGLNHLDSLK	G
3838	2	34	A	4.22	3.58	0.604	0.604	gi|122513|sp|P02088.2|HBB1_MOUSE	65	K	VITAFNDGLNHLDSLK	G
4017	2	34	A	3.89	3.91	0.582	0.582	gi|122513|sp|P02088.2|HBB1_MOUSE	65	K	VITAFNDGLNHLDSLK	G
4045	2	34	A	4.17	4.01	0.603	0.603	gi|122513|sp|P02088.2|HBB1_MOUSE	65	K	VITAFNDGLNHLDSLK	G
4164	2	34	A	0.05	4.86	0.565	0.565	gi|122513|sp|P02088.2|HBB1_MOUSE	65	K	VITAFNDGLNHLDSLK	G
4173	2	34	A	-0	3.96	0.623	0.623	gi|122513|sp|P02088.2|HBB1_MOUSE	65	K	VITAFNDGLNHLDSLK	G
4284	2	34	A	0.19	3.96	0.62	0.62	gi|122513|sp|P02088.2|HBB1_MOUSE	65	K	VITAFNDGLNHLDSLK	G
4291	2	34	A	0.34	3.82	0.685	0.685	gi|122513|sp|P02088.2|HBB1_MOUSE	65	K	VITAFNDGLNHLDSLK	G
4407	2	34	A	-0.1	3.54	0.566	0.566	gi|122513|sp|P02088.2|HBB1_MOUSE	65	K	VITAFNDGLNHLDSLK	G
4420	2	34	A	0.07	3.12	0.542	0.542	gi|122513|sp|P02088.2|HBB1_MOUSE	65	K	VITAFNDGLNHLDSLK	G
4531	2	34	A	0.32	2.84	0.724	0.724	gi|122513|sp|P02088.2|HBB1_MOUSE	65	K	VITAFNDGLNHLDSLK	G
4028	2	34	B	4.19	2.58	0.591	0.591	gi|241913510|pdb|3HRW|C	2	-	VLSGEDKSNIK	A
4191	2	34	B	-0.4	2.69	0.514	0.514	gi|241913510|pdb|3HRW|C	2	-	VLSGEDKSNIK	A
4431	2	34	B	0.08	2.83	0.452	0.452	gi|241913510|pdb|3HRW|C	2	-	VLSGEDKSNIK	A
4560	2	34	B	-0.3	2.75	0.475	0.475	gi|241913510|pdb|3HRW|C	2	-	VLSGEDKSNIK	A
4680	2	34	B	-0.9	2.6	0.491	0.491	gi|241913510|pdb|3HRW|C	2	-	VLSGEDKSNIK	A
4682	2	34	B	-0.9	2.52	0.549	0.549	gi|241913510|pdb|3HRW|C	2	-	VLSGEDKSNIK	A
678	2	34	A	-0.6	2.79	0.478	0.478	gi|241913510|pdb|3HRW|C	2	-	VLSGEDKSNIK	A
1914	2	34	A	1.51	3.46	0.522	0.522	gi|241913510|pdb|3HRW|C	2	-	VLSGEDKSNIK	A
1917	2	34	A	1.51	3.11	0.512	0.512	gi|241913510|pdb|3HRW|C	2	-	VLSGEDKSNIK	A
2040	2	34	A	2.5	3.39	0.52	0.52	gi|241913510|pdb|3HRW|C	2	-	VLSGEDKSNIK	A
2044	2	34	A	2.41	3.29	0.546	0.546	gi|241913510|pdb|3HRW|C	2	-	VLSGEDKSNIK	A
4538	2	34	A	0.14	2.63	0.484	0.484	gi|241913510|pdb|3HRW|C	2	-	VLSGEDKSNIK	A
2317	2	34	B	-0.7	2.53	0	0.184	gi|378548370|sp|B3EWD8.1|HBB_TAMHU	0	K	NVADEVGGEALGR^	L
3135	2	34	B	4.22	3.24	0	0.231	gi|378548370|sp|B3EWD8.1|HBB_TAMHU	0	K	NVADEVGGEALGR^	L
3518	2	34	B	3.37	4.17	0	0.301	gi|378548370|sp|B3EWD8.1|HBB_TAMHU	0	K	NVADEVGGEALGR^	L
3783	2	34	B	4.49	3.64	0	0.333	gi|378548370|sp|B3EWD8.1|HBB_TAMHU	0	K	NVADEVGGEALGR^	L
4031	2	34	B	3.87	4.34	0	0.293	gi|378548370|sp|B3EWD8.1|HBB_TAMHU	0	K	NVADEVGGEALGR^	L
4390	2	34	B	-0.5	4.28	0	0.288	gi|378548370|sp|B3EWD8.1|HBB_TAMHU	0	K	NVADEVGGEALGR^	L
4630	2	34	B	-0.7	3.69	0	0.232	gi|378548370|sp|B3EWD8.1|HBB_TAMHU	0	K	NVADEVGGEALGR^	L
4633	2	34	B	-0.7	3.66	0	0.226	gi|378548370|sp|B3EWD8.1|HBB_TAMHU	0	K	NVADEVGGEALGR^	L
2155	2	34	A	3.12	2.89	0	0.264	gi|378548370|sp|B3EWD8.1|HBB_TAMHU	0	K	NVADEVGGEALGR^	L
3719	2	34	A	3.48	3.79	0	0.246	gi|378548370|sp|B3EWD8.1|HBB_TAMHU	0	K	NVADEVGGEALGR^	L
3992	2	34	A	4.06	4.23	0	0.291	gi|378548370|sp|B3EWD8.1|HBB_TAMHU	0	K	NVADEVGGEALGR^	L

Scan	z	Sample ID	Band	PPM	XCorr	Delta correlation	Unique delta correlation	Reference	Redundancy		Peptides	
4113	2	34	A	2.15	4.29	0	0.288	gi|378548370|sp|B3EWD8.1|HBB_TAMHU	0	K	NVADEVGGEALGR^	L
4346	2	34	A	2.7	4.04	0	0.275	gi|378548370|sp|B3EWD8.1|HBB_TAMHU	0	K	NVADEVGGEALGR^	L
2315	2	34	B	-0.2	3.16	0.893	0.893	gi|12847007|dbj|BAB27399.1|	60	K	VVAGVAAALAHK	Y
2448	2	34	B	2.47	3.5	0.894	0.894	gi|12847007|dbj|BAB27399.1|	60	K	VVAGVAAALAHK	Y
2475	2	34	B	2.43	3.57	0.898	0.898	gi|12847007|dbj|BAB27399.1|	60	K	VVAGVAAALAHK	Y
2613	2	34	B	1.75	3.41	0.956	0.956	gi|12847007|dbj|BAB27399.1|	60	K	VVAGVAAALAHK	Y
2619	2	34	B	1.9	3.84	0.913	0.913	gi|12847007|dbj|BAB27399.1|	60	K	VVAGVAAALAHK	Y
3375	2	34	B	4.15	3.29	0.943	0.943	gi|12847007|dbj|BAB27399.1|	60	K	VVAGVAAALAHK	Y
3513	2	34	B	4.27	3.32	0.894	0.894	gi|12847007|dbj|BAB27399.1|	60	K	VVAGVAAALAHK	Y
3515	2	34	B	4.4	3.1	0.889	0.889	gi|12847007|dbj|BAB27399.1|	60	K	VVAGVAAALAHK	Y
3640	2	34	B	3.71	3.53	0.901	0.901	gi|12847007|dbj|BAB27399.1|	60	K	VVAGVAAALAHK	Y
3643	2	34	B	3.34	3.5	0.89	0.89	gi|12847007|dbj|BAB27399.1|	60	K	VVAGVAAALAHK	Y
3767	2	34	B	4.85	2.76	0.849	0.849	gi|12847007|dbj|BAB27399.1|	60	K	VVAGVAAALAHK	Y
3771	2	34	B	4.85	2.9	0.875	0.875	gi|12847007|dbj|BAB27399.1|	60	K	VVAGVAAALAHK	Y
3895	2	34	B	4.8	2.82	0.876	0.876	gi|12847007|dbj|BAB27399.1|	60	K	VVAGVAAALAHK	Y
3899	2	34	B	4.77	3.51	0.902	0.902	gi|12847007|dbj|BAB27399.1|	60	K	VVAGVAAALAHK	Y
4023	2	34	B	4.08	2.89	0.865	0.865	gi|12847007|dbj|BAB27399.1|	60	K	VVAGVAAALAHK	Y
4027	2	34	B	4.07	3.13	0.878	0.878	gi|12847007|dbj|BAB27399.1|	60	K	VVAGVAAALAHK	Y
4145	2	34	B	-0.2	3.71	0.855	0.855	gi|12847007|dbj|BAB27399.1|	60	K	VVAGVAAALAHK	Y
4154	2	34	B	-0.2	4	0.916	0.916	gi|12847007|dbj|BAB27399.1|	60	K	VVAGVAAALAHK	Y
4264	2	34	B	-0.1	3.55	0.911	0.911	gi|12847007|dbj|BAB27399.1|	60	K	VVAGVAAALAHK	Y
4272	2	34	B	-0.1	3.82	0.885	0.885	gi|12847007|dbj|BAB27399.1|	60	K	VVAGVAAALAHK	Y
4388	2	34	B	0.31	4.12	0.837	0.837	gi|12847007|dbj|BAB27399.1|	60	K	VVAGVAAALAHK	Y
4394	2	34	B	-0.3	3.48	0.901	0.901	gi|12847007|dbj|BAB27399.1|	60	K	VVAGVAAALAHK	Y
4507	2	34	B	-0.1	3.86	0.902	0.902	gi|12847007|dbj|BAB27399.1|	60	K	VVAGVAAALAHK	Y
4512	2	34	B	-0.1	3.72	0.87	0.87	gi|12847007|dbj|BAB27399.1|	60	K	VVAGVAAALAHK	Y
4631	2	34	B	-0.9	3.36	0.87	0.87	gi|12847007|dbj|BAB27399.1|	60	K	VVAGVAAALAHK	Y
4634	2	34	B	-0.9	3.26	0.897	0.897	gi|12847007|dbj|BAB27399.1|	60	K	VVAGVAAALAHK	Y
2145	2	34	A	2.67	3.1	0.871	0.871	gi|12847007|dbj|BAB27399.1|	60	K	VVAGVAAALAHK	Y
4108	2	34	A	3.1	2.91	0.936	0.936	gi|12847007|dbj|BAB27399.1|	60	K	VVAGVAAALAHK	Y
4111	2	34	A	3.44	3.01	0.946	0.946	gi|12847007|dbj|BAB27399.1|	60	K	VVAGVAAALAHK	Y
4228	2	34	A	-0.2	3.47	0.944	0.944	gi|12847007|dbj|BAB27399.1|	60	K	VVAGVAAALAHK	Y
4233	2	34	A	-0.3	3.7	0.931	0.931	gi|12847007|dbj|BAB27399.1|	60	K	VVAGVAAALAHK	Y
4353	2	34	A	3.78	3.67	0.884	0.884	gi|12847007|dbj|BAB27399.1|	60	K	VVAGVAAALAHK	Y
4361	2	34	A	3.67	3.34	0.948	0.948	gi|12847007|dbj|BAB27399.1|	60	K	VVAGVAAALAHK	Y
4478	2	34	A	-0.2	3.48	0.904	0.904	gi|12847007|dbj|BAB27399.1|	60	K	VVAGVAAALAHK	Y
4489	2	34	A	-0.1	3.83	0.862	0.862	gi|12847007|dbj|BAB27399.1|	60	K	VVAGVAAALAHK	Y
4604	2	34	A	-0.5	3.75	0.854	0.854	gi|12847007|dbj|BAB27399.1|	60	K	VVAGVAAALAHK	Y

Scan	z	Sample ID	Band	PPM	XCorr	Delta correlation	Unique delta correlation	Reference	Redundancy		Peptides	
4607	2	34	A	-0.5	3.7	0.925	0.925	gi|12847007|dbj|BAB27399.1|	60	K	VVAGVAAALAHK	Y
2485	2	34	B	0.47	2.8	0.955	0.955	gi|12847007|dbj|BAB27399.1|	60	K	VVAGVAAALAHKYH	-
3346	2	34	B	3.7	3.45	0.954	0.954	gi|12847007|dbj|BAB27399.1|	60	K	VVAGVAAALAHKYH	-
3365	2	34	B	2.87	3.53	0.946	0.946	gi|12847007|dbj|BAB27399.1|	60	K	VVAGVAAALAHKYH	-
3493	2	34	B	2.85	3.93	0.952	0.952	gi|12847007|dbj|BAB27399.1|	60	K	VVAGVAAALAHKYH	-
3495	2	34	B	2.69	3.91	0.953	0.953	gi|12847007|dbj|BAB27399.1|	60	K	VVAGVAAALAHKYH	-
3621	2	34	B	4.22	3.7	0.901	0.901	gi|12847007|dbj|BAB27399.1|	60	K	VVAGVAAALAHKYH	-
3626	2	34	B	4.29	3.99	0.949	0.949	gi|12847007|dbj|BAB27399.1|	60	K	VVAGVAAALAHKYH	-
4004	2	34	B	4.69	3.96	0.951	0.951	gi|12847007|dbj|BAB27399.1|	60	K	VVAGVAAALAHKYH	-
4006	2	34	B	4.54	4.07	0.946	0.946	gi|12847007|dbj|BAB27399.1|	60	K	VVAGVAAALAHKYH	-
4124	2	34	B	0.21	3.6	0.949	0.949	gi|12847007|dbj|BAB27399.1|	60	K	VVAGVAAALAHKYH	-
4130	2	34	B	0.23	3.68	0.95	0.95	gi|12847007|dbj|BAB27399.1|	60	K	VVAGVAAALAHKYH	-
4237	2	34	B	-0.4	3.69	0.945	0.945	gi|12847007|dbj|BAB27399.1|	60	K	VVAGVAAALAHKYH	-
4243	2	34	B	-0.4	3.67	0.903	0.903	gi|12847007|dbj|BAB27399.1|	60	K	VVAGVAAALAHKYH	-
4359	2	34	B	2.32	4.27	0.953	0.953	gi|12847007|dbj|BAB27399.1|	60	K	VVAGVAAALAHKYH	-
4363	2	34	B	2.42	4.17	0.946	0.946	gi|12847007|dbj|BAB27399.1|	60	K	VVAGVAAALAHKYH	-
4477	2	34	B	-0	4.16	0.951	0.951	gi|12847007|dbj|BAB27399.1|	60	K	VVAGVAAALAHKYH	-
4482	2	34	B	0.01	4.04	0.949	0.949	gi|12847007|dbj|BAB27399.1|	60	K	VVAGVAAALAHKYH	-
4610	2	34	B	-0.9	2.68	0.947	0.947	gi|12847007|dbj|BAB27399.1|	60	K	VVAGVAAALAHKYH	-
4157	2	34	A	0.13	3.35	0.938	0.938	gi|12847007|dbj|BAB27399.1|	60	K	VVAGVAAALAHKYH	-
4181	2	34	A	-0	2.83	0.947	0.947	gi|12847007|dbj|BAB27399.1|	60	K	VVAGVAAALAHKYH	-
4295	2	34	A	0.3	3.22	0.948	0.948	gi|12847007|dbj|BAB27399.1|	60	K	VVAGVAAALAHKYH	-
4416	2	34	A	0.14	3.12	0.959	0.959	gi|12847007|dbj|BAB27399.1|	60	K	VVAGVAAALAHKYH	-
4433	2	34	A	0.11	3.33	0.893	0.893	gi|12847007|dbj|BAB27399.1|	60	K	VVAGVAAALAHKYH	-
4539	2	34	A	-0.2	3.5	0.943	0.943	gi|12847007|dbj|BAB27399.1|	60	K	VVAGVAAALAHKYH	-
4541	2	34	A	-0.2	3.51	0.96	0.96	gi|12847007|dbj|BAB27399.1|	60	K	VVAGVAAALAHKYH	-
2580	3	34	B	1.85	3.58	0.391	0.391	gi|187369324|dbj|BAG31393.1|	73	R	YFDSFGDLSSASAIM*GNAK	V
2588	2	34	B	3.32	5.81	0.52	0.52	gi|187369324|dbj|BAG31393.1|	73	R	YFDSFGDLSSASAIM*GNAK	V
2840	2	34	B	2.76	4.92	0.619	0.619	gi|187369324|dbj|BAG31393.1|	73	R	YFDSFGDLSSASAIM*GNAK	V
2842	2	34	B	2.67	4.46	0.597	0.597	gi|187369324|dbj|BAG31393.1|	73	R	YFDSFGDLSSASAIM*GNAK	V
2993	2	34	B	3.97	4.29	0.574	0.574	gi|187369324|dbj|BAG31393.1|	73	R	YFDSFGDLSSASAIM*GNAK	V
2998	2	34	B	4.19	2.81	0.66	0.66	gi|187369324|dbj|BAG31393.1|	73	R	YFDSFGDLSSASAIM*GNAK	V
3126	2	34	B	2.92	3.15	0.638	0.638	gi|187369324|dbj|BAG31393.1|	73	R	YFDSFGDLSSASAIM*GNAK	V
3484	2	34	B	4.15	2.51	0.646	0.646	gi|187369324|dbj|BAG31393.1|	73	R	YFDSFGDLSSASAIM*GNAK	V
3618	2	34	B	4.87	3.92	0.614	0.614	gi|187369324|dbj|BAG31393.1|	73	R	YFDSFGDLSSASAIM*GNAK	V
3744	2	34	B	4.97	4.97	0.561	0.561	gi|187369324|dbj|BAG31393.1|	73	R	YFDSFGDLSSASAIM*GNAK	V
4113	2	34	B	0.44	5.93	0.506	0.506	gi|187369324|dbj|BAG31393.1|	73	R	YFDSFGDLSSASAIM*GNAK	V
4123	2	34	B	0.33	6.08	0.559	0.559	gi|187369324|dbj|BAG31393.1|	73	R	YFDSFGDLSSASAIM*GNAK	V

Scan	z	Sample ID	Band	PPM	XCorr	Delta correlation	Unique delta correlation	Reference	Redundancy		Peptides	
4219	3	34	B	-0.5	3.19	0.258	0.258	gi|187369324|dbj|BAG31393.1|	73	R	YFDSFGDLSSASAIM*GNAK	V
4232	2	34	B	-0.2	5.26	0.62	0.62	gi|187369324|dbj|BAG31393.1|	73	R	YFDSFGDLSSASAIM*GNAK	V
4239	2	34	B	-0.2	5.1	0.553	0.553	gi|187369324|dbj|BAG31393.1|	73	R	YFDSFGDLSSASAIM*GNAK	V
4352	2	34	B	3.14	5.39	0.543	0.543	gi|187369324|dbj|BAG31393.1|	73	R	YFDSFGDLSSASAIM*GNAK	V
4356	2	34	B	3.25	4.8	0.536	0.536	gi|187369324|dbj|BAG31393.1|	73	R	YFDSFGDLSSASAIM*GNAK	V
4435	3	34	B	-1.3	3.08	0.455	0.455	gi|187369324|dbj|BAG31393.1|	73	R	YFDSFGDLSSASAIM*GNAK	V
4445	3	34	B	-0.9	3.53	0.448	0.448	gi|187369324|dbj|BAG31393.1|	73	R	YFDSFGDLSSASAIM*GNAK	V
4471	2	34	B	0.14	4.02	0.603	0.603	gi|187369324|dbj|BAG31393.1|	73	R	YFDSFGDLSSASAIM*GNAK	V
4473	2	34	B	0.16	5.76	0.558	0.558	gi|187369324|dbj|BAG31393.1|	73	R	YFDSFGDLSSASAIM*GNAK	V
2503	2	34	A	3.52	2.85	0.712	0.712	gi|187369324|dbj|BAG31393.1|	73	R	YFDSFGDLSSASAIM*GNAK	V
2505	2	34	A	3.53	3.12	0.726	0.726	gi|187369324|dbj|BAG31393.1|	73	R	YFDSFGDLSSASAIM*GNAK	V
2861	2	34	A	3.89	3.45	0.712	0.712	gi|187369324|dbj|BAG31393.1|	73	R	YFDSFGDLSSASAIM*GNAK	V
2871	2	34	A	3.78	2.95	0.697	0.697	gi|187369324|dbj|BAG31393.1|	73	R	YFDSFGDLSSASAIM*GNAK	V
2984	2	34	A	4.52	3.52	0.562	0.562	gi|187369324|dbj|BAG31393.1|	73	R	YFDSFGDLSSASAIM*GNAK	V
2992	2	34	A	4.51	3.25	0.698	0.698	gi|187369324|dbj|BAG31393.1|	73	R	YFDSFGDLSSASAIM*GNAK	V
3560	2	34	A	4.12	3.36	0.63	0.63	gi|187369324|dbj|BAG31393.1|	73	R	YFDSFGDLSSASAIM*GNAK	V
3836	2	34	A	3.88	4.49	0.614	0.614	gi|187369324|dbj|BAG31393.1|	73	R	YFDSFGDLSSASAIM*GNAK	V
3846	2	34	A	3.99	4.14	0.624	0.624	gi|187369324|dbj|BAG31393.1|	73	R	YFDSFGDLSSASAIM*GNAK	V
3986	2	34	A	4.12	3.43	0.536	0.536	gi|187369324|dbj|BAG31393.1|	73	R	YFDSFGDLSSASAIM*GNAK	V
3989	2	34	A	4.7	4.45	0.583	0.583	gi|187369324|dbj|BAG31393.1|	73	R	YFDSFGDLSSASAIM*GNAK	V
4085	3	34	A	3.65	4.5	0.465	0.465	gi|187369324|dbj|BAG31393.1|	73	R	YFDSFGDLSSASAIM*GNAK	V
4096	3	34	A	2	4.28	0.341	0.341	gi|187369324|dbj|BAG31393.1|	73	R	YFDSFGDLSSASAIM*GNAK	V
4110	2	34	A	2.01	3.67	0.658	0.658	gi|187369324|dbj|BAG31393.1|	73	R	YFDSFGDLSSASAIM*GNAK	V
4114	2	34	A	2.62	4.96	0.534	0.534	gi|187369324|dbj|BAG31393.1|	73	R	YFDSFGDLSSASAIM*GNAK	V
4207	3	34	A	-0.8	3.25	0.413	0.413	gi|187369324|dbj|BAG31393.1|	73	R	YFDSFGDLSSASAIM*GNAK	V
4216	3	34	A	-0.8	3.4	0.406	0.406	gi|187369324|dbj|BAG31393.1|	73	R	YFDSFGDLSSASAIM*GNAK	V
4222	2	34	A	-0	4.32	0.532	0.532	gi|187369324|dbj|BAG31393.1|	73	R	YFDSFGDLSSASAIM*GNAK	V
4226	2	34	A	0.14	5.38	0.6	0.6	gi|187369324|dbj|BAG31393.1|	73	R	YFDSFGDLSSASAIM*GNAK	V
4338	2	34	A	3.89	5.99	0.551	0.551	gi|187369324|dbj|BAG31393.1|	73	R	YFDSFGDLSSASAIM*GNAK	V
4342	3	34	A	3.34	4.84	0.406	0.406	gi|187369324|dbj|BAG31393.1|	73	R	YFDSFGDLSSASAIM*GNAK	V
4345	2	34	A	3.34	5.78	0.524	0.524	gi|187369324|dbj|BAG31393.1|	73	R	YFDSFGDLSSASAIM*GNAK	V
4455	3	34	A	-1	4.02	0.421	0.421	gi|187369324|dbj|BAG31393.1|	73	R	YFDSFGDLSSASAIM*GNAK	V
4460	2	34	A	0.26	6.02	0.505	0.505	gi|187369324|dbj|BAG31393.1|	73	R	YFDSFGDLSSASAIM*GNAK	V
4463	3	34	A	-1.1	3.35	0.44	0.44	gi|187369324|dbj|BAG31393.1|	73	R	YFDSFGDLSSASAIM*GNAK	V
4469	2	34	A	0.35	5.05	0.615	0.615	gi|187369324|dbj|BAG31393.1|	73	R	YFDSFGDLSSASAIM*GNAK	V
2800	2	34	B	3.7	6.35	0.576	0.576	gi|187369324|dbj|BAG31393.1|	73	R	YFDSFGDLSSASAIMGNAK	V
2802	2	34	B	3.81	6.17	0.566	0.566	gi|187369324|dbj|BAG31393.1|	73	R	YFDSFGDLSSASAIMGNAK	V
2886	3	34	B	3.65	3.03	0.31	0.31	gi|187369324|dbj|BAG31393.1|	73	R	YFDSFGDLSSASAIMGNAK	V

Scan	z	Sample ID	Band	PPM	XCorr	Delta correlation	Unique delta correlation	Reference	Redundancy		Peptides	
2918	2	34	B	3.9	5.52	0.549	0.549	gi|187369324|dbj|BAG31393.1|	73	R	YFDSFGDLSSASAIMGNAK	V
2929	2	34	B	3.86	5.7	0.529	0.529	gi|187369324|dbj|BAG31393.1|	73	R	YFDSFGDLSSASAIMGNAK	V
3009	3	34	B	-3	3.32	0.22	0.22	gi|187369324|dbj|BAG31393.1|	73	R	YFDSFGDLSSASAIMGNAK	V
3048	2	34	B	3.96	5.52	0.527	0.527	gi|187369324|dbj|BAG31393.1|	73	R	YFDSFGDLSSASAIMGNAK	V
3060	2	34	B	3.9	6.14	0.526	0.526	gi|187369324|dbj|BAG31393.1|	73	R	YFDSFGDLSSASAIMGNAK	V
3185	2	34	B	4.45	4.96	0.555	0.555	gi|187369324|dbj|BAG31393.1|	73	R	YFDSFGDLSSASAIMGNAK	V
3187	2	34	B	4.74	4.67	0.606	0.606	gi|187369324|dbj|BAG31393.1|	73	R	YFDSFGDLSSASAIMGNAK	V
3320	2	34	B	4.33	3.39	0.59	0.59	gi|187369324|dbj|BAG31393.1|	73	R	YFDSFGDLSSASAIMGNAK	V
3323	2	34	B	4.18	3.92	0.665	0.665	gi|187369324|dbj|BAG31393.1|	73	R	YFDSFGDLSSASAIMGNAK	V
3450	2	34	B	4.26	3.68	0.584	0.584	gi|187369324|dbj|BAG31393.1|	73	R	YFDSFGDLSSASAIMGNAK	V
3453	2	34	B	4.21	4.3	0.642	0.642	gi|187369324|dbj|BAG31393.1|	73	R	YFDSFGDLSSASAIMGNAK	V
3580	2	34	B	4.34	4.27	0.587	0.587	gi|187369324|dbj|BAG31393.1|	73	R	YFDSFGDLSSASAIMGNAK	V
3582	2	34	B	4.41	4.56	0.648	0.648	gi|187369324|dbj|BAG31393.1|	73	R	YFDSFGDLSSASAIMGNAK	V
3706	2	34	B	4.73	5.72	0.571	0.571	gi|187369324|dbj|BAG31393.1|	73	R	YFDSFGDLSSASAIMGNAK	V
3708	2	34	B	4.64	5.17	0.553	0.553	gi|187369324|dbj|BAG31393.1|	73	R	YFDSFGDLSSASAIMGNAK	V
4080	2	34	B	3.23	6.01	0.549	0.549	gi|187369324|dbj|BAG31393.1|	73	R	YFDSFGDLSSASAIMGNAK	V
4084	2	34	B	1.53	6.17	0.536	0.536	gi|187369324|dbj|BAG31393.1|	73	R	YFDSFGDLSSASAIMGNAK	V
4195	2	34	B	0.22	6.02	0.557	0.557	gi|187369324|dbj|BAG31393.1|	73	R	YFDSFGDLSSASAIMGNAK	V
4199	2	34	B	0.12	5.48	0.558	0.558	gi|187369324|dbj|BAG31393.1|	73	R	YFDSFGDLSSASAIMGNAK	V
4207	3	34	B	-1.1	3.4	0.313	0.313	gi|187369324|dbj|BAG31393.1|	73	R	YFDSFGDLSSASAIMGNAK	V
4213	3	34	B	-1.1	3.86	0.342	0.342	gi|187369324|dbj|BAG31393.1|	73	R	YFDSFGDLSSASAIMGNAK	V
4310	2	34	B	3.95	5.15	0.627	0.627	gi|187369324|dbj|BAG31393.1|	73	R	YFDSFGDLSSASAIMGNAK	V
4312	2	34	B	3.86	5.33	0.58	0.58	gi|187369324|dbj|BAG31393.1|	73	R	YFDSFGDLSSASAIMGNAK	V
4326	3	34	B	2.09	3.42	0.274	0.274	gi|187369324|dbj|BAG31393.1|	73	R	YFDSFGDLSSASAIMGNAK	V
4430	2	34	B	0.49	6.26	0.583	0.583	gi|187369324|dbj|BAG31393.1|	73	R	YFDSFGDLSSASAIMGNAK	V
4438	2	34	B	0.45	6.3	0.554	0.554	gi|187369324|dbj|BAG31393.1|	73	R	YFDSFGDLSSASAIMGNAK	V
2626	2	34	A	2.39	2.96	0.716	0.716	gi|187369324|dbj|BAG31393.1|	73	R	YFDSFGDLSSASAIMGNAK	V
2723	2	34	A	4.04	3.31	0.675	0.675	gi|187369324|dbj|BAG31393.1|	73	R	YFDSFGDLSSASAIMGNAK	V
2730	2	34	A	4.17	3.03	0.648	0.648	gi|187369324|dbj|BAG31393.1|	73	R	YFDSFGDLSSASAIMGNAK	V
2845	2	34	A	4.82	4.42	0.588	0.588	gi|187369324|dbj|BAG31393.1|	73	R	YFDSFGDLSSASAIMGNAK	V
2851	2	34	A	4.82	5.36	0.554	0.554	gi|187369324|dbj|BAG31393.1|	73	R	YFDSFGDLSSASAIMGNAK	V
3341	2	34	A	4.97	4.58	0.475	0.475	gi|187369324|dbj|BAG31393.1|	73	R	YFDSFGDLSSASAIMGNAK	V
3343	2	34	A	4.8	5.42	0.574	0.574	gi|187369324|dbj|BAG31393.1|	73	R	YFDSFGDLSSASAIMGNAK	V
3473	2	34	A	4.88	3.48	0.713	0.713	gi|187369324|dbj|BAG31393.1|	73	R	YFDSFGDLSSASAIMGNAK	V
3475	2	34	A	4.88	4.15	0.708	0.708	gi|187369324|dbj|BAG31393.1|	73	R	YFDSFGDLSSASAIMGNAK	V
3554	3	34	A	4.2	3.1	0.294	0.294	gi|187369324|dbj|BAG31393.1|	73	R	YFDSFGDLSSASAIMGNAK	V
3708	3	34	A	4.48	4.51	0.323	0.323	gi|187369324|dbj|BAG31393.1|	73	R	YFDSFGDLSSASAIMGNAK	V
3711	3	34	A	4.23	4.43	0.34	0.34	gi|187369324|dbj|BAG31393.1|	73	R	YFDSFGDLSSASAIMGNAK	V

Scan	z	Sample ID	Band	PPM	XCorr	Delta correlation	Unique delta correlation	Reference	Redundancy		Peptides	
3737	2	34	A	4.5	5.33	0.604	0.604	gi|187369324|dbj|BAG31393.1|	73	R	YFDSFGDLSSASAIMGNAK	V
3739	2	34	A	4.61	5.86	0.555	0.555	gi|187369324|dbj|BAG31393.1|	73	R	YFDSFGDLSSASAIMGNAK	V
3841	3	34	A	4.13	4.76	0.288	0.288	gi|187369324|dbj|BAG31393.1|	73	R	YFDSFGDLSSASAIMGNAK	V
3843	3	34	A	4.14	4.62	0.289	0.289	gi|187369324|dbj|BAG31393.1|	73	R	YFDSFGDLSSASAIMGNAK	V
3868	2	34	A	4.92	4.35	0.631	0.631	gi|187369324|dbj|BAG31393.1|	73	R	YFDSFGDLSSASAIMGNAK	V
3973	3	34	A	4.83	4.09	0.319	0.319	gi|187369324|dbj|BAG31393.1|	73	R	YFDSFGDLSSASAIMGNAK	V
3979	3	34	A	4.47	4.34	0.323	0.323	gi|187369324|dbj|BAG31393.1|	73	R	YFDSFGDLSSASAIMGNAK	V
3998	2	34	A	4.62	5.75	0.523	0.523	gi|187369324|dbj|BAG31393.1|	73	R	YFDSFGDLSSASAIMGNAK	V
4000	2	34	A	4.56	6.35	0.501	0.501	gi|187369324|dbj|BAG31393.1|	73	R	YFDSFGDLSSASAIMGNAK	V
4100	3	34	A	2.76	5.26	0.31	0.31	gi|187369324|dbj|BAG31393.1|	73	R	YFDSFGDLSSASAIMGNAK	V
4102	3	34	A	2.56	3.49	0.234	0.234	gi|187369324|dbj|BAG31393.1|	73	R	YFDSFGDLSSASAIMGNAK	V
4120	2	34	A	2.47	5.99	0.576	0.576	gi|187369324|dbj|BAG31393.1|	73	R	YFDSFGDLSSASAIMGNAK	V
4123	2	34	A	1.81	5.82	0.553	0.553	gi|187369324|dbj|BAG31393.1|	73	R	YFDSFGDLSSASAIMGNAK	V
4213	3	34	A	-0.2	5.35	0.348	0.348	gi|187369324|dbj|BAG31393.1|	73	R	YFDSFGDLSSASAIMGNAK	V
4218	3	34	A	-0.2	4.85	0.365	0.365	gi|187369324|dbj|BAG31393.1|	73	R	YFDSFGDLSSASAIMGNAK	V
4236	2	34	A	0.61	5.91	0.585	0.585	gi|187369324|dbj|BAG31393.1|	73	R	YFDSFGDLSSASAIMGNAK	V
4240	2	34	A	0.65	5.25	0.587	0.587	gi|187369324|dbj|BAG31393.1|	73	R	YFDSFGDLSSASAIMGNAK	V
4334	3	34	A	3.22	4.78	0.303	0.303	gi|187369324|dbj|BAG31393.1|	73	R	YFDSFGDLSSASAIMGNAK	V
4337	3	34	A	3.74	4.37	0.383	0.383	gi|187369324|dbj|BAG31393.1|	73	R	YFDSFGDLSSASAIMGNAK	V
4355	2	34	A	4.24	5.59	0.533	0.533	gi|187369324|dbj|BAG31393.1|	73	R	YFDSFGDLSSASAIMGNAK	V
4359	2	34	A	3.07	3.47	0.701	0.701	gi|187369324|dbj|BAG31393.1|	73	R	YFDSFGDLSSASAIMGNAK	V
4452	3	34	A	-0.1	4.27	0.38	0.38	gi|187369324|dbj|BAG31393.1|	73	R	YFDSFGDLSSASAIMGNAK	V
4459	3	34	A	-0.2	4.32	0.349	0.349	gi|187369324|dbj|BAG31393.1|	73	R	YFDSFGDLSSASAIMGNAK	V
4475	2	34	A	0.92	5.99	0.584	0.584	gi|187369324|dbj|BAG31393.1|	73	R	YFDSFGDLSSASAIMGNAK	V
4486	2	34	A	0.85	6.22	0.586	0.586	gi|187369324|dbj|BAG31393.1|	73	R	YFDSFGDLSSASAIMGNAK	V
2531	2	35	A	0.05	2.63	0.891	0.891	gi|554577472|ref|XP_005880462.1|	74	K	AAVSGLWGK	V
3979	2	35	A	4.69	2.61	0.982	0.982	gi|554577472|ref|XP_005880462.1|	74	K	AAVSGLWGK	V
2375	2	35	B	2.69	3.33	0.897	0.897	gi|554577472|ref|XP_005880462.1|	74	K	AAVSGLWGK	V
2386	2	35	B	2.69	3.2	0.921	0.921	gi|554577472|ref|XP_005880462.1|	74	K	AAVSGLWGK	V
3365	2	35	B	4.94	2.59	0.984	0.984	gi|554577472|ref|XP_005880462.1|	74	K	AAVSGLWGK	V
3512	2	35	B	0.01	2.97	0.894	0.894	gi|554577472|ref|XP_005880462.1|	74	K	AAVSGLWGK	V
3634	2	35	B	-0.5	2.51	0.986	0.986	gi|554577472|ref|XP_005880462.1|	74	K	AAVSGLWGK	V
3762	2	35	B	4.87	2.6	0.878	0.878	gi|554577472|ref|XP_005880462.1|	74	K	AAVSGLWGK	V
3884	2	35	B	-0.6	2.63	0.983	0.983	gi|554577472|ref|XP_005880462.1|	74	K	AAVSGLWGK	V
2528	2	35	A	0.98	4.09	0.969	0.969	gi|532054982|ref|XP_005370208.1|	94	K	DFTPAAQAAFQK	V
2364	2	35	B	3.15	3.06	0.965	0.965	gi|532054982|ref|XP_005370208.1|	94	K	DFTPAAQAAFQK	V
3694	2	35	B	0.39	2.79	0.99	0.99	gi|532054982|ref|XP_005370208.1|	94	K	DFTPAAQAAFQK	V
2706	2	35	A	2.63	3.34	0	0.381	gi|27574244|pdb|1O1N|A	0	K	FLASVSTVLTSK	Y

Scan	z	Sample ID	Band	PPM	XCorr	Delta correlation	Unique delta correlation	Reference	Redundancy		Peptides	
2715	2	35	A	2.65	3.21	0	0.388	gi|27574244|pdb|1O1N|A	0	K	FLASVSTVLTSK	Y
2965	2	35	A	2.16	2.78	0	0.392	gi|27574244|pdb|1O1N|A	0	K	FLASVSTVLTSK	Y
3101	2	35	A	3.26	2.93	0	0.341	gi|27574244|pdb|1O1N|A	0	K	FLASVSTVLTSK	Y
3121	2	35	A	3.22	3.18	0	0.409	gi|27574244|pdb|1O1N|A	0	K	FLASVSTVLTSK	Y
3499	2	35	A	3.94	3	0	0.398	gi|27574244|pdb|1O1N|A	0	K	FLASVSTVLTSK	Y
3501	2	35	A	3.66	2.63	0	0.4	gi|27574244|pdb|1O1N|A	0	K	FLASVSTVLTSK	Y
3637	2	35	A	3.84	2.6	0	0.38	gi|27574244|pdb|1O1N|A	0	K	FLASVSTVLTSK	Y
3640	2	35	A	3.83	2.69	0	0.403	gi|27574244|pdb|1O1N|A	0	K	FLASVSTVLTSK	Y
3773	2	35	A	3.85	3.1	0	0.351	gi|27574244|pdb|1O1N|A	0	K	FLASVSTVLTSK	Y
3776	2	35	A	3.84	2.58	0	0.458	gi|27574244|pdb|1O1N|A	0	K	FLASVSTVLTSK	Y
3907	2	35	A	3.29	3.01	0	0.375	gi|27574244|pdb|1O1N|A	0	K	FLASVSTVLTSK	Y
3923	2	35	A	4.25	2.52	0	0.406	gi|27574244|pdb|1O1N|A	0	K	FLASVSTVLTSK	Y
4193	2	35	A	3.8	2.6	0	0.328	gi|27574244|pdb|1O1N|A	0	K	FLASVSTVLTSK	Y
2550	2	35	B	2.22	3.6	0	0.311	gi|27574244|pdb|1O1N|A	0	K	FLASVSTVLTSK	Y
2561	2	35	B	2.2	3.48	0	0.39	gi|27574244|pdb|1O1N|A	0	K	FLASVSTVLTSK	Y
2677	2	35	B	1.11	3.43	0	0.382	gi|27574244|pdb|1O1N|A	0	K	FLASVSTVLTSK	Y
3045	2	35	B	3.66	2.89	0	0.287	gi|27574244|pdb|1O1N|A	0	K	FLASVSTVLTSK	Y
3501	2	35	B	0.61	2.86	0	0.402	gi|27574244|pdb|1O1N|A	0	K	FLASVSTVLTSK	Y
3504	2	35	B	0.57	2.81	0	0.378	gi|27574244|pdb|1O1N|A	0	K	FLASVSTVLTSK	Y
3622	2	35	B	0.02	2.95	0	0.415	gi|27574244|pdb|1O1N|A	0	K	FLASVSTVLTSK	Y
3736	2	35	B	0.26	3.16	0	0.363	gi|27574244|pdb|1O1N|A	0	K	FLASVSTVLTSK	Y
3740	2	35	B	0.23	3.04	0	0.405	gi|27574244|pdb|1O1N|A	0	K	FLASVSTVLTSK	Y
3857	2	35	B	0.02	2.63	0	0.389	gi|27574244|pdb|1O1N|A	0	K	FLASVSTVLTSK	Y
3860	2	35	B	-0	2.76	0	0.39	gi|27574244|pdb|1O1N|A	0	K	FLASVSTVLTSK	Y
2821	2	35	A	2.08	3.04	0.902	0.902	gi|56967333|pdb|1Y0D|C	4	K	FLASVSTVLTSKY	-
2831	2	35	A	2.06	3	0.901	0.901	gi|56967333|pdb|1Y0D|C	4	K	FLASVSTVLTSKY	-
2698	2	35	B	-0.6	2.72	0.893	0.893	gi|56967333|pdb|1Y0D|C	4	K	FLASVSTVLTSKY	-
2303	2	35	B	0.46	2.65	0.278	0.278	gi|528765669|gb|EPY85328.1|	8	K	IGGHAAEYGAEALER	M
2675	2	35	A	3.21	3.99	0.107	0.107	gi|344254270|gb|EGW10374.1|	99	K	IGGHGAEYGAEALER	M
3653	2	35	B	0.84	2.58	0.11	0.11	gi|344254270|gb|EGW10374.1|	99	K	IGGHGAEYGAEALER	M
2444	3	35	B	1.91	5.17	0.576	0.576	gi|122513|sp|P02088.2|HBB1_MOUSE	65	K	KVITAFNDGLNHLDSLK	G
2841	2	35	A	3.33	2.66	0.159	0.159	gi|122513|sp|P02088.2|HBB1_MOUSE	88	R	LLGNM*IVIVLGHHLGK	D
2653	3	35	B	2.14	3.73	0.105	0.105	gi|122513|sp|P02088.2|HBB1_MOUSE	88	R	LLGNM*IVIVLGHHLGK	D
2655	2	35	B	2.13	3.75	0.165	0.165	gi|122513|sp|P02088.2|HBB1_MOUSE	88	R	LLGNM*IVIVLGHHLGK	D
2666	2	35	B	2.14	4.41	0.177	0.177	gi|122513|sp|P02088.2|HBB1_MOUSE	88	R	LLGNM*IVIVLGHHLGK	D
2911	2	35	A	4.51	3.8	0.125	0.125	gi|122513|sp|P02088.2|HBB1_MOUSE	88	R	LLGNMIVIVLGHHLGK	D
2919	2	35	A	4.51	3.73	0.227	0.227	gi|122513|sp|P02088.2|HBB1_MOUSE	88	R	LLGNMIVIVLGHHLGK	D
3036	2	35	A	3.24	3.3	0.182	0.182	gi|122513|sp|P02088.2|HBB1_MOUSE	88	R	LLGNMIVIVLGHHLGK	D

Scan	z	Sample ID	Band	PPM	XCorr	Delta correlation	Unique delta correlation	Reference	Redundancy		Peptides	
3042	2	35	A	3.24	3.56	0.174	0.174	gi|122513|sp|P02088.2|HBB1_MOUSE	88	R	LLGNMIVIVLGHHLGK	D
2841	2	35	B	2.22	3.75	0.203	0.203	gi|122513|sp|P02088.2|HBB1_MOUSE	88	R	LLGNMIVIVLGHHLGK	D
2853	2	35	B	2.19	3.91	0.218	0.218	gi|122513|sp|P02088.2|HBB1_MOUSE	88	R	LLGNMIVIVLGHHLGK	D
3607	2	35	B	-0	2.71	0.983	0.983	gi|431903465|gb|ELK09417.1|	99	R	LLVVYPWTQR	F
2487	2	35	A	2.83	2.76	0.488	0.488	gi|27574244|pdb|1O1N|A	99	K	LRVDPVNFK	L
2495	2	35	A	2.83	2.73	0.48	0.48	gi|27574244|pdb|1O1N|A	99	K	LRVDPVNFK	L
2331	2	35	B	1.18	2.72	0.464	0.464	gi|27574244|pdb|1O1N|A	99	K	LRVDPVNFK	L
2338	2	35	B	1.19	2.74	0.478	0.478	gi|27574244|pdb|1O1N|A	99	K	LRVDPVNFK	L
2486	2	35	A	2.42	2.7	0.322	0.322	gi|12833511|dbj|BAB22552.1|	31	R	M*FASFPTTK	T
2496	2	35	A	2.42	2.75	0.334	0.334	gi|12833511|dbj|BAB22552.1|	31	R	M*FASFPTTK	T
2328	2	35	B	2.56	2.73	0.337	0.337	gi|12833511|dbj|BAB22552.1|	31	R	M*FASFPTTK	T
2337	2	35	B	2.56	2.73	0.336	0.336	gi|12833511|dbj|BAB22552.1|	31	R	M*FASFPTTK	T
2420	2	35	B	2.34	2.65	0.404	0.404	gi|12833511|dbj|BAB22552.1|	31	R	MFASFPTTK	T
2430	2	35	B	2.34	2.65	0.395	0.395	gi|12833511|dbj|BAB22552.1|	31	R	MFASFPTTK	T
2537	2	35	A	3.01	4.18	0.129	0.129	gi|344254270|gb|EGW10374.1|	99	K	TYFPHFDVSHGSAQVK	A
2376	2	35	B	2.74	4.21	0.124	0.124	gi|344254270|gb|EGW10374.1|	99	K	TYFPHFDVSHGSAQVK	A
2377	3	35	B	2.56	3.37	0.137	0.137	gi|344254270|gb|EGW10374.1|	99	K	TYFPHFDVSHGSAQVK	A
2387	2	35	B	2.74	4.36	0.115	0.115	gi|344254270|gb|EGW10374.1|	99	K	TYFPHFDVSHGSAQVK	A
2904	2	35	A	4.98	3.92	0.179	0.179	gi|12833511|dbj|BAB22552.1|	29	K	VADALANAAGHLDDLPGALSALSDLHAHK	L
2910	2	35	A	4.91	5.41	0.131	0.131	gi|12833511|dbj|BAB22552.1|	29	K	VADALANAAGHLDDLPGALSALSDLHAHK	L
2790	2	35	B	1.47	2.95	0.195	0.195	gi|12833511|dbj|BAB22552.1|	29	K	VADALANAAGHLDDLPGALSALSDLHAHK	L
2873	3	35	A	3.39	9.6	0.921	0.921	gi|159137400|gb|ABW88847.1|	2	K	VADALATAAGHLDDLPGALSALSDLHAHK	L
2879	3	35	A	3.39	8.68	0.941	0.941	gi|159137400|gb|ABW88847.1|	2	K	VADALATAAGHLDDLPGALSALSDLHAHK	L
2755	3	35	B	2.08	6.29	0.955	0.955	gi|159137400|gb|ABW88847.1|	2	K	VADALATAAGHLDDLPGALSALSDLHAHK	L
2809	3	35	B	2.61	9.73	0.929	0.929	gi|159137400|gb|ABW88847.1|	2	K	VADALATAAGHLDDLPGALSALSDLHAHK	L
2683	2	35	A	4.58	4.55	0.597	0.597	gi|122513|sp|P02088.2|HBB1_MOUSE	65	K	VITAFNDGLNHLDSLK	G
3688	2	35	B	0.22	2.8	0.756	0.756	gi|122513|sp|P02088.2|HBB1_MOUSE	65	K	VITAFNDGLNHLDSLK	G
3703	2	35	B	0.13	3.09	0.706	0.706	gi|122513|sp|P02088.2|HBB1_MOUSE	65	K	VITAFNDGLNHLDSLK	G
3832	2	35	B	0.51	2.86	0.664	0.664	gi|122513|sp|P02088.2|HBB1_MOUSE	65	K	VITAFNDGLNHLDSLK	G
3841	2	35	B	0.45	2.73	0.638	0.638	gi|122513|sp|P02088.2|HBB1_MOUSE	65	K	VITAFNDGLNHLDSLK	G
2249	2	35	A	3.98	3.45	0.504	0.504	gi|241913510|pdb|3HRW|C	2	-	VLSGEDKSNIK	A
2255	2	35	A	3.98	3.53	0.517	0.517	gi|241913510|pdb|3HRW|C	2	-	VLSGEDKSNIK	A
2377	2	35	A	3.08	3.77	0.552	0.552	gi|241913510|pdb|3HRW|C	2	-	VLSGEDKSNIK	A
2075	2	35	B	2.45	3.07	0.574	0.574	gi|241913510|pdb|3HRW|C	2	-	VLSGEDKSNIK	A
2212	2	35	B	4.67	2.7	0.556	0.556	gi|241913510|pdb|3HRW|C	2	-	VLSGEDKSNIK	A
2551	2	35	A	1.83	3.26	0	0.171	gi|378548370|sp|B3EWD8.1|HBB_TAMHU	0	K	NVADEVGGEALGR^	L
2565	2	35	A	1.19	3.8	0	0.222	gi|378548370|sp|B3EWD8.1|HBB_TAMHU	0	K	NVADEVGGEALGR^	L
3704	2	35	A	3.7	3.01	0	0.182	gi|378548370|sp|B3EWD8.1|HBB_TAMHU	0	K	NVADEVGGEALGR^	L

Scan	z	Sample ID	Band	PPM	XCorr	Delta correlation	Unique delta correlation	Reference	Redundancy		Peptides	
3846	2	35	A	3.72	3.15	0	0.203	gi|378548370|sp|B3EWD8.1|HBB_TAMHU	0	K	NVADEVGGEALGR^	L
4370	2	35	A	-0.6	3.3	0	0.246	gi|378548370|sp|B3EWD8.1|HBB_TAMHU	0	K	NVADEVGGEALGR^	L
4504	2	35	A	-0.1	4.17	0	0.282	gi|378548370|sp|B3EWD8.1|HBB_TAMHU	0	K	NVADEVGGEALGR^	L
4607	2	35	A	-0.2	3.56	0	0.33	gi|378548370|sp|B3EWD8.1|HBB_TAMHU	0	K	NVADEVGGEALGR^	L
4610	2	35	A	-0.2	3.57	0	0.332	gi|378548370|sp|B3EWD8.1|HBB_TAMHU	0	K	NVADEVGGEALGR^	L
3531	2	35	B	-0.2	3.84	0	0.242	gi|378548370|sp|B3EWD8.1|HBB_TAMHU	0	K	NVADEVGGEALGR^	L
3656	2	35	B	0.06	4.01	0	0.221	gi|378548370|sp|B3EWD8.1|HBB_TAMHU	0	K	NVADEVGGEALGR^	L
4010	2	35	B	-0.2	3.49	0	0.326	gi|378548370|sp|B3EWD8.1|HBB_TAMHU	0	K	NVADEVGGEALGR^	L
4019	2	35	B	-0.2	3.55	0	0.272	gi|378548370|sp|B3EWD8.1|HBB_TAMHU	0	K	NVADEVGGEALGR^	L
4135	2	35	B	-1.2	2.63	0	0.191	gi|378548370|sp|B3EWD8.1|HBB_TAMHU	0	K	NVADEVGGEALGR^	L
4137	2	35	B	-1.1	2.66	0	0.281	gi|378548370|sp|B3EWD8.1|HBB_TAMHU	0	K	NVADEVGGEALGR^	L
4247	2	35	B	-0.2	3.86	0	0.285	gi|378548370|sp|B3EWD8.1|HBB_TAMHU	0	K	NVADEVGGEALGR^	L
4250	2	35	B	-0.2	2.89	0	0.278	gi|378548370|sp|B3EWD8.1|HBB_TAMHU	0	K	NVADEVGGEALGR^	L
4351	2	35	B	-0.8	3.17	0	0.267	gi|378548370|sp|B3EWD8.1|HBB_TAMHU	0	K	NVADEVGGEALGR^	L
4353	2	35	B	-0.8	2.67	0	0.258	gi|378548370|sp|B3EWD8.1|HBB_TAMHU	0	K	NVADEVGGEALGR^	L
2468	2	35	A	3.14	4.08	0.128	0.128	gi|667267074|ref|XP_008569536.1|	4	K	VNVDDVGGEALGR	L
2309	2	35	B	1.54	3.97	0.138	0.138	gi|667267074|ref|XP_008569536.1|	4	K	VNVDDVGGEALGR	L
2435	2	35	A	3.75	3.57	0.88	0.88	gi|12847007|dbj|BAB27399.1|	60	K	VVAGVAAALAHK	Y
2442	2	35	A	3.62	3.38	0.851	0.851	gi|12847007|dbj|BAB27399.1|	60	K	VVAGVAAALAHK	Y
2566	2	35	A	0.37	3.56	0.912	0.912	gi|12847007|dbj|BAB27399.1|	60	K	VVAGVAAALAHK	Y
2569	2	35	A	0.53	4.16	0.885	0.885	gi|12847007|dbj|BAB27399.1|	60	K	VVAGVAAALAHK	Y
2306	2	35	B	2.52	3.62	0.853	0.853	gi|12847007|dbj|BAB27399.1|	60	K	VVAGVAAALAHK	Y
3380	2	35	B	2.79	2.74	0.934	0.934	gi|12847007|dbj|BAB27399.1|	60	K	VVAGVAAALAHK	Y
3518	2	35	B	-0.3	2.67	0.942	0.942	gi|12847007|dbj|BAB27399.1|	60	K	VVAGVAAALAHK	Y
2465	2	35	A	4.36	4.24	0.903	0.903	gi|12847007|dbj|BAB27399.1|	60	K	VVAGVAAALAHKYH	-
2599	2	35	A	1.76	3.9	0.945	0.945	gi|12847007|dbj|BAB27399.1|	60	K	VVAGVAAALAHKYH	-
2608	2	35	A	1.81	3.48	0.936	0.936	gi|12847007|dbj|BAB27399.1|	60	K	VVAGVAAALAHKYH	-
2318	2	35	B	2.3	4.64	0.87	0.87	gi|12847007|dbj|BAB27399.1|	60	K	VVAGVAAALAHKYH	-
2330	2	35	B	2.3	4.08	0.919	0.919	gi|12847007|dbj|BAB27399.1|	60	K	VVAGVAAALAHKYH	-
2749	2	35	A	3.94	5.84	0.484	0.484	gi|187369324|dbj|BAG31393.1|	73	R	YFDSFGDLSSASAIM*GNAK	V
2769	3	35	A	2.59	6.53	0.467	0.467	gi|187369324|dbj|BAG31393.1|	73	R	YFDSFGDLSSASAIM*GNAK	V
2776	3	35	A	2.59	6.17	0.476	0.476	gi|187369324|dbj|BAG31393.1|	73	R	YFDSFGDLSSASAIM*GNAK	V
2865	2	35	A	3.14	5.55	0.533	0.533	gi|187369324|dbj|BAG31393.1|	73	R	YFDSFGDLSSASAIM*GNAK	V
3084	2	35	A	3.07	4.32	0.559	0.559	gi|187369324|dbj|BAG31393.1|	73	R	YFDSFGDLSSASAIM*GNAK	V
3139	2	35	A	3.2	4.62	0.546	0.546	gi|187369324|dbj|BAG31393.1|	73	R	YFDSFGDLSSASAIM*GNAK	V
3151	2	35	A	3.21	2.52	0.543	0.543	gi|187369324|dbj|BAG31393.1|	73	R	YFDSFGDLSSASAIM*GNAK	V
3288	2	35	A	2.61	3.08	0.614	0.614	gi|187369324|dbj|BAG31393.1|	73	R	YFDSFGDLSSASAIM*GNAK	V
3294	2	35	A	2.65	2.55	0.629	0.629	gi|187369324|dbj|BAG31393.1|	73	R	YFDSFGDLSSASAIM*GNAK	V

Scan	z	Sample ID	Band	PPM	XCorr	Delta correlation	Unique delta correlation	Reference	Redundancy		Peptides	
3616	2	35	A	3.67	2.5	0.647	0.647	gi|187369324|dbj|BAG31393.1|	73	R	YFDSFGDLSSASAIM*GNAK	V
3765	2	35	A	4.78	3.64	0.659	0.659	gi|187369324|dbj|BAG31393.1|	73	R	YFDSFGDLSSASAIM*GNAK	V
3775	2	35	A	4.22	3.64	0.631	0.631	gi|187369324|dbj|BAG31393.1|	73	R	YFDSFGDLSSASAIM*GNAK	V
3913	2	35	A	4.38	2.96	0.537	0.537	gi|187369324|dbj|BAG31393.1|	73	R	YFDSFGDLSSASAIM*GNAK	V
4195	3	35	A	3.76	4.02	0.364	0.364	gi|187369324|dbj|BAG31393.1|	73	R	YFDSFGDLSSASAIM*GNAK	V
4362	2	35	A	-0.3	4.28	0.578	0.578	gi|187369324|dbj|BAG31393.1|	73	R	YFDSFGDLSSASAIM*GNAK	V
4371	2	35	A	-0.3	4.06	0.708	0.708	gi|187369324|dbj|BAG31393.1|	73	R	YFDSFGDLSSASAIM*GNAK	V
4412	3	35	A	-0.7	3.87	0.384	0.384	gi|187369324|dbj|BAG31393.1|	73	R	YFDSFGDLSSASAIM*GNAK	V
4436	3	35	A	0.39	3.53	0.343	0.343	gi|187369324|dbj|BAG31393.1|	73	R	YFDSFGDLSSASAIM*GNAK	V
4482	2	35	A	0.19	4.93	0.611	0.611	gi|187369324|dbj|BAG31393.1|	73	R	YFDSFGDLSSASAIM*GNAK	V
4493	2	35	A	0.23	4.87	0.603	0.603	gi|187369324|dbj|BAG31393.1|	73	R	YFDSFGDLSSASAIM*GNAK	V
4739	3	35	A	-0.3	4.51	0.436	0.436	gi|187369324|dbj|BAG31393.1|	73	R	YFDSFGDLSSASAIM*GNAK	V
4804	2	35	A	-0.6	3.42	0.668	0.668	gi|187369324|dbj|BAG31393.1|	73	R	YFDSFGDLSSASAIM*GNAK	V
4807	2	35	A	-0.6	3.13	0.681	0.681	gi|187369324|dbj|BAG31393.1|	73	R	YFDSFGDLSSASAIM*GNAK	V
2594	2	35	B	3.35	7.17	0.435	0.435	gi|187369324|dbj|BAG31393.1|	73	R	YFDSFGDLSSASAIM*GNAK	V
2598	3	35	B	0.79	6.77	0.424	0.424	gi|187369324|dbj|BAG31393.1|	73	R	YFDSFGDLSSASAIM*GNAK	V
2605	2	35	B	3.39	5.07	0.517	0.517	gi|187369324|dbj|BAG31393.1|	73	R	YFDSFGDLSSASAIM*GNAK	V
2607	3	35	B	0.8	7.38	0.402	0.402	gi|187369324|dbj|BAG31393.1|	73	R	YFDSFGDLSSASAIM*GNAK	V
2731	2	35	B	0.65	4.56	0.553	0.553	gi|187369324|dbj|BAG31393.1|	73	R	YFDSFGDLSSASAIM*GNAK	V
2742	2	35	B	0.37	3.95	0.565	0.565	gi|187369324|dbj|BAG31393.1|	73	R	YFDSFGDLSSASAIM*GNAK	V
2861	2	35	B	1.18	4.57	0.527	0.527	gi|187369324|dbj|BAG31393.1|	73	R	YFDSFGDLSSASAIM*GNAK	V
2868	2	35	B	1.24	4.3	0.584	0.584	gi|187369324|dbj|BAG31393.1|	73	R	YFDSFGDLSSASAIM*GNAK	V
3355	2	35	B	3.58	4.5	0.619	0.619	gi|187369324|dbj|BAG31393.1|	73	R	YFDSFGDLSSASAIM*GNAK	V
3371	2	35	B	4.48	4.95	0.59	0.59	gi|187369324|dbj|BAG31393.1|	73	R	YFDSFGDLSSASAIM*GNAK	V
3490	2	35	B	0.9	4.17	0.664	0.664	gi|187369324|dbj|BAG31393.1|	73	R	YFDSFGDLSSASAIM*GNAK	V
3554	3	35	B	-0.5	4.06	0.384	0.384	gi|187369324|dbj|BAG31393.1|	73	R	YFDSFGDLSSASAIM*GNAK	V
3560	3	35	B	-0.7	4.06	0.448	0.448	gi|187369324|dbj|BAG31393.1|	73	R	YFDSFGDLSSASAIM*GNAK	V
3608	2	35	B	1.23	5.28	0.575	0.575	gi|187369324|dbj|BAG31393.1|	73	R	YFDSFGDLSSASAIM*GNAK	V
3611	2	35	B	0.84	5.77	0.559	0.559	gi|187369324|dbj|BAG31393.1|	73	R	YFDSFGDLSSASAIM*GNAK	V
3674	3	35	B	-0.3	3.58	0.457	0.457	gi|187369324|dbj|BAG31393.1|	73	R	YFDSFGDLSSASAIM*GNAK	V
3678	3	35	B	-0	4.27	0.46	0.46	gi|187369324|dbj|BAG31393.1|	73	R	YFDSFGDLSSASAIM*GNAK	V
3725	2	35	B	0.46	5.17	0.619	0.619	gi|187369324|dbj|BAG31393.1|	73	R	YFDSFGDLSSASAIM*GNAK	V
3730	2	35	B	0.45	5.69	0.572	0.572	gi|187369324|dbj|BAG31393.1|	73	R	YFDSFGDLSSASAIM*GNAK	V
3793	3	35	B	0.57	3.6	0.339	0.339	gi|187369324|dbj|BAG31393.1|	73	R	YFDSFGDLSSASAIM*GNAK	V
3796	3	35	B	0.64	3.78	0.441	0.441	gi|187369324|dbj|BAG31393.1|	73	R	YFDSFGDLSSASAIM*GNAK	V
3849	2	35	B	0.47	5.31	0.608	0.608	gi|187369324|dbj|BAG31393.1|	73	R	YFDSFGDLSSASAIM*GNAK	V
3851	2	35	B	0.38	5.02	0.581	0.581	gi|187369324|dbj|BAG31393.1|	73	R	YFDSFGDLSSASAIM*GNAK	V
3923	3	35	B	-0.2	3.51	0.384	0.384	gi|187369324|dbj|BAG31393.1|	73	R	YFDSFGDLSSASAIM*GNAK	V

Scan	z	Sample ID	Band	PPM	XCorr	Delta correlation	Unique delta correlation	Reference	Redundancy		Peptides	
3963	2	35	B	0.28	4.6	0.54	0.54	gi|187369324|dbj|BAG31393.1|	73	R	YFDSFGDLSSASAIM*GNAK	V
3968	2	35	B	0.33	4.09	0.686	0.686	gi|187369324|dbj|BAG31393.1|	73	R	YFDSFGDLSSASAIM*GNAK	V
4081	2	35	B	-0.3	2.79	0.754	0.754	gi|187369324|dbj|BAG31393.1|	73	R	YFDSFGDLSSASAIM*GNAK	V
4085	2	35	B	-0.2	2.96	0.738	0.738	gi|187369324|dbj|BAG31393.1|	73	R	YFDSFGDLSSASAIM*GNAK	V
4204	2	35	B	0.02	3.69	0.613	0.613	gi|187369324|dbj|BAG31393.1|	73	R	YFDSFGDLSSASAIM*GNAK	V
4208	2	35	B	0.03	3.64	0.633	0.633	gi|187369324|dbj|BAG31393.1|	73	R	YFDSFGDLSSASAIM*GNAK	V
4420	2	35	B	-0.4	2.98	0.638	0.638	gi|187369324|dbj|BAG31393.1|	73	R	YFDSFGDLSSASAIM*GNAK	V
2903	2	35	A	4.27	7.3	0.478	0.478	gi|187369324|dbj|BAG31393.1|	73	R	YFDSFGDLSSASAIMGNAK	V
2920	2	35	A	3.4	6.98	0.523	0.523	gi|187369324|dbj|BAG31393.1|	73	R	YFDSFGDLSSASAIMGNAK	V
3030	3	35	A	3.48	3.85	0.292	0.292	gi|187369324|dbj|BAG31393.1|	73	R	YFDSFGDLSSASAIMGNAK	V
3032	2	35	A	3.37	3.18	0.585	0.585	gi|187369324|dbj|BAG31393.1|	73	R	YFDSFGDLSSASAIMGNAK	V
3039	2	35	A	3.32	2.77	0.695	0.695	gi|187369324|dbj|BAG31393.1|	73	R	YFDSFGDLSSASAIMGNAK	V
3200	2	35	A	2.97	3.3	0.684	0.684	gi|187369324|dbj|BAG31393.1|	73	R	YFDSFGDLSSASAIMGNAK	V
3204	2	35	A	2.88	3.8	0.628	0.628	gi|187369324|dbj|BAG31393.1|	73	R	YFDSFGDLSSASAIMGNAK	V
3332	2	35	A	3.4	2.7	0.587	0.587	gi|187369324|dbj|BAG31393.1|	73	R	YFDSFGDLSSASAIMGNAK	V
3336	2	35	A	4.11	2.5	0.463	0.463	gi|187369324|dbj|BAG31393.1|	73	R	YFDSFGDLSSASAIMGNAK	V
3471	2	35	A	4.11	3.39	0.657	0.657	gi|187369324|dbj|BAG31393.1|	73	R	YFDSFGDLSSASAIMGNAK	V
3473	2	35	A	4.25	4.65	0.649	0.649	gi|187369324|dbj|BAG31393.1|	73	R	YFDSFGDLSSASAIMGNAK	V
3613	2	35	A	4.17	2.77	0.602	0.602	gi|187369324|dbj|BAG31393.1|	73	R	YFDSFGDLSSASAIMGNAK	V
3615	2	35	A	3.62	3.15	0.565	0.565	gi|187369324|dbj|BAG31393.1|	73	R	YFDSFGDLSSASAIMGNAK	V
3747	2	35	A	3.06	3.9	0.624	0.624	gi|187369324|dbj|BAG31393.1|	73	R	YFDSFGDLSSASAIMGNAK	V
3749	2	35	A	3.2	2.55	0.573	0.573	gi|187369324|dbj|BAG31393.1|	73	R	YFDSFGDLSSASAIMGNAK	V
3879	2	35	A	4.31	3.21	0.669	0.669	gi|187369324|dbj|BAG31393.1|	73	R	YFDSFGDLSSASAIMGNAK	V
3884	2	35	A	4.52	2.7	0.622	0.622	gi|187369324|dbj|BAG31393.1|	73	R	YFDSFGDLSSASAIMGNAK	V
4321	2	35	A	-0.3	3.07	0.726	0.726	gi|187369324|dbj|BAG31393.1|	73	R	YFDSFGDLSSASAIMGNAK	V
4414	3	35	A	-0.8	3.14	0.197	0.197	gi|187369324|dbj|BAG31393.1|	73	R	YFDSFGDLSSASAIMGNAK	V
4550	2	35	A	0.13	4.68	0.593	0.593	gi|187369324|dbj|BAG31393.1|	73	R	YFDSFGDLSSASAIMGNAK	V
4555	2	35	A	0.08	3.57	0.689	0.689	gi|187369324|dbj|BAG31393.1|	73	R	YFDSFGDLSSASAIMGNAK	V
2750	2	35	B	3.44	6.45	0.528	0.528	gi|187369324|dbj|BAG31393.1|	73	R	YFDSFGDLSSASAIMGNAK	V
2760	3	35	B	0.95	6.13	0.364	0.364	gi|187369324|dbj|BAG31393.1|	73	R	YFDSFGDLSSASAIMGNAK	V
2771	3	35	B	0.95	6.16	0.365	0.365	gi|187369324|dbj|BAG31393.1|	73	R	YFDSFGDLSSASAIMGNAK	V
2889	2	35	B	1.18	4.63	0.53	0.53	gi|187369324|dbj|BAG31393.1|	73	R	YFDSFGDLSSASAIMGNAK	V
2898	2	35	B	1.22	2.65	0.534	0.534	gi|187369324|dbj|BAG31393.1|	73	R	YFDSFGDLSSASAIMGNAK	V
3505	2	35	B	0.01	4.92	0.636	0.636	gi|187369324|dbj|BAG31393.1|	73	R	YFDSFGDLSSASAIMGNAK	V
3520	2	35	B	0.09	4.39	0.631	0.631	gi|187369324|dbj|BAG31393.1|	73	R	YFDSFGDLSSASAIMGNAK	V
3638	2	35	B	0.49	5.81	0.56	0.56	gi|187369324|dbj|BAG31393.1|	73	R	YFDSFGDLSSASAIMGNAK	V
3644	2	35	B	0.48	6.02	0.56	0.56	gi|187369324|dbj|BAG31393.1|	73	R	YFDSFGDLSSASAIMGNAK	V
3759	2	35	B	0.79	4.64	0.683	0.683	gi|187369324|dbj|BAG31393.1|	73	R	YFDSFGDLSSASAIMGNAK	V

Scan	z	Sample ID	Band	PPM	XCorr	Delta correlation	Unique delta correlation	Reference	Redundancy		Peptides	
3770	2	35	B	1.11	5.47	0.611	0.611	gi|187369324|dbj|BAG31393.1|	73	R	YFDSFGDLSSASAIMGNAK	V
3890	2	35	B	0.65	4.89	0.596	0.596	gi|187369324|dbj|BAG31393.1|	73	R	YFDSFGDLSSASAIMGNAK	V
3894	2	35	B	0.57	4.18	0.682	0.682	gi|187369324|dbj|BAG31393.1|	73	R	YFDSFGDLSSASAIMGNAK	V
4020	2	35	B	0.4	2.81	0.712	0.712	gi|187369324|dbj|BAG31393.1|	73	R	YFDSFGDLSSASAIMGNAK	V
4027	2	35	B	0.33	3.05	0.708	0.708	gi|187369324|dbj|BAG31393.1|	73	R	YFDSFGDLSSASAIMGNAK	V
4151	2	35	B	-0.3	2.75	0.573	0.573	gi|187369324|dbj|BAG31393.1|	73	R	YFDSFGDLSSASAIMGNAK	V
3282	2	38	A	1.37	2.94	0.928	0.928	gi|554577472|ref|XP_005880462.1|	74	K	AAVSGLWGK	V
5473	2	38	A	-0.1	2.66	0.98	0.98	gi|554577472|ref|XP_005880462.1|	74	K	AAVSGLWGK	V
5649	2	38	A	-0.4	2.7	0.935	0.935	gi|554577472|ref|XP_005880462.1|	74	K	AAVSGLWGK	V
5796	2	38	A	-0.7	2.57	0.935	0.935	gi|554577472|ref|XP_005880462.1|	74	K	AAVSGLWGK	V
35	2	38	B	-0.2	2.52	0.98	0.98	gi|554577472|ref|XP_005880462.1|	74	K	AAVSGLWGK	V
156	2	38	B	-0.6	2.67	0.968	0.968	gi|554577472|ref|XP_005880462.1|	74	K	AAVSGLWGK	V
159	2	38	B	-0.8	2.67	0.939	0.939	gi|554577472|ref|XP_005880462.1|	74	K	AAVSGLWGK	V
348	2	38	B	-0.4	2.58	0.971	0.971	gi|554577472|ref|XP_005880462.1|	74	K	AAVSGLWGK	V
3334	2	38	B	1.66	3.04	0.92	0.92	gi|554577472|ref|XP_005880462.1|	74	K	AAVSGLWGK	V
3338	2	38	B	1.66	3.44	0.923	0.923	gi|554577472|ref|XP_005880462.1|	74	K	AAVSGLWGK	V
3279	2	38	A	1.54	4.02	0.958	0.958	gi|532054982|ref|XP_005370208.1|	94	K	DFTPAAQAAFQK	V
3283	2	38	A	1.53	4.04	0.96	0.96	gi|532054982|ref|XP_005370208.1|	94	K	DFTPAAQAAFQK	V
346	2	38	B	-0.5	2.69	0.949	0.949	gi|532054982|ref|XP_005370208.1|	94	K	DFTPAAQAAFQK	V
355	2	38	B	-0.5	2.76	0.991	0.991	gi|532054982|ref|XP_005370208.1|	94	K	DFTPAAQAAFQK	V
3304	2	38	B	2.63	4.11	0.955	0.955	gi|532054982|ref|XP_005370208.1|	94	K	DFTPAAQAAFQK	V
3306	3	38	B	0.08	3.75	0.826	0.826	gi|532054982|ref|XP_005370208.1|	94	K	DFTPAAQAAFQK	V
3315	2	38	B	2.66	4.08	0.967	0.967	gi|532054982|ref|XP_005370208.1|	94	K	DFTPAAQAAFQK	V
3316	3	38	B	0.08	4.13	0.821	0.821	gi|532054982|ref|XP_005370208.1|	94	K	DFTPAAQAAFQK	V
3483	2	38	B	-0.3	2.59	0.985	0.985	gi|532054982|ref|XP_005370208.1|	94	K	DFTPAAQAAFQK	V
3600	2	38	A	-0.4	3.33	0	0.324	gi|27574244|pdb|1O1N|A	0	K	FLASVSTVLTSK	Y
5447	2	38	A	0.21	2.94	0	0.359	gi|27574244|pdb|1O1N|A	0	K	FLASVSTVLTSK	Y
5577	2	38	A	-0.3	2.56	0	0.417	gi|27574244|pdb|1O1N|A	0	K	FLASVSTVLTSK	Y
5580	2	38	A	-0.3	2.75	0	0.395	gi|27574244|pdb|1O1N|A	0	K	FLASVSTVLTSK	Y
8	2	38	B	0.11	2.81	0	0.335	gi|27574244|pdb|1O1N|A	0	K	FLASVSTVLTSK	Y
261	2	38	B	-0.1	2.56	0	0.4	gi|27574244|pdb|1O1N|A	0	K	FLASVSTVLTSK	Y
3651	2	38	B	1.3	3.93	0	0.363	gi|27574244|pdb|1O1N|A	0	K	FLASVSTVLTSK	Y
3140	2	38	A	1.54	4.53	0.109	0.109	gi|344254270|gb|EGW10374.1|	99	K	IGGHGAEYGAEALER	M
3449	3	38	B	0.14	5.02	0.553	0.553	gi|122513|sp|P02088.2|HBB1_MOUSE	65	K	KVITAFNDGLNHLDSLK	G
3210	3	38	B	-0.3	3.24	0.855	0.855	gi|431903465|gb|ELK09417.1|	99	K	LHVDPENFR	L
3736	2	38	A	-0.3	4.29	0.225	0.225	gi|122513|sp|P02088.2|HBB1_MOUSE	88	R	LLGNM*IVIVLGHHLGK	D
3747	2	38	A	0.84	3.6	0.215	0.215	gi|122513|sp|P02088.2|HBB1_MOUSE	88	R	LLGNM*IVIVLGHHLGK	D
3835	2	38	B	0.06	3.28	0.229	0.229	gi|122513|sp|P02088.2|HBB1_MOUSE	88	R	LLGNM*IVIVLGHHLGK	D

Scan	z	Sample ID	Band	PPM	XCorr	Delta correlation	Unique delta correlation	Reference	Redundancy		Peptides	
4009	2	38	A	0.23	4.18	0.203	0.203	gi|122513|sp|P02088.2|HBB1_MOUSE	88	R	LLGNMIVIVLGHHLGK	D
4019	2	38	A	0.64	4.24	0.209	0.209	gi|122513|sp|P02088.2|HBB1_MOUSE	88	R	LLGNMIVIVLGHHLGK	D
4171	2	38	B	-0.1	4.31	0.208	0.208	gi|122513|sp|P02088.2|HBB1_MOUSE	88	R	LLGNMIVIVLGHHLGK	D
4177	2	38	B	-0.1	4.62	0.246	0.246	gi|122513|sp|P02088.2|HBB1_MOUSE	88	R	LLGNMIVIVLGHHLGK	D
3233	2	38	A	2.5	2.55	0.447	0.447	gi|27574244|pdb|1O1N|A	99	K	LRVDPVNFK	L
3286	2	38	B	0.16	2.78	0.422	0.422	gi|27574244|pdb|1O1N|A	99	K	LRVDPVNFK	L
3293	2	38	B	0.16	2.54	0.483	0.483	gi|27574244|pdb|1O1N|A	99	K	LRVDPVNFK	L
3283	2	38	B	1.17	2.7	0.305	0.305	gi|12833511|dbj|BAB22552.1|	31	R	M*FASFPTTK	T
3299	3	38	A	0.87	3.7	0.125	0.125	gi|344254270|gb|EGW10374.1|	99	K	TYFPHFDVSHGSAQVK	A
3300	2	38	A	0.83	4.24	0.112	0.112	gi|344254270|gb|EGW10374.1|	99	K	TYFPHFDVSHGSAQVK	A
3308	3	38	A	0.87	3.56	0.125	0.125	gi|344254270|gb|EGW10374.1|	99	K	TYFPHFDVSHGSAQVK	A
3309	2	38	A	0.99	4.16	0.121	0.121	gi|344254270|gb|EGW10374.1|	99	K	TYFPHFDVSHGSAQVK	A
3360	3	38	B	0.78	3.07	0.147	0.147	gi|344254270|gb|EGW10374.1|	99	K	TYFPHFDVSHGSAQVK	A
3361	2	38	B	0.23	3.97	0.123	0.123	gi|344254270|gb|EGW10374.1|	99	K	TYFPHFDVSHGSAQVK	A
3368	3	38	B	0.78	3.37	0.138	0.138	gi|344254270|gb|EGW10374.1|	99	K	TYFPHFDVSHGSAQVK	A
3369	2	38	B	-0.2	4.19	0.111	0.111	gi|344254270|gb|EGW10374.1|	99	K	TYFPHFDVSHGSAQVK	A
4059	3	38	B	-1.2	3.72	0.819	0.819	gi|159137120|gb|ABW88707.1|	79	K	VADALATAADNLDDLPGALSALSDLHAHK	L
4035	3	38	A	-0.2	8.31	0.915	0.915	gi|159137400|gb|ABW88847.1|	2	K	VADALATAAGHLDDLPGALSALSDLHAHK	L
4043	3	38	A	-0.3	8.67	0.898	0.898	gi|159137400|gb|ABW88847.1|	2	K	VADALATAAGHLDDLPGALSALSDLHAHK	L
4183	3	38	A	-0.4	7.7	0.916	0.916	gi|159137400|gb|ABW88847.1|	2	K	VADALATAAGHLDDLPGALSALSDLHAHK	L
4093	3	38	B	-0.9	8.35	0.905	0.905	gi|159137400|gb|ABW88847.1|	2	K	VADALATAAGHLDDLPGALSALSDLHAHK	L
4099	3	38	B	1.92	8.78	0.902	0.902	gi|159137400|gb|ABW88847.1|	2	K	VADALATAAGHLDDLPGALSALSDLHAHK	L
3148	3	38	B	-1.9	3.14	0.324	0.324	gi|414524181|gb|AFX00018.1|	5	K	VGSHAGEYGAEALER	M
3268	2	38	B	-0.1	3.56	0.503	0.503	gi|414524181|gb|AFX00018.1|	5	K	VGSHAGEYGAEALER	M
3590	2	38	A	2.38	5.73	0.57	0.57	gi|122513|sp|P02088.2|HBB1_MOUSE	65	K	VITAFNDGLNHLDSLK	G
3560	2	38	B	0.23	4.91	0.615	0.615	gi|122513|sp|P02088.2|HBB1_MOUSE	65	K	VITAFNDGLNHLDSLK	G
3568	2	38	B	0.23	3.78	0.636	0.636	gi|122513|sp|P02088.2|HBB1_MOUSE	65	K	VITAFNDGLNHLDSLK	G
3681	2	38	B	0.31	5.48	0.591	0.591	gi|122513|sp|P02088.2|HBB1_MOUSE	65	K	VITAFNDGLNHLDSLK	G
3692	2	38	B	0.31	5.41	0.6	0.6	gi|122513|sp|P02088.2|HBB1_MOUSE	65	K	VITAFNDGLNHLDSLK	G
2911	2	38	B	-0.8	2.77	0.392	0.392	gi|241913510|pdb|3HRW|C	2	-	VLSGEDKSNIK	A
5411	2	38	A	-0.1	3.02	0	0.336	gi|378548370|sp|B3EWD8.1|HBB_TAMHU	0	K	NVADEVGGEALGR^	L
5540	2	38	A	-0.5	3.71	0	0.253	gi|378548370|sp|B3EWD8.1|HBB_TAMHU	0	K	NVADEVGGEALGR^	L
5545	2	38	A	-0.3	3.38	0	0.217	gi|378548370|sp|B3EWD8.1|HBB_TAMHU	0	K	NVADEVGGEALGR^	L
5665	2	38	A	-0.5	3.54	0	0.274	gi|378548370|sp|B3EWD8.1|HBB_TAMHU	0	K	NVADEVGGEALGR^	L
5667	2	38	A	-0.5	3.43	0	0.236	gi|378548370|sp|B3EWD8.1|HBB_TAMHU	0	K	NVADEVGGEALGR^	L
5790	2	38	A	-0.1	3.24	0	0.193	gi|378548370|sp|B3EWD8.1|HBB_TAMHU	0	K	NVADEVGGEALGR^	L
5793	2	38	A	-0.2	3.72	0	0.296	gi|378548370|sp|B3EWD8.1|HBB_TAMHU	0	K	NVADEVGGEALGR^	L
7	2	38	B	0.15	3.8	0	0.255	gi|378548370|sp|B3EWD8.1|HBB_TAMHU	0	K	NVADEVGGEALGR^	L

Scan	z	Sample ID	Band	PPM	XCorr	Delta correlation	Unique delta correlation	Reference	Redundancy		Peptides	
20	2	38	B	0.16	3.64	0	0.23	gi|378548370|sp|B3EWD8.1|HBB_TAMHU	0	K	NVADEVGGEALGR^	L
136	2	38	B	-0.1	3.46	0	0.202	gi|378548370|sp|B3EWD8.1|HBB_TAMHU	0	K	NVADEVGGEALGR^	L
260	2	38	B	-0.5	3.57	0	0.254	gi|378548370|sp|B3EWD8.1|HBB_TAMHU	0	K	NVADEVGGEALGR^	L
366	2	38	B	-0.4	4.12	0	0.229	gi|378548370|sp|B3EWD8.1|HBB_TAMHU	0	K	NVADEVGGEALGR^	L
377	2	38	B	-0.4	3.31	0	0.229	gi|378548370|sp|B3EWD8.1|HBB_TAMHU	0	K	NVADEVGGEALGR^	L
3090	2	38	B	0.43	2.75	0	0.2	gi|378548370|sp|B3EWD8.1|HBB_TAMHU	0	K	NVADEVGGEALGR^	L
3260	2	38	B	-0.3	3.84	0	0.241	gi|378548370|sp|B3EWD8.1|HBB_TAMHU	0	K	NVADEVGGEALGR^	L
3409	2	38	B	-0.4	2.92	0	0.296	gi|378548370|sp|B3EWD8.1|HBB_TAMHU	0	K	NVADEVGGEALGR^	L
4147	2	38	B	-1	2.95	0	0.249	gi|378548370|sp|B3EWD8.1|HBB_TAMHU	0	K	NVADEVGGEALGR^	L
4274	2	38	B	-1.5	2.77	0	0.355	gi|378548370|sp|B3EWD8.1|HBB_TAMHU	0	K	NVADEVGGEALGR^	L
4436	2	38	B	-0.2	3.2	0	0.268	gi|378548370|sp|B3EWD8.1|HBB_TAMHU	0	K	NVADEVGGEALGR^	L
4446	2	38	B	0.18	2.92	0	0.235	gi|378548370|sp|B3EWD8.1|HBB_TAMHU	0	K	NVADEVGGEALGR^	L
4572	2	38	B	-0.1	2.8	0	0.204	gi|378548370|sp|B3EWD8.1|HBB_TAMHU	0	K	NVADEVGGEALGR^	L
4579	2	38	B	-0.7	2.93	0	0.204	gi|378548370|sp|B3EWD8.1|HBB_TAMHU	0	K	NVADEVGGEALGR^	L
4702	2	38	B	0.3	3.29	0	0.248	gi|378548370|sp|B3EWD8.1|HBB_TAMHU	0	K	NVADEVGGEALGR^	L
4705	2	38	B	0.15	3.13	0	0.273	gi|378548370|sp|B3EWD8.1|HBB_TAMHU	0	K	NVADEVGGEALGR^	L
4839	2	38	B	-0.1	3.15	0	0.229	gi|378548370|sp|B3EWD8.1|HBB_TAMHU	0	K	NVADEVGGEALGR^	L
4842	2	38	B	-0	2.7	0	0.22	gi|378548370|sp|B3EWD8.1|HBB_TAMHU	0	K	NVADEVGGEALGR^	L
4975	2	38	B	-0.1	3.16	0	0.264	gi|378548370|sp|B3EWD8.1|HBB_TAMHU	0	K	NVADEVGGEALGR^	L
4982	2	38	B	-0.1	3.18	0	0.259	gi|378548370|sp|B3EWD8.1|HBB_TAMHU	0	K	NVADEVGGEALGR^	L
5116	2	38	B	-0.2	3.55	0	0.285	gi|378548370|sp|B3EWD8.1|HBB_TAMHU	0	K	NVADEVGGEALGR^	L
5251	2	38	B	-0	3.17	0	0.231	gi|378548370|sp|B3EWD8.1|HBB_TAMHU	0	K	NVADEVGGEALGR^	L
3170	2	38	A	1.43	3.94	0.84	0.84	gi|12847007|dbj|BAB27399.1|	60	K	VVAGVAAALAHK	Y
3181	2	38	A	1.96	3.82	0.874	0.874	gi|12847007|dbj|BAB27399.1|	60	K	VVAGVAAALAHK	Y
48	2	38	B	0.29	2.79	0.881	0.881	gi|12847007|dbj|BAB27399.1|	60	K	VVAGVAAALAHK	Y
53	2	38	B	-0.1	2.77	0.942	0.942	gi|12847007|dbj|BAB27399.1|	60	K	VVAGVAAALAHK	Y
173	2	38	B	-0.3	3.06	0.956	0.956	gi|12847007|dbj|BAB27399.1|	60	K	VVAGVAAALAHK	Y
175	2	38	B	-0.2	3.09	0.887	0.887	gi|12847007|dbj|BAB27399.1|	60	K	VVAGVAAALAHK	Y
297	2	38	B	-0.6	2.82	0.947	0.947	gi|12847007|dbj|BAB27399.1|	60	K	VVAGVAAALAHK	Y
364	2	38	B	0.29	3.1	0.949	0.949	gi|12847007|dbj|BAB27399.1|	60	K	VVAGVAAALAHK	Y
371	2	38	B	0.26	2.98	0.968	0.968	gi|12847007|dbj|BAB27399.1|	60	K	VVAGVAAALAHK	Y
3222	2	38	B	-0.5	3.29	0.903	0.903	gi|12847007|dbj|BAB27399.1|	60	K	VVAGVAAALAHK	Y
3231	2	38	B	2.94	3.23	0.812	0.812	gi|12847007|dbj|BAB27399.1|	60	K	VVAGVAAALAHK	Y
3236	2	38	A	2.43	4.79	0.931	0.931	gi|12847007|dbj|BAB27399.1|	60	K	VVAGVAAALAHKYH	-
3247	2	38	A	2.43	4.07	0.916	0.916	gi|12847007|dbj|BAB27399.1|	60	K	VVAGVAAALAHKYH	-
3284	2	38	B	0.08	4.67	0.898	0.898	gi|12847007|dbj|BAB27399.1|	60	K	VVAGVAAALAHKYH	-
3649	2	38	A	1.33	5.21	0.605	0.605	gi|187369324|dbj|BAG31393.1|	73	R	YFDSFGDLSSASAIM*GNAK	V
3655	3	38	A	-0.2	5.39	0.458	0.458	gi|187369324|dbj|BAG31393.1|	73	R	YFDSFGDLSSASAIM*GNAK	V

Scan	z	Sample ID	Band	PPM	XCorr	Delta correlation	Unique delta correlation	Reference	Redundancy		Peptides	
3658	2	38	A	1.36	6.63	0.471	0.471	gi|187369324|dbj|BAG31393.1|	73	R	YFDSFGDLSSASAIM*GNAK	V
3660	3	38	A	-0.1	6.65	0.428	0.428	gi|187369324|dbj|BAG31393.1|	73	R	YFDSFGDLSSASAIM*GNAK	V
3800	2	38	A	-0.4	4.21	0.556	0.556	gi|187369324|dbj|BAG31393.1|	73	R	YFDSFGDLSSASAIM*GNAK	V
3806	2	38	A	0.43	5.84	0.572	0.572	gi|187369324|dbj|BAG31393.1|	73	R	YFDSFGDLSSASAIM*GNAK	V
4101	2	38	A	-1.1	5.4	0.518	0.518	gi|187369324|dbj|BAG31393.1|	73	R	YFDSFGDLSSASAIM*GNAK	V
4106	2	38	A	-0.3	5.27	0.546	0.546	gi|187369324|dbj|BAG31393.1|	73	R	YFDSFGDLSSASAIM*GNAK	V
4255	2	38	A	-1.5	3.55	0.599	0.599	gi|187369324|dbj|BAG31393.1|	73	R	YFDSFGDLSSASAIM*GNAK	V
4372	2	38	A	-0.5	4.89	0.579	0.579	gi|187369324|dbj|BAG31393.1|	73	R	YFDSFGDLSSASAIM*GNAK	V
4376	2	38	A	-1.8	4.56	0.592	0.592	gi|187369324|dbj|BAG31393.1|	73	R	YFDSFGDLSSASAIM*GNAK	V
4530	2	38	A	2.81	4.15	0.593	0.593	gi|187369324|dbj|BAG31393.1|	73	R	YFDSFGDLSSASAIM*GNAK	V
4593	2	38	A	-0.5	2.83	0.704	0.704	gi|187369324|dbj|BAG31393.1|	73	R	YFDSFGDLSSASAIM*GNAK	V
4636	2	38	A	1.35	2.64	0.751	0.751	gi|187369324|dbj|BAG31393.1|	73	R	YFDSFGDLSSASAIM*GNAK	V
5581	2	38	A	-0.8	3.22	0.672	0.672	gi|187369324|dbj|BAG31393.1|	73	R	YFDSFGDLSSASAIM*GNAK	V
5767	2	38	A	0.87	4.08	0.647	0.647	gi|187369324|dbj|BAG31393.1|	73	R	YFDSFGDLSSASAIM*GNAK	V
5785	2	38	A	0.94	3.15	0.701	0.701	gi|187369324|dbj|BAG31393.1|	73	R	YFDSFGDLSSASAIM*GNAK	V
5788	3	38	A	-1	3.08	0.428	0.428	gi|187369324|dbj|BAG31393.1|	73	R	YFDSFGDLSSASAIM*GNAK	V
63	2	38	B	0.13	3.14	0.611	0.611	gi|187369324|dbj|BAG31393.1|	73	R	YFDSFGDLSSASAIM*GNAK	V
76	3	38	B	-0.6	3.71	0.499	0.499	gi|187369324|dbj|BAG31393.1|	73	R	YFDSFGDLSSASAIM*GNAK	V
103	3	38	B	0.26	3.08	0.464	0.464	gi|187369324|dbj|BAG31393.1|	73	R	YFDSFGDLSSASAIM*GNAK	V
106	2	38	B	1.08	3.51	0.66	0.66	gi|187369324|dbj|BAG31393.1|	73	R	YFDSFGDLSSASAIM*GNAK	V
202	2	38	B	0.09	3.44	0.634	0.634	gi|187369324|dbj|BAG31393.1|	73	R	YFDSFGDLSSASAIM*GNAK	V
226	2	38	B	3.2	4.14	0.716	0.716	gi|187369324|dbj|BAG31393.1|	73	R	YFDSFGDLSSASAIM*GNAK	V
243	3	38	B	-1.1	3.32	0.483	0.483	gi|187369324|dbj|BAG31393.1|	73	R	YFDSFGDLSSASAIM*GNAK	V
361	2	38	B	-0.3	3.73	0.662	0.662	gi|187369324|dbj|BAG31393.1|	73	R	YFDSFGDLSSASAIM*GNAK	V
3693	2	38	B	1.87	6.18	0.541	0.541	gi|187369324|dbj|BAG31393.1|	73	R	YFDSFGDLSSASAIM*GNAK	V
3807	2	38	B	0.05	3.97	0.573	0.573	gi|187369324|dbj|BAG31393.1|	73	R	YFDSFGDLSSASAIM*GNAK	V
3816	2	38	B	0.13	4.47	0.589	0.589	gi|187369324|dbj|BAG31393.1|	73	R	YFDSFGDLSSASAIM*GNAK	V
3933	2	38	B	-1.8	5.51	0.545	0.545	gi|187369324|dbj|BAG31393.1|	73	R	YFDSFGDLSSASAIM*GNAK	V
3938	2	38	B	-1.9	5.09	0.585	0.585	gi|187369324|dbj|BAG31393.1|	73	R	YFDSFGDLSSASAIM*GNAK	V
3883	2	38	A	4.95	5.87	0.597	0.597	gi|187369324|dbj|BAG31393.1|	73	R	YFDSFGDLSSASAIMGNAK	V
3890	2	38	A	2.77	6.95	0.487	0.487	gi|187369324|dbj|BAG31393.1|	73	R	YFDSFGDLSSASAIMGNAK	V
3963	3	38	A	-0.8	3.23	0.304	0.304	gi|187369324|dbj|BAG31393.1|	73	R	YFDSFGDLSSASAIMGNAK	V
4028	2	38	A	-0.7	5.69	0.542	0.542	gi|187369324|dbj|BAG31393.1|	73	R	YFDSFGDLSSASAIMGNAK	V
4169	2	38	A	-0.5	4.89	0.631	0.631	gi|187369324|dbj|BAG31393.1|	73	R	YFDSFGDLSSASAIMGNAK	V
4457	2	38	A	0.27	5.91	0.554	0.554	gi|187369324|dbj|BAG31393.1|	73	R	YFDSFGDLSSASAIMGNAK	V
4467	2	38	A	0.24	4.27	0.607	0.607	gi|187369324|dbj|BAG31393.1|	73	R	YFDSFGDLSSASAIMGNAK	V
4620	2	38	A	-0.4	4.66	0.71	0.71	gi|187369324|dbj|BAG31393.1|	73	R	YFDSFGDLSSASAIMGNAK	V
4796	2	38	A	0.22	2.94	0.702	0.702	gi|187369324|dbj|BAG31393.1|	73	R	YFDSFGDLSSASAIMGNAK	V

Scan	z	Sample ID	Band	PPM	XCorr	Delta correlation	Unique delta correlation	Reference	Redundancy		Peptides	
4958	2	38	A	-0.2	2.52	0.615	0.615	gi|187369324|dbj|BAG31393.1|	73	R	YFDSFGDLSSASAIMGNAK	V
5366	2	38	A	-1.5	4.48	0.616	0.616	gi|187369324|dbj|BAG31393.1|	73	R	YFDSFGDLSSASAIMGNAK	V
5389	2	38	A	-0.8	3.24	0.648	0.648	gi|187369324|dbj|BAG31393.1|	73	R	YFDSFGDLSSASAIMGNAK	V
5514	2	38	A	0.45	4.03	0.651	0.651	gi|187369324|dbj|BAG31393.1|	73	R	YFDSFGDLSSASAIMGNAK	V
5517	2	38	A	0.35	3.93	0.697	0.697	gi|187369324|dbj|BAG31393.1|	73	R	YFDSFGDLSSASAIMGNAK	V
5636	2	38	A	0.82	4.09	0.641	0.641	gi|187369324|dbj|BAG31393.1|	73	R	YFDSFGDLSSASAIMGNAK	V
5639	2	38	A	-0.5	4.65	0.622	0.622	gi|187369324|dbj|BAG31393.1|	73	R	YFDSFGDLSSASAIMGNAK	V
5645	3	38	A	-0.3	3.33	0.302	0.302	gi|187369324|dbj|BAG31393.1|	73	R	YFDSFGDLSSASAIMGNAK	V
5763	2	38	A	0.76	3.71	0.588	0.588	gi|187369324|dbj|BAG31393.1|	73	R	YFDSFGDLSSASAIMGNAK	V
5766	2	38	A	0.66	5.49	0.58	0.58	gi|187369324|dbj|BAG31393.1|	73	R	YFDSFGDLSSASAIMGNAK	V
5771	3	38	A	0.13	3.18	0.29	0.29	gi|187369324|dbj|BAG31393.1|	73	R	YFDSFGDLSSASAIMGNAK	V
18	2	38	B	0.5	3.95	0.656	0.656	gi|187369324|dbj|BAG31393.1|	73	R	YFDSFGDLSSASAIMGNAK	V
25	2	38	B	0.58	5.49	0.578	0.578	gi|187369324|dbj|BAG31393.1|	73	R	YFDSFGDLSSASAIMGNAK	V
30	3	38	B	-0.3	3.89	0.312	0.312	gi|187369324|dbj|BAG31393.1|	73	R	YFDSFGDLSSASAIMGNAK	V
45	3	38	B	-0.5	3.73	0.309	0.309	gi|187369324|dbj|BAG31393.1|	73	R	YFDSFGDLSSASAIMGNAK	V
146	2	38	B	0.3	5.26	0.611	0.611	gi|187369324|dbj|BAG31393.1|	73	R	YFDSFGDLSSASAIMGNAK	V
151	2	38	B	0.32	5.54	0.594	0.594	gi|187369324|dbj|BAG31393.1|	73	R	YFDSFGDLSSASAIMGNAK	V
168	3	38	B	-0.9	4.39	0.361	0.361	gi|187369324|dbj|BAG31393.1|	73	R	YFDSFGDLSSASAIMGNAK	V
279	2	38	B	0.46	6.1	0.56	0.56	gi|187369324|dbj|BAG31393.1|	73	R	YFDSFGDLSSASAIMGNAK	V
290	2	38	B	0.55	4.11	0.477	0.477	gi|187369324|dbj|BAG31393.1|	73	R	YFDSFGDLSSASAIMGNAK	V
3919	2	38	B	2.71	5.8	0.548	0.548	gi|187369324|dbj|BAG31393.1|	73	R	YFDSFGDLSSASAIMGNAK	V
3930	2	38	B	2.69	4.03	0.677	0.677	gi|187369324|dbj|BAG31393.1|	73	R	YFDSFGDLSSASAIMGNAK	V
4011	3	38	B	-0.4	3.2	0.297	0.297	gi|187369324|dbj|BAG31393.1|	73	R	YFDSFGDLSSASAIMGNAK	V
4046	2	38	B	-0.9	4.22	0.614	0.614	gi|187369324|dbj|BAG31393.1|	73	R	YFDSFGDLSSASAIMGNAK	V
4240	2	38	B	1.07	3.44	0.661	0.661	gi|187369324|dbj|BAG31393.1|	73	R	YFDSFGDLSSASAIMGNAK	V
4251	2	38	B	0.83	3.91	0.61	0.61	gi|187369324|dbj|BAG31393.1|	73	R	YFDSFGDLSSASAIMGNAK	V
4442	2	38	B	0.3	3.29	0.705	0.705	gi|187369324|dbj|BAG31393.1|	73	R	YFDSFGDLSSASAIMGNAK	V
4469	2	38	B	0.04	4.57	0.68	0.68	gi|187369324|dbj|BAG31393.1|	73	R	YFDSFGDLSSASAIMGNAK	V
4483	2	38	B	0.15	4.66	0.574	0.574	gi|187369324|dbj|BAG31393.1|	73	R	YFDSFGDLSSASAIMGNAK	V
4678	2	38	B	-0.6	3.96	0.667	0.667	gi|187369324|dbj|BAG31393.1|	73	R	YFDSFGDLSSASAIMGNAK	V
4692	2	38	B	-1.2	3.87	0.608	0.608	gi|187369324|dbj|BAG31393.1|	73	R	YFDSFGDLSSASAIMGNAK	V
4830	2	38	B	-0.3	4.65	0.545	0.545	gi|187369324|dbj|BAG31393.1|	73	R	YFDSFGDLSSASAIMGNAK	V
4840	2	38	B	-0.4	4.28	0.621	0.621	gi|187369324|dbj|BAG31393.1|	73	R	YFDSFGDLSSASAIMGNAK	V
4974	2	38	B	1.76	3.76	0.704	0.704	gi|187369324|dbj|BAG31393.1|	73	R	YFDSFGDLSSASAIMGNAK	V
4980	2	38	B	1.5	4.25	0.637	0.637	gi|187369324|dbj|BAG31393.1|	73	R	YFDSFGDLSSASAIMGNAK	V
5137	2	38	B	0.53	3.92	0.573	0.573	gi|187369324|dbj|BAG31393.1|	73	R	YFDSFGDLSSASAIMGNAK	V
5275	2	38	B	-0.4	3.46	0.573	0.573	gi|187369324|dbj|BAG31393.1|	73	R	YFDSFGDLSSASAIMGNAK	V
5281	2	38	B	-0.9	3.8	0.679	0.679	gi|187369324|dbj|BAG31393.1|	73	R	YFDSFGDLSSASAIMGNAK	V

Scan	z	Sample ID	Band	PPM	XCorr	Delta correlation	Unique delta correlation	Reference	Redundancy		Peptides	
2531	2	40	A	0.33	3.36	0.894	0.894	gi|554577472|ref|XP_005880462.1|	74	K	AAVSGLWGK	V
2542	2	40	A	0.33	3.36	0.897	0.897	gi|554577472|ref|XP_005880462.1|	74	K	AAVSGLWGK	V
4118	2	40	A	4.97	2.61	0.879	0.879	gi|554577472|ref|XP_005880462.1|	74	K	AAVSGLWGK	V
2394	2	40	B	-0.1	3.28	0.894	0.894	gi|554577472|ref|XP_005880462.1|	74	K	AAVSGLWGK	V
2404	2	40	B	-0.1	3.1	0.887	0.887	gi|554577472|ref|XP_005880462.1|	74	K	AAVSGLWGK	V
2519	2	40	A	1.9	3.38	0.914	0.914	gi|532054982|ref|XP_005370208.1|	94	K	DFTPAAQAAFQK	V
2532	2	40	A	1.75	2.91	0.9	0.9	gi|532054982|ref|XP_005370208.1|	94	K	DFTPAAQAAFQK	V
4373	2	40	A	-0.1	2.62	0.993	0.993	gi|532054982|ref|XP_005370208.1|	94	K	DFTPAAQAAFQK	V
2381	2	40	B	1.77	3.87	0.967	0.967	gi|532054982|ref|XP_005370208.1|	94	K	DFTPAAQAAFQK	V
2713	2	40	A	3.02	3.59	0	0.284	gi|27574244|pdb|1O1N|A	0	K	FLASVSTVLTSK	Y
2716	2	40	A	3.26	3.44	0	0.311	gi|27574244|pdb|1O1N|A	0	K	FLASVSTVLTSK	Y
2968	2	40	A	1.46	2.53	0	0.469	gi|27574244|pdb|1O1N|A	0	K	FLASVSTVLTSK	Y
2972	2	40	A	1.38	3.03	0	0.39	gi|27574244|pdb|1O1N|A	0	K	FLASVSTVLTSK	Y
3136	2	40	A	1.65	2.71	0	0.459	gi|27574244|pdb|1O1N|A	0	K	FLASVSTVLTSK	Y
3280	2	40	A	1.91	2.68	0	0.418	gi|27574244|pdb|1O1N|A	0	K	FLASVSTVLTSK	Y
3288	2	40	A	2.12	3.17	0	0.375	gi|27574244|pdb|1O1N|A	0	K	FLASVSTVLTSK	Y
3567	2	40	A	3.12	2.83	0	0.39	gi|27574244|pdb|1O1N|A	0	K	FLASVSTVLTSK	Y
3570	2	40	A	2.91	3.02	0	0.375	gi|27574244|pdb|1O1N|A	0	K	FLASVSTVLTSK	Y
4076	2	40	A	4.89	2.93	0	0.365	gi|27574244|pdb|1O1N|A	0	K	FLASVSTVLTSK	Y
4196	2	40	A	-0	2.89	0	0.352	gi|27574244|pdb|1O1N|A	0	K	FLASVSTVLTSK	Y
4208	2	40	A	0.49	2.94	0	0.387	gi|27574244|pdb|1O1N|A	0	K	FLASVSTVLTSK	Y
4430	2	40	A	0.34	2.75	0	0.359	gi|27574244|pdb|1O1N|A	0	K	FLASVSTVLTSK	Y
2567	2	40	B	1.33	3.32	0	0.406	gi|27574244|pdb|1O1N|A	0	K	FLASVSTVLTSK	Y
2577	2	40	B	1.29	3.25	0	0.41	gi|27574244|pdb|1O1N|A	0	K	FLASVSTVLTSK	Y
2838	2	40	B	-0.3	2.68	0	0.41	gi|27574244|pdb|1O1N|A	0	K	FLASVSTVLTSK	Y
3034	2	40	B	0.62	2.5	0	0.376	gi|27574244|pdb|1O1N|A	0	K	FLASVSTVLTSK	Y
3048	2	40	B	0.62	2.66	0	0.421	gi|27574244|pdb|1O1N|A	0	K	FLASVSTVLTSK	Y
3866	2	40	B	4.85	3.04	0	0.361	gi|27574244|pdb|1O1N|A	0	K	FLASVSTVLTSK	Y
3870	2	40	B	4.74	3.12	0	0.35	gi|27574244|pdb|1O1N|A	0	K	FLASVSTVLTSK	Y
3991	2	40	B	4.06	2.83	0	0.378	gi|27574244|pdb|1O1N|A	0	K	FLASVSTVLTSK	Y
4106	2	40	B	-0.2	2.61	0	0.44	gi|27574244|pdb|1O1N|A	0	K	FLASVSTVLTSK	Y
2525	2	40	A	0.66	3.24	0.137	0.137	gi|534467043|gb|AGU26656.1|	99	K	GTFASLSELHC#DK	L
2459	2	40	A	-0.1	2.98	0.127	0.127	gi|528765669|gb|EPY85328.1|	8	K	IGGHAAEYGAEALER	M
2316	2	40	B	0.2	2.85	0.231	0.231	gi|528765669|gb|EPY85328.1|	8	K	IGGHAAEYGAEALER	M
2412	2	40	A	3.83	3.6	0.126	0.126	gi|344254270|gb|EGW10374.1|	99	K	IGGHGAEYGAEALER	M
2267	2	40	B	1.93	3.87	0.117	0.117	gi|344254270|gb|EGW10374.1|	99	K	IGGHGAEYGAEALER	M
2596	2	40	A	1.95	3.39	0.632	0.632	gi|122513|sp|P02088.2|HBB1_MOUSE	65	K	KVITAFNDGLNHLDSLK	G
2599	3	40	A	2.5	3.89	0.688	0.688	gi|122513|sp|P02088.2|HBB1_MOUSE	65	K	KVITAFNDGLNHLDSLK	G

Scan	z	Sample ID	Band	PPM	XCorr	Delta correlation	Unique delta correlation	Reference	Redundancy		Peptides	
2942	2	40	A	3.16	3.56	0.213	0.213	gi|122513|sp|P02088.2|HBB1_MOUSE	88	R	LLGNMIVIVLGHHLGK	D
2953	2	40	A	3.1	3.94	0.189	0.189	gi|122513|sp|P02088.2|HBB1_MOUSE	88	R	LLGNMIVIVLGHHLGK	D
2819	2	40	B	1.67	3.8	0.202	0.202	gi|122513|sp|P02088.2|HBB1_MOUSE	88	R	LLGNMIVIVLGHHLGK	D
2830	2	40	B	1.58	4.37	0.209	0.209	gi|122513|sp|P02088.2|HBB1_MOUSE	88	R	LLGNMIVIVLGHHLGK	D
3892	2	40	A	4.8	2.71	0.947	0.947	gi|431903465|gb|ELK09417.1|	99	R	LLVVYPWTQR	F
2357	2	40	B	0.77	2.79	0.474	0.474	gi|27574244|pdb|1O1N|A	99	K	LRVDPVNFK	L
2492	2	40	A	2.08	2.75	0.329	0.329	gi|12833511|dbj|BAB22552.1|	31	R	M*FASFPTTK	T
2349	2	40	B	1.02	2.79	0.324	0.324	gi|12833511|dbj|BAB22552.1|	31	R	M*FASFPTTK	T
2356	2	40	B	1.02	2.52	0.3	0.3	gi|12833511|dbj|BAB22552.1|	31	R	M*FASFPTTK	T
2304	2	40	A	-0.2	2.9	0.334	0.334	gi|12833511|dbj|BAB22552.1|	27	-	M*VLSGEDKSNIK	A
2530	2	40	A	0.98	4.89	0.107	0.107	gi|344254270|gb|EGW10374.1|	99	K	TYFPHFDVSHGSAQVK	A
2541	2	40	A	2.21	5.01	0.106	0.106	gi|344254270|gb|EGW10374.1|	99	K	TYFPHFDVSHGSAQVK	A
2543	3	40	A	1.12	3.35	0.118	0.118	gi|344254270|gb|EGW10374.1|	99	K	TYFPHFDVSHGSAQVK	A
2392	2	40	B	1.3	3.93	0.116	0.116	gi|344254270|gb|EGW10374.1|	99	K	TYFPHFDVSHGSAQVK	A
2393	3	40	B	0.55	3.26	0.145	0.145	gi|344254270|gb|EGW10374.1|	99	K	TYFPHFDVSHGSAQVK	A
2403	2	40	B	1.29	4.59	0.116	0.116	gi|344254270|gb|EGW10374.1|	99	K	TYFPHFDVSHGSAQVK	A
2405	3	40	B	0.55	3.9	0.102	0.102	gi|344254270|gb|EGW10374.1|	99	K	TYFPHFDVSHGSAQVK	A
2943	3	40	A	3.45	7.72	0.903	0.903	gi|159137400|gb|ABW88847.1|	2	K	VADALATAAGHLDDLPGALSALSDLHAHK	L
2954	3	40	A	3.43	9.06	0.907	0.907	gi|159137400|gb|ABW88847.1|	2	K	VADALATAAGHLDDLPGALSALSDLHAHK	L
2866	3	40	B	1.65	8.71	0.931	0.931	gi|159137400|gb|ABW88847.1|	2	K	VADALATAAGHLDDLPGALSALSDLHAHK	L
2877	3	40	B	1.67	7.87	0.933	0.933	gi|159137400|gb|ABW88847.1|	2	K	VADALATAAGHLDDLPGALSALSDLHAHK	L
2135	2	40	B	2.06	2.75	0.2	0.2	gi|378548366|sp|B3EWD6.1|HBB_PERCA	20	-	VHLTDAEK	A
2691	2	40	A	4.04	2.57	0.856	0.856	gi|122513|sp|P02088.2|HBB1_MOUSE	65	K	VITAFNDGLNHLDSLK	G
4106	2	40	A	3.92	2.72	0.789	0.789	gi|122513|sp|P02088.2|HBB1_MOUSE	65	K	VITAFNDGLNHLDSLK	G
4355	2	40	A	0.02	2.93	0.726	0.726	gi|122513|sp|P02088.2|HBB1_MOUSE	65	K	VITAFNDGLNHLDSLK	G
2244	2	40	A	1.22	3.61	0.528	0.528	gi|241913510|pdb|3HRW|C	2	-	VLSGEDKSNIK	A
2255	2	40	A	0.95	3.4	0.596	0.596	gi|241913510|pdb|3HRW|C	2	-	VLSGEDKSNIK	A
2088	2	40	B	3.08	3.63	0.521	0.521	gi|241913510|pdb|3HRW|C	2	-	VLSGEDKSNIK	A
2094	2	40	B	3.03	3.6	0.514	0.514	gi|241913510|pdb|3HRW|C	2	-	VLSGEDKSNIK	A
3600	2	40	A	3.42	2.86	0	0.192	gi|378548370|sp|B3EWD8.1|HBB_TAMHU	0	K	NVADEVGGEALGR^	L
3606	2	40	A	2.94	2.92	0	0.183	gi|378548370|sp|B3EWD8.1|HBB_TAMHU	0	K	NVADEVGGEALGR^	L
3768	2	40	A	2.64	3.23	0	0.213	gi|378548370|sp|B3EWD8.1|HBB_TAMHU	0	K	NVADEVGGEALGR^	L
4264	2	40	A	-0.4	3.52	0	0.28	gi|378548370|sp|B3EWD8.1|HBB_TAMHU	0	K	NVADEVGGEALGR^	L
4369	2	40	A	-0.2	4.85	0	0.305	gi|378548370|sp|B3EWD8.1|HBB_TAMHU	0	K	NVADEVGGEALGR^	L
4484	2	40	A	0.09	3.61	0	0.283	gi|378548370|sp|B3EWD8.1|HBB_TAMHU	0	K	NVADEVGGEALGR^	L
4586	2	40	A	-0.5	2.59	0	0.221	gi|378548370|sp|B3EWD8.1|HBB_TAMHU	0	K	NVADEVGGEALGR^	L
2256	2	40	B	1.87	3.75	0	0.344	gi|378548370|sp|B3EWD8.1|HBB_TAMHU	0	K	NVADEVGGEALGR^	L
2428	2	40	B	-0.9	2.75	0	0.113	gi|378548370|sp|B3EWD8.1|HBB_TAMHU	0	K	NVADEVGGEALGR^	L

Scan	z	Sample ID	Band	PPM	XCorr	Delta correlation	Unique delta correlation	Reference	Redundancy		Peptides	
3468	2	40	B	1.06	2.87	0	0.215	gi|378548370|sp|B3EWD8.1|HBB_TAMHU	0	K	NVADEVGGEALGR^	L
3472	2	40	B	1.01	2.59	0	0.129	gi|378548370|sp|B3EWD8.1|HBB_TAMHU	0	K	NVADEVGGEALGR^	L
3603	2	40	B	4.51	4.29	0	0.256	gi|378548370|sp|B3EWD8.1|HBB_TAMHU	0	K	NVADEVGGEALGR^	L
3857	2	40	B	4.74	4.45	0	0.303	gi|378548370|sp|B3EWD8.1|HBB_TAMHU	0	K	NVADEVGGEALGR^	L
4105	2	40	B	-0.4	3.03	0	0.164	gi|378548370|sp|B3EWD8.1|HBB_TAMHU	0	K	NVADEVGGEALGR^	L
4231	2	40	B	0.22	3.98	0	0.288	gi|378548370|sp|B3EWD8.1|HBB_TAMHU	0	K	NVADEVGGEALGR^	L
4340	2	40	B	-0.1	3.06	0	0.187	gi|378548370|sp|B3EWD8.1|HBB_TAMHU	0	K	NVADEVGGEALGR^	L
2479	2	40	A	0.15	3.33	0.105	0.105	gi|667267074|ref|XP_008569536.1|	4	K	VNVDDVGGEALGR	L
2437	2	40	A	1.4	3.99	0.876	0.876	gi|12847007|dbj|BAB27399.1|	60	K	VVAGVAAALAHK	Y
2445	2	40	A	1.38	3.93	0.877	0.877	gi|12847007|dbj|BAB27399.1|	60	K	VVAGVAAALAHK	Y
2559	2	40	A	0.25	3.96	0.841	0.841	gi|12847007|dbj|BAB27399.1|	60	K	VVAGVAAALAHK	Y
2571	2	40	A	0.45	3.83	0.885	0.885	gi|12847007|dbj|BAB27399.1|	60	K	VVAGVAAALAHK	Y
3676	2	40	A	0.65	3.44	0.943	0.943	gi|12847007|dbj|BAB27399.1|	60	K	VVAGVAAALAHK	Y
3740	2	40	A	3.98	2.97	0.942	0.942	gi|12847007|dbj|BAB27399.1|	60	K	VVAGVAAALAHK	Y
3806	2	40	A	1.14	3.04	0.952	0.952	gi|12847007|dbj|BAB27399.1|	60	K	VVAGVAAALAHK	Y
3862	2	40	A	4.43	3.05	0.931	0.931	gi|12847007|dbj|BAB27399.1|	60	K	VVAGVAAALAHK	Y
3864	2	40	A	4.47	2.84	0.948	0.948	gi|12847007|dbj|BAB27399.1|	60	K	VVAGVAAALAHK	Y
3987	2	40	A	4.92	3.3	0.943	0.943	gi|12847007|dbj|BAB27399.1|	60	K	VVAGVAAALAHK	Y
4116	2	40	A	4.4	3.06	0.946	0.946	gi|12847007|dbj|BAB27399.1|	60	K	VVAGVAAALAHK	Y
4120	2	40	A	4.48	3.04	0.945	0.945	gi|12847007|dbj|BAB27399.1|	60	K	VVAGVAAALAHK	Y
4364	2	40	A	-0.2	3.38	0.948	0.948	gi|12847007|dbj|BAB27399.1|	60	K	VVAGVAAALAHK	Y
2306	2	40	B	0.67	4.11	0.883	0.883	gi|12847007|dbj|BAB27399.1|	60	K	VVAGVAAALAHK	Y
2314	2	40	B	0.63	3.99	0.872	0.872	gi|12847007|dbj|BAB27399.1|	60	K	VVAGVAAALAHK	Y
2450	2	40	B	-1.5	3.71	0.91	0.91	gi|12847007|dbj|BAB27399.1|	60	K	VVAGVAAALAHK	Y
2458	2	40	B	-1.4	3.49	0.893	0.893	gi|12847007|dbj|BAB27399.1|	60	K	VVAGVAAALAHK	Y
3727	2	40	B	3.46	2.71	0.955	0.955	gi|12847007|dbj|BAB27399.1|	60	K	VVAGVAAALAHK	Y
2482	2	40	A	-2.2	4.92	0.909	0.909	gi|12847007|dbj|BAB27399.1|	60	K	VVAGVAAALAHKYH	-
2490	2	40	A	2.52	4.22	0.915	0.915	gi|12847007|dbj|BAB27399.1|	60	K	VVAGVAAALAHKYH	-
2612	2	40	A	1	3.35	0.916	0.916	gi|12847007|dbj|BAB27399.1|	60	K	VVAGVAAALAHKYH	-
2336	2	40	B	1.56	4.58	0.889	0.889	gi|12847007|dbj|BAB27399.1|	60	K	VVAGVAAALAHKYH	-
2345	2	40	B	1.56	3.65	0.91	0.91	gi|12847007|dbj|BAB27399.1|	60	K	VVAGVAAALAHKYH	-
2365	2	40	A	0.89	3.08	0.894	0.894	gi|591333109|ref|XP_007092291.1|	23	K	VVAGVASALAHK	Y
2753	2	40	A	4.37	5.68	0.482	0.482	gi|187369324|dbj|BAG31393.1|	73	R	YFDSFGDLSSASAIM*GNAK	V
2764	2	40	A	4.36	6.47	0.423	0.423	gi|187369324|dbj|BAG31393.1|	73	R	YFDSFGDLSSASAIM*GNAK	V
2766	3	40	A	1.98	6.35	0.475	0.475	gi|187369324|dbj|BAG31393.1|	73	R	YFDSFGDLSSASAIM*GNAK	V
2776	3	40	A	1.99	7.3	0.405	0.405	gi|187369324|dbj|BAG31393.1|	73	R	YFDSFGDLSSASAIM*GNAK	V
3016	2	40	A	0.75	4.74	0.53	0.53	gi|187369324|dbj|BAG31393.1|	73	R	YFDSFGDLSSASAIM*GNAK	V
3028	2	40	A	0.64	3.13	0.571	0.571	gi|187369324|dbj|BAG31393.1|	73	R	YFDSFGDLSSASAIM*GNAK	V

Scan	z	Sample ID	Band	PPM	XCorr	Delta correlation	Unique delta correlation	Reference	Redundancy		Peptides	
3213	2	40	A	2.83	2.8	0.508	0.508	gi|187369324|dbj|BAG31393.1|	73	R	YFDSFGDLSSASAIM*GNAK	V
3643	2	40	A	3.26	2.75	0.587	0.587	gi|187369324|dbj|BAG31393.1|	73	R	YFDSFGDLSSASAIM*GNAK	V
3709	2	40	A	4.94	3.03	0.569	0.569	gi|187369324|dbj|BAG31393.1|	73	R	YFDSFGDLSSASAIM*GNAK	V
3973	3	40	A	4.98	3.18	0.365	0.365	gi|187369324|dbj|BAG31393.1|	73	R	YFDSFGDLSSASAIM*GNAK	V
3996	3	40	A	4.66	3.71	0.355	0.355	gi|187369324|dbj|BAG31393.1|	73	R	YFDSFGDLSSASAIM*GNAK	V
4103	2	40	A	3.63	4.68	0.539	0.539	gi|187369324|dbj|BAG31393.1|	73	R	YFDSFGDLSSASAIM*GNAK	V
4255	2	40	A	0.06	3.03	0.672	0.672	gi|187369324|dbj|BAG31393.1|	73	R	YFDSFGDLSSASAIM*GNAK	V
4352	3	40	A	-0.6	4.16	0.498	0.498	gi|187369324|dbj|BAG31393.1|	73	R	YFDSFGDLSSASAIM*GNAK	V
4358	2	40	A	0.05	4.96	0.577	0.577	gi|187369324|dbj|BAG31393.1|	73	R	YFDSFGDLSSASAIM*GNAK	V
4361	3	40	A	-0.7	3.68	0.451	0.451	gi|187369324|dbj|BAG31393.1|	73	R	YFDSFGDLSSASAIM*GNAK	V
4391	2	40	A	0.08	3	0.693	0.693	gi|187369324|dbj|BAG31393.1|	73	R	YFDSFGDLSSASAIM*GNAK	V
4396	2	40	A	0.13	3.3	0.596	0.596	gi|187369324|dbj|BAG31393.1|	73	R	YFDSFGDLSSASAIM*GNAK	V
2608	2	40	B	2.88	7.15	0.447	0.447	gi|187369324|dbj|BAG31393.1|	73	R	YFDSFGDLSSASAIM*GNAK	V
2612	3	40	B	0.27	6.91	0.446	0.446	gi|187369324|dbj|BAG31393.1|	73	R	YFDSFGDLSSASAIM*GNAK	V
2619	2	40	B	2.86	6.65	0.424	0.424	gi|187369324|dbj|BAG31393.1|	73	R	YFDSFGDLSSASAIM*GNAK	V
2621	3	40	B	-0.2	7.07	0.427	0.427	gi|187369324|dbj|BAG31393.1|	73	R	YFDSFGDLSSASAIM*GNAK	V
2894	2	40	B	0.19	3.14	0.532	0.532	gi|187369324|dbj|BAG31393.1|	73	R	YFDSFGDLSSASAIM*GNAK	V
2920	2	40	B	0.12	2.54	0.553	0.553	gi|187369324|dbj|BAG31393.1|	73	R	YFDSFGDLSSASAIM*GNAK	V
3065	2	40	B	0.6	3.2	0.586	0.586	gi|187369324|dbj|BAG31393.1|	73	R	YFDSFGDLSSASAIM*GNAK	V
3075	2	40	B	1.42	3.25	0.613	0.613	gi|187369324|dbj|BAG31393.1|	73	R	YFDSFGDLSSASAIM*GNAK	V
3369	2	40	B	1.55	2.59	0.584	0.584	gi|187369324|dbj|BAG31393.1|	73	R	YFDSFGDLSSASAIM*GNAK	V
3453	2	40	B	2.02	2.92	0.595	0.595	gi|187369324|dbj|BAG31393.1|	73	R	YFDSFGDLSSASAIM*GNAK	V
3576	3	40	B	4.73	3.63	0.427	0.427	gi|187369324|dbj|BAG31393.1|	73	R	YFDSFGDLSSASAIM*GNAK	V
3769	3	40	B	4.34	3.86	0.335	0.335	gi|187369324|dbj|BAG31393.1|	73	R	YFDSFGDLSSASAIM*GNAK	V
3901	3	40	B	4.08	3.95	0.45	0.45	gi|187369324|dbj|BAG31393.1|	73	R	YFDSFGDLSSASAIM*GNAK	V
3985	2	40	B	4.12	5.1	0.564	0.564	gi|187369324|dbj|BAG31393.1|	73	R	YFDSFGDLSSASAIM*GNAK	V
3993	2	40	B	3.31	4.53	0.529	0.529	gi|187369324|dbj|BAG31393.1|	73	R	YFDSFGDLSSASAIM*GNAK	V
4036	3	40	B	0.35	4.12	0.456	0.456	gi|187369324|dbj|BAG31393.1|	73	R	YFDSFGDLSSASAIM*GNAK	V
4045	3	40	B	-0.1	3.74	0.362	0.362	gi|187369324|dbj|BAG31393.1|	73	R	YFDSFGDLSSASAIM*GNAK	V
4103	2	40	B	0.02	3.51	0.655	0.655	gi|187369324|dbj|BAG31393.1|	73	R	YFDSFGDLSSASAIM*GNAK	V
4109	2	40	B	0.05	3.84	0.715	0.715	gi|187369324|dbj|BAG31393.1|	73	R	YFDSFGDLSSASAIM*GNAK	V
4206	3	40	B	-0.2	3.54	0.435	0.435	gi|187369324|dbj|BAG31393.1|	73	R	YFDSFGDLSSASAIM*GNAK	V
4224	3	40	B	-0.2	3.84	0.347	0.347	gi|187369324|dbj|BAG31393.1|	73	R	YFDSFGDLSSASAIM*GNAK	V
4244	2	40	B	0.1	3.41	0.603	0.603	gi|187369324|dbj|BAG31393.1|	73	R	YFDSFGDLSSASAIM*GNAK	V
4249	2	40	B	0.45	3.5	0.667	0.667	gi|187369324|dbj|BAG31393.1|	73	R	YFDSFGDLSSASAIM*GNAK	V
2863	2	40	B	-0.1	2.87	0.925	0.925	gi|261873669|gb|ACY03366.1|	25	R	YFDSFGDLSSASAIM*GNPK	V
2900	3	40	A	1.52	6.25	0.383	0.383	gi|187369324|dbj|BAG31393.1|	73	R	YFDSFGDLSSASAIMGNAK	V
2908	3	40	A	1.52	5.9	0.342	0.342	gi|187369324|dbj|BAG31393.1|	73	R	YFDSFGDLSSASAIMGNAK	V

Scan	z	Sample ID	Band	PPM	XCorr	Delta correlation	Unique delta correlation	Reference	Redundancy		Peptides	
3054	2	40	A	1.91	4.6	0.442	0.442	gi|187369324|dbj|BAG31393.1|	73	R	YFDSFGDLSSASAIMGNAK	V
3187	2	40	A	2.23	3.88	0.521	0.521	gi|187369324|dbj|BAG31393.1|	73	R	YFDSFGDLSSASAIMGNAK	V
3209	2	40	A	2.72	2.83	0.622	0.622	gi|187369324|dbj|BAG31393.1|	73	R	YFDSFGDLSSASAIMGNAK	V
3350	2	40	A	1.79	2.98	0.534	0.534	gi|187369324|dbj|BAG31393.1|	73	R	YFDSFGDLSSASAIMGNAK	V
3642	2	40	A	2.44	3.12	0.582	0.582	gi|187369324|dbj|BAG31393.1|	73	R	YFDSFGDLSSASAIMGNAK	V
3650	2	40	A	2.61	3.27	0.633	0.633	gi|187369324|dbj|BAG31393.1|	73	R	YFDSFGDLSSASAIMGNAK	V
3770	2	40	A	2.98	4.47	0.631	0.631	gi|187369324|dbj|BAG31393.1|	73	R	YFDSFGDLSSASAIMGNAK	V
3786	2	40	A	2.72	4.13	0.632	0.632	gi|187369324|dbj|BAG31393.1|	73	R	YFDSFGDLSSASAIMGNAK	V
4272	2	40	A	-0.4	4.13	0.709	0.709	gi|187369324|dbj|BAG31393.1|	73	R	YFDSFGDLSSASAIMGNAK	V
4281	2	40	A	-0.4	4.48	0.655	0.655	gi|187369324|dbj|BAG31393.1|	73	R	YFDSFGDLSSASAIMGNAK	V
4397	2	40	A	0.61	3.33	0.719	0.719	gi|187369324|dbj|BAG31393.1|	73	R	YFDSFGDLSSASAIMGNAK	V
4403	2	40	A	0.57	3.78	0.625	0.625	gi|187369324|dbj|BAG31393.1|	73	R	YFDSFGDLSSASAIMGNAK	V
2757	2	40	B	2.59	6.6	0.471	0.471	gi|187369324|dbj|BAG31393.1|	73	R	YFDSFGDLSSASAIMGNAK	V
2760	3	40	B	-0.5	6.13	0.386	0.386	gi|187369324|dbj|BAG31393.1|	73	R	YFDSFGDLSSASAIMGNAK	V
2764	2	40	B	2.52	5.84	0.512	0.512	gi|187369324|dbj|BAG31393.1|	73	R	YFDSFGDLSSASAIMGNAK	V
2766	3	40	B	-0.5	6.51	0.363	0.363	gi|187369324|dbj|BAG31393.1|	73	R	YFDSFGDLSSASAIMGNAK	V
2893	2	40	B	0.27	4.13	0.531	0.531	gi|187369324|dbj|BAG31393.1|	73	R	YFDSFGDLSSASAIMGNAK	V
3073	2	40	B	1.13	2.8	0.451	0.451	gi|187369324|dbj|BAG31393.1|	73	R	YFDSFGDLSSASAIMGNAK	V
3568	2	40	B	4.84	4.22	0.599	0.599	gi|187369324|dbj|BAG31393.1|	73	R	YFDSFGDLSSASAIMGNAK	V
4092	2	40	B	0.18	4.44	0.607	0.607	gi|187369324|dbj|BAG31393.1|	73	R	YFDSFGDLSSASAIMGNAK	V
4102	2	40	B	0.08	3.16	0.655	0.655	gi|187369324|dbj|BAG31393.1|	73	R	YFDSFGDLSSASAIMGNAK	V
4238	2	40	B	2.5	3.78	0.652	0.652	gi|187369324|dbj|BAG31393.1|	73	R	YFDSFGDLSSASAIMGNAK	V
4250	2	40	B	0.52	4.24	0.708	0.708	gi|187369324|dbj|BAG31393.1|	73	R	YFDSFGDLSSASAIMGNAK	V
2623	2	41	A	3.28	3.17	0.922	0.922	gi|554577472|ref|XP_005880462.1|	74	K	AAVSGLWGK	V
2628	2	41	A	3.28	3.16	0.919	0.919	gi|554577472|ref|XP_005880462.1|	74	K	AAVSGLWGK	V
4073	2	41	A	3.2	2.52	0.97	0.97	gi|554577472|ref|XP_005880462.1|	74	K	AAVSGLWGK	V
4212	2	41	A	-0.8	2.69	0.996	0.996	gi|554577472|ref|XP_005880462.1|	74	K	AAVSGLWGK	V
4222	2	41	A	-0.8	2.81	0.987	0.987	gi|554577472|ref|XP_005880462.1|	74	K	AAVSGLWGK	V
4350	2	41	A	-0.5	2.7	0.984	0.984	gi|554577472|ref|XP_005880462.1|	74	K	AAVSGLWGK	V
4474	2	41	A	-0	2.7	0.986	0.986	gi|554577472|ref|XP_005880462.1|	74	K	AAVSGLWGK	V
4591	2	41	A	-0.4	2.53	0.955	0.955	gi|554577472|ref|XP_005880462.1|	74	K	AAVSGLWGK	V
4697	2	41	A	-0.2	2.78	0.971	0.971	gi|554577472|ref|XP_005880462.1|	74	K	AAVSGLWGK	V
4818	2	41	A	-0.5	2.77	0.982	0.982	gi|554577472|ref|XP_005880462.1|	74	K	AAVSGLWGK	V
4821	2	41	A	-0.6	2.78	0.978	0.978	gi|554577472|ref|XP_005880462.1|	74	K	AAVSGLWGK	V
2385	2	41	B	3.68	3.25	0.924	0.924	gi|554577472|ref|XP_005880462.1|	74	K	AAVSGLWGK	V
2394	2	41	B	3.68	2.93	0.934	0.934	gi|554577472|ref|XP_005880462.1|	74	K	AAVSGLWGK	V
3759	2	41	B	3.12	2.69	0.986	0.986	gi|554577472|ref|XP_005880462.1|	74	K	AAVSGLWGK	V
3907	2	41	B	0.12	2.81	0.984	0.984	gi|554577472|ref|XP_005880462.1|	74	K	AAVSGLWGK	V

Scan	z	Sample ID	Band	PPM	XCorr	Delta correlation	Unique delta correlation	Reference	Redundancy		Peptides	
3920	2	41	B	0.02	2.73	0.986	0.986	gi|554577472|ref|XP_005880462.1|	74	K	AAVSGLWGK	V
4042	2	41	B	-0.3	2.82	0.939	0.939	gi|554577472|ref|XP_005880462.1|	74	K	AAVSGLWGK	V
4049	2	41	B	-0.4	2.7	0.983	0.983	gi|554577472|ref|XP_005880462.1|	74	K	AAVSGLWGK	V
4169	2	41	B	-0.3	2.76	0.982	0.982	gi|554577472|ref|XP_005880462.1|	74	K	AAVSGLWGK	V
4172	2	41	B	-0.4	2.73	0.985	0.985	gi|554577472|ref|XP_005880462.1|	74	K	AAVSGLWGK	V
4402	2	41	B	-0.2	2.53	0.984	0.984	gi|554577472|ref|XP_005880462.1|	74	K	AAVSGLWGK	V
4414	2	41	B	-0.2	2.77	0.985	0.985	gi|554577472|ref|XP_005880462.1|	74	K	AAVSGLWGK	V
4525	2	41	B	-0.1	2.76	0.982	0.982	gi|554577472|ref|XP_005880462.1|	74	K	AAVSGLWGK	V
4534	2	41	B	-0.2	2.55	0.95	0.95	gi|554577472|ref|XP_005880462.1|	74	K	AAVSGLWGK	V
4650	2	41	B	-0.3	2.64	0.981	0.981	gi|554577472|ref|XP_005880462.1|	74	K	AAVSGLWGK	V
2653	2	41	A	2.86	2.63	0.991	0.991	gi|532054982|ref|XP_005370208.1|	94	K	DFTPAAQAAFQK	V
2771	2	41	A	3.24	2.87	0.9	0.9	gi|532054982|ref|XP_005370208.1|	94	K	DFTPAAQAAFQK	V
2774	2	41	A	3.34	3.09	0.883	0.883	gi|532054982|ref|XP_005370208.1|	94	K	DFTPAAQAAFQK	V
4582	2	41	A	-0.5	2.61	0.95	0.95	gi|532054982|ref|XP_005370208.1|	94	K	DFTPAAQAAFQK	V
4637	2	41	A	-0.5	2.7	0.956	0.956	gi|532054982|ref|XP_005370208.1|	94	K	DFTPAAQAAFQK	V
2434	2	41	B	4.25	3.99	0.954	0.954	gi|532054982|ref|XP_005370208.1|	94	K	DFTPAAQAAFQK	V
2443	2	41	B	4.23	3.43	0.939	0.939	gi|532054982|ref|XP_005370208.1|	94	K	DFTPAAQAAFQK	V
2564	2	41	B	2.56	2.93	0.899	0.899	gi|532054982|ref|XP_005370208.1|	94	K	DFTPAAQAAFQK	V
4007	2	41	B	0.75	2.62	0.939	0.939	gi|532054982|ref|XP_005370208.1|	94	K	DFTPAAQAAFQK	V
4356	2	41	B	-0.4	2.57	0.95	0.95	gi|532054982|ref|XP_005370208.1|	94	K	DFTPAAQAAFQK	V
2853	2	41	A	3.79	2.99	0	0.306	gi|27574244|pdb|1O1N|A	0	K	FLASVSTVLTSK	Y
2864	2	41	A	3.79	3.1	0	0.29	gi|27574244|pdb|1O1N|A	0	K	FLASVSTVLTSK	Y
4000	2	41	A	3.29	3.18	0	0.327	gi|27574244|pdb|1O1N|A	0	K	FLASVSTVLTSK	Y
4002	2	41	A	4.32	2.57	0	0.295	gi|27574244|pdb|1O1N|A	0	K	FLASVSTVLTSK	Y
4134	2	41	A	-0.2	2.54	0	0.313	gi|27574244|pdb|1O1N|A	0	K	FLASVSTVLTSK	Y
4136	2	41	A	-0.2	2.79	0	0.375	gi|27574244|pdb|1O1N|A	0	K	FLASVSTVLTSK	Y
4263	2	41	A	-0.4	3.05	0	0.372	gi|27574244|pdb|1O1N|A	0	K	FLASVSTVLTSK	Y
4265	2	41	A	-0.3	2.85	0	0.367	gi|27574244|pdb|1O1N|A	0	K	FLASVSTVLTSK	Y
4517	2	41	A	0	2.5	0	0.373	gi|27574244|pdb|1O1N|A	0	K	FLASVSTVLTSK	Y
4866	2	41	A	-0.2	2.62	0	0.344	gi|27574244|pdb|1O1N|A	0	K	FLASVSTVLTSK	Y
2640	2	41	B	3.75	3.44	0	0.319	gi|27574244|pdb|1O1N|A	0	K	FLASVSTVLTSK	Y
2650	2	41	B	3.75	3.23	0	0.325	gi|27574244|pdb|1O1N|A	0	K	FLASVSTVLTSK	Y
3173	2	41	B	4.26	2.63	0	0.295	gi|27574244|pdb|1O1N|A	0	K	FLASVSTVLTSK	Y
3720	2	41	B	3.81	2.64	0	0.312	gi|27574244|pdb|1O1N|A	0	K	FLASVSTVLTSK	Y
3722	2	41	B	3.48	3.17	0	0.333	gi|27574244|pdb|1O1N|A	0	K	FLASVSTVLTSK	Y
3849	2	41	B	0.53	2.9	0	0.357	gi|27574244|pdb|1O1N|A	0	K	FLASVSTVLTSK	Y
3977	2	41	B	-0.1	2.71	0	0.387	gi|27574244|pdb|1O1N|A	0	K	FLASVSTVLTSK	Y
3979	2	41	B	-0.1	3.03	0	0.348	gi|27574244|pdb|1O1N|A	0	K	FLASVSTVLTSK	Y

Scan	z	Sample ID	Band	PPM	XCorr	Delta correlation	Unique delta correlation	Reference	Redundancy		Peptides	
4101	2	41	B	-0.4	2.68	0	0.293	gi|27574244|pdb|1O1N|A	0	K	FLASVSTVLTSK	Y
4104	2	41	B	-0.5	3.04	0	0.344	gi|27574244|pdb|1O1N|A	0	K	FLASVSTVLTSK	Y
4226	2	41	B	-0.5	2.71	0	0.372	gi|27574244|pdb|1O1N|A	0	K	FLASVSTVLTSK	Y
4230	2	41	B	-0.5	3.14	0	0.332	gi|27574244|pdb|1O1N|A	0	K	FLASVSTVLTSK	Y
4341	2	41	B	-0.2	2.72	0	0.362	gi|27574244|pdb|1O1N|A	0	K	FLASVSTVLTSK	Y
4461	2	41	B	-0.1	2.53	0	0.411	gi|27574244|pdb|1O1N|A	0	K	FLASVSTVLTSK	Y
4583	2	41	B	-0.2	2.88	0	0.358	gi|27574244|pdb|1O1N|A	0	K	FLASVSTVLTSK	Y
4585	2	41	B	-0.3	2.85	0	0.364	gi|27574244|pdb|1O1N|A	0	K	FLASVSTVLTSK	Y
2718	2	41	A	4.62	2.98	0.99	0.99	gi|12845853|dbj|BAB26925.1|	0	K	IGGDGAEYGAEALER	M
2656	2	41	A	2.98	3.76	0.114	0.114	gi|344254270|gb|EGW10374.1|	99	K	IGGHGAEYGAEALER	M
4523	2	41	A	-0.4	3.07	0.102	0.102	gi|344254270|gb|EGW10374.1|	99	K	IGGHGAEYGAEALER	M
2563	2	41	B	3.92	4.27	0.104	0.104	gi|344254270|gb|EGW10374.1|	99	K	IGGHGAEYGAEALER	M
2575	2	41	B	3.39	4.11	0.101	0.101	gi|344254270|gb|EGW10374.1|	99	K	IGGHGAEYGAEALER	M
2734	2	41	B	4.25	3.59	0.102	0.102	gi|344254270|gb|EGW10374.1|	99	K	IGGHGAEYGAEALER	M
2743	2	41	B	4.06	3.4	0.101	0.101	gi|344254270|gb|EGW10374.1|	99	K	IGGHGAEYGAEALER	M
4874	3	41	A	-0.1	3.1	0.626	0.626	gi|122513|sp|P02088.2|HBB1_MOUSE	65	K	KVITAFNDGLNHLDSLK	G
4881	3	41	A	-0.1	3.34	0.601	0.601	gi|122513|sp|P02088.2|HBB1_MOUSE	65	K	KVITAFNDGLNHLDSLK	G
4634	3	41	B	0.02	3.01	0.591	0.591	gi|122513|sp|P02088.2|HBB1_MOUSE	65	K	KVITAFNDGLNHLDSLK	G
4659	3	41	B	-0.1	3.09	0.499	0.499	gi|122513|sp|P02088.2|HBB1_MOUSE	65	K	KVITAFNDGLNHLDSLK	G
2362	2	41	B	4.02	2.65	0.487	0.487	gi|27574244|pdb|1O1N|A	99	K	LRVDPVNFK	L
2583	2	41	A	4.27	2.87	0.226	0.226	gi|12833511|dbj|BAB22552.1|	31	R	M*FASFPTTK	T
2630	2	41	A	3.53	3.3	0.141	0.141	gi|344254270|gb|EGW10374.1|	99	K	TYFPHFDVSHGSAQVK	A
2639	2	41	A	3.67	3.35	0.146	0.146	gi|344254270|gb|EGW10374.1|	99	K	TYFPHFDVSHGSAQVK	A
2421	2	41	B	4.96	2.99	0.163	0.163	gi|344254270|gb|EGW10374.1|	99	K	TYFPHFDVSHGSAQVK	A
4763	3	41	A	1.2	5.54	0.954	0.954	gi|159137400|gb|ABW88847.1|	2	K	VADALATAAGHLDDLPGALSALSDLHAHK	L
2806	3	41	B	4.89	3.72	0.95	0.95	gi|159137400|gb|ABW88847.1|	2	K	VADALATAAGHLDDLPGALSALSDLHAHK	L
4539	3	41	B	-0.3	5.63	0.936	0.936	gi|159137400|gb|ABW88847.1|	2	K	VADALATAAGHLDDLPGALSALSDLHAHK	L
3009	3	41	A	-2.4	3.11	0.254	0.254	gi|378548361|sp|B3EWD7.1|HBA_TAMHU	0	K	VAEALATAAGHLDDLPGALSALSDLHAHK	L
4513	2	41	A	-0.2	3.35	0.678	0.678	gi|122513|sp|P02088.2|HBB1_MOUSE	65	K	VITAFNDGLNHLDSLK	G
4519	2	41	A	-0.6	2.97	0.667	0.667	gi|122513|sp|P02088.2|HBB1_MOUSE	65	K	VITAFNDGLNHLDSLK	G
4632	2	41	A	-0.1	3.49	0.671	0.671	gi|122513|sp|P02088.2|HBB1_MOUSE	65	K	VITAFNDGLNHLDSLK	G
4746	2	41	A	0.17	3.17	0.693	0.693	gi|122513|sp|P02088.2|HBB1_MOUSE	65	K	VITAFNDGLNHLDSLK	G
4752	2	41	A	-0.1	2.75	0.737	0.737	gi|122513|sp|P02088.2|HBB1_MOUSE	65	K	VITAFNDGLNHLDSLK	G
4895	2	41	A	-0	2.91	0.618	0.618	gi|122513|sp|P02088.2|HBB1_MOUSE	65	K	VITAFNDGLNHLDSLK	G
4905	2	41	A	0.05	2.53	0.726	0.726	gi|122513|sp|P02088.2|HBB1_MOUSE	65	K	VITAFNDGLNHLDSLK	G
2639	2	41	B	4.8	4.38	0.575	0.575	gi|122513|sp|P02088.2|HBB1_MOUSE	65	K	VITAFNDGLNHLDSLK	G
4283	2	41	B	-0.7	3.03	0.677	0.677	gi|122513|sp|P02088.2|HBB1_MOUSE	65	K	VITAFNDGLNHLDSLK	G
4319	2	41	B	-0.9	3.34	0.7	0.7	gi|122513|sp|P02088.2|HBB1_MOUSE	65	K	VITAFNDGLNHLDSLK	G

Scan	z	Sample ID	Band	PPM	XCorr	Delta correlation	Unique delta correlation	Reference	Redundancy		Peptides	
4325	2	41	B	-0.7	3.25	0.672	0.672	gi|122513|sp|P02088.2|HBB1_MOUSE	65	K	VITAFNDGLNHLDSLK	G
4661	2	41	B	-0.1	2.51	0.835	0.835	gi|122513|sp|P02088.2|HBB1_MOUSE	65	K	VITAFNDGLNHLDSLK	G
2081	2	41	A	-0.4	3.05	0.486	0.486	gi|241913510|pdb|3HRW|C	2	-	VLSGEDKSNIK	A
2207	2	41	A	1.35	3.45	0.492	0.492	gi|241913510|pdb|3HRW|C	2	-	VLSGEDKSNIK	A
2210	2	41	A	1.32	3.24	0.572	0.572	gi|241913510|pdb|3HRW|C	2	-	VLSGEDKSNIK	A
2335	2	41	A	1.31	3.52	0.512	0.512	gi|241913510|pdb|3HRW|C	2	-	VLSGEDKSNIK	A
2340	2	41	A	1.29	3.44	0.528	0.528	gi|241913510|pdb|3HRW|C	2	-	VLSGEDKSNIK	A
2466	2	41	A	1.28	3.51	0.532	0.532	gi|241913510|pdb|3HRW|C	2	-	VLSGEDKSNIK	A
1881	2	41	B	3.63	2.85	0.534	0.534	gi|241913510|pdb|3HRW|C	2	-	VLSGEDKSNIK	A
1884	2	41	B	-0.6	2.87	0.568	0.568	gi|241913510|pdb|3HRW|C	2	-	VLSGEDKSNIK	A
2005	2	41	B	2.17	3.6	0.522	0.522	gi|241913510|pdb|3HRW|C	2	-	VLSGEDKSNIK	A
2007	2	41	B	1.4	3.48	0.518	0.518	gi|241913510|pdb|3HRW|C	2	-	VLSGEDKSNIK	A
2127	2	41	B	1.03	3.27	0.504	0.504	gi|241913510|pdb|3HRW|C	2	-	VLSGEDKSNIK	A
2133	2	41	B	0.99	3.52	0.523	0.523	gi|241913510|pdb|3HRW|C	2	-	VLSGEDKSNIK	A
2660	2	41	A	2.9	3.74	0	0.303	gi|378548370|sp|B3EWD8.1|HBB_TAMHU	0	K	NVADEVGGEALGR^	L
4088	2	41	A	3.05	3.93	0	0.252	gi|378548370|sp|B3EWD8.1|HBB_TAMHU	0	K	NVADEVGGEALGR^	L
4229	2	41	A	0.19	3.98	0	0.279	gi|378548370|sp|B3EWD8.1|HBB_TAMHU	0	K	NVADEVGGEALGR^	L
4710	2	41	A	-0.6	3.38	0	0.165	gi|378548370|sp|B3EWD8.1|HBB_TAMHU	0	K	NVADEVGGEALGR^	L
4717	2	41	A	-0.5	3.72	0	0.273	gi|378548370|sp|B3EWD8.1|HBB_TAMHU	0	K	NVADEVGGEALGR^	L
5072	2	41	A	-0.8	3.38	0	0.22	gi|378548370|sp|B3EWD8.1|HBB_TAMHU	0	K	NVADEVGGEALGR^	L
2576	2	41	B	2.18	3.56	0	0.247	gi|378548370|sp|B3EWD8.1|HBB_TAMHU	0	K	NVADEVGGEALGR^	L
4307	2	41	B	-0.3	2.66	0	0.329	gi|378548370|sp|B3EWD8.1|HBB_TAMHU	0	K	NVADEVGGEALGR^	L
4425	2	41	B	-0.5	3.78	0	0.234	gi|378548370|sp|B3EWD8.1|HBB_TAMHU	0	K	NVADEVGGEALGR^	L
4670	2	41	B	-0.3	4.04	0	0.247	gi|378548370|sp|B3EWD8.1|HBB_TAMHU	0	K	NVADEVGGEALGR^	L
4796	2	41	B	-0.3	3.72	0	0.207	gi|378548370|sp|B3EWD8.1|HBB_TAMHU	0	K	NVADEVGGEALGR^	L
2382	2	41	B	3.08	3.28	0.15	0.15	gi|667267074|ref|XP_008569536.1|	4	K	VNVDDVGGEALGR	L
2388	2	41	B	3.08	2.79	0.152	0.152	gi|667267074|ref|XP_008569536.1|	4	K	VNVDDVGGEALGR	L
2666	2	41	A	2.81	3.91	0.899	0.899	gi|12847007|dbj|BAB27399.1|	60	K	VVAGVAAALAHK	Y
2668	2	41	A	2.81	3.46	0.85	0.85	gi|12847007|dbj|BAB27399.1|	60	K	VVAGVAAALAHK	Y
2789	2	41	A	3.32	3.46	0.882	0.882	gi|12847007|dbj|BAB27399.1|	60	K	VVAGVAAALAHK	Y
2806	2	41	A	3.41	3.71	0.873	0.873	gi|12847007|dbj|BAB27399.1|	60	K	VVAGVAAALAHK	Y
4207	2	41	A	-0.3	3.07	0.876	0.876	gi|12847007|dbj|BAB27399.1|	60	K	VVAGVAAALAHK	Y
4239	2	41	A	1.02	3.21	0.889	0.889	gi|12847007|dbj|BAB27399.1|	60	K	VVAGVAAALAHK	Y
4331	2	41	A	-1	2.92	0.879	0.879	gi|12847007|dbj|BAB27399.1|	60	K	VVAGVAAALAHK	Y
4339	2	41	A	0.62	3.09	0.951	0.951	gi|12847007|dbj|BAB27399.1|	60	K	VVAGVAAALAHK	Y
4466	2	41	A	3.34	3.25	0.955	0.955	gi|12847007|dbj|BAB27399.1|	60	K	VVAGVAAALAHK	Y
4471	2	41	A	-0.5	3.57	0.959	0.959	gi|12847007|dbj|BAB27399.1|	60	K	VVAGVAAALAHK	Y
4586	2	41	A	-0.3	3.14	0.956	0.956	gi|12847007|dbj|BAB27399.1|	60	K	VVAGVAAALAHK	Y

Scan	z	Sample ID	Band	PPM	XCorr	Delta correlation	Unique delta correlation	Reference	Redundancy		Peptides	
4596	2	41	A	-0.4	3.13	0.911	0.911	gi|12847007|dbj|BAB27399.1|	60	K	VVAGVAAALAHK	Y
4700	2	41	A	-0.3	2.96	0.986	0.986	gi|12847007|dbj|BAB27399.1|	60	K	VVAGVAAALAHK	Y
4839	2	41	A	-0.4	3.08	0.949	0.949	gi|12847007|dbj|BAB27399.1|	60	K	VVAGVAAALAHK	Y
4843	2	41	A	-0.4	3.64	0.891	0.891	gi|12847007|dbj|BAB27399.1|	60	K	VVAGVAAALAHK	Y
4957	2	41	A	-0.5	3.54	0.912	0.912	gi|12847007|dbj|BAB27399.1|	60	K	VVAGVAAALAHK	Y
4962	2	41	A	-0.5	3.43	0.873	0.873	gi|12847007|dbj|BAB27399.1|	60	K	VVAGVAAALAHK	Y
2324	2	41	B	3.29	3.63	0.871	0.871	gi|12847007|dbj|BAB27399.1|	60	K	VVAGVAAALAHK	Y
2332	2	41	B	3.31	3.8	0.896	0.896	gi|12847007|dbj|BAB27399.1|	60	K	VVAGVAAALAHK	Y
2451	2	41	B	3.52	3.18	0.843	0.843	gi|12847007|dbj|BAB27399.1|	60	K	VVAGVAAALAHK	Y
2456	2	41	B	3.58	2.99	0.907	0.907	gi|12847007|dbj|BAB27399.1|	60	K	VVAGVAAALAHK	Y
2577	2	41	B	2.32	3.37	0.818	0.818	gi|12847007|dbj|BAB27399.1|	60	K	VVAGVAAALAHK	Y
2591	2	41	B	3.47	3.89	0.894	0.894	gi|12847007|dbj|BAB27399.1|	60	K	VVAGVAAALAHK	Y
2736	2	41	B	3.56	2.73	0.95	0.95	gi|12847007|dbj|BAB27399.1|	60	K	VVAGVAAALAHK	Y
4017	2	41	B	-1.1	3.28	0.946	0.946	gi|12847007|dbj|BAB27399.1|	60	K	VVAGVAAALAHK	Y
4020	2	41	B	1.24	3.16	0.91	0.91	gi|12847007|dbj|BAB27399.1|	60	K	VVAGVAAALAHK	Y
4156	2	41	B	-1.8	3.31	0.946	0.946	gi|12847007|dbj|BAB27399.1|	60	K	VVAGVAAALAHK	Y
4163	2	41	B	-0.6	3.26	0.938	0.938	gi|12847007|dbj|BAB27399.1|	60	K	VVAGVAAALAHK	Y
4282	2	41	B	-0.6	3.56	0.947	0.947	gi|12847007|dbj|BAB27399.1|	60	K	VVAGVAAALAHK	Y
4302	2	41	B	-0.5	3.4	0.893	0.893	gi|12847007|dbj|BAB27399.1|	60	K	VVAGVAAALAHK	Y
4527	2	41	B	0.07	2.81	0.923	0.923	gi|12847007|dbj|BAB27399.1|	60	K	VVAGVAAALAHK	Y
4540	2	41	B	0.05	2.91	0.91	0.91	gi|12847007|dbj|BAB27399.1|	60	K	VVAGVAAALAHK	Y
4652	2	41	B	-0.2	3.43	0.945	0.945	gi|12847007|dbj|BAB27399.1|	60	K	VVAGVAAALAHK	Y
4658	2	41	B	-0.3	2.91	0.911	0.911	gi|12847007|dbj|BAB27399.1|	60	K	VVAGVAAALAHK	Y
2687	2	41	A	2.97	4.36	0.949	0.949	gi|12847007|dbj|BAB27399.1|	60	K	VVAGVAAALAHKYH	-
2690	2	41	A	2.99	4.46	0.938	0.938	gi|12847007|dbj|BAB27399.1|	60	K	VVAGVAAALAHKYH	-
2825	2	41	A	3.65	4.07	0.952	0.952	gi|12847007|dbj|BAB27399.1|	60	K	VVAGVAAALAHKYH	-
2835	2	41	A	3.65	3.62	0.944	0.944	gi|12847007|dbj|BAB27399.1|	60	K	VVAGVAAALAHKYH	-
4570	2	41	A	0.45	2.67	0.952	0.952	gi|12847007|dbj|BAB27399.1|	60	K	VVAGVAAALAHKYH	-
4876	2	41	A	0.18	3.22	0.937	0.937	gi|12847007|dbj|BAB27399.1|	60	K	VVAGVAAALAHKYH	-
4884	2	41	A	0.28	3.06	0.955	0.955	gi|12847007|dbj|BAB27399.1|	60	K	VVAGVAAALAHKYH	-
2351	2	41	B	4.72	4.59	0.918	0.918	gi|12847007|dbj|BAB27399.1|	60	K	VVAGVAAALAHKYH	-
2363	2	41	B	4.72	4.65	0.91	0.91	gi|12847007|dbj|BAB27399.1|	60	K	VVAGVAAALAHKYH	-
2545	2	41	B	3.11	3.63	0.906	0.906	gi|12847007|dbj|BAB27399.1|	60	K	VVAGVAAALAHKYH	-
4632	2	41	B	-1.8	2.62	0.942	0.942	gi|12847007|dbj|BAB27399.1|	60	K	VVAGVAAALAHKYH	-
2920	2	41	A	4.33	4.02	0.515	0.515	gi|187369324|dbj|BAG31393.1|	73	R	YFDSFGDLSSASAIM*GNAK	V
2930	2	41	A	4.3	4.7	0.49	0.49	gi|187369324|dbj|BAG31393.1|	73	R	YFDSFGDLSSASAIM*GNAK	V
3248	2	41	A	3.79	4.06	0.566	0.566	gi|187369324|dbj|BAG31393.1|	73	R	YFDSFGDLSSASAIM*GNAK	V
3398	2	41	A	4.87	2.72	0.606	0.606	gi|187369324|dbj|BAG31393.1|	73	R	YFDSFGDLSSASAIM*GNAK	V

Scan	z	Sample ID	Band	PPM	XCorr	Delta correlation	Unique delta correlation	Reference	Redundancy		Peptides	
4534	2	41	A	1.4	3.32	0.66	0.66	gi|187369324|dbj|BAG31393.1|	73	R	YFDSFGDLSSASAIM*GNAK	V
4609	2	41	A	-0.5	2.57	0.685	0.685	gi|187369324|dbj|BAG31393.1|	73	R	YFDSFGDLSSASAIM*GNAK	V
4742	2	41	A	1.37	3.03	0.657	0.657	gi|187369324|dbj|BAG31393.1|	73	R	YFDSFGDLSSASAIM*GNAK	V
4882	2	41	A	0.14	3.46	0.698	0.698	gi|187369324|dbj|BAG31393.1|	73	R	YFDSFGDLSSASAIM*GNAK	V
4914	2	41	A	0.11	3.51	0.664	0.664	gi|187369324|dbj|BAG31393.1|	73	R	YFDSFGDLSSASAIM*GNAK	V
4924	2	41	A	-0.1	3.66	0.684	0.684	gi|187369324|dbj|BAG31393.1|	73	R	YFDSFGDLSSASAIM*GNAK	V
3058	2	41	B	4.67	2.87	0.666	0.666	gi|187369324|dbj|BAG31393.1|	73	R	YFDSFGDLSSASAIM*GNAK	V
3074	2	41	B	4.91	3.26	0.554	0.554	gi|187369324|dbj|BAG31393.1|	73	R	YFDSFGDLSSASAIM*GNAK	V
4303	2	41	B	0.29	3.77	0.607	0.607	gi|187369324|dbj|BAG31393.1|	73	R	YFDSFGDLSSASAIM*GNAK	V
4315	2	41	B	1.21	4.12	0.611	0.611	gi|187369324|dbj|BAG31393.1|	73	R	YFDSFGDLSSASAIM*GNAK	V
4431	2	41	B	0.28	3.37	0.657	0.657	gi|187369324|dbj|BAG31393.1|	73	R	YFDSFGDLSSASAIM*GNAK	V
4653	2	41	B	-0.5	3.1	0.647	0.647	gi|187369324|dbj|BAG31393.1|	73	R	YFDSFGDLSSASAIM*GNAK	V
4667	2	41	B	-1	3.26	0.638	0.638	gi|187369324|dbj|BAG31393.1|	73	R	YFDSFGDLSSASAIM*GNAK	V
3012	2	41	A	4.85	3.68	0.723	0.723	gi|187369324|dbj|BAG31393.1|	73	R	YFDSFGDLSSASAIMGNAK	V
3029	2	41	A	4.94	4.83	0.64	0.64	gi|187369324|dbj|BAG31393.1|	73	R	YFDSFGDLSSASAIMGNAK	V
3135	2	41	A	4.9	4.08	0.64	0.64	gi|187369324|dbj|BAG31393.1|	73	R	YFDSFGDLSSASAIMGNAK	V
3140	2	41	A	4.82	3.95	0.696	0.696	gi|187369324|dbj|BAG31393.1|	73	R	YFDSFGDLSSASAIMGNAK	V
3253	2	41	A	4.96	4.78	0.58	0.58	gi|187369324|dbj|BAG31393.1|	73	R	YFDSFGDLSSASAIMGNAK	V
3268	2	41	A	4.69	5.37	0.554	0.554	gi|187369324|dbj|BAG31393.1|	73	R	YFDSFGDLSSASAIMGNAK	V
4037	2	41	A	4.46	3.11	0.701	0.701	gi|187369324|dbj|BAG31393.1|	73	R	YFDSFGDLSSASAIMGNAK	V
4069	3	41	A	2.89	4.24	0.382	0.382	gi|187369324|dbj|BAG31393.1|	73	R	YFDSFGDLSSASAIMGNAK	V
4071	3	41	A	3.23	5.56	0.364	0.364	gi|187369324|dbj|BAG31393.1|	73	R	YFDSFGDLSSASAIMGNAK	V
4175	2	41	A	1.46	3.79	0.604	0.604	gi|187369324|dbj|BAG31393.1|	73	R	YFDSFGDLSSASAIMGNAK	V
4200	3	41	A	2.5	3.01	0.274	0.274	gi|187369324|dbj|BAG31393.1|	73	R	YFDSFGDLSSASAIMGNAK	V
4202	3	41	A	2.5	4.62	0.312	0.312	gi|187369324|dbj|BAG31393.1|	73	R	YFDSFGDLSSASAIMGNAK	V
4299	2	41	A	0.43	4.16	0.638	0.638	gi|187369324|dbj|BAG31393.1|	73	R	YFDSFGDLSSASAIMGNAK	V
4301	2	41	A	0.52	4.8	0.67	0.67	gi|187369324|dbj|BAG31393.1|	73	R	YFDSFGDLSSASAIMGNAK	V
4327	3	41	A	-0.5	4.19	0.33	0.33	gi|187369324|dbj|BAG31393.1|	73	R	YFDSFGDLSSASAIMGNAK	V
4329	3	41	A	-0.5	4.73	0.407	0.407	gi|187369324|dbj|BAG31393.1|	73	R	YFDSFGDLSSASAIMGNAK	V
4422	2	41	A	0.28	5.15	0.608	0.608	gi|187369324|dbj|BAG31393.1|	73	R	YFDSFGDLSSASAIMGNAK	V
4424	2	41	A	0.25	5.62	0.567	0.567	gi|187369324|dbj|BAG31393.1|	73	R	YFDSFGDLSSASAIMGNAK	V
4450	3	41	A	0.25	4.88	0.37	0.37	gi|187369324|dbj|BAG31393.1|	73	R	YFDSFGDLSSASAIMGNAK	V
4455	3	41	A	1.01	4.64	0.331	0.331	gi|187369324|dbj|BAG31393.1|	73	R	YFDSFGDLSSASAIMGNAK	V
4538	2	41	A	0.16	6.31	0.528	0.528	gi|187369324|dbj|BAG31393.1|	73	R	YFDSFGDLSSASAIMGNAK	V
4543	2	41	A	0.1	6.74	0.554	0.554	gi|187369324|dbj|BAG31393.1|	73	R	YFDSFGDLSSASAIMGNAK	V
4572	3	41	A	-0.8	4.19	0.351	0.351	gi|187369324|dbj|BAG31393.1|	73	R	YFDSFGDLSSASAIMGNAK	V
4581	3	41	A	-0.8	4.34	0.366	0.366	gi|187369324|dbj|BAG31393.1|	73	R	YFDSFGDLSSASAIMGNAK	V
4656	2	41	A	-0.1	6.04	0.55	0.55	gi|187369324|dbj|BAG31393.1|	73	R	YFDSFGDLSSASAIMGNAK	V

Scan	z	Sample ID	Band	PPM	XCorr	Delta correlation	Unique delta correlation	Reference	Redundancy		Peptides	
4667	2	41	A	-0.1	4.89	0.56	0.56	gi|187369324|dbj|BAG31393.1|	73	R	YFDSFGDLSSASAIMGNAK	V
4705	3	41	A	-0.4	3.82	0.34	0.34	gi|187369324|dbj|BAG31393.1|	73	R	YFDSFGDLSSASAIMGNAK	V
4782	2	41	A	0.18	3.96	0.632	0.632	gi|187369324|dbj|BAG31393.1|	73	R	YFDSFGDLSSASAIMGNAK	V
4785	2	41	A	0.38	4.74	0.636	0.636	gi|187369324|dbj|BAG31393.1|	73	R	YFDSFGDLSSASAIMGNAK	V
4817	3	41	A	-0.4	4.66	0.327	0.327	gi|187369324|dbj|BAG31393.1|	73	R	YFDSFGDLSSASAIMGNAK	V
4820	3	41	A	-0.4	4.46	0.331	0.331	gi|187369324|dbj|BAG31393.1|	73	R	YFDSFGDLSSASAIMGNAK	V
4899	2	41	A	0.2	5.68	0.581	0.581	gi|187369324|dbj|BAG31393.1|	73	R	YFDSFGDLSSASAIMGNAK	V
4908	2	41	A	0.24	5.48	0.587	0.587	gi|187369324|dbj|BAG31393.1|	73	R	YFDSFGDLSSASAIMGNAK	V
2913	2	41	B	4.9	4.76	0.583	0.583	gi|187369324|dbj|BAG31393.1|	73	R	YFDSFGDLSSASAIMGNAK	V
2916	2	41	B	4.78	5.11	0.558	0.558	gi|187369324|dbj|BAG31393.1|	73	R	YFDSFGDLSSASAIMGNAK	V
3030	2	41	B	4.74	5.64	0.579	0.579	gi|187369324|dbj|BAG31393.1|	73	R	YFDSFGDLSSASAIMGNAK	V
3039	2	41	B	4.67	3.64	0.621	0.621	gi|187369324|dbj|BAG31393.1|	73	R	YFDSFGDLSSASAIMGNAK	V
3659	3	41	B	4.74	3.93	0.331	0.331	gi|187369324|dbj|BAG31393.1|	73	R	YFDSFGDLSSASAIMGNAK	V
3667	3	41	B	2.2	3.63	0.333	0.333	gi|187369324|dbj|BAG31393.1|	73	R	YFDSFGDLSSASAIMGNAK	V
3682	2	41	B	4.52	3.79	0.659	0.659	gi|187369324|dbj|BAG31393.1|	73	R	YFDSFGDLSSASAIMGNAK	V
3796	3	41	B	4.23	5.22	0.355	0.355	gi|187369324|dbj|BAG31393.1|	73	R	YFDSFGDLSSASAIMGNAK	V
3799	3	41	B	3.44	4.76	0.297	0.297	gi|187369324|dbj|BAG31393.1|	73	R	YFDSFGDLSSASAIMGNAK	V
3812	2	41	B	4.72	3.62	0.659	0.659	gi|187369324|dbj|BAG31393.1|	73	R	YFDSFGDLSSASAIMGNAK	V
3927	3	41	B	-1.2	4.31	0.352	0.352	gi|187369324|dbj|BAG31393.1|	73	R	YFDSFGDLSSASAIMGNAK	V
3938	2	41	B	-0.2	4.55	0.603	0.603	gi|187369324|dbj|BAG31393.1|	73	R	YFDSFGDLSSASAIMGNAK	V
3940	2	41	B	-1.2	4.75	0.576	0.576	gi|187369324|dbj|BAG31393.1|	73	R	YFDSFGDLSSASAIMGNAK	V
3944	3	41	B	-0.4	4.03	0.319	0.319	gi|187369324|dbj|BAG31393.1|	73	R	YFDSFGDLSSASAIMGNAK	V
4060	2	41	B	0.42	3.82	0.721	0.721	gi|187369324|dbj|BAG31393.1|	73	R	YFDSFGDLSSASAIMGNAK	V
4062	2	41	B	0.35	4.43	0.646	0.646	gi|187369324|dbj|BAG31393.1|	73	R	YFDSFGDLSSASAIMGNAK	V
4065	3	41	B	-0.4	4.49	0.346	0.346	gi|187369324|dbj|BAG31393.1|	73	R	YFDSFGDLSSASAIMGNAK	V
4067	3	41	B	-0.3	5.14	0.375	0.375	gi|187369324|dbj|BAG31393.1|	73	R	YFDSFGDLSSASAIMGNAK	V
4184	2	41	B	-0	5.14	0.578	0.578	gi|187369324|dbj|BAG31393.1|	73	R	YFDSFGDLSSASAIMGNAK	V
4186	2	41	B	-0.3	4.89	0.608	0.608	gi|187369324|dbj|BAG31393.1|	73	R	YFDSFGDLSSASAIMGNAK	V
4188	3	41	B	-0.4	5.33	0.312	0.312	gi|187369324|dbj|BAG31393.1|	73	R	YFDSFGDLSSASAIMGNAK	V
4193	3	41	B	-0.5	4.6	0.346	0.346	gi|187369324|dbj|BAG31393.1|	73	R	YFDSFGDLSSASAIMGNAK	V
4297	2	41	B	-0.2	5.94	0.506	0.506	gi|187369324|dbj|BAG31393.1|	73	R	YFDSFGDLSSASAIMGNAK	V
4306	2	41	B	-0.1	5.72	0.563	0.563	gi|187369324|dbj|BAG31393.1|	73	R	YFDSFGDLSSASAIMGNAK	V
4309	3	41	B	-0.4	4.35	0.321	0.321	gi|187369324|dbj|BAG31393.1|	73	R	YFDSFGDLSSASAIMGNAK	V
4317	3	41	B	-0.5	4.63	0.318	0.318	gi|187369324|dbj|BAG31393.1|	73	R	YFDSFGDLSSASAIMGNAK	V
4413	2	41	B	0.14	4.85	0.618	0.618	gi|187369324|dbj|BAG31393.1|	73	R	YFDSFGDLSSASAIMGNAK	V
4424	2	41	B	0.04	5.15	0.598	0.598	gi|187369324|dbj|BAG31393.1|	73	R	YFDSFGDLSSASAIMGNAK	V
4426	3	41	B	-0.6	3.66	0.302	0.302	gi|187369324|dbj|BAG31393.1|	73	R	YFDSFGDLSSASAIMGNAK	V
4437	3	41	B	-0.4	3.69	0.319	0.319	gi|187369324|dbj|BAG31393.1|	73	R	YFDSFGDLSSASAIMGNAK	V

Scan	z	Sample ID	Band	PPM	XCorr	Delta correlation	Unique delta correlation	Reference	Redundancy		Peptides	
4544	2	41	B	-0.1	4.99	0.584	0.584	gi|187369324|dbj|BAG31393.1|	73	R	YFDSFGDLSSASAIMGNAK	V
4554	2	41	B	-0.6	3.46	0.659	0.659	gi|187369324|dbj|BAG31393.1|	73	R	YFDSFGDLSSASAIMGNAK	V
4560	3	41	B	0.05	3.47	0.223	0.223	gi|187369324|dbj|BAG31393.1|	73	R	YFDSFGDLSSASAIMGNAK	V
4565	3	41	B	-0.2	3.74	0.376	0.376	gi|187369324|dbj|BAG31393.1|	73	R	YFDSFGDLSSASAIMGNAK	V
4672	2	41	B	-0.1	5.43	0.566	0.566	gi|187369324|dbj|BAG31393.1|	73	R	YFDSFGDLSSASAIMGNAK	V
4681	2	41	B	-0.2	5.03	0.616	0.616	gi|187369324|dbj|BAG31393.1|	73	R	YFDSFGDLSSASAIMGNAK	V
2331	2	42	A	3.06	3.37	0.923	0.923	gi|554577472|ref|XP_005880462.1|	74	K	AAVSGLWGK	V
2340	2	42	A	4.27	3.28	0.922	0.922	gi|554577472|ref|XP_005880462.1|	74	K	AAVSGLWGK	V
3779	2	42	A	3.02	2.73	0.987	0.987	gi|554577472|ref|XP_005880462.1|	74	K	AAVSGLWGK	V
3802	2	42	A	3.19	2.8	0.955	0.955	gi|554577472|ref|XP_005880462.1|	74	K	AAVSGLWGK	V
3928	2	42	A	-0.3	2.71	0.983	0.983	gi|554577472|ref|XP_005880462.1|	74	K	AAVSGLWGK	V
3933	2	42	A	-0.3	2.75	0.986	0.986	gi|554577472|ref|XP_005880462.1|	74	K	AAVSGLWGK	V
4056	2	42	A	-0.8	2.55	0.903	0.903	gi|554577472|ref|XP_005880462.1|	74	K	AAVSGLWGK	V
4060	2	42	A	-0.9	2.79	0.978	0.978	gi|554577472|ref|XP_005880462.1|	74	K	AAVSGLWGK	V
4176	2	42	A	-0.3	2.69	0.941	0.941	gi|554577472|ref|XP_005880462.1|	74	K	AAVSGLWGK	V
4180	2	42	A	-0.3	2.72	0.983	0.983	gi|554577472|ref|XP_005880462.1|	74	K	AAVSGLWGK	V
4296	2	42	A	-0.3	2.55	0.985	0.985	gi|554577472|ref|XP_005880462.1|	74	K	AAVSGLWGK	V
4304	2	42	A	-0.3	2.64	0.921	0.921	gi|554577472|ref|XP_005880462.1|	74	K	AAVSGLWGK	V
4551	2	42	A	-0.3	2.57	0.952	0.952	gi|554577472|ref|XP_005880462.1|	74	K	AAVSGLWGK	V
4558	2	42	A	-0.3	2.89	0.946	0.946	gi|554577472|ref|XP_005880462.1|	74	K	AAVSGLWGK	V
4675	2	42	A	-0.2	2.61	0.951	0.951	gi|554577472|ref|XP_005880462.1|	74	K	AAVSGLWGK	V
2238	2	42	B	3.51	2.63	0.906	0.906	gi|554577472|ref|XP_005880462.1|	74	K	AAVSGLWGK	V
2242	2	42	B	3.51	2.53	0.982	0.982	gi|554577472|ref|XP_005880462.1|	74	K	AAVSGLWGK	V
3539	2	42	B	3.79	2.74	0.983	0.983	gi|554577472|ref|XP_005880462.1|	74	K	AAVSGLWGK	V
3673	2	42	B	2.95	2.72	0.892	0.892	gi|554577472|ref|XP_005880462.1|	74	K	AAVSGLWGK	V
3679	2	42	B	2.94	2.51	0.901	0.901	gi|554577472|ref|XP_005880462.1|	74	K	AAVSGLWGK	V
3816	2	42	B	-0.8	2.5	0.984	0.984	gi|554577472|ref|XP_005880462.1|	74	K	AAVSGLWGK	V
3819	2	42	B	-0.7	2.8	0.943	0.943	gi|554577472|ref|XP_005880462.1|	74	K	AAVSGLWGK	V
3936	2	42	B	-0.5	2.82	0.95	0.95	gi|554577472|ref|XP_005880462.1|	74	K	AAVSGLWGK	V
3938	2	42	B	-0.4	2.77	0.98	0.98	gi|554577472|ref|XP_005880462.1|	74	K	AAVSGLWGK	V
4057	2	42	B	-0.4	2.82	0.955	0.955	gi|554577472|ref|XP_005880462.1|	74	K	AAVSGLWGK	V
4063	2	42	B	-0.5	2.85	0.949	0.949	gi|554577472|ref|XP_005880462.1|	74	K	AAVSGLWGK	V
4296	2	42	B	-0	2.68	0.985	0.985	gi|554577472|ref|XP_005880462.1|	74	K	AAVSGLWGK	V
4303	2	42	B	0	2.74	0.985	0.985	gi|554577472|ref|XP_005880462.1|	74	K	AAVSGLWGK	V
4418	2	42	B	-0.5	2.76	0.972	0.972	gi|554577472|ref|XP_005880462.1|	74	K	AAVSGLWGK	V
4424	2	42	B	-0.5	2.7	0.985	0.985	gi|554577472|ref|XP_005880462.1|	74	K	AAVSGLWGK	V
2369	2	42	A	2.09	3.89	0.968	0.968	gi|532054982|ref|XP_005370208.1|	94	K	DFTPAAQAAFQK	V
2380	2	42	A	3.57	3.93	0.948	0.948	gi|532054982|ref|XP_005370208.1|	94	K	DFTPAAQAAFQK	V

Scan	z	Sample ID	Band	PPM	XCorr	Delta correlation	Unique delta correlation	Reference	Redundancy		Peptides	
2389	3	42	A	2.8	3.74	0.878	0.878	gi|532054982|ref|XP_005370208.1|	94	K	DFTPAAQAAFQK	V
2399	3	42	A	2.63	3.04	0.869	0.869	gi|532054982|ref|XP_005370208.1|	94	K	DFTPAAQAAFQK	V
2507	2	42	A	0.18	3.03	0.918	0.918	gi|532054982|ref|XP_005370208.1|	94	K	DFTPAAQAAFQK	V
2515	2	42	A	0.22	2.51	0.981	0.981	gi|532054982|ref|XP_005370208.1|	94	K	DFTPAAQAAFQK	V
4088	2	42	A	-0.1	2.96	0.986	0.986	gi|532054982|ref|XP_005370208.1|	94	K	DFTPAAQAAFQK	V
4381	2	42	A	-0.3	2.51	0.886	0.886	gi|532054982|ref|XP_005370208.1|	94	K	DFTPAAQAAFQK	V
2248	2	42	B	4.24	3.61	0.953	0.953	gi|532054982|ref|XP_005370208.1|	94	K	DFTPAAQAAFQK	V
2258	2	42	B	4.26	3.35	0.882	0.882	gi|532054982|ref|XP_005370208.1|	94	K	DFTPAAQAAFQK	V
3754	2	42	B	-0.4	2.5	0.962	0.962	gi|532054982|ref|XP_005370208.1|	94	K	DFTPAAQAAFQK	V
3919	2	42	B	-0.2	2.62	0.962	0.962	gi|532054982|ref|XP_005370208.1|	94	K	DFTPAAQAAFQK	V
4047	2	42	B	0.21	2.61	0.953	0.953	gi|532054982|ref|XP_005370208.1|	94	K	DFTPAAQAAFQK	V
4092	2	42	B	-0.2	2.69	0.957	0.957	gi|532054982|ref|XP_005370208.1|	94	K	DFTPAAQAAFQK	V
4530	2	42	B	-0.5	2.64	0.941	0.941	gi|532054982|ref|XP_005370208.1|	94	K	DFTPAAQAAFQK	V
2599	2	42	A	2.55	3.32	0	0.314	gi|27574244|pdb|1O1N|A	0	K	FLASVSTVLTSK	Y
2612	2	42	A	1.26	3.26	0	0.316	gi|27574244|pdb|1O1N|A	0	K	FLASVSTVLTSK	Y
2863	2	42	A	4.32	2.73	0	0.278	gi|27574244|pdb|1O1N|A	0	K	FLASVSTVLTSK	Y
2868	2	42	A	4.41	2.65	0	0.345	gi|27574244|pdb|1O1N|A	0	K	FLASVSTVLTSK	Y
2993	2	42	A	4.27	2.67	0	0.283	gi|27574244|pdb|1O1N|A	0	K	FLASVSTVLTSK	Y
2996	2	42	A	4.02	2.81	0	0.277	gi|27574244|pdb|1O1N|A	0	K	FLASVSTVLTSK	Y
3128	2	42	A	3.47	3.21	0	0.362	gi|27574244|pdb|1O1N|A	0	K	FLASVSTVLTSK	Y
3137	2	42	A	3.43	3.24	0	0.311	gi|27574244|pdb|1O1N|A	0	K	FLASVSTVLTSK	Y
3634	2	42	A	4.21	3.15	0	0.378	gi|27574244|pdb|1O1N|A	0	K	FLASVSTVLTSK	Y
3645	2	42	A	3.26	2.89	0	0.395	gi|27574244|pdb|1O1N|A	0	K	FLASVSTVLTSK	Y
3788	2	42	A	1.87	3.19	0	0.335	gi|27574244|pdb|1O1N|A	0	K	FLASVSTVLTSK	Y
3912	2	42	A	-0.3	3.22	0	0.349	gi|27574244|pdb|1O1N|A	0	K	FLASVSTVLTSK	Y
3914	2	42	A	-0.1	2.82	0	0.379	gi|27574244|pdb|1O1N|A	0	K	FLASVSTVLTSK	Y
4035	2	42	A	-0.2	3.12	0	0.369	gi|27574244|pdb|1O1N|A	0	K	FLASVSTVLTSK	Y
4037	2	42	A	-0.2	3.36	0	0.347	gi|27574244|pdb|1O1N|A	0	K	FLASVSTVLTSK	Y
4157	2	42	A	-0.3	2.84	0	0.368	gi|27574244|pdb|1O1N|A	0	K	FLASVSTVLTSK	Y
4159	2	42	A	-0.3	3.13	0	0.312	gi|27574244|pdb|1O1N|A	0	K	FLASVSTVLTSK	Y
4279	2	42	A	-0.4	2.6	0	0.349	gi|27574244|pdb|1O1N|A	0	K	FLASVSTVLTSK	Y
4285	2	42	A	-0.3	2.79	0	0.366	gi|27574244|pdb|1O1N|A	0	K	FLASVSTVLTSK	Y
4406	2	42	A	0.14	2.61	0	0.395	gi|27574244|pdb|1O1N|A	0	K	FLASVSTVLTSK	Y
4521	2	42	A	-0.1	2.56	0	0.346	gi|27574244|pdb|1O1N|A	0	K	FLASVSTVLTSK	Y
4644	2	42	A	-0.2	2.62	0	0.416	gi|27574244|pdb|1O1N|A	0	K	FLASVSTVLTSK	Y
4653	2	42	A	-0.1	2.57	0	0.424	gi|27574244|pdb|1O1N|A	0	K	FLASVSTVLTSK	Y
2460	2	42	B	3.9	2.97	0	0.287	gi|27574244|pdb|1O1N|A	0	K	FLASVSTVLTSK	Y
2467	2	42	B	3.83	2.54	0	0.323	gi|27574244|pdb|1O1N|A	0	K	FLASVSTVLTSK	Y

Scan	z	Sample ID	Band	PPM	XCorr	Delta correlation	Unique delta correlation	Reference	Redundancy		Peptides	
2762	2	42	B	4.11	2.73	0	0.324	gi|27574244|pdb|1O1N|A	0	K	FLASVSTVLTSK	Y
2881	2	42	B	4.77	2.55	0	0.339	gi|27574244|pdb|1O1N|A	0	K	FLASVSTVLTSK	Y
3012	2	42	B	4.8	2.54	0	0.307	gi|27574244|pdb|1O1N|A	0	K	FLASVSTVLTSK	Y
3020	2	42	B	4.73	2.59	0	0.327	gi|27574244|pdb|1O1N|A	0	K	FLASVSTVLTSK	Y
3486	2	42	B	3.26	3.03	0	0.34	gi|27574244|pdb|1O1N|A	0	K	FLASVSTVLTSK	Y
3489	2	42	B	3	2.92	0	0.395	gi|27574244|pdb|1O1N|A	0	K	FLASVSTVLTSK	Y
3618	2	42	B	2.46	3.29	0	0.359	gi|27574244|pdb|1O1N|A	0	K	FLASVSTVLTSK	Y
3620	2	42	B	3.07	3.45	0	0.322	gi|27574244|pdb|1O1N|A	0	K	FLASVSTVLTSK	Y
3745	2	42	B	4.14	2.67	0	0.383	gi|27574244|pdb|1O1N|A	0	K	FLASVSTVLTSK	Y
3749	2	42	B	-0.3	2.68	0	0.4	gi|27574244|pdb|1O1N|A	0	K	FLASVSTVLTSK	Y
3869	2	42	B	-0.3	2.88	0	0.358	gi|27574244|pdb|1O1N|A	0	K	FLASVSTVLTSK	Y
3874	2	42	B	-0.3	2.75	0	0.358	gi|27574244|pdb|1O1N|A	0	K	FLASVSTVLTSK	Y
4119	2	42	B	0.22	2.55	0	0.384	gi|27574244|pdb|1O1N|A	0	K	FLASVSTVLTSK	Y
4368	2	42	B	-0.2	2.82	0	0.376	gi|27574244|pdb|1O1N|A	0	K	FLASVSTVLTSK	Y
4373	2	42	B	0.02	2.83	0	0.377	gi|27574244|pdb|1O1N|A	0	K	FLASVSTVLTSK	Y
4502	2	42	B	0.02	2.77	0	0.378	gi|27574244|pdb|1O1N|A	0	K	FLASVSTVLTSK	Y
2370	2	42	A	-0.3	3.8	0.111	0.111	gi|534467043|gb|AGU26656.1|	99	K	GTFASLSELHC#DK	L
2271	2	42	B	3.78	2.8	0.172	0.172	gi|344254270|gb|EGW10374.1|	99	K	IGGHGAEYGAEALER	M
4164	2	42	B	0.33	2.72	0.101	0.101	gi|344254270|gb|EGW10374.1|	99	K	IGGHGAEYGAEALER	M
2837	2	42	A	3.87	3.56	0.143	0.143	gi|12833511|dbj|BAB22552.1|	29	K	KVADALANAAGHLDDLPGALSALSDLHAHK	L
4646	3	42	A	-0	3.41	0.552	0.552	gi|122513|sp|P02088.2|HBB1_MOUSE	65	K	KVITAFNDGLNHLDSLK	G
2428	3	42	B	3.84	5.45	0.538	0.538	gi|122513|sp|P02088.2|HBB1_MOUSE	65	K	KVITAFNDGLNHLDSLK	G
2281	2	42	A	1.15	2.54	0.987	0.987	gi|431903465|gb|ELK09417.1|	99	K	LHVDPENFR	L
2290	3	42	A	0.91	3.3	0.809	0.809	gi|431903465|gb|ELK09417.1|	99	K	LHVDPENFR	L
2797	2	42	A	4.16	3.42	0.139	0.139	gi|122513|sp|P02088.2|HBB1_MOUSE	88	R	LLGNMIVIVLGHHLGK	D
2806	2	42	A	4.31	3.13	0.149	0.149	gi|122513|sp|P02088.2|HBB1_MOUSE	88	R	LLGNMIVIVLGHHLGK	D
2754	2	42	B	4.91	3.25	0.272	0.272	gi|122513|sp|P02088.2|HBB1_MOUSE	88	R	LLGNMIVIVLGHHLGK	D
2756	2	42	B	4.67	3.55	0.203	0.203	gi|122513|sp|P02088.2|HBB1_MOUSE	88	R	LLGNMIVIVLGHHLGK	D
4141	2	42	B	1.08	2.92	0.306	0.306	gi|122513|sp|P02088.2|HBB1_MOUSE	88	R	LLGNMIVIVLGHHLGK	D
2321	2	42	A	2.6	2.67	0.493	0.493	gi|27574244|pdb|1O1N|A	99	K	LRVDPVNFK	L
2322	2	42	A	2.98	2.58	0.323	0.323	gi|12833511|dbj|BAB22552.1|	31	R	M*FASFPTTK	T
2207	2	42	B	4.29	2.6	0.318	0.318	gi|12833511|dbj|BAB22552.1|	31	R	M*FASFPTTK	T
2212	2	42	B	4.26	2.62	0.328	0.328	gi|12833511|dbj|BAB22552.1|	31	R	M*FASFPTTK	T
2381	2	42	A	3.92	3.53	0.134	0.134	gi|344254270|gb|EGW10374.1|	99	K	TYFPHFDVSHGSAQVK	A
2391	2	42	A	3.83	3.97	0.123	0.123	gi|344254270|gb|EGW10374.1|	99	K	TYFPHFDVSHGSAQVK	A
2392	3	42	A	3.18	3.07	0.126	0.126	gi|344254270|gb|EGW10374.1|	99	K	TYFPHFDVSHGSAQVK	A
2249	2	42	B	4.15	2.78	0.124	0.124	gi|344254270|gb|EGW10374.1|	99	K	TYFPHFDVSHGSAQVK	A
2257	2	42	B	4.97	3.39	0.149	0.149	gi|344254270|gb|EGW10374.1|	99	K	TYFPHFDVSHGSAQVK	A

Scan	z	Sample ID	Band	PPM	XCorr	Delta correlation	Unique delta correlation	Reference	Redundancy		Peptides	
2384	2	42	B	4.68	3.08	0.142	0.142	gi|344254270|gb|EGW10374.1|	99	K	TYFPHFDVSHGSAQVK	A
2781	3	42	A	4.07	7.07	0.885	0.885	gi|159137400|gb|ABW88847.1|	2	K	VADALATAAGHLDDLPGALSALSDLHAHK	L
3014	3	42	A	3.9	6.45	0.911	0.911	gi|159137400|gb|ABW88847.1|	2	K	VADALATAAGHLDDLPGALSALSDLHAHK	L
3018	3	42	A	3.64	7.3	0.926	0.926	gi|159137400|gb|ABW88847.1|	2	K	VADALATAAGHLDDLPGALSALSDLHAHK	L
4372	3	42	A	-0.2	3.8	0.947	0.947	gi|159137400|gb|ABW88847.1|	2	K	VADALATAAGHLDDLPGALSALSDLHAHK	L
4130	3	42	B	1.81	5.05	0.902	0.902	gi|159137400|gb|ABW88847.1|	2	K	VADALATAAGHLDDLPGALSALSDLHAHK	L
4185	3	42	B	0.7	4.26	0.945	0.945	gi|159137400|gb|ABW88847.1|	2	K	VADALATAAGHLDDLPGALSALSDLHAHK	L
4341	3	42	B	-0.3	4.97	0.987	0.987	gi|159137400|gb|ABW88847.1|	2	K	VADALATAAGHLDDLPGALSALSDLHAHK	L
2611	2	42	A	3.12	4.04	0.553	0.553	gi|122513|sp|P02088.2|HBB1_MOUSE	65	K	VITAFNDGLNHLDSLK	G
4289	2	42	A	-0.9	3.67	0.667	0.667	gi|122513|sp|P02088.2|HBB1_MOUSE	65	K	VITAFNDGLNHLDSLK	G
4393	2	42	A	-0	3.15	0.572	0.572	gi|122513|sp|P02088.2|HBB1_MOUSE	65	K	VITAFNDGLNHLDSLK	G
4517	2	42	A	0.12	2.52	0.784	0.784	gi|122513|sp|P02088.2|HBB1_MOUSE	65	K	VITAFNDGLNHLDSLK	G
2578	2	42	B	4.45	2.7	0.776	0.776	gi|122513|sp|P02088.2|HBB1_MOUSE	65	K	VITAFNDGLNHLDSLK	G
3893	2	42	B	-1	3.55	0.672	0.672	gi|122513|sp|P02088.2|HBB1_MOUSE	65	K	VITAFNDGLNHLDSLK	G
3915	2	42	B	0.35	3.76	0.676	0.676	gi|122513|sp|P02088.2|HBB1_MOUSE	65	K	VITAFNDGLNHLDSLK	G
4081	2	42	B	0.4	4.24	0.56	0.56	gi|122513|sp|P02088.2|HBB1_MOUSE	65	K	VITAFNDGLNHLDSLK	G
4086	2	42	B	0.26	4.09	0.617	0.617	gi|122513|sp|P02088.2|HBB1_MOUSE	65	K	VITAFNDGLNHLDSLK	G
4204	2	42	B	0.02	3.32	0.623	0.623	gi|122513|sp|P02088.2|HBB1_MOUSE	65	K	VITAFNDGLNHLDSLK	G
4212	2	42	B	0.07	3.11	0.566	0.566	gi|122513|sp|P02088.2|HBB1_MOUSE	65	K	VITAFNDGLNHLDSLK	G
4327	2	42	B	0.22	2.78	0.665	0.665	gi|122513|sp|P02088.2|HBB1_MOUSE	65	K	VITAFNDGLNHLDSLK	G
1632	2	42	A	-1	3.42	0.524	0.524	gi|241913510|pdb|3HRW|C	2	-	VLSGEDKSNIK	A
1635	2	42	A	-1	3.26	0.557	0.557	gi|241913510|pdb|3HRW|C	2	-	VLSGEDKSNIK	A
1754	2	42	A	0.24	3.52	0.521	0.521	gi|241913510|pdb|3HRW|C	2	-	VLSGEDKSNIK	A
1756	2	42	A	0.17	3.56	0.519	0.519	gi|241913510|pdb|3HRW|C	2	-	VLSGEDKSNIK	A
1873	2	42	A	-0.2	3.39	0.494	0.494	gi|241913510|pdb|3HRW|C	2	-	VLSGEDKSNIK	A
1876	2	42	A	-0.2	3.5	0.528	0.528	gi|241913510|pdb|3HRW|C	2	-	VLSGEDKSNIK	A
2002	2	42	A	0.34	3.61	0.561	0.561	gi|241913510|pdb|3HRW|C	2	-	VLSGEDKSNIK	A
2006	2	42	A	0.31	2.89	0.506	0.506	gi|241913510|pdb|3HRW|C	2	-	VLSGEDKSNIK	A
2126	2	42	A	0.02	3.47	0.54	0.54	gi|241913510|pdb|3HRW|C	2	-	VLSGEDKSNIK	A
2130	2	42	A	0.03	2.99	0.506	0.506	gi|241913510|pdb|3HRW|C	2	-	VLSGEDKSNIK	A
641	2	42	B	-0.7	2.67	0.447	0.447	gi|241913510|pdb|3HRW|C	2	-	VLSGEDKSNIK	A
1798	2	42	B	-1.4	2.97	0.554	0.554	gi|241913510|pdb|3HRW|C	2	-	VLSGEDKSNIK	A
1916	2	42	B	1.58	3.07	0.492	0.492	gi|241913510|pdb|3HRW|C	2	-	VLSGEDKSNIK	A
1923	2	42	B	1.6	3.07	0.48	0.48	gi|241913510|pdb|3HRW|C	2	-	VLSGEDKSNIK	A
2042	2	42	B	0.85	3.48	0.52	0.52	gi|241913510|pdb|3HRW|C	2	-	VLSGEDKSNIK	A
2044	2	42	B	0.87	3.48	0.508	0.508	gi|241913510|pdb|3HRW|C	2	-	VLSGEDKSNIK	A
2251	2	42	A	2.45	3.31	0	0.381	gi|378548370|sp|B3EWD8.1|HBB_TAMHU	0	K	NVADEVGGEALGR^	L
2411	2	42	A	2.42	3.69	0	0.232	gi|378548370|sp|B3EWD8.1|HBB_TAMHU	0	K	NVADEVGGEALGR^	L

Scan	z	Sample ID	Band	PPM	XCorr	Delta correlation	Unique delta correlation	Reference	Redundancy		Peptides	
4586	2	42	A	-0.6	3.98	0	0.269	gi|378548370|sp|B3EWD8.1|HBB_TAMHU	0	K	NVADEVGGEALGR^	L
4711	2	42	A	-0.2	3.74	0	0.235	gi|378548370|sp|B3EWD8.1|HBB_TAMHU	0	K	NVADEVGGEALGR^	L
4827	2	42	A	-0.2	4.04	0	0.211	gi|378548370|sp|B3EWD8.1|HBB_TAMHU	0	K	NVADEVGGEALGR^	L
4829	2	42	A	-0.2	3.76	0	0.158	gi|378548370|sp|B3EWD8.1|HBB_TAMHU	0	K	NVADEVGGEALGR^	L
156	2	42	B	-0.3	3.1	0	0.181	gi|378548370|sp|B3EWD8.1|HBB_TAMHU	0	K	NVADEVGGEALGR^	L
2328	2	42	B	3.76	2.64	0	0.265	gi|378548370|sp|B3EWD8.1|HBB_TAMHU	0	K	NVADEVGGEALGR^	L
3339	2	42	B	2.52	3.45	0	0.326	gi|378548370|sp|B3EWD8.1|HBB_TAMHU	0	K	NVADEVGGEALGR^	L
3492	2	42	B	2.45	4.09	0	0.273	gi|378548370|sp|B3EWD8.1|HBB_TAMHU	0	K	NVADEVGGEALGR^	L
3627	2	42	B	3.26	3.99	0	0.261	gi|378548370|sp|B3EWD8.1|HBB_TAMHU	0	K	NVADEVGGEALGR^	L
3751	2	42	B	0.28	4.05	0	0.271	gi|378548370|sp|B3EWD8.1|HBB_TAMHU	0	K	NVADEVGGEALGR^	L
3873	2	42	B	-0.3	4.27	0	0.29	gi|378548370|sp|B3EWD8.1|HBB_TAMHU	0	K	NVADEVGGEALGR^	L
4244	2	42	B	-0.2	3.67	0	0.27	gi|378548370|sp|B3EWD8.1|HBB_TAMHU	0	K	NVADEVGGEALGR^	L
4358	2	42	B	-0.4	3.83	0	0.299	gi|378548370|sp|B3EWD8.1|HBB_TAMHU	0	K	NVADEVGGEALGR^	L
4366	2	42	B	-0.3	3.99	0	0.284	gi|378548370|sp|B3EWD8.1|HBB_TAMHU	0	K	NVADEVGGEALGR^	L
4604	2	42	B	-0.3	3.77	0	0.212	gi|378548370|sp|B3EWD8.1|HBB_TAMHU	0	K	NVADEVGGEALGR^	L
4729	2	42	B	-0.8	2.71	0	0.225	gi|378548370|sp|B3EWD8.1|HBB_TAMHU	0	K	NVADEVGGEALGR^	L
4732	2	42	B	-0.8	3.11	0	0.224	gi|378548370|sp|B3EWD8.1|HBB_TAMHU	0	K	NVADEVGGEALGR^	L
2269	2	42	A	3.94	2.8	0.884	0.884	gi|12847007|dbj|BAB27399.1|	60	K	VVAGVAAALAHK	Y
2280	2	42	A	3.93	3.12	0.871	0.871	gi|12847007|dbj|BAB27399.1|	60	K	VVAGVAAALAHK	Y
2407	2	42	A	1.49	3.28	0.903	0.903	gi|12847007|dbj|BAB27399.1|	60	K	VVAGVAAALAHK	Y
2414	2	42	A	1.11	3.18	0.884	0.884	gi|12847007|dbj|BAB27399.1|	60	K	VVAGVAAALAHK	Y
3902	2	42	A	-0.3	2.96	0.885	0.885	gi|12847007|dbj|BAB27399.1|	60	K	VVAGVAAALAHK	Y
4011	2	42	A	-0.2	3.24	0.946	0.946	gi|12847007|dbj|BAB27399.1|	60	K	VVAGVAAALAHK	Y
4053	2	42	A	-1.1	3.1	0.898	0.898	gi|12847007|dbj|BAB27399.1|	60	K	VVAGVAAALAHK	Y
4085	2	42	A	-0.3	3.31	0.909	0.909	gi|12847007|dbj|BAB27399.1|	60	K	VVAGVAAALAHK	Y
4092	2	42	A	-1	3.27	0.897	0.897	gi|12847007|dbj|BAB27399.1|	60	K	VVAGVAAALAHK	Y
4211	2	42	A	0.56	3.2	0.947	0.947	gi|12847007|dbj|BAB27399.1|	60	K	VVAGVAAALAHK	Y
4218	2	42	A	-0.1	3.17	0.893	0.893	gi|12847007|dbj|BAB27399.1|	60	K	VVAGVAAALAHK	Y
4334	2	42	A	0.05	3.28	0.906	0.906	gi|12847007|dbj|BAB27399.1|	60	K	VVAGVAAALAHK	Y
4348	2	42	A	0.09	3.3	0.947	0.947	gi|12847007|dbj|BAB27399.1|	60	K	VVAGVAAALAHK	Y
4458	2	42	A	-0.1	2.87	0.911	0.911	gi|12847007|dbj|BAB27399.1|	60	K	VVAGVAAALAHK	Y
4479	2	42	A	-0.2	2.88	0.941	0.941	gi|12847007|dbj|BAB27399.1|	60	K	VVAGVAAALAHK	Y
4600	2	42	A	-1.6	3.45	0.896	0.896	gi|12847007|dbj|BAB27399.1|	60	K	VVAGVAAALAHK	Y
4605	2	42	A	-1.3	3.43	0.938	0.938	gi|12847007|dbj|BAB27399.1|	60	K	VVAGVAAALAHK	Y
4715	2	42	A	-0.4	3.22	0.975	0.975	gi|12847007|dbj|BAB27399.1|	60	K	VVAGVAAALAHK	Y
4721	2	42	A	-0.4	3.3	0.971	0.971	gi|12847007|dbj|BAB27399.1|	60	K	VVAGVAAALAHK	Y
2275	2	42	B	3.23	3.74	0.902	0.902	gi|12847007|dbj|BAB27399.1|	60	K	VVAGVAAALAHK	Y
2279	2	42	B	3.42	2.96	0.946	0.946	gi|12847007|dbj|BAB27399.1|	60	K	VVAGVAAALAHK	Y

Scan	z	Sample ID	Band	PPM	XCorr	Delta correlation	Unique delta correlation	Reference	Redundancy		Peptides	
2411	2	42	B	4.15	3.62	0.864	0.864	gi|12847007|dbj|BAB27399.1|	60	K	VVAGVAAALAHK	Y
3707	2	42	B	2.36	2.95	0.93	0.93	gi|12847007|dbj|BAB27399.1|	60	K	VVAGVAAALAHK	Y
3716	2	42	B	2.06	3.42	0.902	0.902	gi|12847007|dbj|BAB27399.1|	60	K	VVAGVAAALAHK	Y
3841	2	42	B	-0.6	3.34	0.926	0.926	gi|12847007|dbj|BAB27399.1|	60	K	VVAGVAAALAHK	Y
3846	2	42	B	-0.4	3.36	0.911	0.911	gi|12847007|dbj|BAB27399.1|	60	K	VVAGVAAALAHK	Y
3963	2	42	B	-0.3	3.22	0.893	0.893	gi|12847007|dbj|BAB27399.1|	60	K	VVAGVAAALAHK	Y
3969	2	42	B	-1	3.26	0.941	0.941	gi|12847007|dbj|BAB27399.1|	60	K	VVAGVAAALAHK	Y
4085	2	42	B	0.09	3.47	0.919	0.919	gi|12847007|dbj|BAB27399.1|	60	K	VVAGVAAALAHK	Y
4093	2	42	B	0.16	3.7	0.886	0.886	gi|12847007|dbj|BAB27399.1|	60	K	VVAGVAAALAHK	Y
4201	2	42	B	0.07	3.22	0.905	0.905	gi|12847007|dbj|BAB27399.1|	60	K	VVAGVAAALAHK	Y
4210	2	42	B	0.09	3.34	0.91	0.91	gi|12847007|dbj|BAB27399.1|	60	K	VVAGVAAALAHK	Y
4331	2	42	B	-0.3	2.81	0.952	0.952	gi|12847007|dbj|BAB27399.1|	60	K	VVAGVAAALAHK	Y
4447	2	42	B	-0.1	3.5	0.859	0.859	gi|12847007|dbj|BAB27399.1|	60	K	VVAGVAAALAHK	Y
4449	2	42	B	-0.1	3.18	0.941	0.941	gi|12847007|dbj|BAB27399.1|	60	K	VVAGVAAALAHK	Y
4559	2	42	B	-0.5	3.12	0.935	0.935	gi|12847007|dbj|BAB27399.1|	60	K	VVAGVAAALAHK	Y
4562	2	42	B	-0.5	3.25	0.889	0.889	gi|12847007|dbj|BAB27399.1|	60	K	VVAGVAAALAHK	Y
2309	2	42	A	3.49	4.69	0.924	0.924	gi|12847007|dbj|BAB27399.1|	60	K	VVAGVAAALAHKYH	-
2320	2	42	A	4.09	4.64	0.914	0.914	gi|12847007|dbj|BAB27399.1|	60	K	VVAGVAAALAHKYH	-
4673	2	42	A	-0.4	2.74	0.896	0.896	gi|12847007|dbj|BAB27399.1|	60	K	VVAGVAAALAHKYH	-
4834	2	42	A	-0.5	2.67	0.882	0.882	gi|12847007|dbj|BAB27399.1|	60	K	VVAGVAAALAHKYH	-
2317	2	42	B	4.39	4.13	0.946	0.946	gi|12847007|dbj|BAB27399.1|	60	K	VVAGVAAALAHKYH	-
2320	2	42	B	4.4	3.8	0.919	0.919	gi|12847007|dbj|BAB27399.1|	60	K	VVAGVAAALAHKYH	-
2441	2	42	B	4.81	3.86	0.905	0.905	gi|12847007|dbj|BAB27399.1|	60	K	VVAGVAAALAHKYH	-
3901	2	42	B	-0.7	3.32	0.938	0.938	gi|12847007|dbj|BAB27399.1|	60	K	VVAGVAAALAHKYH	-
3905	2	42	B	-0.4	3.19	0.952	0.952	gi|12847007|dbj|BAB27399.1|	60	K	VVAGVAAALAHKYH	-
4038	2	42	B	0.09	3.44	0.947	0.947	gi|12847007|dbj|BAB27399.1|	60	K	VVAGVAAALAHKYH	-
4050	2	42	B	-0.3	3.24	0.955	0.955	gi|12847007|dbj|BAB27399.1|	60	K	VVAGVAAALAHKYH	-
4160	2	42	B	-0	3.09	0.941	0.941	gi|12847007|dbj|BAB27399.1|	60	K	VVAGVAAALAHKYH	-
4168	2	42	B	0.2	3.34	0.94	0.94	gi|12847007|dbj|BAB27399.1|	60	K	VVAGVAAALAHKYH	-
4354	2	42	B	-0.1	2.96	0.914	0.914	gi|12847007|dbj|BAB27399.1|	60	K	VVAGVAAALAHKYH	-
4362	2	42	B	-0.1	2.8	0.899	0.899	gi|12847007|dbj|BAB27399.1|	60	K	VVAGVAAALAHKYH	-
4475	2	42	B	0.11	3.36	0.942	0.942	gi|12847007|dbj|BAB27399.1|	60	K	VVAGVAAALAHKYH	-
4484	2	42	B	0.11	3.31	0.94	0.94	gi|12847007|dbj|BAB27399.1|	60	K	VVAGVAAALAHKYH	-
4595	2	42	B	-0.4	2.58	0.956	0.956	gi|12847007|dbj|BAB27399.1|	60	K	VVAGVAAALAHKYH	-
2666	2	42	A	3.12	5.54	0.611	0.611	gi|187369324|dbj|BAG31393.1|	73	R	YFDSFGDLSSASAIM*GNAK	V
2675	2	42	A	3.12	6.85	0.45	0.45	gi|187369324|dbj|BAG31393.1|	73	R	YFDSFGDLSSASAIM*GNAK	V
2676	3	42	A	1.37	6.3	0.466	0.466	gi|187369324|dbj|BAG31393.1|	73	R	YFDSFGDLSSASAIM*GNAK	V
2937	2	42	A	4.72	3.94	0.484	0.484	gi|187369324|dbj|BAG31393.1|	73	R	YFDSFGDLSSASAIM*GNAK	V

Scan	z	Sample ID	Band	PPM	XCorr	Delta correlation	Unique delta correlation	Reference	Redundancy		Peptides	
2942	2	42	A	4.69	4.47	0.567	0.567	gi|187369324|dbj|BAG31393.1|	73	R	YFDSFGDLSSASAIM*GNAK	V
4023	3	42	A	0.26	3.26	0.433	0.433	gi|187369324|dbj|BAG31393.1|	73	R	YFDSFGDLSSASAIM*GNAK	V
4291	3	42	A	-1.1	3.1	0.293	0.293	gi|187369324|dbj|BAG31393.1|	73	R	YFDSFGDLSSASAIM*GNAK	V
4363	2	42	A	-0.3	3.64	0.593	0.593	gi|187369324|dbj|BAG31393.1|	73	R	YFDSFGDLSSASAIM*GNAK	V
4386	2	42	A	-0.3	3.59	0.607	0.607	gi|187369324|dbj|BAG31393.1|	73	R	YFDSFGDLSSASAIM*GNAK	V
4535	2	42	A	-2.1	2.62	0.742	0.742	gi|187369324|dbj|BAG31393.1|	73	R	YFDSFGDLSSASAIM*GNAK	V
4679	2	42	A	0.71	3.02	0.665	0.665	gi|187369324|dbj|BAG31393.1|	73	R	YFDSFGDLSSASAIM*GNAK	V
4687	2	42	A	0.67	2.85	0.644	0.644	gi|187369324|dbj|BAG31393.1|	73	R	YFDSFGDLSSASAIM*GNAK	V
2748	2	42	B	4.28	2.6	0.764	0.764	gi|187369324|dbj|BAG31393.1|	73	R	YFDSFGDLSSASAIM*GNAK	V
2751	2	42	B	3.91	2.54	0.663	0.663	gi|187369324|dbj|BAG31393.1|	73	R	YFDSFGDLSSASAIM*GNAK	V
3781	2	42	B	4.22	3.33	0.687	0.687	gi|187369324|dbj|BAG31393.1|	73	R	YFDSFGDLSSASAIM*GNAK	V
3887	2	42	B	-0.3	2.72	0.801	0.801	gi|187369324|dbj|BAG31393.1|	73	R	YFDSFGDLSSASAIM*GNAK	V
4040	3	42	B	-0.3	4.1	0.413	0.413	gi|187369324|dbj|BAG31393.1|	73	R	YFDSFGDLSSASAIM*GNAK	V
4139	2	42	B	-0.8	3.8	0.687	0.687	gi|187369324|dbj|BAG31393.1|	73	R	YFDSFGDLSSASAIM*GNAK	V
4150	2	42	B	0.68	4.13	0.689	0.689	gi|187369324|dbj|BAG31393.1|	73	R	YFDSFGDLSSASAIM*GNAK	V
4152	3	42	B	-0.5	3.18	0.436	0.436	gi|187369324|dbj|BAG31393.1|	73	R	YFDSFGDLSSASAIM*GNAK	V
4270	2	42	B	-0.2	2.85	0.661	0.661	gi|187369324|dbj|BAG31393.1|	73	R	YFDSFGDLSSASAIM*GNAK	V
4283	2	42	B	0.18	3.06	0.635	0.635	gi|187369324|dbj|BAG31393.1|	73	R	YFDSFGDLSSASAIM*GNAK	V
4457	2	42	B	-0.5	2.93	0.66	0.66	gi|187369324|dbj|BAG31393.1|	73	R	YFDSFGDLSSASAIM*GNAK	V
4496	2	42	B	-0.8	3.28	0.655	0.655	gi|187369324|dbj|BAG31393.1|	73	R	YFDSFGDLSSASAIM*GNAK	V
4504	2	42	B	0.36	3.18	0.663	0.663	gi|187369324|dbj|BAG31393.1|	73	R	YFDSFGDLSSASAIM*GNAK	V
2792	3	42	A	2.73	4.58	0.432	0.432	gi|187369324|dbj|BAG31393.1|	73	R	YFDSFGDLSSASAIMGNAK	V
2801	3	42	A	2.82	4.92	0.4	0.4	gi|187369324|dbj|BAG31393.1|	73	R	YFDSFGDLSSASAIMGNAK	V
2910	2	42	A	4.77	2.67	0.715	0.715	gi|187369324|dbj|BAG31393.1|	73	R	YFDSFGDLSSASAIMGNAK	V
2912	2	42	A	4.83	4.56	0.633	0.633	gi|187369324|dbj|BAG31393.1|	73	R	YFDSFGDLSSASAIMGNAK	V
3038	2	42	A	3.64	3.88	0.674	0.674	gi|187369324|dbj|BAG31393.1|	73	R	YFDSFGDLSSASAIMGNAK	V
3046	2	42	A	3.61	3.42	0.659	0.659	gi|187369324|dbj|BAG31393.1|	73	R	YFDSFGDLSSASAIMGNAK	V
3179	2	42	A	3.81	3.85	0.622	0.622	gi|187369324|dbj|BAG31393.1|	73	R	YFDSFGDLSSASAIMGNAK	V
3186	2	42	A	3.91	2.71	0.539	0.539	gi|187369324|dbj|BAG31393.1|	73	R	YFDSFGDLSSASAIMGNAK	V
3596	2	42	A	4.65	2.83	0.707	0.707	gi|187369324|dbj|BAG31393.1|	73	R	YFDSFGDLSSASAIMGNAK	V
3600	2	42	A	4.87	3.18	0.645	0.645	gi|187369324|dbj|BAG31393.1|	73	R	YFDSFGDLSSASAIMGNAK	V
3698	3	42	A	4.43	3.32	0.267	0.267	gi|187369324|dbj|BAG31393.1|	73	R	YFDSFGDLSSASAIMGNAK	V
3733	2	42	A	4.08	3.66	0.634	0.634	gi|187369324|dbj|BAG31393.1|	73	R	YFDSFGDLSSASAIMGNAK	V
3735	2	42	A	4.3	3.97	0.726	0.726	gi|187369324|dbj|BAG31393.1|	73	R	YFDSFGDLSSASAIMGNAK	V
3829	3	42	A	-0.1	3.84	0.327	0.327	gi|187369324|dbj|BAG31393.1|	73	R	YFDSFGDLSSASAIMGNAK	V
3861	2	42	A	0.76	3.86	0.646	0.646	gi|187369324|dbj|BAG31393.1|	73	R	YFDSFGDLSSASAIMGNAK	V
3863	2	42	A	2.53	3.87	0.672	0.672	gi|187369324|dbj|BAG31393.1|	73	R	YFDSFGDLSSASAIMGNAK	V
3955	3	42	A	-1.2	4.57	0.389	0.389	gi|187369324|dbj|BAG31393.1|	73	R	YFDSFGDLSSASAIMGNAK	V

↳

Scan	z	Sample ID	Band	PPM	XCorr	Delta correlation	Unique delta correlation	Reference	Redundancy		Peptides	
3957	3	42	A	-1.1	3.35	0.299	0.299	gi|187369324|dbj|BAG31393.1|	73	R	YFDSFGDLSSASAIMGNAK	V
3985	2	42	A	0.52	4.75	0.61	0.61	gi|187369324|dbj|BAG31393.1|	73	R	YFDSFGDLSSASAIMGNAK	V
3987	2	42	A	0.37	3.9	0.683	0.683	gi|187369324|dbj|BAG31393.1|	73	R	YFDSFGDLSSASAIMGNAK	V
4077	3	42	A	-0.6	3.89	0.31	0.31	gi|187369324|dbj|BAG31393.1|	73	R	YFDSFGDLSSASAIMGNAK	V
4081	3	42	A	-0.4	4.86	0.338	0.338	gi|187369324|dbj|BAG31393.1|	73	R	YFDSFGDLSSASAIMGNAK	V
4106	2	42	A	-0.1	4.62	0.696	0.696	gi|187369324|dbj|BAG31393.1|	73	R	YFDSFGDLSSASAIMGNAK	V
4109	2	42	A	-0.1	5.18	0.62	0.62	gi|187369324|dbj|BAG31393.1|	73	R	YFDSFGDLSSASAIMGNAK	V
4198	3	42	A	-0.3	4.57	0.317	0.317	gi|187369324|dbj|BAG31393.1|	73	R	YFDSFGDLSSASAIMGNAK	V
4200	3	42	A	-0.3	3.14	0.222	0.222	gi|187369324|dbj|BAG31393.1|	73	R	YFDSFGDLSSASAIMGNAK	V
4229	2	42	A	0.42	4.69	0.638	0.638	gi|187369324|dbj|BAG31393.1|	73	R	YFDSFGDLSSASAIMGNAK	V
4233	2	42	A	0.23	5.75	0.528	0.528	gi|187369324|dbj|BAG31393.1|	73	R	YFDSFGDLSSASAIMGNAK	V
4316	3	42	A	-0.3	4.72	0.314	0.314	gi|187369324|dbj|BAG31393.1|	73	R	YFDSFGDLSSASAIMGNAK	V
4324	3	42	A	-0.2	4.81	0.289	0.289	gi|187369324|dbj|BAG31393.1|	73	R	YFDSFGDLSSASAIMGNAK	V
4342	2	42	A	0.19	5.58	0.548	0.548	gi|187369324|dbj|BAG31393.1|	73	R	YFDSFGDLSSASAIMGNAK	V
4352	2	42	A	0.28	6.04	0.562	0.562	gi|187369324|dbj|BAG31393.1|	73	R	YFDSFGDLSSASAIMGNAK	V
4464	2	42	A	0.27	6.15	0.542	0.542	gi|187369324|dbj|BAG31393.1|	73	R	YFDSFGDLSSASAIMGNAK	V
4474	2	42	A	0.24	5.75	0.566	0.566	gi|187369324|dbj|BAG31393.1|	73	R	YFDSFGDLSSASAIMGNAK	V
4585	2	42	A	0.69	4.88	0.624	0.624	gi|187369324|dbj|BAG31393.1|	73	R	YFDSFGDLSSASAIMGNAK	V
4590	2	42	A	3.22	4.99	0.597	0.597	gi|187369324|dbj|BAG31393.1|	73	R	YFDSFGDLSSASAIMGNAK	V
4685	3	42	A	-0.5	3.39	0.353	0.353	gi|187369324|dbj|BAG31393.1|	73	R	YFDSFGDLSSASAIMGNAK	V
4693	3	42	A	-0.4	3.27	0.248	0.248	gi|187369324|dbj|BAG31393.1|	73	R	YFDSFGDLSSASAIMGNAK	V
4706	2	42	A	0.25	5.2	0.649	0.649	gi|187369324|dbj|BAG31393.1|	73	R	YFDSFGDLSSASAIMGNAK	V
4712	2	42	A	0.21	3.73	0.615	0.615	gi|187369324|dbj|BAG31393.1|	73	R	YFDSFGDLSSASAIMGNAK	V
3412	2	42	B	3.93	2.91	0.66	0.66	gi|187369324|dbj|BAG31393.1|	73	R	YFDSFGDLSSASAIMGNAK	V
3544	2	42	B	4.39	3.61	0.644	0.644	gi|187369324|dbj|BAG31393.1|	73	R	YFDSFGDLSSASAIMGNAK	V
3652	3	42	B	1.72	4.66	0.355	0.355	gi|187369324|dbj|BAG31393.1|	73	R	YFDSFGDLSSASAIMGNAK	V
3658	3	42	B	1.25	4.54	0.311	0.311	gi|187369324|dbj|BAG31393.1|	73	R	YFDSFGDLSSASAIMGNAK	V
3671	2	42	B	0.65	4.14	0.654	0.654	gi|187369324|dbj|BAG31393.1|	73	R	YFDSFGDLSSASAIMGNAK	V
3780	3	42	B	-0.7	4.72	0.338	0.338	gi|187369324|dbj|BAG31393.1|	73	R	YFDSFGDLSSASAIMGNAK	V
3783	3	42	B	-0.6	4.65	0.316	0.316	gi|187369324|dbj|BAG31393.1|	73	R	YFDSFGDLSSASAIMGNAK	V
3795	2	42	B	1.43	3.64	0.546	0.546	gi|187369324|dbj|BAG31393.1|	73	R	YFDSFGDLSSASAIMGNAK	V
3797	2	42	B	0.71	5.1	0.617	0.617	gi|187369324|dbj|BAG31393.1|	73	R	YFDSFGDLSSASAIMGNAK	V
3899	3	42	B	-0.4	3.19	0.16	0.16	gi|187369324|dbj|BAG31393.1|	73	R	YFDSFGDLSSASAIMGNAK	V
3904	3	42	B	-0.4	4.68	0.375	0.375	gi|187369324|dbj|BAG31393.1|	73	R	YFDSFGDLSSASAIMGNAK	V
3912	2	42	B	0.33	4.53	0.652	0.652	gi|187369324|dbj|BAG31393.1|	73	R	YFDSFGDLSSASAIMGNAK	V
3914	2	42	B	0.24	4.46	0.647	0.647	gi|187369324|dbj|BAG31393.1|	73	R	YFDSFGDLSSASAIMGNAK	V
4021	3	42	B	-1	5.25	0.416	0.416	gi|187369324|dbj|BAG31393.1|	73	R	YFDSFGDLSSASAIMGNAK	V
4023	3	42	B	-0.6	4.48	0.313	0.313	gi|187369324|dbj|BAG31393.1|	73	R	YFDSFGDLSSASAIMGNAK	V

Scan	z	Sample ID	Band	PPM	XCorr	Delta correlation	Unique delta correlation	Reference	Redundancy		Peptides	
4033	2	42	B	0.22	5.04	0.669	0.669	gi|187369324|dbj|BAG31393.1|	73	R	YFDSFGDLSSASAIMGNAK	V
4035	2	42	B	0.26	4.03	0.687	0.687	gi|187369324|dbj|BAG31393.1|	73	R	YFDSFGDLSSASAIMGNAK	V
4137	3	42	B	0.12	4.19	0.294	0.294	gi|187369324|dbj|BAG31393.1|	73	R	YFDSFGDLSSASAIMGNAK	V
4146	2	42	B	0.8	5.54	0.543	0.543	gi|187369324|dbj|BAG31393.1|	73	R	YFDSFGDLSSASAIMGNAK	V
4148	3	42	B	0.23	3.89	0.265	0.265	gi|187369324|dbj|BAG31393.1|	73	R	YFDSFGDLSSASAIMGNAK	V
4155	2	42	B	0.55	6.04	0.562	0.562	gi|187369324|dbj|BAG31393.1|	73	R	YFDSFGDLSSASAIMGNAK	V
4255	3	42	B	-0.4	4.19	0.32	0.32	gi|187369324|dbj|BAG31393.1|	73	R	YFDSFGDLSSASAIMGNAK	V
4265	2	42	B	0.4	5.76	0.552	0.552	gi|187369324|dbj|BAG31393.1|	73	R	YFDSFGDLSSASAIMGNAK	V
4267	3	42	B	-0.4	3.37	0.311	0.311	gi|187369324|dbj|BAG31393.1|	73	R	YFDSFGDLSSASAIMGNAK	V
4276	2	42	B	0.43	5.34	0.556	0.556	gi|187369324|dbj|BAG31393.1|	73	R	YFDSFGDLSSASAIMGNAK	V
4375	3	42	B	-0.8	3.55	0.324	0.324	gi|187369324|dbj|BAG31393.1|	73	R	YFDSFGDLSSASAIMGNAK	V
4387	2	42	B	0.48	3.33	0.655	0.655	gi|187369324|dbj|BAG31393.1|	73	R	YFDSFGDLSSASAIMGNAK	V
4391	2	42	B	0.47	3.23	0.666	0.666	gi|187369324|dbj|BAG31393.1|	73	R	YFDSFGDLSSASAIMGNAK	V
4511	2	42	B	0.35	4.72	0.647	0.647	gi|187369324|dbj|BAG31393.1|	73	R	YFDSFGDLSSASAIMGNAK	V
4523	2	42	B	0.34	4.02	0.619	0.619	gi|187369324|dbj|BAG31393.1|	73	R	YFDSFGDLSSASAIMGNAK	V
2270	2	43	A	1.39	3.21	0.917	0.917	gi|554577472|ref|XP_005880462.1|	74	K	AAVSGLWGK	V
2280	2	43	A	1.39	3.12	0.918	0.918	gi|554577472|ref|XP_005880462.1|	74	K	AAVSGLWGK	V
2282	2	43	A	1.17	3.81	0.962	0.962	gi|532054982|ref|XP_005370208.1|	94	K	DFTPAAQAAFQK	V
2291	2	43	A	2.09	4.09	0.955	0.955	gi|532054982|ref|XP_005370208.1|	94	K	DFTPAAQAAFQK	V
2292	3	43	A	1.14	3.07	0.858	0.858	gi|532054982|ref|XP_005370208.1|	94	K	DFTPAAQAAFQK	V
2393	2	43	B	0.87	3.79	0.966	0.966	gi|532054982|ref|XP_005370208.1|	94	K	DFTPAAQAAFQK	V
2403	2	43	B	0.87	3.98	0.957	0.957	gi|532054982|ref|XP_005370208.1|	94	K	DFTPAAQAAFQK	V
2516	2	43	A	0.41	3.86	0	0.335	gi|27574244|pdb|1O1N|A	0	K	FLASVSTVLTSK	Y
2522	2	43	A	4.75	3.59	0	0.347	gi|27574244|pdb|1O1N|A	0	K	FLASVSTVLTSK	Y
2623	2	43	B	-0.4	3.15	0	0.376	gi|27574244|pdb|1O1N|A	0	K	FLASVSTVLTSK	Y
2635	2	43	B	-0.4	3.55	0	0.334	gi|27574244|pdb|1O1N|A	0	K	FLASVSTVLTSK	Y
2475	2	43	A	0.06	6.22	0.547	0.547	gi|122513|sp|P02088.2|HBB1_MOUSE	65	K	KVITAFNDGLNHLDSLK	G
2479	2	43	A	3.57	6.13	0.538	0.538	gi|122513|sp|P02088.2|HBB1_MOUSE	65	K	KVITAFNDGLNHLDSLK	G
2590	3	43	B	0.07	6.24	0.553	0.553	gi|122513|sp|P02088.2|HBB1_MOUSE	65	K	KVITAFNDGLNHLDSLK	G
2593	2	43	B	0.23	5.01	0.589	0.589	gi|122513|sp|P02088.2|HBB1_MOUSE	65	K	KVITAFNDGLNHLDSLK	G
2835	2	43	A	4.2	4.22	0.178	0.178	gi|122513|sp|P02088.2|HBB1_MOUSE	88	R	LLGNMIVIVLGHHLGK	D
2846	2	43	A	-0.3	4.57	0.186	0.186	gi|122513|sp|P02088.2|HBB1_MOUSE	88	R	LLGNMIVIVLGHHLGK	D
2957	2	43	B	-0.3	3.85	0.201	0.201	gi|122513|sp|P02088.2|HBB1_MOUSE	88	R	LLGNMIVIVLGHHLGK	D
2968	2	43	B	3.3	3.73	0.21	0.21	gi|122513|sp|P02088.2|HBB1_MOUSE	88	R	LLGNMIVIVLGHHLGK	D
2248	2	43	A	1.87	2.96	0.348	0.348	gi|12833511|dbj|BAB22552.1|	31	R	M*FASFPTTK	T
2258	2	43	A	1.87	2.86	0.281	0.281	gi|12833511|dbj|BAB22552.1|	31	R	M*FASFPTTK	T
2347	3	43	A	4.21	3.33	0.138	0.138	gi|344254270|gb|EGW10374.1|	99	K	TYFPHFDVSHGSAQVK	A
2348	2	43	A	4.52	4.2	0.112	0.112	gi|344254270|gb|EGW10374.1|	99	K	TYFPHFDVSHGSAQVK	A

Scan	z	Sample ID	Band	PPM	XCorr	Delta correlation	Unique delta correlation	Reference	Redundancy		Peptides	
2358	3	43	A	0.19	3.19	0.144	0.144	gi|344254270|gb|EGW10374.1|	99	K	TYFPHFDVSHGSAQVK	A
2470	2	43	B	2.85	3.72	0.121	0.121	gi|344254270|gb|EGW10374.1|	99	K	TYFPHFDVSHGSAQVK	A
2838	3	43	A	1.69	8.43	0.917	0.917	gi|159137400|gb|ABW88847.1|	2	K	VADALATAAGHLDDLPGALSALSDLHAHK	L
2845	3	43	A	0.34	8.2	0.884	0.884	gi|159137400|gb|ABW88847.1|	2	K	VADALATAAGHLDDLPGALSALSDLHAHK	L
2856	2	43	A	0.51	4.06	0.988	0.988	gi|159137400|gb|ABW88847.1|	2	K	VADALATAAGHLDDLPGALSALSDLHAHK	L
2956	3	43	B	0.41	8.28	0.907	0.907	gi|159137400|gb|ABW88847.1|	2	K	VADALATAAGHLDDLPGALSALSDLHAHK	L
1353	2	43	B	-0.2	2.68	0.169	0.169	gi|378548366|sp|B3EWD6.1|HBB_PERCA	20	-	VHLTDAEK	A
1355	2	43	B	-0.2	2.51	0.182	0.182	gi|378548366|sp|B3EWD6.1|HBB_PERCA	20	-	VHLTDAEK	A
2555	2	43	A	0.24	5.27	0.612	0.612	gi|122513|sp|P02088.2|HBB1_MOUSE	65	K	VITAFNDGLNHLDSLK	G
2566	2	43	A	3.81	4.88	0.606	0.606	gi|122513|sp|P02088.2|HBB1_MOUSE	65	K	VITAFNDGLNHLDSLK	G
2657	2	43	B	0.11	4.73	0.611	0.611	gi|122513|sp|P02088.2|HBB1_MOUSE	65	K	VITAFNDGLNHLDSLK	G
2669	2	43	B	0.11	5.11	0.598	0.598	gi|122513|sp|P02088.2|HBB1_MOUSE	65	K	VITAFNDGLNHLDSLK	G
1447	2	43	A	-0.1	3.35	0.496	0.496	gi|241913510|pdb|3HRW|C	2	-	VLSGEDKSNIK	A
1451	2	43	A	-0.1	3.33	0.521	0.521	gi|241913510|pdb|3HRW|C	2	-	VLSGEDKSNIK	A
1564	2	43	A	-0.7	2.85	0.491	0.491	gi|241913510|pdb|3HRW|C	2	-	VLSGEDKSNIK	A
1567	2	43	A	-0.7	2.89	0.493	0.493	gi|241913510|pdb|3HRW|C	2	-	VLSGEDKSNIK	A
1685	2	43	A	-1	2.65	0.449	0.449	gi|241913510|pdb|3HRW|C	2	-	VLSGEDKSNIK	A
1798	2	43	A	-0.5	2.51	0.524	0.524	gi|241913510|pdb|3HRW|C	2	-	VLSGEDKSNIK	A
1803	2	43	A	-0.4	2.51	0.48	0.48	gi|241913510|pdb|3HRW|C	2	-	VLSGEDKSNIK	A
1295	2	43	B	-0.4	2.52	0.427	0.427	gi|241913510|pdb|3HRW|C	2	-	VLSGEDKSNIK	A
1297	2	43	B	-0.3	2.84	0.49	0.49	gi|241913510|pdb|3HRW|C	2	-	VLSGEDKSNIK	A
1419	2	43	B	-0.2	2.93	0.462	0.462	gi|241913510|pdb|3HRW|C	2	-	VLSGEDKSNIK	A
1422	2	43	B	-0.2	2.93	0.49	0.49	gi|241913510|pdb|3HRW|C	2	-	VLSGEDKSNIK	A
1538	2	43	B	-0.6	2.51	0.462	0.462	gi|241913510|pdb|3HRW|C	2	-	VLSGEDKSNIK	A
2106	2	43	A	-0.9	3.48	0	0.304	gi|378548370|sp|B3EWD8.1|HBB_TAMHU	0	K	NVADEVGGEALGR^	L
2204	2	43	A	2.16	3.22	0.872	0.872	gi|12847007|dbj|BAB27399.1|	60	K	VVAGVAAALAHK	Y
2215	2	43	A	2.21	4.02	0.818	0.818	gi|12847007|dbj|BAB27399.1|	60	K	VVAGVAAALAHK	Y
2327	2	43	B	0.6	3.84	0.888	0.888	gi|12847007|dbj|BAB27399.1|	60	K	VVAGVAAALAHK	Y
2273	2	43	A	0.11	4.54	0.905	0.905	gi|12847007|dbj|BAB27399.1|	60	K	VVAGVAAALAHKYH	-
2281	2	43	A	0.11	4.43	0.906	0.906	gi|12847007|dbj|BAB27399.1|	60	K	VVAGVAAALAHKYH	-
2394	2	43	B	0	3.12	0.958	0.958	gi|12847007|dbj|BAB27399.1|	60	K	VVAGVAAALAHKYH	-
2405	2	43	B	0	3.72	0.94	0.94	gi|12847007|dbj|BAB27399.1|	60	K	VVAGVAAALAHKYH	-
2561	2	43	A	0.26	6.3	0.537	0.537	gi|187369324|dbj|BAG31393.1|	73	R	YFDSFGDLSSASAIM*GNAK	V
2664	2	43	B	-0.5	6.17	0.531	0.531	gi|187369324|dbj|BAG31393.1|	73	R	YFDSFGDLSSASAIM*GNAK	V
2668	2	43	B	-0.5	6.61	0.518	0.518	gi|187369324|dbj|BAG31393.1|	73	R	YFDSFGDLSSASAIM*GNAK	V
2720	2	43	A	2.08	6.74	0.481	0.481	gi|187369324|dbj|BAG31393.1|	73	R	YFDSFGDLSSASAIMGNAK	V
2722	3	43	A	0.88	5.87	0.323	0.323	gi|187369324|dbj|BAG31393.1|	73	R	YFDSFGDLSSASAIMGNAK	V
2731	2	43	A	1.97	6.78	0.546	0.546	gi|187369324|dbj|BAG31393.1|	73	R	YFDSFGDLSSASAIMGNAK	V

Scan	z	Sample ID	Band	PPM	XCorr	Delta correlation	Unique delta correlation	Reference	Redundancy		Peptides	
2732	3	43	A	0.72	5.73	0.378	0.378	gi|187369324|dbj|BAG31393.1|	73	R	YFDSFGDLSSASAIMGNAK	V
2821	2	43	B	1.95	6.3	0.534	0.534	gi|187369324|dbj|BAG31393.1|	73	R	YFDSFGDLSSASAIMGNAK	V
2822	3	43	B	0.77	5.81	0.362	0.362	gi|187369324|dbj|BAG31393.1|	73	R	YFDSFGDLSSASAIMGNAK	V
2832	3	43	B	0.79	5.75	0.381	0.381	gi|187369324|dbj|BAG31393.1|	73	R	YFDSFGDLSSASAIMGNAK	V
2319	2	44	B	1.91	2.54	0.894	0.894	gi|554577472|ref|XP_005880462.1|	74	K	AAVSGLWGK	V
2579	2	44	A	1.64	3.81	0.957	0.957	gi|532054982|ref|XP_005370208.1|	94	K	DFTPAAQAAFQK	V
2590	2	44	A	1.64	3.91	0.953	0.953	gi|532054982|ref|XP_005370208.1|	94	K	DFTPAAQAAFQK	V
2593	3	44	A	0.18	3.03	0.821	0.821	gi|532054982|ref|XP_005370208.1|	94	K	DFTPAAQAAFQK	V
2351	2	44	B	0.97	3.95	0.951	0.951	gi|532054982|ref|XP_005370208.1|	94	K	DFTPAAQAAFQK	V
2818	2	44	A	-0	3.38	0	0.344	gi|27574244|pdb|1O1N|A	0	K	FLASVSTVLTSK	Y
2821	2	44	A	0.06	3.44	0	0.342	gi|27574244|pdb|1O1N|A	0	K	FLASVSTVLTSK	Y
2593	2	44	B	-0.1	3.66	0	0.345	gi|27574244|pdb|1O1N|A	0	K	FLASVSTVLTSK	Y
2772	3	44	A	-0.3	5.34	0.519	0.519	gi|122513|sp|P02088.2|HBB1_MOUSE	65	K	KVITAFNDGLNHLDSLK	G
2779	3	44	A	-0.3	5.68	0.518	0.518	gi|122513|sp|P02088.2|HBB1_MOUSE	65	K	KVITAFNDGLNHLDSLK	G
2781	2	44	A	-0.8	5.35	0.618	0.618	gi|122513|sp|P02088.2|HBB1_MOUSE	65	K	KVITAFNDGLNHLDSLK	G
2552	2	44	B	0.29	4.4	0.627	0.627	gi|122513|sp|P02088.2|HBB1_MOUSE	65	K	KVITAFNDGLNHLDSLK	G
2926	2	44	A	-0.5	3.8	0.22	0.22	gi|122513|sp|P02088.2|HBB1_MOUSE	88	R	LLGNM*IVIVLGHHLGK	D
2732	2	44	B	-0	2.79	0.212	0.212	gi|122513|sp|P02088.2|HBB1_MOUSE	88	R	LLGNM*IVIVLGHHLGK	D
3116	2	44	A	0.19	4.31	0.173	0.173	gi|122513|sp|P02088.2|HBB1_MOUSE	88	R	LLGNMIVIVLGHHLGK	D
2918	2	44	B	3.54	4.46	0.193	0.193	gi|122513|sp|P02088.2|HBB1_MOUSE	88	R	LLGNMIVIVLGHHLGK	D
2927	2	44	B	0.97	4.42	0.183	0.183	gi|122513|sp|P02088.2|HBB1_MOUSE	88	R	LLGNMIVIVLGHHLGK	D
2298	2	44	B	1.94	2.57	0.283	0.283	gi|12833511|dbj|BAB22552.1|	31	R	M*FASFPTTK	T
2657	3	44	A	0.66	3.24	0.141	0.141	gi|344254270|gb|EGW10374.1|	99	K	TYFPHFDVSHGSAQVK	A
2659	2	44	A	1.19	3.65	0.119	0.119	gi|344254270|gb|EGW10374.1|	99	K	TYFPHFDVSHGSAQVK	A
2406	3	44	B	1.86	3.67	0.126	0.126	gi|344254270|gb|EGW10374.1|	99	K	TYFPHFDVSHGSAQVK	A
2407	2	44	B	2.83	3.99	0.102	0.102	gi|344254270|gb|EGW10374.1|	99	K	TYFPHFDVSHGSAQVK	A
2420	2	44	B	-0.2	3.48	0.12	0.12	gi|344254270|gb|EGW10374.1|	99	K	TYFPHFDVSHGSAQVK	A
3151	3	44	A	-0.9	7.45	0.941	0.941	gi|159137400|gb|ABW88847.1|	2	K	VADALATAAGHLDDLPGALSALSDLHAHK	L
3158	3	44	A	2.66	7.2	0.944	0.944	gi|159137400|gb|ABW88847.1|	2	K	VADALATAAGHLDDLPGALSALSDLHAHK	L
2948	3	44	B	2.04	5.77	0.936	0.936	gi|159137400|gb|ABW88847.1|	2	K	VADALATAAGHLDDLPGALSALSDLHAHK	L
1387	2	44	B	0.09	2.61	0.173	0.173	gi|378548366|sp|B3EWD6.1|HBB_PERCA	20	-	VHLTDAEK	A
2855	2	44	A	0.1	3.63	0.676	0.676	gi|122513|sp|P02088.2|HBB1_MOUSE	65	K	VITAFNDGLNHLDSLK	G
2618	2	44	B	0.02	3.66	0.657	0.657	gi|122513|sp|P02088.2|HBB1_MOUSE	65	K	VITAFNDGLNHLDSLK	G
2628	2	44	B	3.85	3.8	0.668	0.668	gi|122513|sp|P02088.2|HBB1_MOUSE	65	K	VITAFNDGLNHLDSLK	G
1414	2	44	B	-0	3.08	0.528	0.528	gi|241913510|pdb|3HRW|C	2	-	VLSGEDKSNIK	A
1417	2	44	B	-0	3.28	0.487	0.487	gi|241913510|pdb|3HRW|C	2	-	VLSGEDKSNIK	A
2497	2	44	A	0.34	3.98	0.857	0.857	gi|12847007|dbj|BAB27399.1|	60	K	VVAGVAAALAHK	Y
2507	2	44	A	-0.3	3.62	0.874	0.874	gi|12847007|dbj|BAB27399.1|	60	K	VVAGVAAALAHK	Y

Scan	z	Sample ID	Band	PPM	XCorr	Delta correlation	Unique delta correlation	Reference	Redundancy		Peptides	
2247	2	44	B	2.99	3.89	0.903	0.903	gi|12847007|dbj|BAB27399.1|	60	K	VVAGVAAALAHK	Y
2253	2	44	B	2.99	3.63	0.891	0.891	gi|12847007|dbj|BAB27399.1|	60	K	VVAGVAAALAHK	Y
2583	2	44	A	-0.5	3.64	0.939	0.939	gi|12847007|dbj|BAB27399.1|	60	K	VVAGVAAALAHKYH	-
2597	2	44	A	-0.5	3.79	0.936	0.936	gi|12847007|dbj|BAB27399.1|	60	K	VVAGVAAALAHKYH	-
2861	2	44	A	0.65	4.72	0.605	0.605	gi|187369324|dbj|BAG31393.1|	73	R	YFDSFGDLSSASAIM*GNAK	V
2643	2	44	B	-0.5	4.57	0.574	0.574	gi|187369324|dbj|BAG31393.1|	73	R	YFDSFGDLSSASAIM*GNAK	V
2648	2	44	B	4.78	5.04	0.561	0.561	gi|187369324|dbj|BAG31393.1|	73	R	YFDSFGDLSSASAIM*GNAK	V
3018	2	44	A	0.85	5.82	0.636	0.636	gi|187369324|dbj|BAG31393.1|	73	R	YFDSFGDLSSASAIMGNAK	V
3033	2	44	A	1.03	4.9	0.617	0.617	gi|187369324|dbj|BAG31393.1|	73	R	YFDSFGDLSSASAIMGNAK	V
2808	2	44	B	1.72	6.65	0.537	0.537	gi|187369324|dbj|BAG31393.1|	73	R	YFDSFGDLSSASAIMGNAK	V
2813	2	44	B	0.58	6.17	0.55	0.55	gi|187369324|dbj|BAG31393.1|	73	R	YFDSFGDLSSASAIMGNAK	V
2828	3	44	B	4.1	3.04	0.254	0.254	gi|187369324|dbj|BAG31393.1|	73	R	YFDSFGDLSSASAIMGNAK	V
2298	2	45	A	1.19	3.38	0.892	0.892	gi|554577472|ref|XP_005880462.1|	74	K	AAVSGLWGK	V
2308	2	45	A	1.19	3.37	0.894	0.894	gi|554577472|ref|XP_005880462.1|	74	K	AAVSGLWGK	V
2147	2	45	B	4.36	3.27	0.924	0.924	gi|554577472|ref|XP_005880462.1|	74	K	AAVSGLWGK	V
3688	2	45	B	-0.6	2.7	0.982	0.982	gi|554577472|ref|XP_005880462.1|	74	K	AAVSGLWGK	V
3947	2	45	B	-0.2	2.69	0.984	0.984	gi|554577472|ref|XP_005880462.1|	74	K	AAVSGLWGK	V
4059	2	45	B	-0	2.77	0.983	0.983	gi|554577472|ref|XP_005880462.1|	74	K	AAVSGLWGK	V
2340	2	45	A	2.15	4.03	0.958	0.958	gi|532054982|ref|XP_005370208.1|	94	K	DFTPAAQAAFQK	V
2346	2	45	A	3.19	3.96	0.954	0.954	gi|532054982|ref|XP_005370208.1|	94	K	DFTPAAQAAFQK	V
2347	3	45	A	0.88	4.42	0.834	0.834	gi|532054982|ref|XP_005370208.1|	94	K	DFTPAAQAAFQK	V
2357	3	45	A	0.93	4.26	0.824	0.824	gi|532054982|ref|XP_005370208.1|	94	K	DFTPAAQAAFQK	V
2202	2	45	B	4.14	4.07	0.954	0.954	gi|532054982|ref|XP_005370208.1|	94	K	DFTPAAQAAFQK	V
2211	2	45	B	4.12	3.45	0.949	0.949	gi|532054982|ref|XP_005370208.1|	94	K	DFTPAAQAAFQK	V
2215	3	45	B	2.44	4.26	0.821	0.821	gi|532054982|ref|XP_005370208.1|	94	K	DFTPAAQAAFQK	V
2585	2	45	A	1.8	3.3	0	0.282	gi|27574244|pdb|1O1N|A	0	K	FLASVSTVLTSK	Y
2596	2	45	A	1.87	3.81	0	0.335	gi|27574244|pdb|1O1N|A	0	K	FLASVSTVLTSK	Y
2694	2	45	B	4.65	2.66	0	0.302	gi|27574244|pdb|1O1N|A	0	K	FLASVSTVLTSK	Y
2701	2	45	B	4.73	2.66	0	0.318	gi|27574244|pdb|1O1N|A	0	K	FLASVSTVLTSK	Y
2953	2	45	B	3.92	2.71	0	0.287	gi|27574244|pdb|1O1N|A	0	K	FLASVSTVLTSK	Y
2959	2	45	B	3.79	2.94	0	0.266	gi|27574244|pdb|1O1N|A	0	K	FLASVSTVLTSK	Y
3100	2	45	B	3.86	2.77	0	0.357	gi|27574244|pdb|1O1N|A	0	K	FLASVSTVLTSK	Y
3460	2	45	B	-0.4	2.55	0	0.411	gi|27574244|pdb|1O1N|A	0	K	FLASVSTVLTSK	Y
3462	2	45	B	-0.6	2.71	0	0.388	gi|27574244|pdb|1O1N|A	0	K	FLASVSTVLTSK	Y
3595	2	45	B	0.06	3.09	0	0.341	gi|27574244|pdb|1O1N|A	0	K	FLASVSTVLTSK	Y
3600	2	45	B	0.06	3.56	0	0.329	gi|27574244|pdb|1O1N|A	0	K	FLASVSTVLTSK	Y
3724	2	45	B	-0.5	3.23	0	0.337	gi|27574244|pdb|1O1N|A	0	K	FLASVSTVLTSK	Y
3726	2	45	B	-0.5	2.71	0	0.37	gi|27574244|pdb|1O1N|A	0	K	FLASVSTVLTSK	Y

Scan	z	Sample ID	Band	PPM	XCorr	Delta correlation	Unique delta correlation	Reference	Redundancy		Peptides	
3847	2	45	B	-0.4	2.68	0	0.38	gi|27574244|pdb|1O1N|A	0	K	FLASVSTVLTSK	Y
3849	2	45	B	-0.4	3.03	0	0.337	gi|27574244|pdb|1O1N|A	0	K	FLASVSTVLTSK	Y
3968	2	45	B	-0.1	2.93	0	0.38	gi|27574244|pdb|1O1N|A	0	K	FLASVSTVLTSK	Y
3971	2	45	B	-0.1	2.77	0	0.372	gi|27574244|pdb|1O1N|A	0	K	FLASVSTVLTSK	Y
4089	2	45	B	-0	2.91	0	0.354	gi|27574244|pdb|1O1N|A	0	K	FLASVSTVLTSK	Y
4094	2	45	B	-0.1	3.02	0	0.351	gi|27574244|pdb|1O1N|A	0	K	FLASVSTVLTSK	Y
4206	2	45	B	0.03	2.83	0	0.343	gi|27574244|pdb|1O1N|A	0	K	FLASVSTVLTSK	Y
4217	2	45	B	0	2.64	0	0.376	gi|27574244|pdb|1O1N|A	0	K	FLASVSTVLTSK	Y
4326	2	45	B	0.1	2.57	0	0.393	gi|27574244|pdb|1O1N|A	0	K	FLASVSTVLTSK	Y
4443	2	45	B	-0.6	2.72	0	0.367	gi|27574244|pdb|1O1N|A	0	K	FLASVSTVLTSK	Y
4447	2	45	B	-0.5	2.61	0	0.438	gi|27574244|pdb|1O1N|A	0	K	FLASVSTVLTSK	Y
4571	2	45	B	-0.3	2.57	0	0.371	gi|27574244|pdb|1O1N|A	0	K	FLASVSTVLTSK	Y
2370	2	45	A	-1.3	3.3	0.102	0.102	gi|534467043|gb|AGU26656.1|	99	K	GTFASLSELHC#DK	L
2266	2	45	A	3.09	2.55	0.125	0.125	gi|528765669|gb|EPY85328.1|	8	K	IGGHAAEYGAEALER	M
2101	2	45	B	2.81	2.65	0.131	0.131	gi|528765669|gb|EPY85328.1|	8	K	IGGHAAEYGAEALER	M
2106	2	45	B	2.8	2.91	0.183	0.183	gi|528765669|gb|EPY85328.1|	8	K	IGGHAAEYGAEALER	M
2490	3	45	A	2.06	4.42	0.569	0.569	gi|122513|sp|P02088.2|HBB1_MOUSE	65	K	KVITAFNDGLNHLDSLK	G
2543	2	45	A	0.95	5.9	0.528	0.528	gi|122513|sp|P02088.2|HBB1_MOUSE	65	K	KVITAFNDGLNHLDSLK	G
4787	3	45	B	-0.4	5.59	0.511	0.511	gi|122513|sp|P02088.2|HBB1_MOUSE	65	K	KVITAFNDGLNHLDSLK	G
2120	3	45	B	0.65	3.05	0.792	0.792	gi|431903465|gb|ELK09417.1|	99	K	LHVDPENFR	L
2875	2	45	A	0.32	4.41	0.186	0.186	gi|122513|sp|P02088.2|HBB1_MOUSE	88	R	LLGNMIVIVLGHHLGK	D
2880	2	45	A	0.37	4.83	0.172	0.172	gi|122513|sp|P02088.2|HBB1_MOUSE	88	R	LLGNMIVIVLGHHLGK	D
4179	2	45	B	0.95	2.68	0.2	0.2	gi|122513|sp|P02088.2|HBB1_MOUSE	88	R	LLGNMIVIVLGHHLGK	D
2299	2	45	A	-0.2	2.74	0.492	0.492	gi|27574244|pdb|1O1N|A	99	K	LRVDPVNFK	L
2310	2	45	A	-0.2	2.81	0.465	0.465	gi|27574244|pdb|1O1N|A	99	K	LRVDPVNFK	L
2151	2	45	B	3.25	2.57	0.48	0.48	gi|27574244|pdb|1O1N|A	99	K	LRVDPVNFK	L
2287	2	45	A	1.93	2.78	0.325	0.325	gi|12833511|dbj|BAB22552.1|	31	R	M*FASFPTTK	T
2139	2	45	B	3.68	2.6	0.32	0.32	gi|12833511|dbj|BAB22552.1|	31	R	M*FASFPTTK	T
2150	2	45	B	3.68	2.69	0.325	0.325	gi|12833511|dbj|BAB22552.1|	31	R	M*FASFPTTK	T
3677	2	45	B	-0.1	2.63	0.326	0.326	gi|12833511|dbj|BAB22552.1|	31	R	M*FASFPTTK	T
2407	2	45	A	0.42	2.52	0.426	0.426	gi|12833511|dbj|BAB22552.1|	31	R	MFASFPTTK	T
2380	2	45	A	0.49	4.22	0.106	0.106	gi|344254270|gb|EGW10374.1|	99	K	TYFPHFDVSHGSAQVK	A
2387	2	45	A	0.48	3.71	0.12	0.12	gi|344254270|gb|EGW10374.1|	99	K	TYFPHFDVSHGSAQVK	A
2223	2	45	B	4.29	2.75	0.137	0.137	gi|344254270|gb|EGW10374.1|	99	K	TYFPHFDVSHGSAQVK	A
2233	2	45	B	4.2	3.88	0.122	0.122	gi|344254270|gb|EGW10374.1|	99	K	TYFPHFDVSHGSAQVK	A
2901	3	45	A	0.66	8.09	0.912	0.912	gi|159137400|gb|ABW88847.1|	2	K	VADALATAAGHLDDLPGALSALSDLHAHK	L
2906	3	45	A	0.18	8.29	0.901	0.901	gi|159137400|gb|ABW88847.1|	2	K	VADALATAAGHLDDLPGALSALSDLHAHK	L
2930	2	45	A	0.6	3.3	0.923	0.923	gi|159137400|gb|ABW88847.1|	2	K	VADALATAAGHLDDLPGALSALSDLHAHK	L

Scan	z	Sample ID	Band	PPM	XCorr	Delta correlation	Unique delta correlation	Reference	Redundancy		Peptides	
2651	3	45	B	4.7	5.91	0.915	0.915	gi|159137400|gb|ABW88847.1|	2	K	VADALATAAGHLDDLPGALSALSDLHAHK	L
4406	3	45	B	2.57	3.75	0.946	0.946	gi|159137400|gb|ABW88847.1|	2	K	VADALATAAGHLDDLPGALSALSDLHAHK	L
1506	2	45	A	0.65	2.61	0.173	0.173	gi|378548366|sp|B3EWD6.1|HBB_PERCA	20	-	VHLTDAEK	A
1753	2	45	A	-0.1	2.57	0.175	0.175	gi|378548366|sp|B3EWD6.1|HBB_PERCA	20	-	VHLTDAEK	A
1758	2	45	A	-0.1	2.56	0.177	0.177	gi|378548366|sp|B3EWD6.1|HBB_PERCA	20	-	VHLTDAEK	A
1870	2	45	A	-0.1	2.68	0.168	0.168	gi|378548366|sp|B3EWD6.1|HBB_PERCA	20	-	VHLTDAEK	A
1699	2	45	B	0.52	2.5	0.181	0.181	gi|378548366|sp|B3EWD6.1|HBB_PERCA	20	-	VHLTDAEK	A
2546	2	45	A	0.19	4.9	0.674	0.674	gi|122513|sp|P02088.2|HBB1_MOUSE	65	K	VITAFNDGLNHLDSLK	G
2553	2	45	A	1.35	4.38	0.654	0.654	gi|122513|sp|P02088.2|HBB1_MOUSE	65	K	VITAFNDGLNHLDSLK	G
4119	2	45	B	0.4	3.34	0.662	0.662	gi|122513|sp|P02088.2|HBB1_MOUSE	65	K	VITAFNDGLNHLDSLK	G
4142	2	45	B	0.5	2.96	0.648	0.648	gi|122513|sp|P02088.2|HBB1_MOUSE	65	K	VITAFNDGLNHLDSLK	G
4402	2	45	B	-0	2.75	0.761	0.761	gi|122513|sp|P02088.2|HBB1_MOUSE	65	K	VITAFNDGLNHLDSLK	G
4755	2	45	B	0.07	3.4	0.633	0.633	gi|122513|sp|P02088.2|HBB1_MOUSE	65	K	VITAFNDGLNHLDSLK	G
1293	2	45	A	-0.4	3.55	0.491	0.491	gi|241913510|pdb|3HRW|C	2	-	VLSGEDKSNIK	A
1296	2	45	A	-0.4	3.45	0.508	0.508	gi|241913510|pdb|3HRW|C	2	-	VLSGEDKSNIK	A
1414	2	45	A	1.34	3.36	0.509	0.509	gi|241913510|pdb|3HRW|C	2	-	VLSGEDKSNIK	A
1417	2	45	A	1.3	3.08	0.528	0.528	gi|241913510|pdb|3HRW|C	2	-	VLSGEDKSNIK	A
1537	2	45	A	0.53	3.04	0.479	0.479	gi|241913510|pdb|3HRW|C	2	-	VLSGEDKSNIK	A
1539	2	45	A	0.48	2.98	0.519	0.519	gi|241913510|pdb|3HRW|C	2	-	VLSGEDKSNIK	A
1652	2	45	A	-0	3.05	0.47	0.47	gi|241913510|pdb|3HRW|C	2	-	VLSGEDKSNIK	A
1655	2	45	A	-0	3.03	0.441	0.441	gi|241913510|pdb|3HRW|C	2	-	VLSGEDKSNIK	A
1768	2	45	A	-0.3	3.05	0.483	0.483	gi|241913510|pdb|3HRW|C	2	-	VLSGEDKSNIK	A
1773	2	45	A	-0.3	3.28	0.51	0.51	gi|241913510|pdb|3HRW|C	2	-	VLSGEDKSNIK	A
1890	2	45	A	-0.5	3.32	0.522	0.522	gi|241913510|pdb|3HRW|C	2	-	VLSGEDKSNIK	A
1898	2	45	A	-0.5	3.1	0.505	0.505	gi|241913510|pdb|3HRW|C	2	-	VLSGEDKSNIK	A
1512	2	45	B	0.24	3.27	0.594	0.594	gi|241913510|pdb|3HRW|C	2	-	VLSGEDKSNIK	A
1516	2	45	B	0.45	3.39	0.522	0.522	gi|241913510|pdb|3HRW|C	2	-	VLSGEDKSNIK	A
1633	2	45	B	-0.1	3.54	0.544	0.544	gi|241913510|pdb|3HRW|C	2	-	VLSGEDKSNIK	A
1641	2	45	B	-0.1	3.45	0.526	0.526	gi|241913510|pdb|3HRW|C	2	-	VLSGEDKSNIK	A
1761	2	45	B	0.13	3.7	0.527	0.527	gi|241913510|pdb|3HRW|C	2	-	VLSGEDKSNIK	A
1766	2	45	B	0.12	3.68	0.542	0.542	gi|241913510|pdb|3HRW|C	2	-	VLSGEDKSNIK	A
1881	2	45	B	-0.1	3.37	0.527	0.527	gi|241913510|pdb|3HRW|C	2	-	VLSGEDKSNIK	A
1886	2	45	B	-0.1	3.42	0.527	0.527	gi|241913510|pdb|3HRW|C	2	-	VLSGEDKSNIK	A
2000	2	45	B	-0	3.11	0.506	0.506	gi|241913510|pdb|3HRW|C	2	-	VLSGEDKSNIK	A
2011	2	45	B	0.04	2.65	0.528	0.528	gi|241913510|pdb|3HRW|C	2	-	VLSGEDKSNIK	A
3684	2	45	A	0.34	3.53	0	0.24	gi|378548370|sp|B3EWD8.1|HBB_TAMHU	0	K	NVADEVGGEALGR^	L
3706	2	45	A	-1.6	2.95	0	0.245	gi|378548370|sp|B3EWD8.1|HBB_TAMHU	0	K	NVADEVGGEALGR^	L
3841	2	45	A	-1.3	2.81	0	0.276	gi|378548370|sp|B3EWD8.1|HBB_TAMHU	0	K	NVADEVGGEALGR^	L

Scan	z	Sample ID	Band	PPM	XCorr	Delta correlation	Unique delta correlation	Reference	Redundancy		Peptides	
3964	2	45	A	1.32	2.9	0	0.155	gi|378548370|sp|B3EWD8.1|HBB_TAMHU	0	K	NVADEVGGEALGR^	L
4226	2	45	A	-0.5	2.7	0	0.267	gi|378548370|sp|B3EWD8.1|HBB_TAMHU	0	K	NVADEVGGEALGR^	L
4629	2	45	A	-0.2	2.79	0	0.159	gi|378548370|sp|B3EWD8.1|HBB_TAMHU	0	K	NVADEVGGEALGR^	L
3489	2	45	B	1.13	3.78	0	0.221	gi|378548370|sp|B3EWD8.1|HBB_TAMHU	0	K	NVADEVGGEALGR^	L
3636	2	45	B	-0.2	3.31	0	0.191	gi|378548370|sp|B3EWD8.1|HBB_TAMHU	0	K	NVADEVGGEALGR^	L
3887	2	45	B	-0.6	3.81	0	0.26	gi|378548370|sp|B3EWD8.1|HBB_TAMHU	0	K	NVADEVGGEALGR^	L
3891	2	45	B	-0.6	3.37	0	0.196	gi|378548370|sp|B3EWD8.1|HBB_TAMHU	0	K	NVADEVGGEALGR^	L
4007	2	45	B	-0.9	3.08	0	0.186	gi|378548370|sp|B3EWD8.1|HBB_TAMHU	0	K	NVADEVGGEALGR^	L
4127	2	45	B	-0.4	3.61	0	0.192	gi|378548370|sp|B3EWD8.1|HBB_TAMHU	0	K	NVADEVGGEALGR^	L
4137	2	45	B	-0.5	4.39	0	0.291	gi|378548370|sp|B3EWD8.1|HBB_TAMHU	0	K	NVADEVGGEALGR^	L
4244	2	45	B	-0.1	3.36	0	0.235	gi|378548370|sp|B3EWD8.1|HBB_TAMHU	0	K	NVADEVGGEALGR^	L
4250	2	45	B	-0.1	3.48	0	0.274	gi|378548370|sp|B3EWD8.1|HBB_TAMHU	0	K	NVADEVGGEALGR^	L
4371	2	45	B	-0.1	3.47	0	0.282	gi|378548370|sp|B3EWD8.1|HBB_TAMHU	0	K	NVADEVGGEALGR^	L
4383	2	45	B	-0.3	2.96	0	0.147	gi|378548370|sp|B3EWD8.1|HBB_TAMHU	0	K	NVADEVGGEALGR^	L
4495	2	45	B	-0.2	3.59	0	0.21	gi|378548370|sp|B3EWD8.1|HBB_TAMHU	0	K	NVADEVGGEALGR^	L
4500	2	45	B	-0.2	3.7	0	0.207	gi|378548370|sp|B3EWD8.1|HBB_TAMHU	0	K	NVADEVGGEALGR^	L
4617	2	45	B	-0.4	3.23	0	0.173	gi|378548370|sp|B3EWD8.1|HBB_TAMHU	0	K	NVADEVGGEALGR^	L
4619	2	45	B	-0.4	3.27	0	0.24	gi|378548370|sp|B3EWD8.1|HBB_TAMHU	0	K	NVADEVGGEALGR^	L
4736	2	45	B	-0.4	3.17	0	0.159	gi|378548370|sp|B3EWD8.1|HBB_TAMHU	0	K	NVADEVGGEALGR^	L
4738	2	45	B	-0.4	3.04	0	0.155	gi|378548370|sp|B3EWD8.1|HBB_TAMHU	0	K	NVADEVGGEALGR^	L
2145	2	45	B	2.2	2.61	0.928	0.928	gi|17985949|ref|NP_150237.1|	7	K	VNPDDVGGEALGR	L
2278	2	45	A	3.13	3.63	0.148	0.148	gi|667267074|ref|XP_008569536.1|	4	K	VNVDDVGGEALGR	L
2152	2	45	B	3.31	4	0.163	0.163	gi|667267074|ref|XP_008569536.1|	4	K	VNVDDVGGEALGR	L
2244	2	45	A	2.5	3	0.846	0.846	gi|12847007|dbj|BAB27399.1|	60	K	VVAGVAAALAHK	Y
2255	2	45	A	2.5	3.78	0.879	0.879	gi|12847007|dbj|BAB27399.1|	60	K	VVAGVAAALAHK	Y
2093	2	45	B	4.08	2.96	0.897	0.897	gi|12847007|dbj|BAB27399.1|	60	K	VVAGVAAALAHK	Y
2225	2	45	B	2.68	2.96	0.833	0.833	gi|12847007|dbj|BAB27399.1|	60	K	VVAGVAAALAHK	Y
2235	2	45	B	2.65	3.37	0.846	0.846	gi|12847007|dbj|BAB27399.1|	60	K	VVAGVAAALAHK	Y
2392	2	45	B	0.44	3.75	0.864	0.864	gi|12847007|dbj|BAB27399.1|	60	K	VVAGVAAALAHK	Y
3628	2	45	B	-1.9	3.39	0.849	0.849	gi|12847007|dbj|BAB27399.1|	60	K	VVAGVAAALAHK	Y
3638	2	45	B	0.49	3.09	0.934	0.934	gi|12847007|dbj|BAB27399.1|	60	K	VVAGVAAALAHK	Y
3776	2	45	B	0.06	3.23	0.907	0.907	gi|12847007|dbj|BAB27399.1|	60	K	VVAGVAAALAHK	Y
3783	2	45	B	0.14	3.13	0.892	0.892	gi|12847007|dbj|BAB27399.1|	60	K	VVAGVAAALAHK	Y
3901	2	45	B	-0.7	3.31	0.912	0.912	gi|12847007|dbj|BAB27399.1|	60	K	VVAGVAAALAHK	Y
3903	2	45	B	-0.8	3.09	0.902	0.902	gi|12847007|dbj|BAB27399.1|	60	K	VVAGVAAALAHK	Y
4015	2	45	B	-0.4	3.43	0.913	0.913	gi|12847007|dbj|BAB27399.1|	60	K	VVAGVAAALAHK	Y
4017	2	45	B	-0.4	3.43	0.895	0.895	gi|12847007|dbj|BAB27399.1|	60	K	VVAGVAAALAHK	Y
4136	2	45	B	-0.2	3.53	0.84	0.84	gi|12847007|dbj|BAB27399.1|	60	K	VVAGVAAALAHK	Y

Scan	z	Sample ID	Band	PPM	XCorr	Delta correlation	Unique delta correlation	Reference	Redundancy		Peptides	
4146	2	45	B	-0.1	3.69	0.903	0.903	gi|12847007|dbj|BAB27399.1|	60	K	VVAGVAAALAHK	Y
4267	2	45	B	-0.2	3.03	0.92	0.92	gi|12847007|dbj|BAB27399.1|	60	K	VVAGVAAALAHK	Y
4271	2	45	B	-0.1	2.78	0.938	0.938	gi|12847007|dbj|BAB27399.1|	60	K	VVAGVAAALAHK	Y
4397	2	45	B	0.74	3.05	0.888	0.888	gi|12847007|dbj|BAB27399.1|	60	K	VVAGVAAALAHK	Y
4506	2	45	B	-0.2	3.39	0.886	0.886	gi|12847007|dbj|BAB27399.1|	60	K	VVAGVAAALAHK	Y
4510	2	45	B	-0.2	3.14	0.915	0.915	gi|12847007|dbj|BAB27399.1|	60	K	VVAGVAAALAHK	Y
4628	2	45	B	-0.6	3.44	0.924	0.924	gi|12847007|dbj|BAB27399.1|	60	K	VVAGVAAALAHK	Y
4631	2	45	B	-0.6	3.23	0.897	0.897	gi|12847007|dbj|BAB27399.1|	60	K	VVAGVAAALAHK	Y
2302	2	45	A	0.26	4.95	0.91	0.91	gi|12847007|dbj|BAB27399.1|	60	K	VVAGVAAALAHKYH	-
2311	2	45	A	0.26	4.82	0.922	0.922	gi|12847007|dbj|BAB27399.1|	60	K	VVAGVAAALAHKYH	-
2165	2	45	B	3.38	3.53	0.843	0.843	gi|12847007|dbj|BAB27399.1|	60	K	VVAGVAAALAHKYH	-
2169	2	45	B	3.38	4.31	0.888	0.888	gi|12847007|dbj|BAB27399.1|	60	K	VVAGVAAALAHKYH	-
2618	2	45	A	2.21	6.41	0.468	0.468	gi|187369324|dbj|BAG31393.1|	73	R	YFDSFGDLSSASAIM*GNAK	V
2620	3	45	A	0.43	6.98	0.444	0.444	gi|187369324|dbj|BAG31393.1|	73	R	YFDSFGDLSSASAIM*GNAK	V
2629	3	45	A	0.42	6.71	0.429	0.429	gi|187369324|dbj|BAG31393.1|	73	R	YFDSFGDLSSASAIM*GNAK	V
2756	2	45	A	4.54	5.24	0.544	0.544	gi|187369324|dbj|BAG31393.1|	73	R	YFDSFGDLSSASAIM*GNAK	V
2772	2	45	A	-0.1	5.77	0.56	0.56	gi|187369324|dbj|BAG31393.1|	73	R	YFDSFGDLSSASAIM*GNAK	V
2927	2	45	A	-0.7	3.09	0.624	0.624	gi|187369324|dbj|BAG31393.1|	73	R	YFDSFGDLSSASAIM*GNAK	V
2983	2	45	A	-0.6	4.62	0.634	0.634	gi|187369324|dbj|BAG31393.1|	73	R	YFDSFGDLSSASAIM*GNAK	V
3702	3	45	A	0.35	3.47	0.429	0.429	gi|187369324|dbj|BAG31393.1|	73	R	YFDSFGDLSSASAIM*GNAK	V
3717	3	45	A	-0.6	3.5	0.5	0.5	gi|187369324|dbj|BAG31393.1|	73	R	YFDSFGDLSSASAIM*GNAK	V
3920	3	45	A	0.93	3.48	0.374	0.374	gi|187369324|dbj|BAG31393.1|	73	R	YFDSFGDLSSASAIM*GNAK	V
2487	3	45	B	4.22	3.3	0.285	0.285	gi|187369324|dbj|BAG31393.1|	73	R	YFDSFGDLSSASAIM*GNAK	V
2492	3	45	B	4.16	3.34	0.291	0.291	gi|187369324|dbj|BAG31393.1|	73	R	YFDSFGDLSSASAIM*GNAK	V
3572	3	45	B	0.09	4.11	0.485	0.485	gi|187369324|dbj|BAG31393.1|	73	R	YFDSFGDLSSASAIM*GNAK	V
3651	2	45	B	-0.1	3.62	0.682	0.682	gi|187369324|dbj|BAG31393.1|	73	R	YFDSFGDLSSASAIM*GNAK	V
3665	2	45	B	0.76	3.45	0.654	0.654	gi|187369324|dbj|BAG31393.1|	73	R	YFDSFGDLSSASAIM*GNAK	V
3666	3	45	B	-0.2	5.14	0.458	0.458	gi|187369324|dbj|BAG31393.1|	73	R	YFDSFGDLSSASAIM*GNAK	V
3795	2	45	B	-0	2.7	0.689	0.689	gi|187369324|dbj|BAG31393.1|	73	R	YFDSFGDLSSASAIM*GNAK	V
3798	3	45	B	-0.2	4.09	0.463	0.463	gi|187369324|dbj|BAG31393.1|	73	R	YFDSFGDLSSASAIM*GNAK	V
3807	3	45	B	0.43	3.67	0.388	0.388	gi|187369324|dbj|BAG31393.1|	73	R	YFDSFGDLSSASAIM*GNAK	V
3924	2	45	B	-0	5.03	0.625	0.625	gi|187369324|dbj|BAG31393.1|	73	R	YFDSFGDLSSASAIM*GNAK	V
3929	2	45	B	0.05	4.18	0.59	0.59	gi|187369324|dbj|BAG31393.1|	73	R	YFDSFGDLSSASAIM*GNAK	V
3931	3	45	B	0.03	3.96	0.441	0.441	gi|187369324|dbj|BAG31393.1|	73	R	YFDSFGDLSSASAIM*GNAK	V
3941	3	45	B	0.11	3.75	0.462	0.462	gi|187369324|dbj|BAG31393.1|	73	R	YFDSFGDLSSASAIM*GNAK	V
4042	2	45	B	0.2	3.18	0.633	0.633	gi|187369324|dbj|BAG31393.1|	73	R	YFDSFGDLSSASAIM*GNAK	V
4049	2	45	B	0.64	3.99	0.61	0.61	gi|187369324|dbj|BAG31393.1|	73	R	YFDSFGDLSSASAIM*GNAK	V
4060	3	45	B	-0.5	4.81	0.477	0.477	gi|187369324|dbj|BAG31393.1|	73	R	YFDSFGDLSSASAIM*GNAK	V

Scan	z	Sample ID	Band	PPM	XCorr	Delta correlation	Unique delta correlation	Reference	Redundancy		Peptides	
4067	3	45	B	-0.4	4.46	0.433	0.433	gi|187369324|dbj|BAG31393.1|	73	R	YFDSFGDLSSASAIM*GNAK	V
4168	2	45	B	0.67	4.58	0.564	0.564	gi|187369324|dbj|BAG31393.1|	73	R	YFDSFGDLSSASAIM*GNAK	V
4176	2	45	B	0.73	4.8	0.503	0.503	gi|187369324|dbj|BAG31393.1|	73	R	YFDSFGDLSSASAIM*GNAK	V
4177	3	45	B	-0.1	3.64	0.409	0.409	gi|187369324|dbj|BAG31393.1|	73	R	YFDSFGDLSSASAIM*GNAK	V
4188	3	45	B	-0.3	3.5	0.429	0.429	gi|187369324|dbj|BAG31393.1|	73	R	YFDSFGDLSSASAIM*GNAK	V
4405	2	45	B	2.47	4.33	0.663	0.663	gi|187369324|dbj|BAG31393.1|	73	R	YFDSFGDLSSASAIM*GNAK	V
4408	2	45	B	0.61	4.06	0.661	0.661	gi|187369324|dbj|BAG31393.1|	73	R	YFDSFGDLSSASAIM*GNAK	V
4432	3	45	B	-0.5	4.1	0.432	0.432	gi|187369324|dbj|BAG31393.1|	73	R	YFDSFGDLSSASAIM*GNAK	V
4438	3	45	B	-0.7	3.35	0.324	0.324	gi|187369324|dbj|BAG31393.1|	73	R	YFDSFGDLSSASAIM*GNAK	V
4524	2	45	B	0.26	3.85	0.572	0.572	gi|187369324|dbj|BAG31393.1|	73	R	YFDSFGDLSSASAIM*GNAK	V
4526	2	45	B	0.28	4.63	0.683	0.683	gi|187369324|dbj|BAG31393.1|	73	R	YFDSFGDLSSASAIM*GNAK	V
4554	3	45	B	-0.8	3.2	0.399	0.399	gi|187369324|dbj|BAG31393.1|	73	R	YFDSFGDLSSASAIM*GNAK	V
4557	3	45	B	-0.9	3.52	0.465	0.465	gi|187369324|dbj|BAG31393.1|	73	R	YFDSFGDLSSASAIM*GNAK	V
4819	2	45	B	-0.9	3.66	0.656	0.656	gi|187369324|dbj|BAG31393.1|	73	R	YFDSFGDLSSASAIM*GNAK	V
2778	2	45	A	4.04	5.85	0.594	0.594	gi|187369324|dbj|BAG31393.1|	73	R	YFDSFGDLSSASAIMGNAK	V
2781	3	45	A	1.8	5.62	0.378	0.378	gi|187369324|dbj|BAG31393.1|	73	R	YFDSFGDLSSASAIMGNAK	V
2787	2	45	A	2.46	6.67	0.506	0.506	gi|187369324|dbj|BAG31393.1|	73	R	YFDSFGDLSSASAIMGNAK	V
2788	3	45	A	0.46	6.27	0.356	0.356	gi|187369324|dbj|BAG31393.1|	73	R	YFDSFGDLSSASAIMGNAK	V
2981	2	45	A	2.06	3.63	0.632	0.632	gi|187369324|dbj|BAG31393.1|	73	R	YFDSFGDLSSASAIMGNAK	V
3026	2	45	B	4.21	3.27	0.679	0.679	gi|187369324|dbj|BAG31393.1|	73	R	YFDSFGDLSSASAIMGNAK	V
3510	2	45	B	1.57	3.05	0.584	0.584	gi|187369324|dbj|BAG31393.1|	73	R	YFDSFGDLSSASAIMGNAK	V
3668	2	45	B	-0.2	2.99	0.657	0.657	gi|187369324|dbj|BAG31393.1|	73	R	YFDSFGDLSSASAIMGNAK	V
3686	3	45	B	0.54	3.07	0.285	0.285	gi|187369324|dbj|BAG31393.1|	73	R	YFDSFGDLSSASAIMGNAK	V
3785	2	45	B	-0.2	3	0.617	0.617	gi|187369324|dbj|BAG31393.1|	73	R	YFDSFGDLSSASAIMGNAK	V
3884	3	45	B	-2.1	3.1	0.281	0.281	gi|187369324|dbj|BAG31393.1|	73	R	YFDSFGDLSSASAIMGNAK	V
3906	2	45	B	0.24	4.52	0.672	0.672	gi|187369324|dbj|BAG31393.1|	73	R	YFDSFGDLSSASAIMGNAK	V
3909	2	45	B	-0.2	4.26	0.652	0.652	gi|187369324|dbj|BAG31393.1|	73	R	YFDSFGDLSSASAIMGNAK	V
4027	2	45	B	-0.3	4.72	0.659	0.659	gi|187369324|dbj|BAG31393.1|	73	R	YFDSFGDLSSASAIMGNAK	V
4035	2	45	B	-0.3	4.63	0.612	0.612	gi|187369324|dbj|BAG31393.1|	73	R	YFDSFGDLSSASAIMGNAK	V
4151	2	45	B	0.12	3.69	0.636	0.636	gi|187369324|dbj|BAG31393.1|	73	R	YFDSFGDLSSASAIMGNAK	V
4163	2	45	B	0.41	5.2	0.638	0.638	gi|187369324|dbj|BAG31393.1|	73	R	YFDSFGDLSSASAIMGNAK	V
4268	2	45	B	0.2	2.52	0.713	0.713	gi|187369324|dbj|BAG31393.1|	73	R	YFDSFGDLSSASAIMGNAK	V
4391	2	45	B	-0.6	2.93	0.6	0.6	gi|187369324|dbj|BAG31393.1|	73	R	YFDSFGDLSSASAIMGNAK	V
4399	2	45	B	2.02	3.93	0.634	0.634	gi|187369324|dbj|BAG31393.1|	73	R	YFDSFGDLSSASAIMGNAK	V
4507	2	45	B	-0.6	3.38	0.654	0.654	gi|187369324|dbj|BAG31393.1|	73	R	YFDSFGDLSSASAIMGNAK	V
4512	2	45	B	0.45	3.1	0.665	0.665	gi|187369324|dbj|BAG31393.1|	73	R	YFDSFGDLSSASAIMGNAK	V
2586	2	46	A	3.01	2.74	0	0.389	gi|27574244|pdb|1O1N|A	0	K	FLASVSTVLTSK	Y
3449	2	46	A	0	2.65	0	0.313	gi|378548370|sp|B3EWD8.1|HBB_TAMHU	0	K	NVADEVGGEALGR^	L

Scan	z	Sample ID	Band	PPM	XCorr	Delta correlation	Unique delta correlation	Reference	Redundancy		Peptides	
3568	2	46	A	-0.1	3.1	0	0.187	gi|378548370|sp|B3EWD8.1|HBB_TAMHU	0	K	NVADEVGGEALGR^	L
3571	2	46	A	0.05	3.41	0	0.164	gi|378548370|sp|B3EWD8.1|HBB_TAMHU	0	K	NVADEVGGEALGR^	L
3701	2	46	A	0.1	3.11	0	0.175	gi|378548370|sp|B3EWD8.1|HBB_TAMHU	0	K	NVADEVGGEALGR^	L
3828	2	46	A	-0.3	3.16	0	0.153	gi|378548370|sp|B3EWD8.1|HBB_TAMHU	0	K	NVADEVGGEALGR^	L
3830	2	46	A	-0.3	3.35	0	0.276	gi|378548370|sp|B3EWD8.1|HBB_TAMHU	0	K	NVADEVGGEALGR^	L
3970	2	46	A	-0.4	3.45	0	0.249	gi|378548370|sp|B3EWD8.1|HBB_TAMHU	0	K	NVADEVGGEALGR^	L
4203	2	46	A	-0.1	3.07	0	0.208	gi|378548370|sp|B3EWD8.1|HBB_TAMHU	0	K	NVADEVGGEALGR^	L
4205	2	46	A	-0.1	2.75	0	0.318	gi|378548370|sp|B3EWD8.1|HBB_TAMHU	0	K	NVADEVGGEALGR^	L
4448	2	46	A	-0.6	3.5	0	0.317	gi|378548370|sp|B3EWD8.1|HBB_TAMHU	0	K	NVADEVGGEALGR^	L
4567	2	46	A	-0.2	3.07	0	0.195	gi|378548370|sp|B3EWD8.1|HBB_TAMHU	0	K	NVADEVGGEALGR^	L
4571	2	46	A	-0.2	3.18	0	0.251	gi|378548370|sp|B3EWD8.1|HBB_TAMHU	0	K	NVADEVGGEALGR^	L
4687	2	46	A	-0.3	2.87	0	0.258	gi|378548370|sp|B3EWD8.1|HBB_TAMHU	0	K	NVADEVGGEALGR^	L
3583	2	46	B	0.07	2.66	0	0.264	gi|378548370|sp|B3EWD8.1|HBB_TAMHU	0	K	NVADEVGGEALGR^	L
3837	2	46	B	-0.3	2.96	0	0.26	gi|378548370|sp|B3EWD8.1|HBB_TAMHU	0	K	NVADEVGGEALGR^	L
3956	2	46	B	0.13	3.02	0	0.212	gi|378548370|sp|B3EWD8.1|HBB_TAMHU	0	K	NVADEVGGEALGR^	L
4338	2	46	B	3.43	3.39	0	0.245	gi|378548370|sp|B3EWD8.1|HBB_TAMHU	0	K	NVADEVGGEALGR^	L
4352	2	46	B	1.22	3	0	0.23	gi|378548370|sp|B3EWD8.1|HBB_TAMHU	0	K	NVADEVGGEALGR^	L
4475	2	46	B	-0.3	2.51	0	0.208	gi|378548370|sp|B3EWD8.1|HBB_TAMHU	0	K	NVADEVGGEALGR^	L
4598	2	46	B	-0.3	2.57	0	0.209	gi|378548370|sp|B3EWD8.1|HBB_TAMHU	0	K	NVADEVGGEALGR^	L
2253	2	46	A	0.2	3.46	0.896	0.896	gi|12847007|dbj|BAB27399.1|	60	K	VVAGVAAALAHK	Y
4111	2	46	A	-0.4	2.8	0.872	0.872	gi|12847007|dbj|BAB27399.1|	60	K	VVAGVAAALAHK	Y
4423	2	46	A	-0.2	3.41	0.88	0.88	gi|12847007|dbj|BAB27399.1|	60	K	VVAGVAAALAHK	Y
4430	2	46	A	-0.3	3.2	0.945	0.945	gi|12847007|dbj|BAB27399.1|	60	K	VVAGVAAALAHK	Y
2635	2	46	A	4.03	4.41	0.601	0.601	gi|187369324|dbj|BAG31393.1|	73	R	YFDSFGDLSSASAIM*GNAK	V
2819	2	46	A	-0	5.29	0.648	0.648	gi|187369324|dbj|BAG31393.1|	73	R	YFDSFGDLSSASAIMGNAK	V
2873	2	49	A	-2.3	2.83	0	0.275	gi|378548370|sp|B3EWD8.1|HBB_TAMHU	0	K	NVADEVGGEALGR^	L
2499	2	50	A	0.32	2.81	0	0.359	gi|27574244|pdb|1O1N|A	0	K	FLASVSTVLTSK	Y
2210	2	50	A	0.23	3	0	0.236	gi|378548370|sp|B3EWD8.1|HBB_TAMHU	0	K	NVADEVGGEALGR^	L
2252	2	50	A	-0.7	3.4	0.794	0.794	gi|12847007|dbj|BAB27399.1|	60	K	VVAGVAAALAHK	Y
2511	2	50	B	0.51	5.05	0.587	0.587	gi|187369324|dbj|BAG31393.1|	73	R	YFDSFGDLSSASAIM*GNAK	V
2305	2	51	A	0.07	2.89	0.961	0.961	gi|554577472|ref|XP_005880462.1|	74	K	AAVSGLWGK	V
2216	2	51	A	-0.5	2.86	0	0.187	gi|378548370|sp|B3EWD8.1|HBB_TAMHU	0	K	NVADEVGGEALGR^	L
2248	2	51	A	-0.5	2.94	0.921	0.921	gi|12847007|dbj|BAB27399.1|	60	K	VVAGVAAALAHK	Y
2518	2	51	A	1.08	3.16	0.681	0.681	gi|187369324|dbj|BAG31393.1|	73	R	YFDSFGDLSSASAIM*GNAK	V
4558	2	4	A	-3.8	3.18	0	0.324	pdb|1O1N|A	0	K	FLASVSTVLTSK	Y
4569	2	4	A	-3.6	2.67	0	0.452	pdb|1O1N|A	0	K	FLASVSTVLTSK	Y
4800	3	4	A	-1.3	8.38	0.149	0.149	ABU63212.1	11	K	IADALASAAGHLDDLPGALSALSDLHAHK	L
4415	2	4	A	-1.6	3.47	0.915	0.915	AAB59723.1	13	K	IGGHGAEYVAEALER	M

Scan	z	Sample ID	Band	PPM	XCorr	Delta correlation	Unique delta correlation	Reference	Redundancy		Peptides	
4426	2	4	A	-1.6	2.75	0.866	0.866	AAB59723.1	13	K	IGGHGAEYVAEALER	M
4327	2	4	A	-1.6	2.85	0.836	0.836	pdb|1I3R|D	88	K	KVITAFNEGLK	G
4647	3	4	A	0.6	3.27	0.118	0.118	ABU63177.1	99	R	LLGNM*IVIVLGHHLGK	D
4802	2	4	A	-1.5	4.16	0	0.658	AJQ20761.1	18	K	VADALATAAGHLDDLPGALSALSDLHAHK	L
4508	2	4	A	-0.9	3.14	0.845	0.845	ABU63177.1	99	K	VITAFNDGLNHLDSLK	G
4048	2	4	A	-2.1	3.31	0.553	0.553	JAB00156.1	3	-	VLSGEDKSNIK	A
5040	2	4	B	-1.4	2.66	0.902	0.902	NP_032246.2	99	K	VVAGVAAALAHK	Y
4332	2	4	A	-1.3	3.94	0.894	0.894	NP_032246.2	99	K	VVAGVAAALAHKYH	-
4263	2	4	A	-4.7	3.55	0.688	0.688	XP_011381407.1	99	K	VVAGVATALAHK	Y
4591	2	4	A	-0.9	6.52	0.464	0.464	ABU63177.1	99	R	YFDSFGDLSSASAIM*GNAK	V
4602	2	4	A	-0.9	6.78	0.509	0.509	ABU63177.1	99	R	YFDSFGDLSSASAIM*GNAK	V
4594	2	4	A	-1.6	5.36	0.893	0.893	CAA32225.1	99	R	YFDSFGDLSSASAIM*GNPK	V
4604	2	4	A	-1.6	3.37	0.908	0.908	CAA32225.1	99	R	YFDSFGDLSSASAIM*GNPK	V
4715	2	4	A	-3.6	3.11	0.442	0.442	ABU63177.1	99	R	YFDSFGDLSSASAIMGNAK	V
4999	2	4	B	-0.9	4.65	0.606	0.606	ABU63177.1	99	R	YFDSFGDLSSASAIMGNAK	V
5010	2	4	B	-0.9	4.26	0.624	0.624	ABU63177.1	99	R	YFDSFGDLSSASAIMGNAK	V
5342	2	4	B	-1.3	3.74	0.607	0.607	ABU63177.1	99	R	YFDSFGDLSSASAIMGNAK	V
5353	2	4	B	-1.3	3.19	0.553	0.553	ABU63177.1	99	R	YFDSFGDLSSASAIMGNAK	V
4723	2	4	A	-0.2	6.28	0.6	0.6	CAA32225.1	99	R	YFDSFGDLSSASAIMGNPK	V
4113	2	10	B	-4.6	3.43	0.898	0.898	CAA32225.1	99	K	DFTPAAQAAFQK	V
4114	3	10	B	-0.7	4.22	0.824	0.824	CAA32225.1	99	K	DFTPAAQAAFQK	V
4314	2	10	B	-2.9	3.25	0	0.325	pdb|1O1N|A	0	K	FLASVSTVLTSK	Y
4321	2	10	B	-2.9	3.31	0	0.348	pdb|1O1N|A	0	K	FLASVSTVLTSK	Y
4533	3	10	B	-0	8.43	0.181	0.181	ABU63212.1	11	K	IADALASAAGHLDDLPGALSALSDLHAHK	L
3960	2	10	B	-2.4	4.1	0.116	0.116	EGW10374.1	99	K	IGGHGAEYGAEALER	M
3971	2	10	B	-2.4	4.24	0.106	0.106	EGW10374.1	99	K	IGGHGAEYGAEALER	M
4179	2	10	B	-1.4	3.95	0.904	0.904	AAB59723.1	13	K	IGGHGAEYVAEALER	M
4180	3	10	B	-4.9	3.82	0.842	0.842	AAB59723.1	13	K	IGGHGAEYVAEALER	M
4189	3	10	B	-4.9	3.96	0.864	0.864	AAB59723.1	13	K	IGGHGAEYVAEALER	M
4190	2	10	B	-1.4	3.11	0.876	0.876	AAB59723.1	13	K	IGGHGAEYVAEALER	M
4536	3	10	B	-0.4	6.53	0.149	0.149	ABU63212.1	11	K	KIADALASAAGHLDDLPGALSALSDLHAHK	L
**4535**	3	10	B	-1.2	4.83	0.756	0.756	sp|Q7M2Y4.1|HBA_CHAMP	1	K	KVGDALGNAVAHLDDLPGALSALSDLHAHK	L
**4543**	3	10	B	-1.3	5.39	0.797	0.797	sp|Q7M2Y4.1|HBA_CHAMP	1	K	KVGDALGNAVAHLDDLPGALSALSDLHAHK	L
4242	2	10	B	-1.2	4.82	0.525	0.525	ABU63177.1	99	K	KVITAFNDGLNHLDSLK	G
4244	2	10	B	-1.2	5.61	0.498	0.498	ABU63177.1	99	K	KVITAFNDGLNHLDSLK	G
4061	2	10	B	-1.6	3.54	0.869	0.869	pdb|1I3R|D	88	K	KVITAFNEGLK	G
4062	3	10	B	-0.5	4.49	0.854	0.854	pdb|1I3R|D	88	K	KVITAFNEGLK	G
4029	3	10	B	4.23	3.03	0.713	0.713	ERE78771.1	99	K	LHVDPENFR	L

Scan	z	Sample ID	Band	PPM	XCorr	Delta correlation	Unique delta correlation	Reference	Redundancy		Peptides	
4442	2	10	B	-3.7	2.75	0.186	0.186	ELK09417.1	99	R	LLVVYPWTQR	F
4904	2	10	B	0.16	2.67	0.82	0.82	ACY03378.1	0	R	LPGNM*IVIVLGHHLGK	E
4515	2	10	B	-0.4	3	0.165	0.165	BAB27277.1	33	K	VADALANAAGHLDDLPGALSALSDLHAHK	L
3397	2	10	A	-3	2.52	0.223	0.223	pdb|3HRW|D	26	-	VHLTDAEK	A
3308	2	10	B	-1	2.63	0.176	0.176	pdb|3HRW|D	26	-	VHLTDAEK	A
3440	2	10	B	-0.9	2.52	0.201	0.201	pdb|3HRW|D	26	-	VHLTDAEK	A
3578	2	10	B	-1.5	2.53	0.204	0.204	pdb|3HRW|D	26	-	VHLTDAEK	A
3700	2	10	B	-4.1	2.62	0.188	0.188	pdb|3HRW|D	26	-	VHLTDAEK	A
4273	2	10	B	-0.7	2.63	0.79	0.79	ABU63177.1	99	K	VITAFNDGLNHLDSLK	G
4156	2	10	B	-2.4	2.76	0.552	0.552	pdb|1I3R|D	88	K	VITAFNEGLK	G
3735	2	10	B	-2.2	3.13	0.509	0.509	JAB00156.1	3	-	VLSGEDKSNIK	A
4025	2	10	B	1.93	3.25	0.826	0.826	CAA32225.1	62	K	VNPDEVGGEALGR	L
3916	2	10	B	-3.6	2.61	0.97	0.97	sp|P02088.2|HBB1_MOUSE	57	K	VNSDEVGGEALGR	L
3934	2	10	B	-4.3	4.18	0.915	0.915	sp|P02088.2|HBB1_MOUSE	57	K	VNSDEVGGEALGR	L
4037	2	10	B	-2.3	4.01	0.872	0.872	NP_032246.2	99	K	VVAGVAAALAHK	Y
4083	2	10	B	-2.9	2.81	0.882	0.882	NP_032246.2	99	K	VVAGVAAALAHKYH	-
3990	2	10	B	-1.7	3.62	0.664	0.664	XP_011381407.1	99	K	VVAGVATALAHK	Y
3993	2	10	B	-1.7	3.68	0.708	0.708	XP_011381407.1	99	K	VVAGVATALAHK	Y
4346	2	10	B	-0.2	6.18	0.467	0.467	ABU63177.1	99	R	YFDSFGDLSSASAIM*GNAK	V
4354	2	10	B	-0.2	6.45	0.485	0.485	ABU63177.1	99	R	YFDSFGDLSSASAIM*GNAK	V
4355	3	10	B	-0.2	5.85	0.477	0.477	ABU63177.1	99	R	YFDSFGDLSSASAIM*GNAK	V
4358	2	10	B	-1.3	5.33	0.915	0.915	CAA32225.1	99	R	YFDSFGDLSSASAIM*GNPK	V
4466	2	10	B	-2.6	6.34	0.535	0.535	ABU63177.1	99	R	YFDSFGDLSSASAIMGNAK	V
4475	2	10	B	-2.7	4.35	0.489	0.489	ABU63177.1	99	R	YFDSFGDLSSASAIMGNAK	V
5236	2	10	B	-0.8	2.99	0.664	0.664	ABU63177.1	99	R	YFDSFGDLSSASAIMGNAK	V
4221	2	11	A	-2.7	3.12	0.558	0.558	ABU63177.1	99	K	AAVSGLWGK	V
4216	2	11	B	-1.3	2.71	0.687	0.687	AAL73052.2	0	K	ADDIGADALGR	L
4011	2	11	A	-1.2	3.18	0.677	0.677	AAL73052.2	0	K	ADDIGADALGR	L
4391	2	11	B	-3.4	3.92	0.897	0.897	CAA32225.1	99	K	DFTPAAQAAFQK	V
4406	3	11	B	1.42	4.11	0.794	0.794	CAA32225.1	99	K	DFTPAAQAAFQK	V
4214	3	11	A	0.02	3.71	0.818	0.818	CAA32225.1	99	K	DFTPAAQAAFQK	V
4589	2	11	B	-1.3	2.69	0	0.419	pdb|1O1N|A	0	K	FLASVSTVLTSK	Y
4426	2	11	A	-4.2	2.96	0	0.353	pdb|1O1N|A	0	K	FLASVSTVLTSK	Y
4437	2	11	A	-3.7	2.85	0	0.43	pdb|1O1N|A	0	K	FLASVSTVLTSK	Y
4223	2	11	A	-2	3.73	0.111	0.111	ERE78771.1	99	K	GTFASLSELHC#DK	L
4300	2	11	A	-2.1	3.12	0.102	0.102	ERE78771.1	99	K	GTFASLSELHCDK	L
4844	3	11	B	-1.7	8.34	0.15	0.15	ABU63212.1	11	K	IADALASAAGHLDDLPGALSALSDLHAHK	L
4272	2	11	B	-1.6	4.63	0.117	0.117	EGW10374.1	99	K	IGGHGAEYGAEALER	M

Scan	z	Sample ID	Band	PPM	XCorr	Delta correlation	Unique delta correlation	Reference	Redundancy		Peptides	
4456	2	11	B	-1.4	3.69	0.897	0.897	AAB59723.1	13	K	IGGHGAEYVAEALER	M
4272	2	11	A	-1.9	3.46	0.896	0.896	AAB59723.1	13	K	IGGHGAEYVAEALER	M
4283	2	11	A	-1.9	3.76	0.897	0.897	AAB59723.1	13	K	IGGHGAEYVAEALER	M
4660	3	11	A	-3.9	7.59	0.128	0.128	ABU63212.1	11	K	KIADALASAAGHLDDLPGALSALSDLHAHK	L
4671	3	11	A	-3.9	3.17	0.136	0.136	ABU63212.1	11	K	KIADALASAAGHLDDLPGALSALSDLHAHK	L
4227	3	11	B	-1.6	5.74	0.807	0.807	AQN67653.1	0	K	KVGAHAGEYGAEALER	M
4030	3	11	A	-1.8	4.02	0.879	0.879	AQN67653.1	0	K	KVGAHAGEYGAEALER	M
4519	2	11	B	-0.8	5.22	0.493	0.493	ABU63177.1	99	K	KVITAFNDGLNHLDSLK	G
4349	2	11	A	-2	5.93	0.553	0.553	ABU63177.1	99	K	KVITAFNDGLNHLDSLK	G
4347	2	11	B	-0.9	3.93	0.83	0.83	pdb|1I3R|D	88	K	KVITAFNEGLK	G
4348	3	11	B	-0.3	3.86	0.863	0.863	pdb|1I3R|D	88	K	KVITAFNEGLK	G
4165	2	11	A	-2.4	4.13	0.833	0.833	pdb|1I3R|D	88	K	KVITAFNEGLK	G
4166	3	11	A	-0.7	4.8	0.864	0.864	pdb|1I3R|D	88	K	KVITAFNEGLK	G
4124	3	11	A	-1.6	3.06	0.671	0.671	ERE78771.1	99	K	LHVDPENFR	L
4688	3	11	B	-1.1	3.31	0.13	0.13	ABU63177.1	99	R	LLGNM*IVIVLGHHLGK	D
4525	3	11	A	-3.1	3.12	0.134	0.134	ABU63177.1	99	R	LLGNM*IVIVLGHHLGK	D
4528	2	11	A	-2.7	3.23	0.145	0.145	ABU63177.1	99	R	LLGNM*IVIVLGHHLGK	D
4864	2	11	B	-3.4	3.25	0.193	0.193	ABU63177.1	99	R	LLGNMIVIVLGHHLGK	D
4876	2	11	B	-2.5	3.5	0.181	0.181	ABU63177.1	99	R	LLGNMIVIVLGHHLGK	D
4679	2	11	A	-0.5	4.9	0.184	0.184	ABU63177.1	99	R	LLGNMIVIVLGHHLGK	D
4547	2	11	A	-4.2	2.64	0.188	0.188	ELK09417.1	99	R	LLVVYPWTQR	F
4381	2	11	B	-1.6	2.54	0	0.407	pdb|1O1N|A	0	K	LRVDPVNFK	L
4198	3	11	A	-2.5	3.06	0	0.819	pdb|1O1N|A	0	K	LRVDPVNFK	L
4425	2	11	B	-2.3	3.64	0.123	0.123	EGW10374.1	99	K	TYFPHFDVSHGSAQVK	A
4242	3	11	A	-2.3	3.37	0.111	0.111	EGW10374.1	99	K	TYFPHFDVSHGSAQVK	A
4870	3	11	B	-3.1	3.38	0.79	0.79	sp|Q7M2Y4.1|HBA_CHAMP	1	K	VGDALGNAVAHLDDLPGALSALSDLHAHK	L
4067	2	11	B	-4.2	2.55	0.138	0.138	pdb|3HRW|D	26	-	VHLTDAEK	A
3757	2	11	A	-3.7	2.51	0.226	0.226	pdb|3HRW|D	26	-	VHLTDAEK	A
3880	2	11	A	-3.4	2.56	0.216	0.216	pdb|3HRW|D	26	-	VHLTDAEK	A
4597	2	11	B	-1.5	4.72	0.594	0.594	ABU63177.1	99	K	VITAFNDGLNHLDSLK	G
4379	2	11	A	-1.2	2.52	0.78	0.78	ABU63177.1	99	K	VITAFNDGLNHLDSLK	G
4445	2	11	B	-3.3	2.53	0.506	0.506	pdb|1I3R|D	88	K	VITAFNEGLK	G
4261	2	11	A	-5	2.75	0.498	0.498	pdb|1I3R|D	88	K	VITAFNEGLK	G
3850	2	11	A	-1.6	2.77	0.498	0.498	JAB00156.1	3	-	VLSGEDKSNIK	A
4022	2	11	A	-3.1	3.32	0.893	0.893	sp|P02088.2|HBB1_MOUSE	57	K	VNSDEVGGEALGR	L
4051	3	11	A	-0.2	3.88	0.93	0.93	sp|P02088.2|HBB1_MOUSE	57	K	VNSDEVGGEALGR	L
4294	2	11	B	-4.6	3.45	0.696	0.696	XP_011381407.1	99	K	VVAGVATALAHK	Y
4089	2	11	A	-4.8	3.88	0.729	0.729	XP_011381407.1	99	K	VVAGVATALAHK	Y

Scan	z	Sample ID	Band	PPM	XCorr	Delta correlation	Unique delta correlation	Reference	Redundancy		Peptides	
4100	2	11	A	-4.8	3.51	0.702	0.702	XP_011381407.1	99	K	VVAGVATALAHK	Y
4624	2	11	B	-0.9	6.4	0.454	0.454	ABU63177.1	99	R	YFDSFGDLSSASAIM*GNAK	V
4626	3	11	B	-1	5.54	0.494	0.494	ABU63177.1	99	R	YFDSFGDLSSASAIM*GNAK	V
4632	2	11	B	-0.9	6.72	0.496	0.496	ABU63177.1	99	R	YFDSFGDLSSASAIM*GNAK	V
4633	3	11	B	-1	5.1	0.534	0.534	ABU63177.1	99	R	YFDSFGDLSSASAIM*GNAK	V
4756	2	11	B	-1.5	4.41	0.576	0.576	ABU63177.1	99	R	YFDSFGDLSSASAIM*GNAK	V
4459	2	11	A	-1.2	6.57	0.513	0.513	ABU63177.1	99	R	YFDSFGDLSSASAIM*GNAK	V
4460	3	11	A	-1.1	5.69	0.471	0.471	ABU63177.1	99	R	YFDSFGDLSSASAIM*GNAK	V
4602	2	11	A	-3.1	3.38	0.524	0.524	ABU63177.1	99	R	YFDSFGDLSSASAIM*GNAK	V
4635	2	11	B	-2.3	4.16	0.939	0.939	CAA32225.1	99	R	YFDSFGDLSSASAIM*GNPK	V
4466	2	11	A	-1.5	6.05	0.901	0.901	CAA32225.1	99	R	YFDSFGDLSSASAIM*GNPK	V
4475	2	11	A	-1.5	4.91	0.904	0.904	CAA32225.1	99	R	YFDSFGDLSSASAIM*GNPK	V
4754	2	11	B	-2.1	6.66	0.56	0.56	ABU63177.1	99	R	YFDSFGDLSSASAIMGNAK	V
4764	2	11	B	-2.1	5.73	0.549	0.549	ABU63177.1	99	R	YFDSFGDLSSASAIMGNAK	V
4562	2	11	A	-2.1	3.83	0.391	0.391	ABU63177.1	99	R	YFDSFGDLSSASAIMGNAK	V
4588	2	11	A	-2	4.56	0.657	0.657	ABU63177.1	99	R	YFDSFGDLSSASAIMGNAK	V
4599	3	11	A	-1.5	6.32	0.369	0.369	ABU63177.1	99	R	YFDSFGDLSSASAIMGNAK	V
4768	3	11	B	-2	4.51	0.581	0.581	CAA32225.1	99	R	YFDSFGDLSSASAIMGNPK	V
2618	2	15	A	2.81	3.36	0.616	0.616	ABU63177.1	99	K	AAVSGLWGK	V
2627	2	15	A	2.81	3.36	0.619	0.619	ABU63177.1	99	K	AAVSGLWGK	V
3906	2	15	A	-0.5	2.58	0.621	0.621	ABU63177.1	99	K	AAVSGLWGK	V
3908	2	15	A	-0.5	2.53	0.646	0.646	ABU63177.1	99	K	AAVSGLWGK	V
4025	2	15	A	-0.5	2.52	0.66	0.66	ABU63177.1	99	K	AAVSGLWGK	V
4144	2	15	A	-0.4	2.56	0.626	0.626	ABU63177.1	99	K	AAVSGLWGK	V
4148	2	15	A	-0.4	2.56	0.644	0.644	ABU63177.1	99	K	AAVSGLWGK	V
2511	2	15	B	2.98	3.16	0.599	0.599	ABU63177.1	99	K	AAVSGLWGK	V
3386	2	15	B	1.41	2.71	0.67	0.67	ABU63177.1	99	K	AAVSGLWGK	V
3438	2	15	B	4.02	2.59	0.614	0.614	ABU63177.1	99	K	AAVSGLWGK	V
3447	2	15	B	3.66	2.6	0.607	0.607	ABU63177.1	99	K	AAVSGLWGK	V
3729	2	15	B	-0.3	2.53	0.648	0.648	ABU63177.1	99	K	AAVSGLWGK	V
2295	2	15	B	0.06	3.19	0.724	0.724	AAL73052.2	0	K	ADDIGADALGR	L
2596	2	15	A	3.73	2.95	0.871	0.871	CAA32225.1	99	K	DFTPAAQAAFQK	V
2607	2	15	A	3.73	3.07	0.879	0.879	CAA32225.1	99	K	DFTPAAQAAFQK	V
3894	2	15	A	0.2	2.9	0.855	0.855	CAA32225.1	99	K	DFTPAAQAAFQK	V
3898	2	15	A	0.21	2.93	0.887	0.887	CAA32225.1	99	K	DFTPAAQAAFQK	V
4009	2	15	A	0	2.96	0.885	0.885	CAA32225.1	99	K	DFTPAAQAAFQK	V
4012	2	15	A	0.05	2.6	0.907	0.907	CAA32225.1	99	K	DFTPAAQAAFQK	V
4128	2	15	A	0.39	2.69	0.909	0.909	CAA32225.1	99	K	DFTPAAQAAFQK	V

Scan	z	Sample ID	Band	PPM	XCorr	Delta correlation	Unique delta correlation	Reference	Redundancy		Peptides	
4133	2	15	A	0.42	2.61	0.878	0.878	CAA32225.1	99	K	DFTPAAQAAFQK	V
2478	2	15	B	2.67	3.4	0.889	0.889	CAA32225.1	99	K	DFTPAAQAAFQK	V
2481	3	15	B	0.04	4.18	0.821	0.821	CAA32225.1	99	K	DFTPAAQAAFQK	V
2489	2	15	B	2.68	3.26	0.887	0.887	CAA32225.1	99	K	DFTPAAQAAFQK	V
3598	2	15	B	0.32	3.04	0.895	0.895	CAA32225.1	99	K	DFTPAAQAAFQK	V
3720	2	15	B	0.15	2.75	0.912	0.912	CAA32225.1	99	K	DFTPAAQAAFQK	V
3728	2	15	B	0.12	2.7	0.877	0.877	CAA32225.1	99	K	DFTPAAQAAFQK	V
2800	2	15	A	2.12	3.14	0	0.325	pdb|1O1N|A	0	K	FLASVSTVLTSK	Y
2810	2	15	A	2.12	3.41	0	0.315	pdb|1O1N|A	0	K	FLASVSTVLTSK	Y
2940	2	15	A	2.94	2.59	0	0.383	pdb|1O1N|A	0	K	FLASVSTVLTSK	Y
3091	2	15	A	0.46	2.74	0	0.416	pdb|1O1N|A	0	K	FLASVSTVLTSK	Y
3097	2	15	A	0.46	2.5	0	0.416	pdb|1O1N|A	0	K	FLASVSTVLTSK	Y
3901	2	15	A	0.18	2.52	0	0.402	pdb|1O1N|A	0	K	FLASVSTVLTSK	Y
3905	2	15	A	0.1	2.97	0	0.351	pdb|1O1N|A	0	K	FLASVSTVLTSK	Y
4018	2	15	A	0.16	2.98	0	0.36	pdb|1O1N|A	0	K	FLASVSTVLTSK	Y
4021	2	15	A	0.19	2.63	0	0.381	pdb|1O1N|A	0	K	FLASVSTVLTSK	Y
4143	2	15	A	0.35	3.09	0	0.382	pdb|1O1N|A	0	K	FLASVSTVLTSK	Y
2687	2	15	B	4.33	3.08	0	0.347	pdb|1O1N|A	0	K	FLASVSTVLTSK	Y
2820	2	15	B	4.62	2.61	0	0.417	pdb|1O1N|A	0	K	FLASVSTVLTSK	Y
2962	2	15	B	1.6	2.89	0	0.369	pdb|1O1N|A	0	K	FLASVSTVLTSK	Y
2969	2	15	B	2.51	2.58	0	0.384	pdb|1O1N|A	0	K	FLASVSTVLTSK	Y
3558	2	15	B	0.32	3.03	0	0.279	pdb|1O1N|A	0	K	FLASVSTVLTSK	Y
3564	2	15	B	0.34	3.33	0	0.348	pdb|1O1N|A	0	K	FLASVSTVLTSK	Y
3674	2	15	B	0.25	2.97	0	0.369	pdb|1O1N|A	0	K	FLASVSTVLTSK	Y
3679	2	15	B	0.25	3.09	0	0.345	pdb|1O1N|A	0	K	FLASVSTVLTSK	Y
3787	2	15	B	0.26	2.75	0	0.391	pdb|1O1N|A	0	K	FLASVSTVLTSK	Y
3791	2	15	B	0.25	2.91	0	0.403	pdb|1O1N|A	0	K	FLASVSTVLTSK	Y
3903	2	15	B	0.2	3.11	0	0.351	pdb|1O1N|A	0	K	FLASVSTVLTSK	Y
3906	2	15	B	0.19	2.88	0	0.372	pdb|1O1N|A	0	K	FLASVSTVLTSK	Y
4016	2	15	B	0.11	2.78	0	0.354	pdb|1O1N|A	0	K	FLASVSTVLTSK	Y
4020	2	15	B	0.14	2.77	0	0.393	pdb|1O1N|A	0	K	FLASVSTVLTSK	Y
4257	2	15	B	-0.2	2.66	0	0.408	pdb|1O1N|A	0	K	FLASVSTVLTSK	Y
2998	3	15	A	2.34	9.16	0.108	0.108	ABU63212.1	11	K	IADALASAAGHLDDLPGALSALSDLHAHK	L
3066	3	15	A	1.81	11.1	0.141	0.141	ABU63212.1	11	K	IADALASAAGHLDDLPGALSALSDLHAHK	L
3077	3	15	A	1.8	10.5	0.132	0.132	ABU63212.1	11	K	IADALASAAGHLDDLPGALSALSDLHAHK	L
2937	3	15	B	4.2	6.75	0.2	0.2	ABU63212.1	11	K	IADALASAAGHLDDLPGALSALSDLHAHK	L
2471	2	15	B	0.88	3.48	0.966	0.966	BAB26925.1	0	K	IGGDGAEYGAEALER	M
3883	2	15	A	0.54	2.75	0.103	0.103	EGW10374.1	99	K	IGGHGAEYGAEALER	M

Scan	z	Sample ID	Band	PPM	XCorr	Delta correlation	Unique delta correlation	Reference	Redundancy		Peptides	
3610	2	15	B	-0	2.54	0.11	0.11	EGW10374.1	99	K	IGGHGAEYGAEALER	M
2556	2	15	B	4.89	3.96	0.915	0.915	AAB59723.1	13	K	IGGHGAEYVAEALER	M
2566	2	15	B	4.89	3.63	0.888	0.888	AAB59723.1	13	K	IGGHGAEYVAEALER	M
2567	3	15	B	4.52	3.78	0.769	0.769	AAB59723.1	13	K	IGGHGAEYVAEALER	M
4067	3	15	A	-0	3.45	0.86	0.86	sp|P04919.1|B3AT_MOUSE	1	R	ISGLLVSVLVGLSILM*EPILSR	I
3079	3	15	A	0.95	4.04	0.145	0.145	ABU63212.1	11	K	KIADALASAAGHLDDLPGALSALSDLHAHK	L
2944	3	15	B	3.37	7.34	0.18	0.18	ABU63212.1	11	K	KIADALASAAGHLDDLPGALSALSDLHAHK	L
2308	3	15	B	4.29	6.19	0.808	0.808	AQN67653.1	0	K	KVGAHAGEYGAEALER	M
2309	2	15	B	4.45	3.84	0.865	0.865	AQN67653.1	0	K	KVGAHAGEYGAEALER	M
3081	2	15	A	1.26	2.87	0.945	0.945	sp|Q7M2Y4.1|HBA_CHAMP	1	K	KVGDALGNAVAHLDDLPGALSALSDLHAHK	L
2866	3	15	B	1.86	6.1	0.791	0.791	sp|Q7M2Y4.1|HBA_CHAMP	1	K	KVGDALGNAVAHLDDLPGALSALSDLHAHK	L
2702	3	15	A	4.69	3.95	0.653	0.653	ABU63177.1	99	K	KVITAFNDGLNHLDSLK	G
2705	2	15	A	4.31	3.55	0.758	0.758	ABU63177.1	99	K	KVITAFNDGLNHLDSLK	G
2745	2	15	A	4.31	2.91	0.67	0.67	ABU63177.1	99	K	KVITAFNDGLNHLDSLK	G
2755	2	15	A	4.31	5.44	0.59	0.59	ABU63177.1	99	K	KVITAFNDGLNHLDSLK	G
2905	2	15	B	3.91	3.9	0.889	0.889	CAA32225.1	82	R	LLGNAIVIVLGHHLGK	D
2916	2	15	A	3.42	3.23	0.25	0.25	ABU63177.1	99	R	LLGNM*IVIVLGHHLGK	D
2924	2	15	A	3.41	2.86	0.204	0.204	ABU63177.1	99	R	LLGNM*IVIVLGHHLGK	D
3101	2	15	A	1.95	4.8	0.162	0.162	ABU63177.1	99	R	LLGNMIVIVLGHHLGK	D
3112	2	15	A	1.95	3.6	0.213	0.213	ABU63177.1	99	R	LLGNMIVIVLGHHLGK	D
2979	2	15	B	2.98	3.86	0.196	0.196	ABU63177.1	99	R	LLGNMIVIVLGHHLGK	D
2985	2	15	B	2.95	4.31	0.166	0.166	ABU63177.1	99	R	LLGNMIVIVLGHHLGK	D
2965	3	15	A	1.54	3.05	0.173	0.173	ELK09417.1	99	R	LLVVYPWTQR	F
3873	2	15	A	-0	2.59	0.237	0.237	ELK09417.1	99	R	LLVVYPWTQR	F
3879	2	15	A	0.03	2.61	0.207	0.207	ELK09417.1	99	R	LLVVYPWTQR	F
3991	2	15	A	0.23	2.61	0.236	0.236	ELK09417.1	99	R	LLVVYPWTQR	F
4113	2	15	A	0.24	2.74	0.23	0.23	ELK09417.1	99	R	LLVVYPWTQR	F
4117	2	15	A	0.28	2.63	0.242	0.242	ELK09417.1	99	R	LLVVYPWTQR	F
4226	2	15	A	-0	2.83	0.212	0.212	ELK09417.1	99	R	LLVVYPWTQR	F
4233	2	15	A	0	2.59	0.242	0.242	ELK09417.1	99	R	LLVVYPWTQR	F
2841	3	15	B	0.91	3.19	0.169	0.169	ELK09417.1	99	R	LLVVYPWTQR	F
2847	3	15	B	1.43	3.25	0.167	0.167	ELK09417.1	99	R	LLVVYPWTQR	F
2970	2	15	B	3.08	2.62	0.187	0.187	ELK09417.1	99	R	LLVVYPWTQR	F
3335	2	15	B	4.82	2.64	0.231	0.231	ELK09417.1	99	R	LLVVYPWTQR	F
3587	2	15	B	0.72	2.7	0.223	0.223	ELK09417.1	99	R	LLVVYPWTQR	F
3595	2	15	B	0.8	2.61	0.237	0.237	ELK09417.1	99	R	LLVVYPWTQR	F
3706	2	15	B	0.12	2.56	0.232	0.232	ELK09417.1	99	R	LLVVYPWTQR	F
3821	2	15	B	0.16	2.68	0.227	0.227	ELK09417.1	99	R	LLVVYPWTQR	F

Scan	z	Sample ID	Band	PPM	XCorr	Delta correlation	Unique delta correlation	Reference	Redundancy		Peptides	
3825	2	15	B	0.19	2.76	0.219	0.219	ELK09417.1	99	R	LLVVYPWTQR	F
3937	2	15	B	0.17	2.56	0.258	0.258	ELK09417.1	99	R	LLVVYPWTQR	F
3942	2	15	B	0.2	2.6	0.25	0.25	ELK09417.1	99	R	LLVVYPWTQR	F
2560	2	15	A	2.02	2.76	0	0.472	pdb|1O1N|A	0	K	LRVDPVNFK	L
2569	2	15	A	2.02	2.67	0	0.487	pdb|1O1N|A	0	K	LRVDPVNFK	L
2448	2	15	B	2.72	2.7	0	0.493	pdb|1O1N|A	0	K	LRVDPVNFK	L
2456	2	15	B	2.71	2.69	0	0.489	pdb|1O1N|A	0	K	LRVDPVNFK	L
2557	2	15	A	2.68	2.71	0.323	0.323	BAB27277.1	79	R	M*FASFPTTK	T
2568	2	15	A	2.68	2.75	0.335	0.335	BAB27277.1	79	R	M*FASFPTTK	T
4103	2	15	A	-0.4	2.56	0.314	0.314	BAB27277.1	79	R	M*FASFPTTK	T
3648	2	15	A	4.75	3.94	0	0.292	sp|B3EWD8.1|HBB_TAMHU	0	K	NVADEVGGEALGR^	L
3727	2	15	A	4.45	4.43	0	0.319	sp|B3EWD8.1|HBB_TAMHU	0	K	NVADEVGGEALGR^	L
3840	2	15	A	-0.2	3.89	0	0.205	sp|B3EWD8.1|HBB_TAMHU	0	K	NVADEVGGEALGR^	L
3956	2	15	A	0.06	4.07	0	0.259	sp|B3EWD8.1|HBB_TAMHU	0	K	NVADEVGGEALGR^	L
3960	2	15	A	0.03	4.17	0	0.26	sp|B3EWD8.1|HBB_TAMHU	0	K	NVADEVGGEALGR^	L
4076	2	15	A	-0.2	4.21	0	0.265	sp|B3EWD8.1|HBB_TAMHU	0	K	NVADEVGGEALGR^	L
4196	2	15	A	-0.1	4.19	0	0.246	sp|B3EWD8.1|HBB_TAMHU	0	K	NVADEVGGEALGR^	L
4432	2	15	A	-0.4	3.16	0	0.228	sp|B3EWD8.1|HBB_TAMHU	0	K	NVADEVGGEALGR^	L
4539	2	15	A	-0.4	2.78	0	0.292	sp|B3EWD8.1|HBB_TAMHU	0	K	NVADEVGGEALGR^	L
3435	2	15	B	4.83	3.72	0	0.291	sp|B3EWD8.1|HBB_TAMHU	0	K	NVADEVGGEALGR^	L
3492	2	15	B	0.18	4.05	0	0.285	sp|B3EWD8.1|HBB_TAMHU	0	K	NVADEVGGEALGR^	L
3511	2	15	B	0.55	4.01	0	0.295	sp|B3EWD8.1|HBB_TAMHU	0	K	NVADEVGGEALGR^	L
3745	2	15	B	0.05	3.82	0	0.286	sp|B3EWD8.1|HBB_TAMHU	0	K	NVADEVGGEALGR^	L
3854	2	15	B	0.11	3.82	0	0.295	sp|B3EWD8.1|HBB_TAMHU	0	K	NVADEVGGEALGR^	L
3969	2	15	B	-0.1	3.57	0	0.267	sp|B3EWD8.1|HBB_TAMHU	0	K	NVADEVGGEALGR^	L
4083	2	15	B	-0.5	2.56	0	0.31	sp|B3EWD8.1|HBB_TAMHU	0	K	NVADEVGGEALGR^	L
4087	2	15	B	-0.5	2.51	0	0.249	sp|B3EWD8.1|HBB_TAMHU	0	K	NVADEVGGEALGR^	L
4216	2	15	B	-0.5	2.59	0	0.249	sp|B3EWD8.1|HBB_TAMHU	0	K	NVADEVGGEALGR^	L
4325	2	15	B	-0.6	2.64	0	0.376	sp|B3EWD8.1|HBB_TAMHU	0	K	NVADEVGGEALGR^	L
4329	2	15	B	-0.6	2.62	0	0.324	sp|B3EWD8.1|HBB_TAMHU	0	K	NVADEVGGEALGR^	L
2619	3	15	A	2.03	3.22	0.145	0.145	EGW10374.1	99	K	TYFPHFDVSHGSAQVK	A
2620	2	15	A	2.14	4.17	0.123	0.123	EGW10374.1	99	K	TYFPHFDVSHGSAQVK	A
2628	3	15	A	1.99	3.44	0.114	0.114	EGW10374.1	99	K	TYFPHFDVSHGSAQVK	A
2629	2	15	A	2.16	3.73	0.128	0.128	EGW10374.1	99	K	TYFPHFDVSHGSAQVK	A
2492	3	15	B	2.11	3.16	0.11	0.11	EGW10374.1	99	K	TYFPHFDVSHGSAQVK	A
2493	2	15	B	2.27	4.05	0.123	0.123	EGW10374.1	99	K	TYFPHFDVSHGSAQVK	A
2500	3	15	B	2.66	3.41	0.11	0.11	EGW10374.1	99	K	TYFPHFDVSHGSAQVK	A
2501	2	15	B	2.73	4.5	0.111	0.111	EGW10374.1	99	K	TYFPHFDVSHGSAQVK	A

Scan	z	Sample ID	Band	PPM	XCorr	Delta correlation	Unique delta correlation	Reference	Redundancy		Peptides	
3008	3	15	A	2.88	5.14	0.8	0.8	sp|Q7M2Y4.1|HBA_CHAMP	1	K	VGDALGNAVAHLDDLPGALSALSDLHAHK	L
3020	3	15	A	2.7	4.91	0.797	0.797	sp|Q7M2Y4.1|HBA_CHAMP	1	K	VGDALGNAVAHLDDLPGALSALSDLHAHK	L
3139	3	15	A	2.21	5.62	0.784	0.784	sp|Q7M2Y4.1|HBA_CHAMP	1	K	VGDALGNAVAHLDDLPGALSALSDLHAHK	L
3146	3	15	A	2.19	5.19	0.748	0.748	sp|Q7M2Y4.1|HBA_CHAMP	1	K	VGDALGNAVAHLDDLPGALSALSDLHAHK	L
3045	3	15	B	3.56	4.06	0.809	0.809	sp|Q7M2Y4.1|HBA_CHAMP	1	K	VGDALGNAVAHLDDLPGALSALSDLHAHK	L
1000	2	15	B	-0.6	2.53	0.185	0.185	pdb|3HRW|D	26	-	VHLTDAEK	A
2799	2	15	A	2.85	5.23	0.538	0.538	ABU63177.1	99	K	VITAFNDGLNHLDSLK	G
3915	2	15	A	-0.1	2.64	0.721	0.721	ABU63177.1	99	K	VITAFNDGLNHLDSLK	G
4080	2	15	A	0.86	2.72	0.835	0.835	ABU63177.1	99	K	VITAFNDGLNHLDSLK	G
4088	2	15	A	0.59	2.71	0.692	0.692	ABU63177.1	99	K	VITAFNDGLNHLDSLK	G
4394	2	15	A	0.09	2.83	0.807	0.807	ABU63177.1	99	K	VITAFNDGLNHLDSLK	G
3871	2	15	B	0.81	2.65	0.731	0.731	ABU63177.1	99	K	VITAFNDGLNHLDSLK	G
2659	2	15	A	0.49	3.01	0.466	0.466	pdb|1I3R|D	88	K	VITAFNEGLK	G
2537	2	15	B	0.71	2.52	0.609	0.609	pdb|1I3R|D	88	K	VITAFNEGLK	G
2276	2	15	A	4	2.78	0.528	0.528	JAB00156.1	3	-	VLSGEDKSNIK	A
2285	2	15	A	3.96	2.91	0.463	0.463	JAB00156.1	3	-	VLSGEDKSNIK	A
2318	2	15	B	2.6	4.26	0.918	0.918	sp|P02088.2|HBB1_MOUSE	57	K	VNSDEVGGEALGR	L
2538	2	15	A	1.78	3.97	0.136	0.136	AFH54892.1	4	K	VNVDDVGGEALGR	L
2416	2	15	B	2.78	3.86	0.125	0.125	AFH54892.1	4	K	VNVDDVGGEALGR	L
2535	2	15	A	2.88	3.43	0.86	0.86	NP_032246.2	99	K	VVAGVAAALAHK	Y
2414	2	15	B	4.06	2.59	0.881	0.881	NP_032246.2	99	K	VVAGVAAALAHK	Y
3416	2	15	B	4.38	3.51	0.926	0.926	NP_032246.2	99	K	VVAGVAAALAHK	Y
3453	2	15	B	4.35	3.09	0.888	0.888	NP_032246.2	99	K	VVAGVAAALAHK	Y
3614	2	15	B	-0.6	2.64	0.944	0.944	NP_032246.2	99	K	VVAGVAAALAHK	Y
2547	2	15	A	2.48	4.34	0.864	0.864	NP_032246.2	99	K	VVAGVAAALAHKYH	-
2559	2	15	A	2.48	4.35	0.897	0.897	NP_032246.2	99	K	VVAGVAAALAHKYH	-
2436	2	15	B	2.78	4.8	0.907	0.907	NP_032246.2	99	K	VVAGVAAALAHKYH	-
2449	2	15	B	2.78	5.14	0.901	0.901	NP_032246.2	99	K	VVAGVAAALAHKYH	-
2350	2	15	B	1.92	2.7	0.676	0.676	XP_011381407.1	99	K	VVAGVATALAHK	Y
2526	2	15	A	1.29	5.04	0.325	0.325	XP_011381407.1	99	K	VVAGVATALAHKYH	-
2420	2	15	B	1.87	3.78	0.416	0.416	XP_011381407.1	99	K	VVAGVATALAHKYH	-
2846	2	15	A	4.42	3.49	0.488	0.488	ABU63177.1	99	R	YFDSFGDLSSASAIM*GNAK	V
2848	3	15	A	2.01	6.78	0.458	0.458	ABU63177.1	99	R	YFDSFGDLSSASAIM*GNAK	V
2858	3	15	A	2	7.11	0.434	0.434	ABU63177.1	99	R	YFDSFGDLSSASAIM*GNAK	V
2969	2	15	A	2.08	6.46	0.503	0.503	ABU63177.1	99	R	YFDSFGDLSSASAIM*GNAK	V
2976	2	15	A	2.2	6.14	0.503	0.503	ABU63177.1	99	R	YFDSFGDLSSASAIM*GNAK	V
3116	2	15	A	0.71	4.43	0.576	0.576	ABU63177.1	99	R	YFDSFGDLSSASAIM*GNAK	V
3125	2	15	A	0.8	4.93	0.587	0.587	ABU63177.1	99	R	YFDSFGDLSSASAIM*GNAK	V

Scan	z	Sample ID	Band	PPM	XCorr	Delta correlation	Unique delta correlation	Reference	Redundancy		Peptides	
3393	2	15	A	4.82	2.51	0.476	0.476	ABU63177.1	99	R	YFDSFGDLSSASAIM*GNAK	V
3724	2	15	A	4.88	4.71	0.584	0.584	ABU63177.1	99	R	YFDSFGDLSSASAIM*GNAK	V
3855	2	15	A	0.82	6.13	0.52	0.52	ABU63177.1	99	R	YFDSFGDLSSASAIM*GNAK	V
3863	2	15	A	1.05	5.19	0.589	0.589	ABU63177.1	99	R	YFDSFGDLSSASAIM*GNAK	V
3867	3	15	A	-0.3	3.27	0.387	0.387	ABU63177.1	99	R	YFDSFGDLSSASAIM*GNAK	V
3881	3	15	A	-0.3	4.58	0.39	0.39	ABU63177.1	99	R	YFDSFGDLSSASAIM*GNAK	V
3971	2	15	A	0.69	5.53	0.571	0.571	ABU63177.1	99	R	YFDSFGDLSSASAIM*GNAK	V
3974	2	15	A	0.72	5.06	0.539	0.539	ABU63177.1	99	R	YFDSFGDLSSASAIM*GNAK	V
3994	3	15	A	-0.1	3.72	0.376	0.376	ABU63177.1	99	R	YFDSFGDLSSASAIM*GNAK	V
4087	2	15	A	0.49	5.22	0.552	0.552	ABU63177.1	99	R	YFDSFGDLSSASAIM*GNAK	V
4090	2	15	A	0.47	5.31	0.564	0.564	ABU63177.1	99	R	YFDSFGDLSSASAIM*GNAK	V
4120	3	15	A	-0	3.4	0.351	0.351	ABU63177.1	99	R	YFDSFGDLSSASAIM*GNAK	V
4123	3	15	A	-0.1	3.78	0.373	0.373	ABU63177.1	99	R	YFDSFGDLSSASAIM*GNAK	V
4200	2	15	A	0.6	4.34	0.637	0.637	ABU63177.1	99	R	YFDSFGDLSSASAIM*GNAK	V
4205	2	15	A	0.63	4.24	0.565	0.565	ABU63177.1	99	R	YFDSFGDLSSASAIM*GNAK	V
4319	2	15	A	-0.5	2.87	0.759	0.759	ABU63177.1	99	R	YFDSFGDLSSASAIM*GNAK	V
4322	2	15	A	-0.5	2.99	0.629	0.629	ABU63177.1	99	R	YFDSFGDLSSASAIM*GNAK	V
4447	2	15	A	0.21	2.81	0.704	0.704	ABU63177.1	99	R	YFDSFGDLSSASAIM*GNAK	V
2722	3	15	B	3.97	6.05	0.463	0.463	ABU63177.1	99	R	YFDSFGDLSSASAIM*GNAK	V
2733	3	15	B	3.97	6.97	0.403	0.403	ABU63177.1	99	R	YFDSFGDLSSASAIM*GNAK	V
2858	2	15	B	2.55	2.77	0.684	0.684	ABU63177.1	99	R	YFDSFGDLSSASAIM*GNAK	V
2871	2	15	B	2.97	5.51	0.514	0.514	ABU63177.1	99	R	YFDSFGDLSSASAIM*GNAK	V
3009	2	15	B	2.21	5.24	0.51	0.51	ABU63177.1	99	R	YFDSFGDLSSASAIM*GNAK	V
3022	2	15	B	3.56	4.77	0.575	0.575	ABU63177.1	99	R	YFDSFGDLSSASAIM*GNAK	V
3545	2	15	B	0.47	5.17	0.598	0.598	ABU63177.1	99	R	YFDSFGDLSSASAIM*GNAK	V
3550	2	15	B	0.54	6	0.536	0.536	ABU63177.1	99	R	YFDSFGDLSSASAIM*GNAK	V
3569	3	15	B	0.16	3.8	0.416	0.416	ABU63177.1	99	R	YFDSFGDLSSASAIM*GNAK	V
3659	2	15	B	0.97	5.14	0.566	0.566	ABU63177.1	99	R	YFDSFGDLSSASAIM*GNAK	V
3667	2	15	B	1.03	4.11	0.658	0.658	ABU63177.1	99	R	YFDSFGDLSSASAIM*GNAK	V
3685	3	15	B	-0.2	3.28	0.405	0.405	ABU63177.1	99	R	YFDSFGDLSSASAIM*GNAK	V
3692	3	15	B	0.02	3.08	0.419	0.419	ABU63177.1	99	R	YFDSFGDLSSASAIM*GNAK	V
3774	2	15	B	0.67	4.92	0.534	0.534	ABU63177.1	99	R	YFDSFGDLSSASAIM*GNAK	V
3781	2	15	B	0.6	4.56	0.574	0.574	ABU63177.1	99	R	YFDSFGDLSSASAIM*GNAK	V
3892	2	15	B	1.25	4.3	0.709	0.709	ABU63177.1	99	R	YFDSFGDLSSASAIM*GNAK	V
3898	2	15	B	1.08	4.44	0.675	0.675	ABU63177.1	99	R	YFDSFGDLSSASAIM*GNAK	V
4007	2	15	B	0.54	3.37	0.694	0.694	ABU63177.1	99	R	YFDSFGDLSSASAIM*GNAK	V
4013	2	15	B	0.54	3.74	0.626	0.626	ABU63177.1	99	R	YFDSFGDLSSASAIM*GNAK	V
4124	2	15	B	-1	2.61	0.661	0.661	ABU63177.1	99	R	YFDSFGDLSSASAIM*GNAK	V

Scan	z	Sample ID	Band	PPM	XCorr	Delta correlation	Unique delta correlation	Reference	Redundancy		Peptides	
4252	2	15	B	0.27	2.73	0.718	0.718	ABU63177.1	99	R	YFDSFGDLSSASAIM*GNAK	V
3009	3	15	A	2.23	5.84	0.401	0.401	ABU63177.1	99	R	YFDSFGDLSSASAIMGNAK	V
3017	3	15	A	2.23	5.18	0.416	0.416	ABU63177.1	99	R	YFDSFGDLSSASAIMGNAK	V
3026	2	15	A	4.68	6.14	0.505	0.505	ABU63177.1	99	R	YFDSFGDLSSASAIMGNAK	V
3037	2	15	A	4.06	7.05	0.511	0.511	ABU63177.1	99	R	YFDSFGDLSSASAIMGNAK	V
3425	2	15	A	4.7	3.29	0.623	0.623	ABU63177.1	99	R	YFDSFGDLSSASAIMGNAK	V
3681	2	15	A	4.72	3.9	0.684	0.684	ABU63177.1	99	R	YFDSFGDLSSASAIMGNAK	V
3693	2	15	A	4.51	4.41	0.638	0.638	ABU63177.1	99	R	YFDSFGDLSSASAIMGNAK	V
3809	2	15	A	1.27	5.81	0.577	0.577	ABU63177.1	99	R	YFDSFGDLSSASAIMGNAK	V
3928	2	15	A	0.53	5.5	0.576	0.576	ABU63177.1	99	R	YFDSFGDLSSASAIMGNAK	V
3933	2	15	A	0.54	6	0.559	0.559	ABU63177.1	99	R	YFDSFGDLSSASAIMGNAK	V
4043	2	15	A	0.68	5.71	0.586	0.586	ABU63177.1	99	R	YFDSFGDLSSASAIMGNAK	V
4047	2	15	A	0.75	5.54	0.597	0.597	ABU63177.1	99	R	YFDSFGDLSSASAIMGNAK	V
4159	2	15	A	0.33	5.28	0.596	0.596	ABU63177.1	99	R	YFDSFGDLSSASAIMGNAK	V
4166	2	15	A	0.42	5.07	0.626	0.626	ABU63177.1	99	R	YFDSFGDLSSASAIMGNAK	V
4274	2	15	A	0.12	2.92	0.688	0.688	ABU63177.1	99	R	YFDSFGDLSSASAIMGNAK	V
4276	2	15	A	0.18	2.99	0.597	0.597	ABU63177.1	99	R	YFDSFGDLSSASAIMGNAK	V
4403	2	15	A	0	4.23	0.694	0.694	ABU63177.1	99	R	YFDSFGDLSSASAIMGNAK	V
4427	2	15	A	0.48	2.7	0.586	0.586	ABU63177.1	99	R	YFDSFGDLSSASAIMGNAK	V
3530	2	15	B	0.06	5.25	0.651	0.651	ABU63177.1	99	R	YFDSFGDLSSASAIMGNAK	V
3546	2	15	B	0.36	5.39	0.603	0.603	ABU63177.1	99	R	YFDSFGDLSSASAIMGNAK	V
3661	2	15	B	0.56	5.09	0.611	0.611	ABU63177.1	99	R	YFDSFGDLSSASAIMGNAK	V
3669	2	15	B	0.59	4.6	0.613	0.613	ABU63177.1	99	R	YFDSFGDLSSASAIMGNAK	V
3776	2	15	B	0.89	4.64	0.675	0.675	ABU63177.1	99	R	YFDSFGDLSSASAIMGNAK	V
3783	2	15	B	0.99	4.12	0.666	0.666	ABU63177.1	99	R	YFDSFGDLSSASAIMGNAK	V
3899	2	15	B	-0.3	3.43	0.652	0.652	ABU63177.1	99	R	YFDSFGDLSSASAIMGNAK	V
3904	2	15	B	0.31	3.51	0.639	0.639	ABU63177.1	99	R	YFDSFGDLSSASAIMGNAK	V
4017	2	15	B	0.66	3.05	0.655	0.655	ABU63177.1	99	R	YFDSFGDLSSASAIMGNAK	V
4021	2	15	B	0.66	2.85	0.672	0.672	ABU63177.1	99	R	YFDSFGDLSSASAIMGNAK	V
2960	2	15	A	3.34	4.53	0.578	0.578	ABU63177.1	99	R	YFDSFGDLSSASAIMGNAKVK	A
2853	2	17	A	1.09	2.89	0.622	0.622	ABU63177.1	99	K	AAVSGLWGK	V
2624	2	17	A	-0.7	3.75	0.813	0.813	sp|P04919.1|B3AT_MOUSE	1	K	ASGPGAAAQIQEVK	E
2797	2	17	A	3.99	3.4	0.888	0.888	CAA32225.1	99	K	DFTPAAQAAFQK	V
2800	3	17	A	2.83	4.49	0.844	0.844	CAA32225.1	99	K	DFTPAAQAAFQK	V
2528	2	17	B	2.4	3.32	0.887	0.887	CAA32225.1	99	K	DFTPAAQAAFQK	V
3029	2	17	A	0.02	2.88	0	0.286	pdb|1O1N|A	0	K	FLASVSTVLTSK	Y
2744	2	17	B	0.98	3.17	0	0.341	pdb|1O1N|A	0	K	FLASVSTVLTSK	Y
2748	2	17	B	0.95	3.28	0	0.305	pdb|1O1N|A	0	K	FLASVSTVLTSK	Y

Scan	z	Sample ID	Band	PPM	XCorr	Delta correlation	Unique delta correlation	Reference	Redundancy		Peptides	
3050	2	17	B	4.79	2.81	0	0.278	pdb|1O1N|A	0	K	FLASVSTVLTSK	Y
3244	2	17	B	0.22	2.67	0	0.372	pdb|1O1N|A	0	K	FLASVSTVLTSK	Y
3513	2	17	B	0.04	2.99	0	0.371	pdb|1O1N|A	0	K	FLASVSTVLTSK	Y
3520	2	17	B	0.01	2.81	0	0.379	pdb|1O1N|A	0	K	FLASVSTVLTSK	Y
3630	2	17	B	-0.2	2.78	0	0.378	pdb|1O1N|A	0	K	FLASVSTVLTSK	Y
3750	2	17	B	-0.3	2.58	0	0.424	pdb|1O1N|A	0	K	FLASVSTVLTSK	Y
3752	2	17	B	-0.2	2.91	0	0.389	pdb|1O1N|A	0	K	FLASVSTVLTSK	Y
3867	2	17	B	-0.2	2.56	0	0.425	pdb|1O1N|A	0	K	FLASVSTVLTSK	Y
3297	2	17	A	-0.9	2.54	0	0.812	ABU63212.1	11	K	IADALASAAGHLDDLPGALSALSDLHAHK	L
2896	3	17	A	0.25	5.05	0.89	0.89	AAB59723.1	13	K	IGGHGAEYVAEALER	M
2605	2	17	B	1.4	4.84	0.913	0.913	AAB59723.1	13	K	IGGHGAEYVAEALER	M
2616	2	17	B	1.41	4.54	0.914	0.914	AAB59723.1	13	K	IGGHGAEYVAEALER	M
2617	3	17	B	0.98	4.74	0.858	0.858	AAB59723.1	13	K	IGGHGAEYVAEALER	M
2627	3	17	B	0.97	4.69	0.881	0.881	AAB59723.1	13	K	IGGHGAEYVAEALER	M
2963	2	17	A	0.39	6.15	0.539	0.539	ABU63177.1	99	K	KVITAFNDGLNHLDSLK	G
2682	2	17	B	4.1	5.45	0.522	0.522	ABU63177.1	99	K	KVITAFNDGLNHLDSLK	G
2721	2	17	A	1.11	2.6	0.976	0.976	ERE78771.1	99	K	LHVDPENFR	L
3458	2	17	A	-0.1	2.58	0	0.863	sp|P04919.1|B3AT_MOUSE	0	R	LILPLIFR	E
3187	2	17	B	-0.1	2.63	0	0.877	sp|P04919.1|B3AT_MOUSE	0	R	LILPLIFR	E
3096	2	17	A	0.85	3.68	0.181	0.181	ABU63177.1	99	R	LLGNM*IVIVLGHHLGK	D
3108	2	17	A	4.77	3.57	0.202	0.202	ABU63177.1	99	R	LLGNM*IVIVLGHHLGK	D
3336	2	17	A	0.43	4	0.209	0.209	ABU63177.1	99	R	LLGNMIVIVLGHHLGK	D
3159	2	17	A	0.75	2.92	0.21	0.21	ELK09417.1	99	R	LLVVYPWTQR	F
2891	2	17	B	1.88	2.96	0.17	0.17	ELK09417.1	99	R	LLVVYPWTQR	F
3572	2	17	B	0.6	2.52	0.236	0.236	ELK09417.1	99	R	LLVVYPWTQR	F
3686	2	17	B	-0.4	2.65	0.23	0.23	ELK09417.1	99	R	LLVVYPWTQR	F
3691	2	17	B	-0.3	2.53	0.24	0.24	ELK09417.1	99	R	LLVVYPWTQR	F
3812	2	17	B	-0.4	2.59	0.238	0.238	ELK09417.1	99	R	LLVVYPWTQR	F
4091	2	17	B	-0.5	2.61	0.236	0.236	ELK09417.1	99	R	LLVVYPWTQR	F
4100	2	17	B	-0.4	2.8	0.217	0.217	ELK09417.1	99	R	LLVVYPWTQR	F
2770	2	17	A	0.2	2.68	0	0.496	pdb|1O1N|A	0	K	LRVDPVNFK	L
2775	2	17	A	0.2	2.75	0	0.488	pdb|1O1N|A	0	K	LRVDPVNFK	L
2475	2	17	B	0.71	2.76	0	0.469	pdb|1O1N|A	0	K	LRVDPVNFK	L
2486	2	17	B	0.71	2.69	0	0.481	pdb|1O1N|A	0	K	LRVDPVNFK	L
2484	2	17	B	1.64	2.77	0.332	0.332	BAB27277.1	79	R	M*FASFPTTK	T
2495	2	17	B	1.63	2.75	0.337	0.337	BAB27277.1	79	R	M*FASFPTTK	T
2606	2	17	B	2.33	2.59	0.395	0.395	BAB27277.1	79	R	MFASFPTTK	T
2625	2	17	A	0.29	2.92	0	0.26	sp|B3EWD8.1|HBB_TAMHU	0	K	NVADEVGGEALGR^	L

Scan	z	Sample ID	Band	PPM	XCorr	Delta correlation	Unique delta correlation	Reference	Redundancy		Peptides	
3530	2	17	B	0.23	2.97	0	0.23	sp|B3EWD8.1|HBB_TAMHU	0	K	NVADEVGGEALGR^	L
3693	2	17	B	-0.1	3.16	0	0.215	sp|B3EWD8.1|HBB_TAMHU	0	K	NVADEVGGEALGR^	L
3698	2	17	B	-0.2	3.12	0	0.212	sp|B3EWD8.1|HBB_TAMHU	0	K	NVADEVGGEALGR^	L
3813	2	17	B	-0.3	3.03	0	0.286	sp|B3EWD8.1|HBB_TAMHU	0	K	NVADEVGGEALGR^	L
3839	2	17	B	-0.2	3.37	0	0.294	sp|B3EWD8.1|HBB_TAMHU	0	K	NVADEVGGEALGR^	L
3977	2	17	B	-0.4	2.92	0	0.276	sp|B3EWD8.1|HBB_TAMHU	0	K	NVADEVGGEALGR^	L
3657	2	17	B	-0.3	3.55	0.993	0.993	sp|P04919.1|B3AT_MOUSE	2	K	STPASLALPFVLILTVPLR	R
2854	3	17	A	0.2	3.38	0.116	0.116	EGW10374.1	99	K	TYFPHFDVSHGSAQVK	A
2855	2	17	A	0.51	4.35	0.117	0.117	EGW10374.1	99	K	TYFPHFDVSHGSAQVK	A
2864	2	17	A	4.31	3.51	0.125	0.125	EGW10374.1	99	K	TYFPHFDVSHGSAQVK	A
2550	3	17	B	1.22	3.34	0.116	0.116	EGW10374.1	99	K	TYFPHFDVSHGSAQVK	A
2562	2	17	B	1.31	4.2	0.119	0.119	EGW10374.1	99	K	TYFPHFDVSHGSAQVK	A
3338	3	17	A	-1	4.01	0.786	0.786	sp|Q7M2Y4.1|HBA_CHAMP	1	K	VGDALGNAVAHLDDLPGALSALSDLHAHK	L
2456	2	17	A	-0	2.6	0.177	0.177	pdb|3HRW|D	26	-	VHLTDAEK	A
3028	2	17	A	0.59	5.53	0.561	0.561	ABU63177.1	99	K	VITAFNDGLNHLDSLK	G
3039	2	17	A	3.66	5.71	0.58	0.58	ABU63177.1	99	K	VITAFNDGLNHLDSLK	G
2445	2	17	A	-0.1	3.48	0.521	0.521	JAB00156.1	3	-	VLSGEDKSNIK	A
2209	2	17	B	1.07	3.74	0.545	0.545	JAB00156.1	3	-	VLSGEDKSNIK	A
2221	2	17	B	1.07	3.66	0.515	0.515	JAB00156.1	3	-	VLSGEDKSNIK	A
2378	2	17	B	4.54	5.15	0.93	0.93	sp|P02088.2|HBB1_MOUSE	57	K	VNSDEVGGEALGR	L
2737	2	17	A	0.09	2.93	0.127	0.127	AFH54892.1	4	K	VNVDDVGGEALGR	L
2590	2	17	B	-0.8	2.52	0.877	0.877	NP_032246.2	99	K	VVAGVAAALAHK	Y
2754	2	17	A	1.98	4.5	0.904	0.904	NP_032246.2	99	K	VVAGVAAALAHKYH	-
2765	2	17	A	2.01	4.07	0.893	0.893	NP_032246.2	99	K	VVAGVAAALAHKYH	-
2474	2	17	B	1.56	4.61	0.903	0.903	NP_032246.2	99	K	VVAGVAAALAHKYH	-
2487	2	17	B	1.56	5	0.902	0.902	NP_032246.2	99	K	VVAGVAAALAHKYH	-
2690	2	17	A	-0.4	2.75	0.76	0.76	XP_011381407.1	99	K	VVAGVATALAHK	Y
2733	2	17	A	0.35	4.48	0.352	0.352	XP_011381407.1	99	K	VVAGVATALAHKYH	-
3061	2	17	A	4.2	6.37	0.476	0.476	ABU63177.1	99	R	YFDSFGDLSSASAIM*GNAK	V
3072	2	17	A	0.35	5.89	0.583	0.583	ABU63177.1	99	R	YFDSFGDLSSASAIM*GNAK	V
3669	2	17	A	-0.5	4.04	0.676	0.676	ABU63177.1	99	R	YFDSFGDLSSASAIM*GNAK	V
3729	2	17	A	0.05	3.55	0.685	0.685	ABU63177.1	99	R	YFDSFGDLSSASAIM*GNAK	V
3743	2	17	A	0.53	3.16	0.642	0.642	ABU63177.1	99	R	YFDSFGDLSSASAIM*GNAK	V
3852	2	17	A	-0.3	3.33	0.727	0.727	ABU63177.1	99	R	YFDSFGDLSSASAIM*GNAK	V
3881	2	17	A	-0.4	3.19	0.7	0.7	ABU63177.1	99	R	YFDSFGDLSSASAIM*GNAK	V
4000	2	17	A	0.71	3.01	0.627	0.627	ABU63177.1	99	R	YFDSFGDLSSASAIM*GNAK	V
4002	2	17	A	0.47	2.56	0.699	0.699	ABU63177.1	99	R	YFDSFGDLSSASAIM*GNAK	V
4130	2	17	A	-0.2	2.9	0.718	0.718	ABU63177.1	99	R	YFDSFGDLSSASAIM*GNAK	V

Scan	z	Sample ID	Band	PPM	XCorr	Delta correlation	Unique delta correlation	Reference	Redundancy		Peptides	
2790	2	17	B	2.46	6.63	0.435	0.435	ABU63177.1	99	R	YFDSFGDLSSASAIM*GNAK	V
2792	2	17	B	2.02	6.49	0.442	0.442	ABU63177.1	99	R	YFDSFGDLSSASAIM*GNAK	V
2793	3	17	B	0.35	7	0.403	0.403	ABU63177.1	99	R	YFDSFGDLSSASAIM*GNAK	V
2913	2	17	B	4.73	3.64	0.593	0.593	ABU63177.1	99	R	YFDSFGDLSSASAIM*GNAK	V
2923	2	17	B	4.64	5.27	0.542	0.542	ABU63177.1	99	R	YFDSFGDLSSASAIM*GNAK	V
3081	2	17	B	4.69	5.08	0.54	0.54	ABU63177.1	99	R	YFDSFGDLSSASAIM*GNAK	V
3122	2	17	B	0.15	5.45	0.609	0.609	ABU63177.1	99	R	YFDSFGDLSSASAIM*GNAK	V
3157	2	17	B	0.05	5.03	0.636	0.636	ABU63177.1	99	R	YFDSFGDLSSASAIM*GNAK	V
3288	2	17	B	-0.1	5.1	0.521	0.521	ABU63177.1	99	R	YFDSFGDLSSASAIM*GNAK	V
3414	2	17	B	0.62	4.74	0.587	0.587	ABU63177.1	99	R	YFDSFGDLSSASAIM*GNAK	V
3424	2	17	B	0.52	5.07	0.536	0.536	ABU63177.1	99	R	YFDSFGDLSSASAIM*GNAK	V
3506	3	17	B	-0.7	4.02	0.485	0.485	ABU63177.1	99	R	YFDSFGDLSSASAIM*GNAK	V
3538	2	17	B	0.14	4.08	0.639	0.639	ABU63177.1	99	R	YFDSFGDLSSASAIM*GNAK	V
3557	2	17	B	0.15	4.31	0.7	0.7	ABU63177.1	99	R	YFDSFGDLSSASAIM*GNAK	V
3672	2	17	B	0.08	5.61	0.591	0.591	ABU63177.1	99	R	YFDSFGDLSSASAIM*GNAK	V
3675	2	17	B	0.04	4.56	0.677	0.677	ABU63177.1	99	R	YFDSFGDLSSASAIM*GNAK	V
3789	2	17	B	0.1	5.8	0.527	0.527	ABU63177.1	99	R	YFDSFGDLSSASAIM*GNAK	V
3792	2	17	B	0.03	5.69	0.532	0.532	ABU63177.1	99	R	YFDSFGDLSSASAIM*GNAK	V
3796	3	17	B	-0.5	3.51	0.302	0.302	ABU63177.1	99	R	YFDSFGDLSSASAIM*GNAK	V
3907	2	17	B	0.14	4.69	0.583	0.583	ABU63177.1	99	R	YFDSFGDLSSASAIM*GNAK	V
3910	2	17	B	0.08	4.44	0.602	0.602	ABU63177.1	99	R	YFDSFGDLSSASAIM*GNAK	V
4029	2	17	B	0.05	4.28	0.516	0.516	ABU63177.1	99	R	YFDSFGDLSSASAIM*GNAK	V
4043	2	17	B	0.1	4.2	0.587	0.587	ABU63177.1	99	R	YFDSFGDLSSASAIM*GNAK	V
4160	2	17	B	0.05	3.25	0.707	0.707	ABU63177.1	99	R	YFDSFGDLSSASAIM*GNAK	V
4394	2	17	B	0.03	2.72	0.694	0.694	ABU63177.1	99	R	YFDSFGDLSSASAIM*GNAK	V
4396	2	17	B	0.04	2.77	0.642	0.642	ABU63177.1	99	R	YFDSFGDLSSASAIM*GNAK	V
2945	3	17	B	4.91	4.94	0.355	0.355	ABU63177.1	99	R	YFDSFGDLSSASAIMGNAK	V
2957	3	17	B	4.89	3.46	0.306	0.306	ABU63177.1	99	R	YFDSFGDLSSASAIMGNAK	V
3114	2	17	B	0.35	4.39	0.728	0.728	ABU63177.1	99	R	YFDSFGDLSSASAIMGNAK	V
3265	2	17	B	4.69	2.59	0.738	0.738	ABU63177.1	99	R	YFDSFGDLSSASAIMGNAK	V
3460	2	17	B	0.1	3	0.688	0.688	ABU63177.1	99	R	YFDSFGDLSSASAIMGNAK	V
3466	2	17	B	0.08	3.62	0.613	0.613	ABU63177.1	99	R	YFDSFGDLSSASAIMGNAK	V
3583	2	17	B	0.05	5.16	0.627	0.627	ABU63177.1	99	R	YFDSFGDLSSASAIMGNAK	V
3591	2	17	B	0.2	4.59	0.628	0.628	ABU63177.1	99	R	YFDSFGDLSSASAIMGNAK	V
3703	2	17	B	0.09	4.76	0.605	0.605	ABU63177.1	99	R	YFDSFGDLSSASAIMGNAK	V
3716	2	17	B	0.22	4.79	0.659	0.659	ABU63177.1	99	R	YFDSFGDLSSASAIMGNAK	V
3830	2	17	B	0.03	4.54	0.643	0.643	ABU63177.1	99	R	YFDSFGDLSSASAIMGNAK	V
3843	2	17	B	0.15	4.32	0.66	0.66	ABU63177.1	99	R	YFDSFGDLSSASAIMGNAK	V

Scan	z	Sample ID	Band	PPM	XCorr	Delta correlation	Unique delta correlation	Reference	Redundancy		Peptides	
3964	2	17	B	-0.4	3.1	0.603	0.603	ABU63177.1	99	R	YFDSFGDLSSASAIMGNAK	V
3973	2	17	B	-0.8	3.88	0.596	0.596	ABU63177.1	99	R	YFDSFGDLSSASAIMGNAK	V
4102	2	17	B	0.19	3.38	0.804	0.804	ABU63177.1	99	R	YFDSFGDLSSASAIMGNAK	V
3407	2	19	A	1.63	3.21	0	0.417	pdb|1O1N|A	0	K	FLASVSTVLTSK	Y
3413	2	19	A	-4.5	2.76	0	0.439	pdb|1O1N|A	0	K	FLASVSTVLTSK	Y
4062	2	19	A	-1.2	2.56	0	0.439	pdb|1O1N|A	0	K	FLASVSTVLTSK	Y
4183	2	19	A	-1.6	2.61	0	0.454	pdb|1O1N|A	0	K	FLASVSTVLTSK	Y
3237	2	19	B	0.5	3.33	0.904	0.904	AAB59723.1	13	K	IGGHGAEYVAEALER	M
3254	2	19	B	0.47	2.63	0.899	0.899	AAB59723.1	13	K	IGGHGAEYVAEALER	M
3358	2	19	A	1.08	4.79	0.519	0.519	ABU63177.1	99	K	KVITAFNDGLNHLDSLK	G
4084	2	19	A	-1.2	2.59	0.255	0.255	ELK09417.1	99	R	LLVVYPWTQR	F
3921	2	19	A	-1	2.6	0	0.165	sp|B3EWD8.1|HBB_TAMHU	0	K	NVADEVGGEALGR^	L
4042	2	19	A	-1.2	3.04	0	0.173	sp|B3EWD8.1|HBB_TAMHU	0	K	NVADEVGGEALGR^	L
4153	2	19	A	-1.7	2.99	0	0.16	sp|B3EWD8.1|HBB_TAMHU	0	K	NVADEVGGEALGR^	L
4631	2	19	A	-1.4	3.18	0	0.195	sp|B3EWD8.1|HBB_TAMHU	0	K	NVADEVGGEALGR^	L
4533	3	19	A	-0.8	4.52	0.762	0.762	sp|Q7M2Y4.1|HBA_CHAMP	1	K	VGDALGNAVAHLDDLPGALSALSDLHAHK	L
4542	3	19	A	-0.7	3.01	0.87	0.87	sp|Q7M2Y4.1|HBA_CHAMP	1	K	VGDALGNAVAHLDDLPGALSALSDLHAHK	L
2941	2	19	A	-2.8	2.52	0.142	0.142	pdb|3HRW|D	26	-	VHLTDAEK	A
2310	2	19	B	-2.9	2.56	0.179	0.179	pdb|3HRW|D	26	-	VHLTDAEK	A
2430	2	19	B	-1.2	2.65	0.173	0.173	pdb|3HRW|D	26	-	VHLTDAEK	A
2549	2	19	B	-1.1	2.54	0.178	0.178	pdb|3HRW|D	26	-	VHLTDAEK	A
2665	2	19	B	-1.2	2.53	0.14	0.14	pdb|3HRW|D	26	-	VHLTDAEK	A
2672	2	19	B	-1.2	2.53	0.146	0.146	pdb|3HRW|D	26	-	VHLTDAEK	A
2796	2	19	B	-1	2.76	0.16	0.16	pdb|3HRW|D	26	-	VHLTDAEK	A
3410	2	19	A	0.53	3.12	0.718	0.718	ABU63177.1	99	K	VITAFNDGLNHLDSLK	G
4569	2	19	A	-0.8	2.76	0.798	0.798	ABU63177.1	99	K	VITAFNDGLNHLDSLK	G
4576	2	19	A	-0.9	2.91	0.746	0.746	ABU63177.1	99	K	VITAFNDGLNHLDSLK	G
2907	2	19	A	-2.3	2.76	0.499	0.499	JAB00156.1	3	-	VLSGEDKSNIK	A
2482	2	19	B	-1.7	2.58	0.428	0.428	JAB00156.1	3	-	VLSGEDKSNIK	A
2595	2	19	B	-1.9	3.08	0.56	0.56	JAB00156.1	3	-	VLSGEDKSNIK	A
2601	2	19	B	-1.9	2.96	0.567	0.567	JAB00156.1	3	-	VLSGEDKSNIK	A
2709	2	19	B	-1.6	3.35	0.489	0.489	JAB00156.1	3	-	VLSGEDKSNIK	A
2718	2	19	B	-1.5	2.91	0.514	0.514	JAB00156.1	3	-	VLSGEDKSNIK	A
3892	2	19	A	-1.3	2.84	0.867	0.867	NP_032246.2	99	K	VVAGVAAALAHK	Y
3911	2	19	A	-1.4	3.31	0.9	0.9	NP_032246.2	99	K	VVAGVAAALAHK	Y
4019	2	19	A	-1.3	3.68	0.837	0.837	NP_032246.2	99	K	VVAGVAAALAHK	Y
4031	2	19	A	-1.3	3.32	0.882	0.882	NP_032246.2	99	K	VVAGVAAALAHK	Y
4139	2	19	A	-1.5	3.38	0.889	0.889	NP_032246.2	99	K	VVAGVAAALAHK	Y

Scan	z	Sample ID	Band	PPM	XCorr	Delta correlation	Unique delta correlation	Reference	Redundancy		Peptides	
4151	2	19	A	-1.6	3.09	0.922	0.922	NP_032246.2	99	K	VVAGVAAALAHK	Y
4608	2	19	A	-1.2	3.7	0.897	0.897	NP_032246.2	99	K	VVAGVAAALAHK	Y
4620	2	19	A	-1.3	3.03	0.882	0.882	NP_032246.2	99	K	VVAGVAAALAHK	Y
4118	2	19	B	-1.6	2.66	0.877	0.877	NP_032246.2	99	K	VVAGVAAALAHK	Y
4601	2	19	B	-1.4	2.92	0.87	0.87	NP_032246.2	99	K	VVAGVAAALAHK	Y
4611	2	19	B	-1.4	2.72	0.853	0.853	NP_032246.2	99	K	VVAGVAAALAHK	Y
3044	2	19	B	-1	3.6	0.701	0.701	XP_011381407.1	99	K	VVAGVATALAHK	Y
3403	2	19	B	-3.6	3.45	0.652	0.652	ACY03371.1	3	R	YFDSFGDLSSACAIMGNAK	V
3412	2	19	B	-2.1	3.06	0.512	0.512	ACY03371.1	3	R	YFDSFGDLSSACAIMGNAK	V
3988	2	19	A	-1.3	2.62	0.433	0.433	ABU63177.1	99	R	YFDSFGDLSSASAIM*GNAK	V
4592	2	19	A	-1.2	3.12	0.632	0.632	ABU63177.1	99	R	YFDSFGDLSSASAIM*GNAK	V
4602	2	19	A	-1.4	3.49	0.614	0.614	ABU63177.1	99	R	YFDSFGDLSSASAIM*GNAK	V
3908	2	19	A	-1	4.15	0.511	0.511	ABU63177.1	99	R	YFDSFGDLSSASAIMGNAK	V
4018	2	19	A	-0.9	5.51	0.603	0.603	ABU63177.1	99	R	YFDSFGDLSSASAIMGNAK	V
4030	2	19	A	-1	5.14	0.6	0.6	ABU63177.1	99	R	YFDSFGDLSSASAIMGNAK	V
4140	2	19	A	-1.1	4.2	0.63	0.63	ABU63177.1	99	R	YFDSFGDLSSASAIMGNAK	V
4150	2	19	A	-1.3	4.59	0.551	0.551	ABU63177.1	99	R	YFDSFGDLSSASAIMGNAK	V
4610	2	19	A	-1	5.68	0.581	0.581	ABU63177.1	99	R	YFDSFGDLSSASAIMGNAK	V
4622	2	19	A	-1	4.49	0.549	0.549	ABU63177.1	99	R	YFDSFGDLSSASAIMGNAK	V
3995	2	19	B	-2.3	3.4	0.461	0.461	ABU63177.1	99	R	YFDSFGDLSSASAIMGNAK	V
4116	2	19	B	-1.3	4.51	0.565	0.565	ABU63177.1	99	R	YFDSFGDLSSASAIMGNAK	V
4121	3	19	B	-1.5	3.21	0.41	0.41	ABU63177.1	99	R	YFDSFGDLSSASAIMGNAK	V
4600	2	19	B	-1.1	4.8	0.626	0.626	ABU63177.1	99	R	YFDSFGDLSSASAIMGNAK	V
4612	2	19	B	-1.1	5.36	0.603	0.603	ABU63177.1	99	R	YFDSFGDLSSASAIMGNAK	V
3161	3	22	A	-0.4	4.26	0.824	0.824	pdb|2QMB|A	16	K	VVAALIEAANHIDDIAGTLSK	L
3164	2	22	A	-0.3	4.13	0.88	0.88	pdb|2QMB|A	16	K	VVAALIEAANHIDDIAGTLSK	L
2535	2	22	A	-1.2	3.62	0.888	0.888	NP_032246.2	99	K	VVAGVAAALAHK	Y
5204	2	23	A	0.85	2.71	0	0.374	pdb|1O1N|A	0	K	FLASVSTVLTSK	Y
5457	2	23	A	4.03	3.6	0.529	0.529	ABU63177.1	99	R	YFDSFGDLSSASAIMGNAK	V
4164	2	24	A	-3.4	3.22	0.635	0.635	ABU63177.1	99	K	AAVSGLWGK	V
3898	2	24	A	-0.5	3.03	0.691	0.691	AAL73052.2	0	K	ADDIGADALGR	L
4782	2	24	B	0.62	3.66	0.868	0.868	CAA32225.1	99	K	DFTPAAQAAFQK	V
4786	2	24	B	-0.6	3.66	0.884	0.884	CAA32225.1	99	K	DFTPAAQAAFQK	V
4443	2	24	A	0.63	3.5	0	0.312	pdb|1O1N|A	0	K	FLASVSTVLTSK	Y
4454	2	24	A	-1.1	3.48	0	0.347	pdb|1O1N|A	0	K	FLASVSTVLTSK	Y
4850	2	24	A	3.98	2.73	0	0.403	pdb|1O1N|A	0	K	FLASVSTVLTSK	Y
5075	2	24	B	-0.8	3.41	0	0.403	pdb|1O1N|A	0	K	FLASVSTVLTSK	Y
4806	3	24	A	-0.2	8.25	0.181	0.181	ABU63212.1	11	K	IADALASAAGHLDDLPGALSALSDLHAHK	L

Scan	z	Sample ID	Band	PPM	XCorr	Delta correlation	Unique delta correlation	Reference	Redundancy		Peptides	
5491	3	24	B	0.31	8.8	0.101	0.101	ABU63212.1	11	K	IADALASAAGHLDDLPGALSALSDLHAHK	L
5498	2	24	B	0.86	3.36	0	0.667	ABU63212.1	11	K	IADALASAAGHLDDLPGALSALSDLHAHK	L
3973	2	24	A	0.23	4.3	0.102	0.102	EGW10374.1	99	K	IGGHGAEYGAEALER	M
4278	2	24	A	0.15	4.49	0.916	0.916	AAB59723.1	13	K	IGGHGAEYVAEALER	M
4289	2	24	A	0.15	4.18	0.918	0.918	AAB59723.1	13	K	IGGHGAEYVAEALER	M
4290	3	24	A	-0.7	3.9	0.862	0.862	AAB59723.1	13	K	IGGHGAEYVAEALER	M
4888	2	24	B	-2.8	2.79	0.9	0.9	AAB59723.1	13	K	IGGHGAEYVAEALER	M
4899	2	24	B	-1.9	3.77	0.908	0.908	AAB59723.1	13	K	IGGHGAEYVAEALER	M
4835	3	24	A	1.31	10	0.213	0.213	ABU63212.1	11	K	KIADALASAAGHLDDLPGALSALSDLHAHK	L
5506	3	24	B	-3.2	9.97	0.165	0.165	ABU63212.1	11	K	KIADALASAAGHLDDLPGALSALSDLHAHK	L
4366	3	24	A	-0.1	5.79	0.522	0.522	ABU63177.1	99	K	KVITAFNDGLNHLDSLK	G
4377	3	24	A	-0.1	5.68	0.501	0.501	ABU63177.1	99	K	KVITAFNDGLNHLDSLK	G
4998	2	24	B	-1.6	4.42	0.618	0.618	ABU63177.1	99	K	KVITAFNDGLNHLDSLK	G
5197	2	24	B	-0.3	3.68	0.739	0.739	pdb|5GHK|A	0	K	LGEYGFQNALLVR	Y
5501	2	24	A	3.53	2.58	0.116	0.116	AJA37790.1	99	R	LLGDILIIVLAAHFSK	D
5099	2	24	A	1.39	2.62	0.302	0.302	XP_005074210.1	0	R	LLGNMIIIALSHHLGK	E
4818	2	24	A	0	3.85	0.175	0.175	ABU63177.1	99	R	LLGNMIVIVLGHHLGK	D
4826	2	24	A	0.18	4.11	0.189	0.189	ABU63177.1	99	R	LLGNMIVIVLGHHLGK	D
5494	2	24	B	1.74	3.86	0.17	0.17	ABU63177.1	99	R	LLGNMIVIVLGHHLGK	D
5502	2	24	B	-4.5	3.43	0.162	0.162	ABU63177.1	99	R	LLGNMIVIVLGHHLGK	D
4666	2	24	A	0.2	3.08	0.206	0.206	ELK09417.1	99	R	LLVVYPWTQR	F
4739	3	24	B	3.03	3.39	0	0.816	pdb|1O1N|A	0	K	LRVDPVNFK	L
4749	3	24	B	3.03	3.34	0	0.814	pdb|1O1N|A	0	K	LRVDPVNFK	L
4120	2	24	A	-2.4	2.9	0.289	0.289	BAB27277.1	79	R	M*FASFPTTK	T
4738	2	24	B	1.59	2.67	0.33	0.33	BAB27277.1	79	R	M*FASFPTTK	T
4867	2	24	B	-1.1	2.7	0.39	0.39	BAB27277.1	79	R	MFASFPTTK	T
4877	2	24	B	-1.1	2.66	0.379	0.379	BAB27277.1	79	R	MFASFPTTK	T
3940	2	24	A	-2.1	3.16	0	0.288	sp|B3EWD8.1|HBB_TAMHU	0	K	NVADEVGGEALGR^	L
4558	2	24	B	-3.1	3.36	0	0.284	sp|B3EWD8.1|HBB_TAMHU	0	K	NVADEVGGEALGR^	L
4765	2	24	B	1.13	2.74	0	0.245	sp|B3EWD8.1|HBB_TAMHU	0	K	NVADEVGGEALGR^	L
6414	2	24	B	-2.6	2.64	0	0.239	sp|B3EWD8.1|HBB_TAMHU	0	K	NVADEVGGEALGR^	L
4824	2	24	B	-1.4	3.98	0.11	0.11	EGW10374.1	99	K	TYFPHFDVSHGSAQVK	A
4845	3	24	B	-3.8	3.61	0.116	0.116	EGW10374.1	99	K	TYFPHFDVSHGSAQVK	A
4870	3	24	A	1.51	4.03	0.828	0.828	sp|Q7M2Y4.1|HBA_CHAMP	1	K	VGDALGNAVAHLDDLPGALSALSDLHAHK	L
5404	3	24	B	-2.4	4.47	0.735	0.735	sp|Q7M2Y4.1|HBA_CHAMP	1	K	VGDALGNAVAHLDDLPGALSALSDLHAHK	L
3582	2	24	A	-2.3	2.55	0.192	0.192	pdb|3HRW|D	26	-	VHLTDAEK	A
4220	2	24	B	-2.5	2.59	0.194	0.194	pdb|3HRW|D	26	-	VHLTDAEK	A
4400	2	24	A	-0.5	3.98	0.748	0.748	ABU63177.1	99	K	VITAFNDGLNHLDSLK	G

Scan	z	Sample ID	Band	PPM	XCorr	Delta correlation	Unique delta correlation	Reference	Redundancy		Peptides	
5035	2	24	B	-2.8	2.69	0.914	0.914	ABU63177.1	99	K	VITAFNDGLNHLDSLK	G
3699	2	24	A	-1.9	3.01	0.603	0.603	JAB00156.1	3	-	VLSGEDKSNIK	A
4553	2	24	B	-4.4	2.93	0	0.26	ABU63177.1	99	K	VNADEVGGEALGR	L
4040	2	24	A	1.73	2.77	0.962	0.962	sp|P02088.2|HBB1_MOUSE	57	K	VNSDEVGGEALGR	L
4023	2	24	A	-1.3	3.73	0.863	0.863	NP_032246.2	99	K	VVAGVAAALAHK	Y
4027	2	24	A	-3.3	3.93	0.856	0.856	NP_032246.2	99	K	VVAGVAAALAHK	Y
4196	2	24	A	0.6	3.02	0.884	0.884	NP_032246.2	99	K	VVAGVAAALAHK	Y
4212	2	24	A	0.68	3.05	0.809	0.809	NP_032246.2	99	K	VVAGVAAALAHK	Y
4633	2	24	B	-0.8	3.4	0.876	0.876	NP_032246.2	99	K	VVAGVAAALAHK	Y
4642	2	24	B	-2.1	3.02	0.848	0.848	NP_032246.2	99	K	VVAGVAAALAHK	Y
4809	2	24	B	-1.4	2.78	0.875	0.875	NP_032246.2	99	K	VVAGVAAALAHK	Y
4814	2	24	B	-1.5	2.89	0.799	0.799	NP_032246.2	99	K	VVAGVAAALAHK	Y
4111	2	24	A	-0.4	3.7	0.898	0.898	NP_032246.2	99	K	VVAGVAAALAHKYH	-
4123	2	24	A	-0.4	3.54	0.914	0.914	NP_032246.2	99	K	VVAGVAAALAHKYH	-
4743	2	24	B	2.1	3.1	0.901	0.901	NP_032246.2	99	K	VVAGVAAALAHKYH	-
4503	2	24	A	1.11	6.21	0.531	0.531	ABU63177.1	99	R	YFDSFGDLSSASAIM*GNAK	V
4514	2	24	A	1.16	6.61	0.492	0.492	ABU63177.1	99	R	YFDSFGDLSSASAIM*GNAK	V
4686	2	24	A	0.29	2.5	0.381	0.381	ABU63177.1	99	R	YFDSFGDLSSASAIM*GNAK	V
4705	2	24	A	0.68	2.84	0.66	0.66	ABU63177.1	99	R	YFDSFGDLSSASAIM*GNAK	V
5140	2	24	B	-1.2	6.03	0.505	0.505	ABU63177.1	99	R	YFDSFGDLSSASAIM*GNAK	V
5141	3	24	B	-2.3	3.94	0.395	0.395	ABU63177.1	99	R	YFDSFGDLSSASAIM*GNAK	V
5322	2	24	B	-0.8	3.28	0.486	0.486	ABU63177.1	99	R	YFDSFGDLSSASAIM*GNAK	V
5330	2	24	B	-1.6	2.98	0.317	0.317	ABU63177.1	99	R	YFDSFGDLSSASAIM*GNAK	V
4707	2	24	A	0.33	5.47	0.523	0.523	ABU63177.1	99	R	YFDSFGDLSSASAIMGNAK	V
4708	3	24	A	0.54	5.38	0.338	0.338	ABU63177.1	99	R	YFDSFGDLSSASAIMGNAK	V
4718	2	24	A	-0.3	5.8	0.509	0.509	ABU63177.1	99	R	YFDSFGDLSSASAIMGNAK	V
4719	3	24	A	-0.2	5.41	0.343	0.343	ABU63177.1	99	R	YFDSFGDLSSASAIMGNAK	V
4899	2	24	A	1.16	4.29	0.712	0.712	ABU63177.1	99	R	YFDSFGDLSSASAIMGNAK	V
4912	2	24	A	-1.7	5.22	0.587	0.587	ABU63177.1	99	R	YFDSFGDLSSASAIMGNAK	V
5336	2	24	A	-0.1	2.91	0.582	0.582	ABU63177.1	99	R	YFDSFGDLSSASAIMGNAK	V
5511	2	24	A	0.19	3.54	0.522	0.522	ABU63177.1	99	R	YFDSFGDLSSASAIMGNAK	V
5520	2	24	A	-0.2	3.45	0.651	0.651	ABU63177.1	99	R	YFDSFGDLSSASAIMGNAK	V
5691	2	24	A	-0.5	3.82	0.585	0.585	ABU63177.1	99	R	YFDSFGDLSSASAIMGNAK	V
5860	2	24	A	-0.2	3.06	0.668	0.668	ABU63177.1	99	R	YFDSFGDLSSASAIMGNAK	V
6185	2	24	A	0.09	3.14	0.545	0.545	ABU63177.1	99	R	YFDSFGDLSSASAIMGNAK	V
6189	2	24	A	-0.1	2.62	0.771	0.771	ABU63177.1	99	R	YFDSFGDLSSASAIMGNAK	V
5338	2	24	B	0.63	5.64	0.594	0.594	ABU63177.1	99	R	YFDSFGDLSSASAIMGNAK	V
5345	2	24	B	-2.8	6.43	0.528	0.528	ABU63177.1	99	R	YFDSFGDLSSASAIMGNAK	V

Scan	z	Sample ID	Band	PPM	XCorr	Delta correlation	Unique delta correlation	Reference	Redundancy		Peptides	
5352	3	24	B	1.87	6.21	0.384	0.384	ABU63177.1	99	R	YFDSFGDLSSASAIMGNAK	V
5363	3	24	B	1.87	4.68	0.346	0.346	ABU63177.1	99	R	YFDSFGDLSSASAIMGNAK	V
5517	2	24	B	1.66	2.72	0.673	0.673	ABU63177.1	99	R	YFDSFGDLSSASAIMGNAK	V
5529	2	24	B	-0.9	5.15	0.569	0.569	ABU63177.1	99	R	YFDSFGDLSSASAIMGNAK	V
5917	2	24	B	-1.3	3.11	0.562	0.562	ABU63177.1	99	R	YFDSFGDLSSASAIMGNAK	V
6100	2	24	B	-2.6	2.96	0.787	0.787	ABU63177.1	99	R	YFDSFGDLSSASAIMGNAK	V
6266	2	24	B	-2	3.09	0.648	0.648	ABU63177.1	99	R	YFDSFGDLSSASAIMGNAK	V
6446	2	24	B	-2.1	3.43	0.586	0.586	ABU63177.1	99	R	YFDSFGDLSSASAIMGNAK	V
6450	2	24	B	-2.8	3.47	0.598	0.598	ABU63177.1	99	R	YFDSFGDLSSASAIMGNAK	V
6625	2	24	B	-2	2.76	0.749	0.749	ABU63177.1	99	R	YFDSFGDLSSASAIMGNAK	V
6806	2	24	B	-1.6	2.81	0.742	0.742	ABU63177.1	99	R	YFDSFGDLSSASAIMGNAK	V
6982	2	24	B	-1.5	2.85	0.658	0.658	ABU63177.1	99	R	YFDSFGDLSSASAIMGNAK	V
5915	2	26	A	-2.4	3.14	0.151	0.151	AJA37790.1	99	R	LLGDILIIVLAAHFSK	D
5924	2	26	A	-2	3.23	0.184	0.184	AJA37790.1	99	R	LLGDILIIVLAAHFSK	D
3554	2	27	B	-1.3	2.56	0.225	0.225	ELK09417.1	99	R	LLVVYPWTQR	F
2725	2	30	B	-0.4	3.33	0.624	0.624	ABU63177.1	99	K	AAVSGLWGK	V
2616	2	30	A	0.57	4	0.899	0.899	CAA32225.1	99	K	DFTPAAQAAFQK	V
2837	2	30	A	0.86	3.56	0	0.291	pdb|1O1N|A	0	K	FLASVSTVLTSK	Y
2888	2	30	B	0.76	2.66	0	0.291	pdb|1O1N|A	0	K	FLASVSTVLTSK	Y
2493	2	30	A	-0.3	4.41	0.105	0.105	EGW10374.1	99	K	IGGHGAEYGAEALER	M
2693	3	30	A	0.45	4.87	0.878	0.878	AAB59723.1	13	K	IGGHGAEYVAEALER	M
2694	2	30	A	0.31	4.03	0.943	0.943	AAB59723.1	13	K	IGGHGAEYVAEALER	M
2804	3	30	B	-0.3	5.15	0.476	0.476	ABU63177.1	99	K	KVITAFNDGLNHLDSLK	G
3005	2	30	A	0.72	3.27	0.191	0.191	ELK09417.1	99	R	LLVVYPWTQR	F
3053	2	30	B	0.12	3.13	0.182	0.182	ELK09417.1	99	R	LLVVYPWTQR	F
2882	2	30	B	0.05	2.56	0.107	0.107	pdb|1O1N|A	99	R	M*FLSFPTTK	T
2550	2	30	B	1	3.95	0	0.258	sp|B3EWD8.1|HBB_TAMHU	0	K	NVADEVGGEALGR^	L
3378	3	30	B	0.41	5.58	0	0.935	XP_004403127.1	14	K	VADALTTAVAHIDDLPGALSALSDLHAYK	L
2853	2	30	A	-0.1	3.62	0.727	0.727	ABU63177.1	99	K	VITAFNDGLNHLDSLK	G
2870	2	30	A	0.44	6.12	0.538	0.538	ABU63177.1	99	R	YFDSFGDLSSASAIM*GNAK	V
3013	2	30	A	0.3	6.2	0.562	0.562	ABU63177.1	99	R	YFDSFGDLSSASAIMGNAK	V
3245	2	54	A	-1.9	2.67	0.632	0.632	ABU63177.1	99	K	AAVSGLWGK	V
3256	2	54	A	-1.9	2.67	0.598	0.598	ABU63177.1	99	K	AAVSGLWGK	V
3233	2	54	B	-1.9	2.78	0.577	0.577	ABU63177.1	99	K	AAVSGLWGK	V
3213	2	54	A	-2.9	4.14	0.896	0.896	CAA32225.1	99	K	DFTPAAQAAFQK	V
3222	2	54	A	-2.9	3.42	0.891	0.891	CAA32225.1	99	K	DFTPAAQAAFQK	V
3201	2	54	B	-1.7	3.81	0.895	0.895	CAA32225.1	99	K	DFTPAAQAAFQK	V
3211	2	54	B	-1.7	4.18	0.898	0.898	CAA32225.1	99	K	DFTPAAQAAFQK	V

Scan	z	Sample ID	Band	PPM	XCorr	Delta correlation	Unique delta correlation	Reference	Redundancy		Peptides	
3409	2	54	A	-3.7	3.54	0	0.364	pdb|1O1N|A	0	K	FLASVSTVLTSK	Y
3387	2	54	B	-1	3.39	0	0.413	pdb|1O1N|A	0	K	FLASVSTVLTSK	Y
3399	2	54	B	-1	3.54	0	0.39	pdb|1O1N|A	0	K	FLASVSTVLTSK	Y
3596	3	54	A	-1.1	9.26	0.121	0.121	ABU63212.1	11	K	IADALASAAGHLDDLPGALSALSDLHAHK	L
3587	3	54	B	-0.8	8.73	0.149	0.149	ABU63212.1	11	K	IADALASAAGHLDDLPGALSALSDLHAHK	L
3277	2	54	A	-1.3	4.72	0.912	0.912	AAB59723.1	13	K	IGGHGAEYVAEALER	M
3288	2	54	A	-1.3	3.94	0.891	0.891	AAB59723.1	13	K	IGGHGAEYVAEALER	M
3255	2	54	B	-1	3.75	0.899	0.899	AAB59723.1	13	K	IGGHGAEYVAEALER	M
3605	3	54	A	-1.3	5.36	0.783	0.783	sp|Q7M2Y4.1|HBA_CHAMP	1	K	KVGDALGNAVAHLDDLPGALSALSDLHAHK	L
3608	3	54	A	-1.3	4.81	0.799	0.799	sp|Q7M2Y4.1|HBA_CHAMP	1	K	KVGDALGNAVAHLDDLPGALSALSDLHAHK	L
3599	3	54	B	-1	4.8	0.738	0.738	sp|Q7M2Y4.1|HBA_CHAMP	1	K	KVGDALGNAVAHLDDLPGALSALSDLHAHK	L
3333	2	54	A	-1.3	5.53	0.542	0.542	ABU63177.1	99	K	KVITAFNDGLNHLDSLK	G
3314	3	54	B	-1.5	5.45	0.538	0.538	ABU63177.1	99	K	KVITAFNDGLNHLDSLK	G
3322	3	54	B	-1.4	5.48	0.574	0.574	ABU63177.1	99	K	KVITAFNDGLNHLDSLK	G
3179	2	54	A	-2	3	0.799	0.799	pdb|1I3R|D	88	K	KVITAFNEGLK	G
3127	3	54	B	-2.1	3.25	0.731	0.731	ERE78771.1	99	K	LHVDPENFR	L
3558	2	54	A	-1.8	3.01	0.854	0.854	CAA32225.1	82	R	LLGNAIVIVLGHHLGK	D
3476	3	54	A	-2.3	3.18	0.173	0.173	ABU63177.1	99	R	LLGNM*IVIVLGHHLGK	D
3465	3	54	B	-0.8	3.6	0.12	0.12	ABU63177.1	99	R	LLGNM*IVIVLGHHLGK	D
3466	2	54	B	-0.8	3.77	0.196	0.196	ABU63177.1	99	R	LLGNM*IVIVLGHHLGK	D
3632	2	54	A	-1.6	4.52	0.161	0.161	ABU63177.1	99	R	LLGNMIVIVLGHHLGK	D
3641	2	54	A	-1.6	4.82	0.17	0.17	ABU63177.1	99	R	LLGNMIVIVLGHHLGK	D
3622	2	54	B	-1.9	3.94	0.243	0.243	ABU63177.1	99	R	LLGNMIVIVLGHHLGK	D
3630	2	54	B	-1.9	3.76	0.176	0.176	ABU63177.1	99	R	LLGNMIVIVLGHHLGK	D
3499	3	54	A	-0.8	3.01	0.174	0.174	ELK09417.1	99	R	LLVVYPWTQR	F
3508	2	54	A	-4.6	2.79	0.197	0.197	ELK09417.1	99	R	LLVVYPWTQR	F
3519	2	54	A	-4.5	2.71	0.293	0.293	ELK09417.1	99	R	LLVVYPWTQR	F
3508	2	54	B	-3.7	2.99	0.19	0.19	ELK09417.1	99	R	LLVVYPWTQR	F
3194	3	54	A	-1.2	3.05	0	0.815	pdb|1O1N|A	0	K	LRVDPVNFK	L
3195	2	54	A	-2.8	2.53	0	0.431	pdb|1O1N|A	0	K	LRVDPVNFK	L
3200	3	54	A	-1.2	3.41	0	0.814	pdb|1O1N|A	0	K	LRVDPVNFK	L
3181	2	54	B	-1.2	2.5	0	0.438	pdb|1O1N|A	0	K	LRVDPVNFK	L
3190	3	54	B	0.72	3.37	0	0.774	pdb|1O1N|A	0	K	LRVDPVNFK	L
3211	2	54	A	-2.6	2.73	0.345	0.345	BAB27277.1	79	R	M*FASFPTTK	T
3178	2	54	B	-1.7	2.92	0.334	0.334	BAB27277.1	79	R	M*FASFPTTK	T
3266	2	54	B	-2.8	2.68	0.4	0.4	BAB27277.1	79	R	MFASFPTTK	T
3234	3	54	A	-2.6	3.22	0.102	0.102	EGW10374.1	99	K	TYFPHFDVSHGSAQVK	A
3246	3	54	A	-2.6	3.45	0.128	0.128	EGW10374.1	99	K	TYFPHFDVSHGSAQVK	A

Scan	z	Sample ID	Band	PPM	XCorr	Delta correlation	Unique delta correlation	Reference	Redundancy		Peptides	
3247	2	54	A	-1.9	4.27	0.11	0.11	EGW10374.1	99	K	TYFPHFDVSHGSAQVK	A
3227	2	54	B	-1.5	3.58	0.122	0.122	EGW10374.1	99	K	TYFPHFDVSHGSAQVK	A
3235	2	54	B	-1.5	4.45	0.106	0.106	EGW10374.1	99	K	TYFPHFDVSHGSAQVK	A
3619	3	54	A	-1.3	4.7	0.817	0.817	sp|Q7M2Y4.1|HBA_CHAMP	1	K	VGDALGNAVAHLDDLPGALSALSDLHAHK	L
3631	3	54	A	-1.3	4.87	0.818	0.818	sp|Q7M2Y4.1|HBA_CHAMP	1	K	VGDALGNAVAHLDDLPGALSALSDLHAHK	L
3610	3	54	B	-1.1	3.8	0.793	0.793	sp|Q7M2Y4.1|HBA_CHAMP	1	K	VGDALGNAVAHLDDLPGALSALSDLHAHK	L
3621	3	54	B	-1.1	3.96	0.803	0.803	sp|Q7M2Y4.1|HBA_CHAMP	1	K	VGDALGNAVAHLDDLPGALSALSDLHAHK	L
2318	2	54	A	-2.2	2.88	0.158	0.158	pdb|3HRW|D	26	-	VHLTDAEK	A
2321	2	54	A	-2.2	2.52	0.182	0.182	pdb|3HRW|D	26	-	VHLTDAEK	A
2455	2	54	A	-3.9	2.73	0.17	0.17	pdb|3HRW|D	26	-	VHLTDAEK	A
2579	2	54	A	-3.3	2.67	0.171	0.171	pdb|3HRW|D	26	-	VHLTDAEK	A
2697	2	54	A	-3.1	2.5	0.143	0.143	pdb|3HRW|D	26	-	VHLTDAEK	A
2699	2	54	A	-3.1	2.63	0.174	0.174	pdb|3HRW|D	26	-	VHLTDAEK	A
2813	2	54	A	-2.4	2.7	0.171	0.171	pdb|3HRW|D	26	-	VHLTDAEK	A
2822	2	54	A	-2.3	2.69	0.171	0.171	pdb|3HRW|D	26	-	VHLTDAEK	A
2676	2	54	B	-1.8	2.51	0.225	0.225	pdb|3HRW|D	26	-	VHLTDAEK	A
2679	2	54	B	-2	2.57	0.141	0.141	pdb|3HRW|D	26	-	VHLTDAEK	A
2789	2	54	B	-2.1	2.7	0.168	0.168	pdb|3HRW|D	26	-	VHLTDAEK	A
2795	2	54	B	-2	2.78	0.178	0.178	pdb|3HRW|D	26	-	VHLTDAEK	A
3363	2	54	A	-1.3	2.92	0.756	0.756	ABU63177.1	99	K	VITAFNDGLNHLDSLK	G
3388	2	54	B	-1.1	4.87	0.616	0.616	ABU63177.1	99	K	VITAFNDGLNHLDSLK	G
3398	2	54	B	-1.1	5.22	0.621	0.621	ABU63177.1	99	K	VITAFNDGLNHLDSLK	G
3125	2	54	B	-1.5	3.31	0.796	0.796	CAA32225.1	62	K	VNPDEVGGEALGR	L
3068	2	54	A	-4.9	4.01	0.923	0.923	sp|P02088.2|HBB1_MOUSE	57	K	VNSDEVGGEALGR	L
4629	2	54	A	-1.4	2.8	0.915	0.915	NP_032246.2	99	K	VVAGVAAALAHK	Y
3135	2	54	B	-2.8	3.81	0.87	0.87	NP_032246.2	99	K	VVAGVAAALAHK	Y
3145	2	54	B	-2.8	3.6	0.876	0.876	NP_032246.2	99	K	VVAGVAAALAHK	Y
3102	2	54	A	-1.3	4	0.711	0.711	XP_011381407.1	99	K	VVAGVATALAHK	Y
3114	2	54	A	-1.3	3.77	0.728	0.728	XP_011381407.1	99	K	VVAGVATALAHK	Y
3091	2	54	B	-4.8	3.66	0.729	0.729	XP_011381407.1	99	K	VVAGVATALAHK	Y
3104	2	54	B	-1.4	2.87	0.698	0.698	XP_011381407.1	99	K	VVAGVATALAHK	Y
3422	2	54	A	-0.8	2.85	0.72	0.72	ABU63177.1	99	R	YFDSFGDLSSASAIM*GNAK	V
3431	2	54	A	-0.8	6.31	0.472	0.472	ABU63177.1	99	R	YFDSFGDLSSASAIM*GNAK	V
3432	3	54	A	-1.1	6.43	0.496	0.496	ABU63177.1	99	R	YFDSFGDLSSASAIM*GNAK	V
3455	3	54	A	-1.1	3.36	0.336	0.336	ABU63177.1	99	R	YFDSFGDLSSASAIM*GNAK	V
3409	2	54	B	-1	5.94	0.535	0.535	ABU63177.1	99	R	YFDSFGDLSSASAIM*GNAK	V
3414	3	54	B	-0.8	3.74	0.477	0.477	ABU63177.1	99	R	YFDSFGDLSSASAIM*GNAK	V
3420	2	54	B	-0.9	6.23	0.489	0.489	ABU63177.1	99	R	YFDSFGDLSSASAIM*GNAK	V

Scan	z	Sample ID	Band	PPM	XCorr	Delta correlation	Unique delta correlation	Reference	Redundancy		Peptides	
3532	2	54	A	-1	6.66	0.547	0.547	ABU63177.1	99	R	YFDSFGDLSSASAIMGNAK	V
3541	2	54	A	-1	5.59	0.515	0.515	ABU63177.1	99	R	YFDSFGDLSSASAIMGNAK	V
3542	3	54	A	-1.3	6.22	0.386	0.386	ABU63177.1	99	R	YFDSFGDLSSASAIMGNAK	V
3553	3	54	A	-1.3	6.3	0.398	0.398	ABU63177.1	99	R	YFDSFGDLSSASAIMGNAK	V
3682	2	54	A	-1.8	2.57	0.742	0.742	ABU63177.1	99	R	YFDSFGDLSSASAIMGNAK	V

z: charge state; #: acrylamidation on C; *: oxidation on M; ^: manually adjusted sequence to VNADEVGGEALGR-see Supplementary data I (Table I, Fig. 1) for details.
