# Supplementary material for: Protein mass spectrometry extends temporal blood meal detection over polymerase chain reaction in mouse-fed Chagas disease vectors
Source: Mem Inst Oswaldo Cruz. 2018 Aug 27;113(10):e180160. doi: 10.1590/0074-02760180160 (PMC6167943; doi:10.1590/0074-02760180160)
Supplement: Supplementary file 3 [file 1678-8060-mioc-113-10-e180160-s3.pdf]

TABLE V
SEQUEST identification of peptides.
Here we show the albumin peptides identified in both experiments, post-feeding and post-molting

Scan	z	Sample ID	Band	PPM	XCorr	Delta correlation	Unique delta correlation	Reference	Redundancy		Peptides	
4097	2	4	F	-2.67	2.598	0.802	0.802	gi|3121749|sp|O35090.1|ALBU_MERUN	26	K	APQVSTPTLVEAAR	S
4879	2	4	F	-1.22	2.751	0.875	0.875	gi|432092344|gb|ELK24962.1|	88	K	DVFLGTFLYEYSR	R
4749	2	4	F	-1.35	2.676	0.891	0.891	gi|432092344|gb|ELK24962.1|	88	K	DVFLGTFLYEYSR	R
4625	2	4	F	-1.56	2.894	0.909	0.909	gi|432092344|gb|ELK24962.1|	88	K	DVFLGTFLYEYSR	R
4276	2	4	F	0.32	3.075	0.971	0.971	gi|5915682|sp|P07724.3|ALBU_MOUSE	16	K	ENPTTFMGHYLHEVAR	R
4615	3	4	F	-1.78	5.618	0.488	0.488	gi|124028612|sp|P02770.2|ALBU_RAT	27	K	GLVLIAFSQYLQK	C
4614	2	4	F	-3.67	5.065	0.534	0.534	gi|124028612|sp|P02770.2|ALBU_RAT	27	K	GLVLIAFSQYLQK	C
4605	2	4	F	-3.46	4.508	0.631	0.631	gi|124028612|sp|P02770.2|ALBU_RAT	27	K	GLVLIAFSQYLQK	C
4289	3	4	F	-0.51	3.82	0	0.762	gi|554561044|ref|XP_005874890.1|	0	R	HPDYSVSLLLR	L
4045	3	4	F	-0.16	4.495	0.808	0.808	gi|731280814|ref|XP_010609182.1|	34	K	KQTALAELVK	H
4038	2	4	F	-1.9	3.014	0.867	0.867	gi|731280814|ref|XP_010609182.1|	34	K	KQTALAELVK	H
4042	2	4	F	-1.9	2.97	0.872	0.872	gi|731280814|ref|XP_010609182.1|	34	K	KQTALAELVK	H
4661	2	4	F	-0.36	3.651	0	0.977	gi|3121749|sp|O35090.1|ALBU_MERUN	3	R	LPC#VEDYLSAILNR	V
4312	2	4	F	-1.43	3.772	0.923	0.923	gi|5915682|sp|P07724.3|ALBU_MOUSE	12	R	LSQTFPNADFAEITK	L
4318	2	4	F	-1.43	4.038	0.931	0.931	gi|5915682|sp|P07724.3|ALBU_MOUSE	12	R	LSQTFPNADFAEITK	L
4098	2	4	F	-3.16	3.289	0.325	0.325	gi|124028612|sp|P02770.2|ALBU_RAT	19	K	LVQEVTDFAK	T
4197	2	4	F	-1.65	2.845	0	0.936	gi|554561044|ref|XP_005874890.1|	0	R	RHPDYSVSLLLR	L
3866	2	4	F	-1.74	3.248	0.811	0.811	gi|5915682|sp|P07724.3|ALBU_MOUSE	15	R	YNDLGEQHFK	G
4296	2	10	F	-4.49	3.605	0.876	0.876	gi|3121749|sp|O35090.1|ALBU_MERUN	26	K	APQVSTPTLVEAAR	S
4791	2	10	F	-1.02	2.505	0.879	0.879	gi|432092344|gb|ELK24962.1|	88	K	DVFLGTFLYEYSR	R
4784	3	10	F	-1.93	3.555	0.923	0.923	gi|432092344|gb|ELK24962.1|	88	K	DVFLGTFLYEYSR	R
4464	2	10	F	-0.89	3.8	0.953	0.953	gi|5915682|sp|P07724.3|ALBU_MOUSE	16	K	ENPTTFMGHYLHEVAR	R
4769	3	10	F	-2.78	5.776	0.518	0.518	gi|124028612|sp|P02770.2|ALBU_RAT	27	K	GLVLIAFSQYLQK	C
4712	2	10	F	-4.41	2.764	0.736	0.736	gi|124028612|sp|P02770.2|ALBU_RAT	27	K	GLVLIAFSQYLQK	C
4472	3	10	F	-0.88	3.885	0	0.818	gi|554561044|ref|XP_005874890.1|	0	R	HPDYSVSLLLR	L
4242	3	10	F	0.22	4.444	0.831	0.831	gi|731280814|ref|XP_010609182.1|	34	K	KQTALAELVK	H
4241	2	10	F	-2.76	2.954	0.903	0.903	gi|731280814|ref|XP_010609182.1|	34	K	KQTALAELVK	H
4549	3	10	F	-0.76	4.995	0	0.855	gi|432092344|gb|ELK24962.1|	15	K	LGEYGFQNALLVR^	Y
4814	3	10	F	-1.26	3.082	0	0.895	gi|3121749|sp|O35090.1|ALBU_MERUN	3	R	LPC#VEDYLSAILNR	V
4813	2	10	F	-0.28	3.349	0	0.938	gi|3121749|sp|O35090.1|ALBU_MERUN	3	R	LPC#VEDYLSAILNR	V
4502	3	10	F	-1.21	3.899	0.742	0.742	gi|5915682|sp|P07724.3|ALBU_MOUSE	12	R	LSQTFPNADFAEITK	L
4508	3	10	F	-1.21	3.871	0.792	0.792	gi|5915682|sp|P07724.3|ALBU_MOUSE	12	R	LSQTFPNADFAEITK	L
4494	2	10	F	-2.75	3.724	0.921	0.921	gi|5915682|sp|P07724.3|ALBU_MOUSE	12	R	LSQTFPNADFAEITK	L
4505	2	10	F	-2.75	4.508	0.931	0.931	gi|5915682|sp|P07724.3|ALBU_MOUSE	12	R	LSQTFPNADFAEITK	L

CC BY

Scan	z	Sample ID	Band	PPM	XCorr	Delta correlation	Unique delta correlation	Reference	Redundancy		Peptides	
4307	2	10	F	-1.98	2.509	0.36	0.36	gi|124028612|sp|P02770.2|ALBU_RAT	19	K	LVQEVTDFAK	T
3506	2	10	F	-1.48	2.511	0.719	0.719	gi|5915682|sp|P07724.3|ALBU_MOUSE	13	K	TPVSEHVTK	C
3638	2	10	F	-1.35	2.532	0.72	0.72	gi|5915682|sp|P07724.3|ALBU_MOUSE	13	K	TPVSEHVTK	C
3992	2	10	F	-1.25	3.375	0.302	0.302	gi|124028612|sp|P02770.2|ALBU_RAT	18	K	YM*C#ENQATISSK	L
4065	2	10	F	-1.51	3.746	0.314	0.314	gi|124028612|sp|P02770.2|ALBU_RAT	18	K	YMC#ENQATISSK	L
4076	3	10	F	-4.41	3.188	0.854	0.854	gi|5915682|sp|P07724.3|ALBU_MOUSE	15	R	YNDLGEQHFK	G
4077	2	10	F	-2.72	3.208	0.856	0.856	gi|5915682|sp|P07724.3|ALBU_MOUSE	15	R	YNDLGEQHFK	G
4088	3	10	F	-4.34	3.19	0.856	0.856	gi|5915682|sp|P07724.3|ALBU_MOUSE	15	R	YNDLGEQHFK	G
4089	2	10	F	-2.72	2.783	0.886	0.886	gi|5915682|sp|P07724.3|ALBU_MOUSE	15	R	YNDLGEQHFK	G
4225	2	11	F	-1.67	4.731	0.931	0.931	gi|5915682|sp|P07724.3|ALBU_MOUSE	11	K	AADKDTC#FSTEGPNLVTR	C
4219	3	11	F	-1.44	5.963	0.937	0.937	gi|5915682|sp|P07724.3|ALBU_MOUSE	11	K	AADKDTC#FSTEGPNLVTR	C
4385	2	11	F	-1.46	3.45	0.945	0.945	gi|5915682|sp|P07724.3|ALBU_MOUSE	12	K	AETFTFHSDIC#TLPEK	E
4306	3	11	F	-1.55	5.444	0.871	0.871	gi|5915682|sp|P07724.3|ALBU_MOUSE	12	K	AETFTFHSDIC#TLPEKEK	Q
4260	2	11	F	-3.22	3.019	0.871	0.871	gi|3121749|sp|O35090.1|ALBU_MERUN	26	K	APQVSTPTLVEAAR	S
4271	2	11	F	-3.22	3.215	0.886	0.886	gi|3121749|sp|O35090.1|ALBU_MERUN	26	K	APQVSTPTLVEAAR	S
5141	2	11	F	-0.66	2.697	0.866	0.866	gi|432092344|gb|ELK24962.1|	88	K	DVFLGTFLYEYSR	R
4766	2	11	F	-2.1	3.006	0.916	0.916	gi|432092344|gb|ELK24962.1|	88	K	DVFLGTFLYEYSR	R
4780	3	11	F	-1.08	4.553	0.938	0.938	gi|432092344|gb|ELK24962.1|	88	K	DVFLGTFLYEYSR	R
4771	3	11	F	-1.08	4.481	0.948	0.948	gi|432092344|gb|ELK24962.1|	88	K	DVFLGTFLYEYSR	R
4177	2	11	F	-2.05	3.054	0.835	0.835	gi|5915682|sp|P07724.3|ALBU_MOUSE	16	K	ENPTTFM*GHYLHEVAR	R
4439	2	11	F	-0.83	4.129	0.92	0.92	gi|5915682|sp|P07724.3|ALBU_MOUSE	16	K	ENPTTFMGHYLHEVAR	R
4436	3	11	F	-1.83	3.114	0.927	0.927	gi|5915682|sp|P07724.3|ALBU_MOUSE	16	K	ENPTTFMGHYLHEVAR	R
4770	3	11	F	-1.67	4.829	0.544	0.544	gi|124028612|sp|P02770.2|ALBU_RAT	27	K	GLVLIAFSQYLQK	C
4756	3	11	F	-1.67	5.52	0.566	0.566	gi|124028612|sp|P02770.2|ALBU_RAT	27	K	GLVLIAFSQYLQK	C
4755	2	11	F	-3.94	4.794	0.58	0.58	gi|124028612|sp|P02770.2|ALBU_RAT	27	K	GLVLIAFSQYLQK	C
4888	2	11	F	-3.89	2.812	0.595	0.595	gi|124028612|sp|P02770.2|ALBU_RAT	27	K	GLVLIAFSQYLQK	C
4441	3	11	F	-0.44	3.782	0	0.774	gi|554561044|ref|XP_005874890.1|	0	R	HPDYSVSLLLR	L
4450	3	11	F	-0.44	4.089	0	0.827	gi|554561044|ref|XP_005874890.1|	0	R	HPDYSVSLLLR	L
4218	3	11	F	0.25	4.484	0.851	0.851	gi|731280814|ref|XP_010609182.1|	34	K	KQTALAELVK	H
4216	2	11	F	-2.96	3.115	0.862	0.862	gi|731280814|ref|XP_010609182.1|	34	K	KQTALAELVK	H
4207	2	11	F	-2.96	3.12	0.9	0.9	gi|731280814|ref|XP_010609182.1|	34	K	KQTALAELVK	H
4525	3	11	F	-0.98	5.183	0	0.869	gi|432092344|gb|ELK24962.1|	15	K	LGEYGFQNALLVR^	Y
4800	3	11	F	-0.98	4.12	0	0.796	gi|3121749|sp|O35090.1|ALBU_MERUN	3	R	LPC#VEDYLSAILNR	V
4799	2	11	F	-0.91	4.797	0	0.933	gi|3121749|sp|O35090.1|ALBU_MERUN	3	R	LPC#VEDYLSAILNR	V
4810	2	11	F	-0.91	3.357	0	0.955	gi|3121749|sp|O35090.1|ALBU_MERUN	3	R	LPC#VEDYLSAILNR	V
4482	3	11	F	-1.24	4.158	0.798	0.798	gi|5915682|sp|P07724.3|ALBU_MOUSE	12	R	LSQTFPNADFAEITK	L
4480	2	11	F	-2.74	4.443	0.932	0.932	gi|5915682|sp|P07724.3|ALBU_MOUSE	12	R	LSQTFPNADFAEITK	L
4469	2	11	F	-2.73	3.459	0.943	0.943	gi|5915682|sp|P07724.3|ALBU_MOUSE	12	R	LSQTFPNADFAEITK	L

Scan	z	Sample ID	Band	PPM	XCorr	Delta correlation	Unique delta correlation	Reference	Redundancy		Peptides	
4272	2	11	F	-3.71	3.12	0.322	0.322	gi|124028612|sp|P02770.2|ALBU_RAT	19	K	LVQEVTDFAK	T
4261	2	11	F	-3.71	2.67	0.349	0.349	gi|124028612|sp|P02770.2|ALBU_RAT	19	K	LVQEVTDFAK	T
4359	2	11	F	-1.66	3.205	0	0.923	gi|554561044|ref|XP_005874890.1|	0	R	RHPDYSVSLLLR	L
4828	3	11	F	-1.2	3.28	0	0.99	gi|554561044|ref|XP_005874890.1|	0	R	RHPDYSVSLLLR	L
4404	3	11	F	-1.01	4.448	0.608	0.608	gi|124028612|sp|P02770.2|ALBU_RAT	18	R	RPC#FSALTVDETYVPK	E
4408	2	11	F	-1.28	3.602	0.621	0.621	gi|124028612|sp|P02770.2|ALBU_RAT	18	R	RPC#FSALTVDETYVPK	E
3988	2	11	F	-2.07	2.541	0.513	0.513	gi|5915682|sp|P07724.3|ALBU_MOUSE	12	K	TNC#DLYEK	L
3584	2	11	F	-1.35	2.81	0.684	0.684	gi|5915682|sp|P07724.3|ALBU_MOUSE	13	K	TPVSEHVTK	C
3712	2	11	F	-1.41	2.765	0.692	0.692	gi|5915682|sp|P07724.3|ALBU_MOUSE	13	K	TPVSEHVTK	C
3848	2	11	F	-2.47	3.021	0.705	0.705	gi|5915682|sp|P07724.3|ALBU_MOUSE	13	K	TPVSEHVTK	C
3581	2	11	F	-1.33	2.784	0.713	0.713	gi|5915682|sp|P07724.3|ALBU_MOUSE	13	K	TPVSEHVTK	C
3857	2	11	F	-2.57	2.785	0.727	0.727	gi|5915682|sp|P07724.3|ALBU_MOUSE	13	K	TPVSEHVTK	C
3715	2	11	F	-1.4	2.58	0.759	0.759	gi|5915682|sp|P07724.3|ALBU_MOUSE	13	K	TPVSEHVTK	C
3445	2	11	F	-1.73	2.678	0.769	0.769	gi|5915682|sp|P07724.3|ALBU_MOUSE	13	K	TPVSEHVTK	C
3448	2	11	F	-1.76	2.65	0.803	0.803	gi|5915682|sp|P07724.3|ALBU_MOUSE	13	K	TPVSEHVTK	C
4119	2	11	F	-3.22	2.672	0.35	0.35	gi|124028612|sp|P02770.2|ALBU_RAT	18	K	YMCENQATISSK	L
4051	3	11	F	-4.15	3.318	0.861	0.861	gi|5915682|sp|P07724.3|ALBU_MOUSE	15	R	YNDLGEQHFK	G
4062	3	11	F	-4.15	3.094	0.869	0.869	gi|5915682|sp|P07724.3|ALBU_MOUSE	15	R	YNDLGEQHFK	G
4052	2	11	F	-3.35	3.504	0.878	0.878	gi|5915682|sp|P07724.3|ALBU_MOUSE	15	R	YNDLGEQHFK	G
4064	2	11	F	-3.33	3.511	0.883	0.883	gi|5915682|sp|P07724.3|ALBU_MOUSE	15	R	YNDLGEQHFK	G
3549	2	15	F	-2.08	3.206	0.853	0.853	gi|3121749|sp|O35090.1|ALBU_MERUN	26	K	APQVSTPTLVEAAR	S
4209	2	15	F	-0.82	2.612	0.871	0.871	gi|432092344|gb|ELK24962.1|	88	K	DVFLGTFLYEYSR	R
4220	2	15	F	-0.78	2.514	0.871	0.871	gi|432092344|gb|ELK24962.1|	88	K	DVFLGTFLYEYSR	R
4088	2	15	F	-0.91	4.116	0.919	0.919	gi|432092344|gb|ELK24962.1|	88	K	DVFLGTFLYEYSR	R
4097	3	15	F	-0.48	3.631	0.963	0.963	gi|432092344|gb|ELK24962.1|	88	K	DVFLGTFLYEYSR	R
3705	2	15	F	1.69	3.421	0.888	0.888	gi|5915682|sp|P07724.3|ALBU_MOUSE	16	K	ENPTTFMGHYLHEVAR	R
3716	2	15	F	1.69	3.086	0.926	0.926	gi|5915682|sp|P07724.3|ALBU_MOUSE	16	K	ENPTTFMGHYLHEVAR	R
4056	3	15	F	-0.91	5.739	0.532	0.532	gi|124028612|sp|P02770.2|ALBU_RAT	27	K	GLVLIAFSQYLQK	C
4055	2	15	F	-1.63	4.975	0.544	0.544	gi|124028612|sp|P02770.2|ALBU_RAT	27	K	GLVLIAFSQYLQK	C
3717	3	15	F	-0.18	3.968	0	0.819	gi|554561044|ref|XP_005874890.1|	0	R	HPDYSVSLLLR	L
3498	3	15	F	0.06	3.95	0.797	0.797	gi|731280814|ref|XP_010609182.1|	34	K	KQTALAELVK	H
3494	2	15	F	-1.79	3.14	0.879	0.879	gi|731280814|ref|XP_010609182.1|	34	K	KQTALAELVK	H
3505	2	15	F	-1.78	2.554	0.89	0.89	gi|731280814|ref|XP_010609182.1|	34	K	KQTALAELVK	H
4211	2	15	F	2.26	2.562	0	0.779	gi|432092344|gb|ELK24962.1|	15	K	LGEYGFQNALLVR^	Y
4111	2	15	F	-1.11	4.134	0	0.895	gi|3121749|sp|O35090.1|ALBU_MERUN	3	R	LPC#VEDYLSAILNR	V
3747	2	15	F	-1.08	4.759	0.93	0.93	gi|5915682|sp|P07724.3|ALBU_MOUSE	12	R	LSQTFPNADFAEITK	L
3550	2	15	F	-1.98	2.846	0.327	0.327	gi|124028612|sp|P02770.2|ALBU_RAT	19	K	LVQEVTDFAK	T
3560	2	15	F	-1.98	3.28	0.335	0.335	gi|124028612|sp|P02770.2|ALBU_RAT	19	K	LVQEVTDFAK	T

Scan	z	Sample ID	Band	PPM	XCorr	Delta correlation	Unique delta correlation	Reference	Redundancy		Peptides	
3637	2	15	F	-1.35	2.758	0	0.899	gi|554561044|ref|XP_005874890.1|	0	R	RHPDYSVSLLLR	L
2948	2	15	F	-1.3	2.556	0.754	0.754	gi|5915682|sp|P07724.3|ALBU_MOUSE	13	K	TPVSEHVTK	C
3309	2	15	F	-0.77	3.548	0.281	0.281	gi|124028612|sp|P02770.2|ALBU_RAT	18	K	YMC#ENQATISSK	L
3319	2	15	F	-1.67	3.074	0.861	0.861	gi|5915682|sp|P07724.3|ALBU_MOUSE	15	R	YNDLGEQHFK	G
3332	2	15	F	-1.67	3.532	0.877	0.877	gi|5915682|sp|P07724.3|ALBU_MOUSE	15	R	YNDLGEQHFK	G
3636	3	17	F	-0.7	3.286	0.818	0.818	gi|5915682|sp|P07724.3|ALBU_MOUSE	12	K	AETFTFHSDIC#TLPEK	E
3638	2	17	F	-1.27	3.459	0.844	0.844	gi|5915682|sp|P07724.3|ALBU_MOUSE	12	K	AETFTFHSDIC#TLPEK	E
3549	3	17	F	0.42	4.117	0.908	0.908	gi|5915682|sp|P07724.3|ALBU_MOUSE	12	K	AETFTFHSDIC#TLPEKEK	Q
3676	2	17	F	2.99	3.109	0.843	0.843	gi|5915682|sp|P07724.3|ALBU_MOUSE	16	K	ENPTTFMGHYLHEVAR	R
3681	2	17	F	3.97	3.588	0.916	0.916	gi|5915682|sp|P07724.3|ALBU_MOUSE	16	K	ENPTTFMGHYLHEVAR	R
4066	3	17	F	-0.53	5.296	0.535	0.535	gi|124028612|sp|P02770.2|ALBU_RAT	27	K	GLVLIAFSQYLQK	C
4188	2	17	F	-1.64	2.858	0.571	0.571	gi|124028612|sp|P02770.2|ALBU_RAT	27	K	GLVLIAFSQYLQK	C
4065	2	17	F	-0.92	4.045	0.578	0.578	gi|124028612|sp|P02770.2|ALBU_RAT	27	K	GLVLIAFSQYLQK	C
3692	3	17	F	0.75	3.698	0	0.805	gi|554561044|ref|XP_005874890.1|	0	R	HPDYSVSLLLR	L
3685	3	17	F	0.75	4.004	0	0.832	gi|554561044|ref|XP_005874890.1|	0	R	HPDYSVSLLLR	L
4142	2	17	F	-4.31	2.685	0	0.907	gi|3121749|sp|O35090.1|ALBU_MERUN	3	R	LPC#VEDYLSAILNR	V
4131	2	17	F	-0.05	3.678	0	0.977	gi|3121749|sp|O35090.1|ALBU_MERUN	3	R	LPC#VEDYLSAILNR	V
3735	2	17	F	0.69	4.048	0.916	0.916	gi|5915682|sp|P07724.3|ALBU_MOUSE	12	R	LSQTFPNADFAEITK	L
4365	2	17	F	-1.17	2.741	0.303	0.303	gi|124028612|sp|P02770.2|ALBU_RAT	19	K	LVQEVTDFAK	T
4377	2	17	F	-1.15	2.819	0.357	0.357	gi|124028612|sp|P02770.2|ALBU_RAT	19	K	LVQEVTDFAK	T
4270	3	17	F	-1	3.821	0	0.834	gi|554561044|ref|XP_005874890.1|	0	R	RHPDYSVSLLLR	L
4282	3	17	F	-1.19	3.46	0	0.909	gi|554561044|ref|XP_005874890.1|	0	R	RHPDYSVSLLLR	L
4396	3	17	F	-1.37	3.81	0	0.997	gi|554561044|ref|XP_005874890.1|	0	R	RHPDYSVSLLLR	L
2915	2	17	F	-1.42	3.091	0.65	0.65	gi|5915682|sp|P07724.3|ALBU_MOUSE	13	K	TPVSEHVTK	C
3031	2	17	F	-1.42	2.755	0.689	0.689	gi|5915682|sp|P07724.3|ALBU_MOUSE	13	K	TPVSEHVTK	C
2792	2	17	F	-2.46	3.093	0.695	0.695	gi|5915682|sp|P07724.3|ALBU_MOUSE	13	K	TPVSEHVTK	C
2665	2	17	F	-3.3	2.799	0.707	0.707	gi|5915682|sp|P07724.3|ALBU_MOUSE	13	K	TPVSEHVTK	C
2788	2	17	F	-2.48	2.901	0.711	0.711	gi|5915682|sp|P07724.3|ALBU_MOUSE	13	K	TPVSEHVTK	C
2911	2	17	F	-1.42	3.081	0.716	0.716	gi|5915682|sp|P07724.3|ALBU_MOUSE	13	K	TPVSEHVTK	C
4428	2	19	F	-1.75	3.355	0.836	0.836	gi|3121749|sp|O35090.1|ALBU_MERUN	26	K	APQVSTPTLVEAAR	S
4417	2	19	F	-1.75	3.278	0.879	0.879	gi|3121749|sp|O35090.1|ALBU_MERUN	26	K	APQVSTPTLVEAAR	S
4597	3	19	F	-0.65	3.847	0	0.817	gi|554561044|ref|XP_005874890.1|	0	R	HPDYSVSLLLR	L
4363	3	19	F	-0.16	4.235	0.833	0.833	gi|731280814|ref|XP_010609182.1|	34	K	KQTALAELVK	H
4679	3	19	F	1.96	4.381	0	0.857	gi|432092344|gb|ELK24962.1|	15	K	LGEYGFQNALLVR^	Y
4670	2	19	F	-0.84	3.206	0	0.912	gi|432092344|gb|ELK24962.1|	15	K	LGEYGFQNALLVR^	Y
4637	2	19	F	-1.41	4.001	0.922	0.922	gi|5915682|sp|P07724.3|ALBU_MOUSE	12	R	LSQTFPNADFAEITK	L
4418	2	19	F	-1.71	3.113	0.328	0.328	gi|124028612|sp|P02770.2|ALBU_RAT	19	K	LVQEVTDFAK	T
4188	2	19	F	-1.5	3.287	0.847	0.847	gi|5915682|sp|P07724.3|ALBU_MOUSE	15	R	YNDLGEQHFK	G

Scan	z	Sample ID	Band	PPM	XCorr	Delta correlation	Unique delta correlation	Reference	Redundancy		Peptides	
5114	2	23	F	1.39	3.784	0.698	0.698	gi|124028612|sp|P02770.2|ALBU_RAT	27	K	GLVLIAFSQYLQK	C
4967	2	24	F	-1.17	2.538	0.904	0.904	gi|432092344|gb|ELK24962.1|	88	K	DVFLGTFLYEYSR	R
3572	2	24	F	-1.24	3.034	0.735	0.735	gi|5915682|sp|P07724.3|ALBU_MOUSE	13	K	TPVSEHVTK	C
3577	2	24	F	-1.25	2.913	0.745	0.745	gi|5915682|sp|P07724.3|ALBU_MOUSE	13	K	TPVSEHVTK	C
3454	2	24	F	-1.36	2.929	0.878	0.878	gi|5915682|sp|P07724.3|ALBU_MOUSE	13	K	TPVSEHVTK	C
4615	2	30	F	-1.28	2.975	0.666	0.666	gi|124028612|sp|P02770.2|ALBU_RAT	27	K	GLVLIAFSQYLQK	C
2264	2	33	F	1.53	3.658	0.846	0.846	gi|3121749|sp|O35090.1|ALBU_MERUN	26	K	APQVSTPTLVEAAR	S
3468	2	33	F	3.29	2.757	0.893	0.893	gi|3121749|sp|O35090.1|ALBU_MERUN	26	K	APQVSTPTLVEAAR	S
3585	2	33	F	-0.19	2.709	0.935	0.935	gi|3121749|sp|O35090.1|ALBU_MERUN	26	K	APQVSTPTLVEAAR	S
3835	2	33	F	0.26	2.548	0.945	0.945	gi|3121749|sp|O35090.1|ALBU_MERUN	26	K	APQVSTPTLVEAAR	S
4337	2	33	F	0.02	2.885	0.95	0.95	gi|3121749|sp|O35090.1|ALBU_MERUN	26	K	APQVSTPTLVEAAR	S
3591	2	33	F	-0.1	2.541	0.951	0.951	gi|3121749|sp|O35090.1|ALBU_MERUN	26	K	APQVSTPTLVEAAR	S
3709	2	33	F	0.19	2.631	0.959	0.959	gi|3121749|sp|O35090.1|ALBU_MERUN	26	K	APQVSTPTLVEAAR	S
3963	2	33	F	0.15	2.502	0.967	0.967	gi|3121749|sp|O35090.1|ALBU_MERUN	26	K	APQVSTPTLVEAAR	S
3434	2	33	F	3.85	3.647	0.55	0.55	gi|432092344|gb|ELK24962.1|	88	K	DVFLGTFLYEYSR	R
3441	2	33	F	3.88	3.592	0.672	0.672	gi|432092344|gb|ELK24962.1|	88	K	DVFLGTFLYEYSR	R
4423	2	33	F	0.12	2.541	0.874	0.874	gi|432092344|gb|ELK24962.1|	88	K	DVFLGTFLYEYSR	R
3984	3	33	F	-0.08	3.16	0.886	0.886	gi|432092344|gb|ELK24962.1|	88	K	DVFLGTFLYEYSR	R
3934	2	33	F	0.51	2.554	0.887	0.887	gi|432092344|gb|ELK24962.1|	88	K	DVFLGTFLYEYSR	R
4430	2	33	F	0.1	3.086	0.896	0.896	gi|432092344|gb|ELK24962.1|	88	K	DVFLGTFLYEYSR	R
4054	2	33	F	-0.06	3.041	0.898	0.898	gi|432092344|gb|ELK24962.1|	88	K	DVFLGTFLYEYSR	R
4169	2	33	F	-0.13	3.154	0.9	0.9	gi|432092344|gb|ELK24962.1|	88	K	DVFLGTFLYEYSR	R
4171	2	33	F	-0.13	3.048	0.901	0.901	gi|432092344|gb|ELK24962.1|	88	K	DVFLGTFLYEYSR	R
3928	2	33	F	0.52	2.837	0.903	0.903	gi|432092344|gb|ELK24962.1|	88	K	DVFLGTFLYEYSR	R
4046	2	33	F	-0.01	3.667	0.905	0.905	gi|432092344|gb|ELK24962.1|	88	K	DVFLGTFLYEYSR	R
4306	2	33	F	0.09	4.323	0.908	0.908	gi|432092344|gb|ELK24962.1|	88	K	DVFLGTFLYEYSR	R
4301	2	33	F	0.06	4.13	0.909	0.909	gi|432092344|gb|ELK24962.1|	88	K	DVFLGTFLYEYSR	R
3717	3	33	F	0.14	3.254	0.919	0.919	gi|432092344|gb|ELK24962.1|	88	K	DVFLGTFLYEYSR	R
3999	3	33	F	-0.06	3.158	0.932	0.932	gi|432092344|gb|ELK24962.1|	88	K	DVFLGTFLYEYSR	R
3851	3	33	F	-0.18	4.106	0.933	0.933	gi|432092344|gb|ELK24962.1|	88	K	DVFLGTFLYEYSR	R
3839	3	33	F	-0.2	3.757	0.949	0.949	gi|432092344|gb|ELK24962.1|	88	K	DVFLGTFLYEYSR	R
3694	3	33	F	0.19	3.543	0.963	0.963	gi|432092344|gb|ELK24962.1|	88	K	DVFLGTFLYEYSR	R
2224	2	33	F	2.87	3.047	0.877	0.877	gi|5915682|sp|P07724.3|ALBU_MOUSE	16	K	ENPTTFM*GHYLHEVAR	R
2214	2	33	F	2.87	2.922	0.911	0.911	gi|5915682|sp|P07724.3|ALBU_MOUSE	16	K	ENPTTFM*GHYLHEVAR	R
2502	3	33	F	1.41	3.094	0.862	0.862	gi|5915682|sp|P07724.3|ALBU_MOUSE	16	K	ENPTTFMGHYLHEVAR	R
2513	3	33	F	1.41	3.106	0.878	0.878	gi|5915682|sp|P07724.3|ALBU_MOUSE	16	K	ENPTTFMGHYLHEVAR	R
2504	2	33	F	1.21	4.479	0.916	0.916	gi|5915682|sp|P07724.3|ALBU_MOUSE	16	K	ENPTTFMGHYLHEVAR	R
2515	2	33	F	1.21	4.332	0.938	0.938	gi|5915682|sp|P07724.3|ALBU_MOUSE	16	K	ENPTTFMGHYLHEVAR	R

Scan	z	Sample ID	Band	PPM	XCorr	Delta correlation	Unique delta correlation	Reference	Redundancy		Peptides	
3630	3	33	F	0.72	3.235	0.938	0.938	gi|3646361|emb|CAA09471.1|	1	K	FIDENTAYLAIIAFSQYVQEASFDEVETLVK	V
3628	3	33	F	0.68	3.416	0.947	0.947	gi|3646361|emb|CAA09471.1|	1	K	FIDENTAYLAIIAFSQYVQEASFDEVETLVK	V
3716	3	33	F	-0.31	3.467	0.385	0.385	gi|124028612|sp|P02770.2|ALBU_RAT	27	K	GLVLIAFSQYLQK	C
3695	3	33	F	-0.41	3.361	0.43	0.43	gi|124028612|sp|P02770.2|ALBU_RAT	27	K	GLVLIAFSQYLQK	C
3837	3	33	F	-0.47	3.598	0.45	0.45	gi|124028612|sp|P02770.2|ALBU_RAT	27	K	GLVLIAFSQYLQK	C
3949	2	33	F	-0.88	4.455	0.571	0.571	gi|124028612|sp|P02770.2|ALBU_RAT	27	K	GLVLIAFSQYLQK	C
3959	2	33	F	-0.85	4.596	0.572	0.572	gi|124028612|sp|P02770.2|ALBU_RAT	27	K	GLVLIAFSQYLQK	C
3834	2	33	F	-0.78	4.529	0.574	0.574	gi|124028612|sp|P02770.2|ALBU_RAT	27	K	GLVLIAFSQYLQK	C
3829	2	33	F	-0.83	4.783	0.578	0.578	gi|124028612|sp|P02770.2|ALBU_RAT	27	K	GLVLIAFSQYLQK	C
3708	2	33	F	-0.59	4.59	0.593	0.593	gi|124028612|sp|P02770.2|ALBU_RAT	27	K	GLVLIAFSQYLQK	C
4071	2	33	F	-1.34	3.704	0.595	0.595	gi|124028612|sp|P02770.2|ALBU_RAT	27	K	GLVLIAFSQYLQK	C
4077	2	33	F	-1.43	4.068	0.602	0.602	gi|124028612|sp|P02770.2|ALBU_RAT	27	K	GLVLIAFSQYLQK	C
3711	2	33	F	-0.62	4.69	0.604	0.604	gi|124028612|sp|P02770.2|ALBU_RAT	27	K	GLVLIAFSQYLQK	C
4410	2	33	F	-2.91	3.401	0.608	0.608	gi|124028612|sp|P02770.2|ALBU_RAT	27	K	GLVLIAFSQYLQK	C
3470	2	33	F	3.55	4.694	0.622	0.622	gi|124028612|sp|P02770.2|ALBU_RAT	27	K	GLVLIAFSQYLQK	C
3461	2	33	F	3.57	3.692	0.685	0.685	gi|124028612|sp|P02770.2|ALBU_RAT	27	K	GLVLIAFSQYLQK	C
2220	3	33	F	4.58	3.718	0.809	0.809	gi|731280814|ref|XP_010609182.1|	34	K	KQTALAELVK	H
2232	3	33	F	0.75	4.361	0.84	0.84	gi|731280814|ref|XP_010609182.1|	34	K	KQTALAELVK	H
2628	3	33	F	1.3	5.73	0	0.87	gi|432092344|gb|ELK24962.1|	15	K	LGEYGFQNALLVR^	Y
4286	2	33	F	4.93	3.553	0	0.884	gi|432092344|gb|ELK24962.1|	15	K	LGEYGFQNALLVR^	Y
2617	3	33	F	-0.31	5.325	0	0.894	gi|432092344|gb|ELK24962.1|	15	K	LGEYGFQNALLVR^	Y
2730	2	33	F	4.09	3.346	0	0.894	gi|432092344|gb|ELK24962.1|	15	K	LGEYGFQNALLVR^	Y
2718	2	33	F	3.91	3.374	0	0.922	gi|432092344|gb|ELK24962.1|	15	K	LGEYGFQNALLVR^	Y
2570	3	33	F	0.25	4.695	0.814	0.814	gi|5915682|sp|P07724.3|ALBU_MOUSE	12	R	LSQTFPNADFAEITK	L
2560	3	33	F	0.25	4.959	0.841	0.841	gi|5915682|sp|P07724.3|ALBU_MOUSE	12	R	LSQTFPNADFAEITK	L
2557	2	33	F	2.59	4.594	0.88	0.88	gi|5915682|sp|P07724.3|ALBU_MOUSE	12	R	LSQTFPNADFAEITK	L
4369	2	33	F	0.14	2.529	0.894	0.894	gi|5915682|sp|P07724.3|ALBU_MOUSE	12	R	LSQTFPNADFAEITK	L
3590	2	33	F	0	2.86	0.914	0.914	gi|5915682|sp|P07724.3|ALBU_MOUSE	12	R	LSQTFPNADFAEITK	L
4366	2	33	F	0.15	2.857	0.916	0.916	gi|5915682|sp|P07724.3|ALBU_MOUSE	12	R	LSQTFPNADFAEITK	L
3586	2	33	F	-0.14	3.112	0.922	0.922	gi|5915682|sp|P07724.3|ALBU_MOUSE	12	R	LSQTFPNADFAEITK	L
2546	2	33	F	2.47	4.83	0.934	0.934	gi|5915682|sp|P07724.3|ALBU_MOUSE	12	R	LSQTFPNADFAEITK	L
4250	2	33	F	-0.03	2.506	0.29	0.29	gi|124028612|sp|P02770.2|ALBU_RAT	19	K	LVQEVTDFAK	T
3624	2	33	F	-0.33	2.799	0.295	0.295	gi|124028612|sp|P02770.2|ALBU_RAT	19	K	LVQEVTDFAK	T
3620	2	33	F	-0.32	2.575	0.35	0.35	gi|124028612|sp|P02770.2|ALBU_RAT	19	K	LVQEVTDFAK	T
2284	2	33	F	3.86	2.708	0.369	0.369	gi|124028612|sp|P02770.2|ALBU_RAT	19	K	LVQEVTDFAK	T
4247	3	33	F	-0.36	3.234	0	0.9	gi|554561044|ref|XP_005874890.1|	0	R	RHPDYSVSLLLR	L
3970	3	33	F	-0.07	3.693	0	0.915	gi|554561044|ref|XP_005874890.1|	0	R	RHPDYSVSLLLR	L
3715	3	33	F	-0.08	3.782	0	0.919	gi|554561044|ref|XP_005874890.1|	0	R	RHPDYSVSLLLR	L

Scan	z	Sample ID	Band	PPM	XCorr	Delta correlation	Unique delta correlation	Reference	Redundancy		Peptides	
2487	3	33	F	0.98	3.094	0	0.924	gi|554561044|ref|XP_005874890.1|	0	R	RHPDYSVSLLLR	L
3836	3	33	F	-0.27	3.408	0	0.932	gi|554561044|ref|XP_005874890.1|	0	R	RHPDYSVSLLLR	L
3844	3	33	F	-0.24	3.913	0	0.935	gi|554561044|ref|XP_005874890.1|	0	R	RHPDYSVSLLLR	L
3693	3	33	F	-0.3	3.633	0	0.936	gi|554561044|ref|XP_005874890.1|	0	R	RHPDYSVSLLLR	L
3962	3	33	F	-0.05	3.715	0	0.937	gi|554561044|ref|XP_005874890.1|	0	R	RHPDYSVSLLLR	L
2381	2	33	F	0.31	3.257	0	0.976	gi|554561044|ref|XP_005874890.1|	0	R	RHPDYSVSLLLR	L
2392	2	33	F	0.3	3.307	0	0.985	gi|554561044|ref|XP_005874890.1|	0	R	RHPDYSVSLLLR	L
2485	2	33	F	-0.89	3.443	0.579	0.579	gi|124028612|sp|P02770.2|ALBU_RAT	18	R	RPC#FSALTVDETYVPK	E
1843	2	33	F	-0.24	3.074	0.641	0.641	gi|5915682|sp|P07724.3|ALBU_MOUSE	13	K	TPVSEHVTK	C
1720	2	33	F	1.86	3.165	0.659	0.659	gi|5915682|sp|P07724.3|ALBU_MOUSE	13	K	TPVSEHVTK	C
1593	2	33	F	-0.38	2.67	0.66	0.66	gi|5915682|sp|P07724.3|ALBU_MOUSE	13	K	TPVSEHVTK	C
1597	2	33	F	-0.45	2.651	0.66	0.66	gi|5915682|sp|P07724.3|ALBU_MOUSE	13	K	TPVSEHVTK	C
1971	2	33	F	-0.52	2.797	0.673	0.673	gi|5915682|sp|P07724.3|ALBU_MOUSE	13	K	TPVSEHVTK	C
1845	2	33	F	-0.25	2.93	0.677	0.677	gi|5915682|sp|P07724.3|ALBU_MOUSE	13	K	TPVSEHVTK	C
1973	2	33	F	-0.53	2.808	0.682	0.682	gi|5915682|sp|P07724.3|ALBU_MOUSE	13	K	TPVSEHVTK	C
1718	2	33	F	1.89	2.844	0.686	0.686	gi|5915682|sp|P07724.3|ALBU_MOUSE	13	K	TPVSEHVTK	C
2079	2	33	F	-0.66	3.514	0.351	0.351	gi|124028612|sp|P02770.2|ALBU_RAT	18	K	YMC#ENQATISSK	L
2113	2	33	F	1.11	2.759	0.349	0.349	gi|124028612|sp|P02770.2|ALBU_RAT	18	K	YMCENQATISSK	L
2087	2	33	F	0.09	3.262	0.862	0.862	gi|5915682|sp|P07724.3|ALBU_MOUSE	15	R	YNDLGEQHFK	G
2076	2	33	F	0.09	3.538	0.87	0.87	gi|5915682|sp|P07724.3|ALBU_MOUSE	15	R	YNDLGEQHFK	G
4378	2	34	F	-0.06	2.638	0.929	0.929	gi|3121749|sp|O35090.1|ALBU_MERUN	26	K	APQVSTPTLVEAAR	S
4383	2	34	F	0.08	2.579	0.976	0.976	gi|3121749|sp|O35090.1|ALBU_MERUN	26	K	APQVSTPTLVEAAR	S
3545	3	34	F	3.17	3.423	0.423	0.423	gi|432092344|gb|ELK24962.1|	88	K	DVFLGTFLYEYSR	R
3517	3	34	F	2.63	4.571	0.513	0.513	gi|432092344|gb|ELK24962.1|	88	K	DVFLGTFLYEYSR	R
3746	2	34	F	0.35	3.478	0.899	0.899	gi|432092344|gb|ELK24962.1|	88	K	DVFLGTFLYEYSR	R
3876	2	34	F	0.27	3.465	0.909	0.909	gi|432092344|gb|ELK24962.1|	88	K	DVFLGTFLYEYSR	R
4239	2	34	F	0.16	3.151	0.909	0.909	gi|432092344|gb|ELK24962.1|	88	K	DVFLGTFLYEYSR	R
3874	2	34	F	0.34	3.435	0.91	0.91	gi|432092344|gb|ELK24962.1|	88	K	DVFLGTFLYEYSR	R
3248	2	34	F	0.6	3.514	0.912	0.912	gi|432092344|gb|ELK24962.1|	88	K	DVFLGTFLYEYSR	R
3380	2	34	F	0.6	2.957	0.912	0.912	gi|432092344|gb|ELK24962.1|	88	K	DVFLGTFLYEYSR	R
3819	3	34	F	0.12	3.563	0.913	0.913	gi|432092344|gb|ELK24962.1|	88	K	DVFLGTFLYEYSR	R
4380	2	34	F	0.24	3.546	0.913	0.913	gi|432092344|gb|ELK24962.1|	88	K	DVFLGTFLYEYSR	R
3506	2	34	F	0.64	3.622	0.914	0.914	gi|432092344|gb|ELK24962.1|	88	K	DVFLGTFLYEYSR	R
3624	2	34	F	0.56	3.618	0.914	0.914	gi|432092344|gb|ELK24962.1|	88	K	DVFLGTFLYEYSR	R
3743	2	34	F	0.35	3.135	0.915	0.915	gi|432092344|gb|ELK24962.1|	88	K	DVFLGTFLYEYSR	R
4116	2	34	F	0.02	3.157	0.915	0.915	gi|432092344|gb|ELK24962.1|	88	K	DVFLGTFLYEYSR	R
3085	2	34	F	0.48	3.939	0.918	0.918	gi|432092344|gb|ELK24962.1|	88	K	DVFLGTFLYEYSR	R
3258	2	34	F	0.8	3.812	0.918	0.918	gi|432092344|gb|ELK24962.1|	88	K	DVFLGTFLYEYSR	R

Scan	z	Sample ID	Band	PPM	XCorr	Delta correlation	Unique delta correlation	Reference	Redundancy		Peptides	
3992	2	34	F	0.27	3.472	0.918	0.918	gi|432092344|gb|ELK24962.1|	88	K	DVFLGTFLYEYSR	R
4376	2	34	F	0.31	3.363	0.918	0.918	gi|432092344|gb|ELK24962.1|	88	K	DVFLGTFLYEYSR	R
3622	2	34	F	0.6	3.614	0.919	0.919	gi|432092344|gb|ELK24962.1|	88	K	DVFLGTFLYEYSR	R
3814	3	34	F	0.14	3.254	0.922	0.922	gi|432092344|gb|ELK24962.1|	88	K	DVFLGTFLYEYSR	R
3372	2	34	F	0.61	3.322	0.923	0.923	gi|432092344|gb|ELK24962.1|	88	K	DVFLGTFLYEYSR	R
3999	2	34	F	0.22	3.272	0.923	0.923	gi|432092344|gb|ELK24962.1|	88	K	DVFLGTFLYEYSR	R
2952	2	34	F	1.52	4.131	0.924	0.924	gi|432092344|gb|ELK24962.1|	88	K	DVFLGTFLYEYSR	R
3096	2	34	F	0.38	4.228	0.924	0.924	gi|432092344|gb|ELK24962.1|	88	K	DVFLGTFLYEYSR	R
4109	2	34	F	0.01	3.247	0.924	0.924	gi|432092344|gb|ELK24962.1|	88	K	DVFLGTFLYEYSR	R
3950	3	34	F	-0.18	3.382	0.928	0.928	gi|432092344|gb|ELK24962.1|	88	K	DVFLGTFLYEYSR	R
4645	3	34	F	-1.28	3.245	0.928	0.928	gi|432092344|gb|ELK24962.1|	88	K	DVFLGTFLYEYSR	R
3943	3	34	F	-0.22	3.366	0.933	0.933	gi|432092344|gb|ELK24962.1|	88	K	DVFLGTFLYEYSR	R
4242	2	34	F	0.13	4.007	0.933	0.933	gi|432092344|gb|ELK24962.1|	88	K	DVFLGTFLYEYSR	R
3499	2	34	F	0.5	4.223	0.937	0.937	gi|432092344|gb|ELK24962.1|	88	K	DVFLGTFLYEYSR	R
3232	3	34	F	2.03	3.711	0.944	0.944	gi|432092344|gb|ELK24962.1|	88	K	DVFLGTFLYEYSR	R
3002	3	34	F	0.3	5.099	0.948	0.948	gi|432092344|gb|ELK24962.1|	88	K	DVFLGTFLYEYSR	R
4068	3	34	F	-0.37	3.411	0.95	0.95	gi|432092344|gb|ELK24962.1|	88	K	DVFLGTFLYEYSR	R
3691	3	34	F	-0.42	3.498	0.957	0.957	gi|432092344|gb|ELK24962.1|	88	K	DVFLGTFLYEYSR	R
86	2	34	F	-0.47	2.665	0.964	0.964	gi|432092344|gb|ELK24962.1|	88	K	DVFLGTFLYEYSR	R
2529	3	34	F	1.18	3.22	0.88	0.88	gi|5915682|sp|P07724.3|ALBU_MOUSE	16	K	ENPTTFMGHYLHEVAR	R
2530	2	34	F	1.41	4.281	0.93	0.93	gi|5915682|sp|P07724.3|ALBU_MOUSE	16	K	ENPTTFMGHYLHEVAR	R
2539	2	34	F	1.41	4.46	0.935	0.935	gi|5915682|sp|P07724.3|ALBU_MOUSE	16	K	ENPTTFMGHYLHEVAR	R
3673	3	34	F	0.34	3.112	0.413	0.413	gi|124028612|sp|P02770.2|ALBU_RAT	27	K	GLVLIAFSQYLQK	C
3547	3	34	F	3.31	4.079	0.414	0.414	gi|124028612|sp|P02770.2|ALBU_RAT	27	K	GLVLIAFSQYLQK	C
3518	3	34	F	2.16	3.626	0.421	0.421	gi|124028612|sp|P02770.2|ALBU_RAT	27	K	GLVLIAFSQYLQK	C
3692	3	34	F	-0.2	3.185	0.449	0.449	gi|124028612|sp|P02770.2|ALBU_RAT	27	K	GLVLIAFSQYLQK	C
2943	3	34	F	2.87	6.163	0.497	0.497	gi|124028612|sp|P02770.2|ALBU_RAT	27	K	GLVLIAFSQYLQK	C
3286	3	34	F	1.03	4.333	0.503	0.503	gi|124028612|sp|P02770.2|ALBU_RAT	27	K	GLVLIAFSQYLQK	C
2932	3	34	F	2.87	5.677	0.527	0.527	gi|124028612|sp|P02770.2|ALBU_RAT	27	K	GLVLIAFSQYLQK	C
3055	2	34	F	4.19	3.967	0.576	0.576	gi|124028612|sp|P02770.2|ALBU_RAT	27	K	GLVLIAFSQYLQK	C
3562	2	34	F	-1.02	3.907	0.577	0.577	gi|124028612|sp|P02770.2|ALBU_RAT	27	K	GLVLIAFSQYLQK	C
3173	2	34	F	-0.86	3.241	0.587	0.587	gi|124028612|sp|P02770.2|ALBU_RAT	27	K	GLVLIAFSQYLQK	C
3682	2	34	F	-1.58	3.792	0.587	0.587	gi|124028612|sp|P02770.2|ALBU_RAT	27	K	GLVLIAFSQYLQK	C
4047	2	34	F	-2.77	3.073	0.588	0.588	gi|124028612|sp|P02770.2|ALBU_RAT	27	K	GLVLIAFSQYLQK	C
3806	2	34	F	-1.31	3.578	0.589	0.589	gi|124028612|sp|P02770.2|ALBU_RAT	27	K	GLVLIAFSQYLQK	C
4367	2	34	F	-2.84	2.993	0.591	0.591	gi|124028612|sp|P02770.2|ALBU_RAT	27	K	GLVLIAFSQYLQK	C
3429	2	34	F	-0.71	3.922	0.592	0.592	gi|124028612|sp|P02770.2|ALBU_RAT	27	K	GLVLIAFSQYLQK	C
3557	2	34	F	-0.97	4.01	0.595	0.595	gi|124028612|sp|P02770.2|ALBU_RAT	27	K	GLVLIAFSQYLQK	C

Scan	z	Sample ID	Band	PPM	XCorr	Delta correlation	Unique delta correlation	Reference	Redundancy		Peptides	
3427	2	34	F	-0.71	3.66	0.602	0.602	gi|124028612|sp|P02770.2|ALBU_RAT	27	K	GLVLIAFSQYLQK	C
3935	2	34	F	-2.02	3.456	0.608	0.608	gi|124028612|sp|P02770.2|ALBU_RAT	27	K	GLVLIAFSQYLQK	C
3684	2	34	F	-1.5	3.843	0.611	0.611	gi|124028612|sp|P02770.2|ALBU_RAT	27	K	GLVLIAFSQYLQK	C
3314	2	34	F	3.51	3.391	0.614	0.614	gi|124028612|sp|P02770.2|ALBU_RAT	27	K	GLVLIAFSQYLQK	C
3808	2	34	F	-1.41	3.771	0.615	0.615	gi|124028612|sp|P02770.2|ALBU_RAT	27	K	GLVLIAFSQYLQK	C
4362	2	34	F	-3.11	3.024	0.615	0.615	gi|124028612|sp|P02770.2|ALBU_RAT	27	K	GLVLIAFSQYLQK	C
4612	2	34	F	-0.46	2.646	0.617	0.617	gi|124028612|sp|P02770.2|ALBU_RAT	27	K	GLVLIAFSQYLQK	C
3044	2	34	F	4.19	3.604	0.62	0.62	gi|124028612|sp|P02770.2|ALBU_RAT	27	K	GLVLIAFSQYLQK	C
3180	2	34	F	-1.06	3.576	0.638	0.638	gi|124028612|sp|P02770.2|ALBU_RAT	27	K	GLVLIAFSQYLQK	C
3930	2	34	F	-1.96	3.544	0.642	0.642	gi|124028612|sp|P02770.2|ALBU_RAT	27	K	GLVLIAFSQYLQK	C
4614	2	34	F	-0.42	2.558	0.661	0.661	gi|124028612|sp|P02770.2|ALBU_RAT	27	K	GLVLIAFSQYLQK	C
4055	2	34	F	-2.83	2.948	0.665	0.665	gi|124028612|sp|P02770.2|ALBU_RAT	27	K	GLVLIAFSQYLQK	C
2519	3	34	F	4.27	3.962	0	0.801	gi|554561044|ref|XP_005874890.1|	0	R	HPDYSVSLLLR	L
2243	3	34	F	1.64	4.298	0.809	0.809	gi|731280814|ref|XP_010609182.1|	34	K	KQTALAELVK	H
2232	3	34	F	1.64	4.359	0.846	0.846	gi|731280814|ref|XP_010609182.1|	34	K	KQTALAELVK	H
2231	2	34	F	1.45	2.859	0.866	0.866	gi|731280814|ref|XP_010609182.1|	34	K	KQTALAELVK	H
2242	2	34	F	1.45	3.595	0.898	0.898	gi|731280814|ref|XP_010609182.1|	34	K	KQTALAELVK	H
2637	3	34	F	3.6	5.325	0	0.861	gi|432092344|gb|ELK24962.1|	15	K	LGEYGFQNALLVR^	Y
2747	2	34	F	0.01	3.3	0	0.918	gi|432092344|gb|ELK24962.1|	15	K	LGEYGFQNALLVR^	Y
2763	2	34	F	3.9	3.466	0	0.922	gi|432092344|gb|ELK24962.1|	15	K	LGEYGFQNALLVR^	Y
4491	2	34	F	1.39	2.724	0	0.925	gi|432092344|gb|ELK24962.1|	15	K	LGEYGFQNALLVR^	Y
3027	2	34	F	1.07	4.648	0	0.94	gi|3121749|sp|O35090.1|ALBU_MERUN	3	R	LPC#VEDYLSAILNR	V
2574	3	34	F	0.74	4.07	0.791	0.791	gi|5915682|sp|P07724.3|ALBU_MOUSE	12	R	LSQTFPNADFAEITK	L
2584	3	34	F	1.3	4.701	0.829	0.829	gi|5915682|sp|P07724.3|ALBU_MOUSE	12	R	LSQTFPNADFAEITK	L
2583	2	34	F	3.89	5.026	0.939	0.939	gi|5915682|sp|P07724.3|ALBU_MOUSE	12	R	LSQTFPNADFAEITK	L
2286	2	34	F	1.93	2.95	0.337	0.337	gi|124028612|sp|P02770.2|ALBU_RAT	19	K	LVQEVTDFAK	T
3549	3	34	F	0.73	3.639	0	0.905	gi|554561044|ref|XP_005874890.1|	0	R	RHPDYSVSLLLR	L
3820	3	34	F	-0.2	3.666	0	0.914	gi|554561044|ref|XP_005874890.1|	0	R	RHPDYSVSLLLR	L
3693	3	34	F	-0.42	4.077	0	0.918	gi|554561044|ref|XP_005874890.1|	0	R	RHPDYSVSLLLR	L
3674	3	34	F	-0.56	3.277	0	0.92	gi|554561044|ref|XP_005874890.1|	0	R	RHPDYSVSLLLR	L
3942	3	34	F	-0.27	3.369	0	0.923	gi|554561044|ref|XP_005874890.1|	0	R	RHPDYSVSLLLR	L
3817	3	34	F	-0.25	3.371	0	0.928	gi|554561044|ref|XP_005874890.1|	0	R	RHPDYSVSLLLR	L
4399	3	34	F	-0.1	3.791	0	0.939	gi|554561044|ref|XP_005874890.1|	0	R	RHPDYSVSLLLR	L
2407	2	34	F	-0.36	3.263	0	0.943	gi|554561044|ref|XP_005874890.1|	0	R	RHPDYSVSLLLR	L
2418	2	34	F	-0.36	3.479	0	0.99	gi|554561044|ref|XP_005874890.1|	0	R	RHPDYSVSLLLR	L
2501	2	34	F	3.49	2.74	0.943	0.943	gi|124028612|sp|P02770.2|ALBU_RAT	18	R	RPC#FSALTVDETYVPK	E
1762	2	34	F	-0.89	2.972	0.646	0.646	gi|5915682|sp|P07724.3|ALBU_MOUSE	13	K	TPVSEHVTK	C
1760	2	34	F	-0.9	3.013	0.657	0.657	gi|5915682|sp|P07724.3|ALBU_MOUSE	13	K	TPVSEHVTK	C

Scan	z	Sample ID	Band	PPM	XCorr	Delta correlation	Unique delta correlation	Reference	Redundancy		Peptides	
1886	2	34	F	-1.04	2.651	0.68	0.68	gi|5915682|sp|P07724.3|ALBU_MOUSE	13	K	TPVSEHVTK	C
1631	2	34	F	0.43	2.863	0.682	0.682	gi|5915682|sp|P07724.3|ALBU_MOUSE	13	K	TPVSEHVTK	C
1636	2	34	F	0.51	2.85	0.698	0.698	gi|5915682|sp|P07724.3|ALBU_MOUSE	13	K	TPVSEHVTK	C
2077	3	34	F	1.34	3.074	0.847	0.847	gi|5915682|sp|P07724.3|ALBU_MOUSE	15	R	YNDLGEQHFK	G
2089	2	34	F	3.55	3.671	0.875	0.875	gi|5915682|sp|P07724.3|ALBU_MOUSE	15	R	YNDLGEQHFK	G
2078	2	34	F	-0.14	3.652	0.876	0.876	gi|5915682|sp|P07724.3|ALBU_MOUSE	15	R	YNDLGEQHFK	G
2215	3	35	F	2.81	4.742	0.852	0.852	gi|5915682|sp|P07724.3|ALBU_MOUSE	12	K	AETFTFHSDIC#TLPEKEK	Q
2112	3	35	F	0.58	4.195	0.632	0.632	gi|3121749|sp|O35090.1|ALBU_MERUN	26	K	APQVSTPTLVEAAR	S
2122	3	35	F	0.94	4.482	0.665	0.665	gi|3121749|sp|O35090.1|ALBU_MERUN	26	K	APQVSTPTLVEAAR	S
2108	2	35	F	1.6	3.642	0.86	0.86	gi|3121749|sp|O35090.1|ALBU_MERUN	26	K	APQVSTPTLVEAAR	S
2082	2	35	F	0.16	2.668	0.866	0.866	gi|3121749|sp|O35090.1|ALBU_MERUN	26	K	APQVSTPTLVEAAR	S
2765	3	35	F	3.85	5.182	0.429	0.429	gi|432092344|gb|ELK24962.1|	88	K	DVFLGTFLYEYSR	R
3073	3	35	F	3.81	4.575	0.485	0.485	gi|432092344|gb|ELK24962.1|	88	K	DVFLGTFLYEYSR	R
3141	2	35	F	4.78	3.195	0.499	0.499	gi|432092344|gb|ELK24962.1|	88	K	DVFLGTFLYEYSR	R
3968	2	35	F	-0.29	3.408	0.899	0.899	gi|432092344|gb|ELK24962.1|	88	K	DVFLGTFLYEYSR	R
3535	2	35	F	-0.16	4.352	0.904	0.904	gi|432092344|gb|ELK24962.1|	88	K	DVFLGTFLYEYSR	R
4394	2	35	F	-0.19	3.429	0.905	0.905	gi|432092344|gb|ELK24962.1|	88	K	DVFLGTFLYEYSR	R
3258	2	35	F	-0.2	3.394	0.906	0.906	gi|432092344|gb|ELK24962.1|	88	K	DVFLGTFLYEYSR	R
3372	3	35	F	-0.62	3.297	0.906	0.906	gi|432092344|gb|ELK24962.1|	88	K	DVFLGTFLYEYSR	R
3532	2	35	F	-0.22	3.607	0.907	0.907	gi|432092344|gb|ELK24962.1|	88	K	DVFLGTFLYEYSR	R
3839	2	35	F	0.22	3.311	0.908	0.908	gi|432092344|gb|ELK24962.1|	88	K	DVFLGTFLYEYSR	R
4093	2	35	F	0.13	3.258	0.909	0.909	gi|432092344|gb|ELK24962.1|	88	K	DVFLGTFLYEYSR	R
2875	2	35	F	0.1	3.5	0.91	0.91	gi|432092344|gb|ELK24962.1|	88	K	DVFLGTFLYEYSR	R
4391	2	35	F	-0.01	3.227	0.91	0.91	gi|432092344|gb|ELK24962.1|	88	K	DVFLGTFLYEYSR	R
3006	2	35	F	0.08	3.369	0.913	0.913	gi|432092344|gb|ELK24962.1|	88	K	DVFLGTFLYEYSR	R
3260	2	35	F	-0.16	3.214	0.913	0.913	gi|432092344|gb|ELK24962.1|	88	K	DVFLGTFLYEYSR	R
3961	2	35	F	-0.34	3.36	0.913	0.913	gi|432092344|gb|ELK24962.1|	88	K	DVFLGTFLYEYSR	R
3841	2	35	F	0.09	3.168	0.914	0.914	gi|432092344|gb|ELK24962.1|	88	K	DVFLGTFLYEYSR	R
3388	2	35	F	0.01	3.715	0.915	0.915	gi|432092344|gb|ELK24962.1|	88	K	DVFLGTFLYEYSR	R
3394	2	35	F	0.1	4.174	0.915	0.915	gi|432092344|gb|ELK24962.1|	88	K	DVFLGTFLYEYSR	R
3132	2	35	F	-0.01	3.08	0.916	0.916	gi|432092344|gb|ELK24962.1|	88	K	DVFLGTFLYEYSR	R
3807	3	35	F	-0.58	3.189	0.916	0.916	gi|432092344|gb|ELK24962.1|	88	K	DVFLGTFLYEYSR	R
3702	2	35	F	-0.07	2.645	0.919	0.919	gi|432092344|gb|ELK24962.1|	88	K	DVFLGTFLYEYSR	R
3001	2	35	F	0.02	4.625	0.92	0.92	gi|432092344|gb|ELK24962.1|	88	K	DVFLGTFLYEYSR	R
3241	3	35	F	-0.93	3.743	0.921	0.921	gi|432092344|gb|ELK24962.1|	88	K	DVFLGTFLYEYSR	R
2755	2	35	F	1.2	2.883	0.922	0.922	gi|432092344|gb|ELK24962.1|	88	K	DVFLGTFLYEYSR	R
4096	2	35	F	0.11	3.451	0.922	0.922	gi|432092344|gb|ELK24962.1|	88	K	DVFLGTFLYEYSR	R
2880	2	35	F	0.17	3.813	0.924	0.924	gi|432092344|gb|ELK24962.1|	88	K	DVFLGTFLYEYSR	R

Scan	z	Sample ID	Band	PPM	XCorr	Delta correlation	Unique delta correlation	Reference	Redundancy		Peptides	
3670	3	35	F	-0.64	3.371	0.927	0.927	gi|432092344|gb|ELK24962.1|	88	K	DVFLGTFLYEYSR	R
3533	3	35	F	-0.43	3.537	0.928	0.928	gi|432092344|gb|ELK24962.1|	88	K	DVFLGTFLYEYSR	R
4248	2	35	F	0.21	3.624	0.928	0.928	gi|432092344|gb|ELK24962.1|	88	K	DVFLGTFLYEYSR	R
3098	3	35	F	2.37	3.497	0.929	0.929	gi|432092344|gb|ELK24962.1|	88	K	DVFLGTFLYEYSR	R
3369	3	35	F	-0.47	4.315	0.934	0.934	gi|432092344|gb|ELK24962.1|	88	K	DVFLGTFLYEYSR	R
3667	3	35	F	-0.57	3.234	0.937	0.937	gi|432092344|gb|ELK24962.1|	88	K	DVFLGTFLYEYSR	R
3809	3	35	F	-0.68	3.437	0.938	0.938	gi|432092344|gb|ELK24962.1|	88	K	DVFLGTFLYEYSR	R
3945	3	35	F	-0.39	3.053	0.938	0.938	gi|432092344|gb|ELK24962.1|	88	K	DVFLGTFLYEYSR	R
2915	3	35	F	0.78	4.243	0.939	0.939	gi|432092344|gb|ELK24962.1|	88	K	DVFLGTFLYEYSR	R
3530	3	35	F	-0.59	3.821	0.939	0.939	gi|432092344|gb|ELK24962.1|	88	K	DVFLGTFLYEYSR	R
3225	3	35	F	-0.5	3.609	0.946	0.946	gi|432092344|gb|ELK24962.1|	88	K	DVFLGTFLYEYSR	R
2756	3	35	F	-0.63	4.935	0.948	0.948	gi|432092344|gb|ELK24962.1|	88	K	DVFLGTFLYEYSR	R
4250	2	35	F	0.21	2.703	0.966	0.966	gi|432092344|gb|ELK24962.1|	88	K	DVFLGTFLYEYSR	R
3403	3	35	F	-0.35	3.283	0.425	0.425	gi|124028612|sp|P02770.2|ALBU_RAT	27	K	GLVLIAFSQYLQK	C
2917	3	35	F	2.4	3.785	0.451	0.451	gi|124028612|sp|P02770.2|ALBU_RAT	27	K	GLVLIAFSQYLQK	C
2742	3	35	F	-0.22	5.915	0.476	0.476	gi|124028612|sp|P02770.2|ALBU_RAT	27	K	GLVLIAFSQYLQK	C
3099	3	35	F	2.97	3.966	0.51	0.51	gi|124028612|sp|P02770.2|ALBU_RAT	27	K	GLVLIAFSQYLQK	C
2744	3	35	F	-0.22	6.054	0.55	0.55	gi|124028612|sp|P02770.2|ALBU_RAT	27	K	GLVLIAFSQYLQK	C
2955	2	35	F	-1.56	2.77	0.552	0.552	gi|124028612|sp|P02770.2|ALBU_RAT	27	K	GLVLIAFSQYLQK	C
3087	2	35	F	-1.61	3.575	0.572	0.572	gi|124028612|sp|P02770.2|ALBU_RAT	27	K	GLVLIAFSQYLQK	C
2826	2	35	F	-0.16	4.394	0.599	0.599	gi|124028612|sp|P02770.2|ALBU_RAT	27	K	GLVLIAFSQYLQK	C
3473	2	35	F	-1.93	3.307	0.612	0.612	gi|124028612|sp|P02770.2|ALBU_RAT	27	K	GLVLIAFSQYLQK	C
2838	2	35	F	-0.38	3.738	0.613	0.613	gi|124028612|sp|P02770.2|ALBU_RAT	27	K	GLVLIAFSQYLQK	C
3748	2	35	F	-2	3.147	0.613	0.613	gi|124028612|sp|P02770.2|ALBU_RAT	27	K	GLVLIAFSQYLQK	C
2959	2	35	F	-0.69	3.042	0.617	0.617	gi|124028612|sp|P02770.2|ALBU_RAT	27	K	GLVLIAFSQYLQK	C
4346	2	35	F	-4.86	3.041	0.617	0.617	gi|124028612|sp|P02770.2|ALBU_RAT	27	K	GLVLIAFSQYLQK	C
3205	2	35	F	-2.85	3.673	0.618	0.618	gi|124028612|sp|P02770.2|ALBU_RAT	27	K	GLVLIAFSQYLQK	C
3470	2	35	F	-1.81	3.285	0.619	0.619	gi|124028612|sp|P02770.2|ALBU_RAT	27	K	GLVLIAFSQYLQK	C
3337	2	35	F	-1.02	3.582	0.62	0.62	gi|124028612|sp|P02770.2|ALBU_RAT	27	K	GLVLIAFSQYLQK	C
3083	2	35	F	-1.16	2.979	0.624	0.624	gi|124028612|sp|P02770.2|ALBU_RAT	27	K	GLVLIAFSQYLQK	C
3612	2	35	F	-1.52	3.894	0.625	0.625	gi|124028612|sp|P02770.2|ALBU_RAT	27	K	GLVLIAFSQYLQK	C
3889	2	35	F	-1.78	4.012	0.626	0.626	gi|124028612|sp|P02770.2|ALBU_RAT	27	K	GLVLIAFSQYLQK	C
3751	2	35	F	-1.94	3.357	0.631	0.631	gi|124028612|sp|P02770.2|ALBU_RAT	27	K	GLVLIAFSQYLQK	C
4782	2	35	F	-4.96	3.444	0.633	0.633	gi|124028612|sp|P02770.2|ALBU_RAT	27	K	GLVLIAFSQYLQK	C
3334	2	35	F	-1.11	3.549	0.634	0.634	gi|124028612|sp|P02770.2|ALBU_RAT	27	K	GLVLIAFSQYLQK	C
3615	2	35	F	-2.16	3.518	0.64	0.64	gi|124028612|sp|P02770.2|ALBU_RAT	27	K	GLVLIAFSQYLQK	C
3208	2	35	F	-1.41	3.373	0.655	0.655	gi|124028612|sp|P02770.2|ALBU_RAT	27	K	GLVLIAFSQYLQK	C
3886	2	35	F	-4.35	3.281	0.657	0.657	gi|124028612|sp|P02770.2|ALBU_RAT	27	K	GLVLIAFSQYLQK	C

Scan	z	Sample ID	Band	PPM	XCorr	Delta correlation	Unique delta correlation	Reference	Redundancy		Peptides	
2341	3	35	F	4.06	4.239	0	0.814	gi|554561044|ref|XP_005874890.1|	0	R	HPDYSVSLLLR	L
2075	2	35	F	0.79	3.289	0.907	0.907	gi|731280814|ref|XP_010609182.1|	34	K	KQTALAELVK	H
4339	2	35	F	2.58	2.56	0	0.844	gi|432092344|gb|ELK24962.1|	15	K	LGEYGFQNALLVR^	Y
2680	2	35	F	-2.11	2.756	0	0.884	gi|432092344|gb|ELK24962.1|	15	K	LGEYGFQNALLVR^	Y
2429	3	35	F	2.93	5.085	0	0.888	gi|432092344|gb|ELK24962.1|	15	K	LGEYGFQNALLVR^	Y
2539	2	35	F	-0.04	3.347	0	0.9	gi|432092344|gb|ELK24962.1|	15	K	LGEYGFQNALLVR^	Y
2552	2	35	F	3.66	2.853	0	0.903	gi|432092344|gb|ELK24962.1|	15	K	LGEYGFQNALLVR^	Y
4785	2	35	F	1.41	3.219	0	0.914	gi|432092344|gb|ELK24962.1|	15	K	LGEYGFQNALLVR^	Y
2386	3	35	F	3.31	5.173	0.831	0.831	gi|5915682|sp|P07724.3|ALBU_MOUSE	12	R	LSQTFPNADFAEITK	L
2375	3	35	F	0.05	4.916	0.836	0.836	gi|5915682|sp|P07724.3|ALBU_MOUSE	12	R	LSQTFPNADFAEITK	L
2374	2	35	F	1.72	4.042	0.918	0.918	gi|5915682|sp|P07724.3|ALBU_MOUSE	12	R	LSQTFPNADFAEITK	L
4934	2	35	F	-4.6	3.535	0.931	0.931	gi|5915682|sp|P07724.3|ALBU_MOUSE	12	R	LSQTFPNADFAEITK	L
2121	2	35	F	2.94	3.206	0.306	0.306	gi|124028612|sp|P02770.2|ALBU_RAT	19	K	LVQEVTDFAK	T
2209	2	35	F	4.13	2.504	0.766	0.766	gi|731280814|ref|XP_010609182.1|	36	K	QTALAELVK	H
3284	3	35	F	-0.06	3.437	0	0.923	gi|554561044|ref|XP_005874890.1|	0	R	RHPDYSVSLLLR	L
3322	3	35	F	-0.43	3.055	0	0.936	gi|554561044|ref|XP_005874890.1|	0	R	RHPDYSVSLLLR	L
1292	2	35	F	-2.08	2.515	0.67	0.67	gi|5915682|sp|P07724.3|ALBU_MOUSE	13	K	TPVSEHVTK	C
1402	2	35	F	-1.38	2.673	0.691	0.691	gi|5915682|sp|P07724.3|ALBU_MOUSE	13	K	TPVSEHVTK	C
2377	3	38	F	4.27	5.021	0.407	0.407	gi|432092344|gb|ELK24962.1|	88	K	DVFLGTFLYEYSR	R
2709	2	38	F	3.99	3.485	0.557	0.557	gi|432092344|gb|ELK24962.1|	88	K	DVFLGTFLYEYSR	R
3923	2	38	F	0.02	2.975	0.893	0.893	gi|432092344|gb|ELK24962.1|	88	K	DVFLGTFLYEYSR	R
3439	2	38	F	0.12	3.399	0.897	0.897	gi|432092344|gb|ELK24962.1|	88	K	DVFLGTFLYEYSR	R
3570	2	38	F	0.27	3.472	0.897	0.897	gi|432092344|gb|ELK24962.1|	88	K	DVFLGTFLYEYSR	R
3449	2	38	F	0.11	3.693	0.901	0.901	gi|432092344|gb|ELK24962.1|	88	K	DVFLGTFLYEYSR	R
2584	2	38	F	-0.2	3.291	0.905	0.905	gi|432092344|gb|ELK24962.1|	88	K	DVFLGTFLYEYSR	R
3314	2	38	F	0.26	3.443	0.907	0.907	gi|432092344|gb|ELK24962.1|	88	K	DVFLGTFLYEYSR	R
3795	2	38	F	-0.09	3.444	0.91	0.91	gi|432092344|gb|ELK24962.1|	88	K	DVFLGTFLYEYSR	R
3798	2	38	F	-0.06	3.023	0.911	0.911	gi|432092344|gb|ELK24962.1|	88	K	DVFLGTFLYEYSR	R
3675	2	38	F	-0.06	3.495	0.913	0.913	gi|432092344|gb|ELK24962.1|	88	K	DVFLGTFLYEYSR	R
3257	3	38	F	-0.19	3.503	0.918	0.918	gi|432092344|gb|ELK24962.1|	88	K	DVFLGTFLYEYSR	R
3370	3	38	F	-0.26	3.369	0.918	0.918	gi|432092344|gb|ELK24962.1|	88	K	DVFLGTFLYEYSR	R
3920	2	38	F	0.1	3.236	0.918	0.918	gi|432092344|gb|ELK24962.1|	88	K	DVFLGTFLYEYSR	R
3322	2	38	F	0.3	3.365	0.92	0.92	gi|432092344|gb|ELK24962.1|	88	K	DVFLGTFLYEYSR	R
3558	2	38	F	0.22	3.614	0.92	0.92	gi|432092344|gb|ELK24962.1|	88	K	DVFLGTFLYEYSR	R
3016	3	38	F	-0.5	3.842	0.922	0.922	gi|432092344|gb|ELK24962.1|	88	K	DVFLGTFLYEYSR	R
2636	3	38	F	-0.11	3.549	0.925	0.925	gi|432092344|gb|ELK24962.1|	88	K	DVFLGTFLYEYSR	R
3253	3	38	F	-0.22	3.381	0.926	0.926	gi|432092344|gb|ELK24962.1|	88	K	DVFLGTFLYEYSR	R
3127	3	38	F	-0.59	3.403	0.929	0.929	gi|432092344|gb|ELK24962.1|	88	K	DVFLGTFLYEYSR	R

Scan	z	Sample ID	Band	PPM	XCorr	Delta correlation	Unique delta correlation	Reference	Redundancy		Peptides	
2645	3	38	F	-0.24	3.052	0.93	0.93	gi|432092344|gb|ELK24962.1|	88	K	DVFLGTFLYEYSR	R
3373	3	38	F	-0.28	3.393	0.936	0.936	gi|432092344|gb|ELK24962.1|	88	K	DVFLGTFLYEYSR	R
2766	3	38	F	-0.7	4.162	0.939	0.939	gi|432092344|gb|ELK24962.1|	88	K	DVFLGTFLYEYSR	R
2885	3	38	F	-0.42	3.246	0.948	0.948	gi|432092344|gb|ELK24962.1|	88	K	DVFLGTFLYEYSR	R
2373	3	38	F	-0.3	5.387	0.95	0.95	gi|432092344|gb|ELK24962.1|	88	K	DVFLGTFLYEYSR	R
3132	3	38	F	-0.64	3.17	0.958	0.958	gi|432092344|gb|ELK24962.1|	88	K	DVFLGTFLYEYSR	R
2976	3	38	F	-1.04	3.136	0.414	0.414	gi|124028612|sp|P02770.2|ALBU_RAT	27	K	GLVLIAFSQYLQK	C
2599	3	38	F	0.61	3.767	0.42	0.42	gi|124028612|sp|P02770.2|ALBU_RAT	27	K	GLVLIAFSQYLQK	C
3213	3	38	F	-0.79	3.299	0.423	0.423	gi|124028612|sp|P02770.2|ALBU_RAT	27	K	GLVLIAFSQYLQK	C
2724	3	38	F	-1.09	3.221	0.433	0.433	gi|124028612|sp|P02770.2|ALBU_RAT	27	K	GLVLIAFSQYLQK	C
2981	3	38	F	-1.03	3.229	0.436	0.436	gi|124028612|sp|P02770.2|ALBU_RAT	27	K	GLVLIAFSQYLQK	C
2860	3	38	F	1.74	3.733	0.439	0.439	gi|124028612|sp|P02770.2|ALBU_RAT	27	K	GLVLIAFSQYLQK	C
3218	3	38	F	-0.8	3.414	0.454	0.454	gi|124028612|sp|P02770.2|ALBU_RAT	27	K	GLVLIAFSQYLQK	C
3094	3	38	F	-0.94	3.338	0.46	0.46	gi|124028612|sp|P02770.2|ALBU_RAT	27	K	GLVLIAFSQYLQK	C
3092	3	38	F	-0.9	3.808	0.465	0.465	gi|124028612|sp|P02770.2|ALBU_RAT	27	K	GLVLIAFSQYLQK	C
2854	3	38	F	-0.69	3.697	0.474	0.474	gi|124028612|sp|P02770.2|ALBU_RAT	27	K	GLVLIAFSQYLQK	C
2742	3	38	F	3.44	3.777	0.48	0.48	gi|124028612|sp|P02770.2|ALBU_RAT	27	K	GLVLIAFSQYLQK	C
2301	3	38	F	0.03	6.189	0.49	0.49	gi|124028612|sp|P02770.2|ALBU_RAT	27	K	GLVLIAFSQYLQK	C
2451	3	38	F	1.58	5.472	0.499	0.499	gi|124028612|sp|P02770.2|ALBU_RAT	27	K	GLVLIAFSQYLQK	C
2442	3	38	F	-0.23	5.992	0.508	0.508	gi|124028612|sp|P02770.2|ALBU_RAT	27	K	GLVLIAFSQYLQK	C
2309	3	38	F	4.37	6.028	0.512	0.512	gi|124028612|sp|P02770.2|ALBU_RAT	27	K	GLVLIAFSQYLQK	C
2602	3	38	F	-1.09	5.219	0.534	0.534	gi|124028612|sp|P02770.2|ALBU_RAT	27	K	GLVLIAFSQYLQK	C
3294	2	38	F	-2.08	3.764	0.57	0.57	gi|124028612|sp|P02770.2|ALBU_RAT	27	K	GLVLIAFSQYLQK	C
3655	2	38	F	-3.16	3.033	0.572	0.572	gi|124028612|sp|P02770.2|ALBU_RAT	27	K	GLVLIAFSQYLQK	C
2821	2	38	F	-1.55	3.661	0.573	0.573	gi|124028612|sp|P02770.2|ALBU_RAT	27	K	GLVLIAFSQYLQK	C
2696	2	38	F	-1.53	2.998	0.578	0.578	gi|124028612|sp|P02770.2|ALBU_RAT	27	K	GLVLIAFSQYLQK	C
2934	2	38	F	-1.11	4.468	0.581	0.581	gi|124028612|sp|P02770.2|ALBU_RAT	27	K	GLVLIAFSQYLQK	C
3179	2	38	F	-2.03	4.813	0.582	0.582	gi|124028612|sp|P02770.2|ALBU_RAT	27	K	GLVLIAFSQYLQK	C
2939	2	38	F	-1.07	4.511	0.584	0.584	gi|124028612|sp|P02770.2|ALBU_RAT	27	K	GLVLIAFSQYLQK	C
3428	2	38	F	-1.97	3.491	0.585	0.585	gi|124028612|sp|P02770.2|ALBU_RAT	27	K	GLVLIAFSQYLQK	C
3299	2	38	F	-2.06	3.87	0.593	0.593	gi|124028612|sp|P02770.2|ALBU_RAT	27	K	GLVLIAFSQYLQK	C
2817	2	38	F	-1.55	4.262	0.596	0.596	gi|124028612|sp|P02770.2|ALBU_RAT	27	K	GLVLIAFSQYLQK	C
3174	2	38	F	-1.99	4.44	0.596	0.596	gi|124028612|sp|P02770.2|ALBU_RAT	27	K	GLVLIAFSQYLQK	C
3055	2	38	F	-1.95	4.455	0.6	0.6	gi|124028612|sp|P02770.2|ALBU_RAT	27	K	GLVLIAFSQYLQK	C
3059	2	38	F	-1.9	4.389	0.6	0.6	gi|124028612|sp|P02770.2|ALBU_RAT	27	K	GLVLIAFSQYLQK	C
3418	2	38	F	-1.96	4.333	0.606	0.606	gi|124028612|sp|P02770.2|ALBU_RAT	27	K	GLVLIAFSQYLQK	C
2428	2	38	F	-0.38	3.7	0.614	0.614	gi|124028612|sp|P02770.2|ALBU_RAT	27	K	GLVLIAFSQYLQK	C
2578	2	38	F	-0.93	2.627	0.62	0.62	gi|124028612|sp|P02770.2|ALBU_RAT	27	K	GLVLIAFSQYLQK	C

Scan	z	Sample ID	Band	PPM	XCorr	Delta correlation	Unique delta correlation	Reference	Redundancy		Peptides	
3911	2	38	F	-3.22	3.455	0.624	0.624	gi|124028612|sp|P02770.2|ALBU_RAT	27	K	GLVLIAFSQYLQK	C
2437	2	38	F	-0.69	3.629	0.629	0.629	gi|124028612|sp|P02770.2|ALBU_RAT	27	K	GLVLIAFSQYLQK	C
3538	2	38	F	-2.49	3.588	0.632	0.632	gi|124028612|sp|P02770.2|ALBU_RAT	27	K	GLVLIAFSQYLQK	C
3779	2	38	F	-3.22	3.084	0.632	0.632	gi|124028612|sp|P02770.2|ALBU_RAT	27	K	GLVLIAFSQYLQK	C
2703	2	38	F	-1.82	2.958	0.637	0.637	gi|124028612|sp|P02770.2|ALBU_RAT	27	K	GLVLIAFSQYLQK	C
3913	2	38	F	-3.1	3.429	0.642	0.642	gi|124028612|sp|P02770.2|ALBU_RAT	27	K	GLVLIAFSQYLQK	C
2567	2	38	F	-1.2	2.54	0.658	0.658	gi|124028612|sp|P02770.2|ALBU_RAT	27	K	GLVLIAFSQYLQK	C
1921	3	38	F	-0.05	4.096	0	0.809	gi|554561044|ref|XP_005874890.1|	0	R	HPDYSVSLLLR	L
2115	2	38	F	0.16	3.445	0	0.876	gi|432092344|gb|ELK24962.1|	15	K	LGEYGFQNALLVR^	Y
2033	3	38	F	-0.65	5.072	0	0.879	gi|432092344|gb|ELK24962.1|	15	K	LGEYGFQNALLVR^	Y
2130	2	38	F	4.14	3.042	0	0.914	gi|432092344|gb|ELK24962.1|	15	K	LGEYGFQNALLVR^	Y
2413	3	38	F	0.07	3.533	0	0.829	gi|3121749|sp|O35090.1|ALBU_MERUN	3	R	LPC#VEDYLSAILNR	V
2416	2	38	F	-0.39	4.918	0	0.914	gi|3121749|sp|O35090.1|ALBU_MERUN	3	R	LPC#VEDYLSAILNR	V
2411	2	38	F	-0.33	4.928	0	0.943	gi|3121749|sp|O35090.1|ALBU_MERUN	3	R	LPC#VEDYLSAILNR	V
1965	3	38	F	-0.27	3.491	0.812	0.812	gi|5915682|sp|P07724.3|ALBU_MOUSE	12	R	LSQTFPNADFAEITK	L
1983	2	38	F	1.57	4.036	0.93	0.93	gi|5915682|sp|P07724.3|ALBU_MOUSE	12	R	LSQTFPNADFAEITK	L
3593	3	38	F	-0.14	3.355	0	0.9	gi|554561044|ref|XP_005874890.1|	0	R	RHPDYSVSLLLR	L
2715	3	38	F	2.97	3.747	0	0.912	gi|554561044|ref|XP_005874890.1|	0	R	RHPDYSVSLLLR	L
2835	3	38	F	0.01	3.797	0	0.917	gi|554561044|ref|XP_005874890.1|	0	R	RHPDYSVSLLLR	L
2956	3	38	F	-0.45	3.789	0	0.919	gi|554561044|ref|XP_005874890.1|	0	R	RHPDYSVSLLLR	L
2841	3	38	F	0.33	4.184	0	0.92	gi|554561044|ref|XP_005874890.1|	0	R	RHPDYSVSLLLR	L
3202	3	38	F	-0.19	4.043	0	0.92	gi|554561044|ref|XP_005874890.1|	0	R	RHPDYSVSLLLR	L
2685	3	38	F	3.59	4.236	0	0.921	gi|554561044|ref|XP_005874890.1|	0	R	RHPDYSVSLLLR	L
2952	3	38	F	-0.56	3.677	0	0.936	gi|554561044|ref|XP_005874890.1|	0	R	RHPDYSVSLLLR	L
3823	3	38	F	-0.67	3.712	0	0.944	gi|554561044|ref|XP_005874890.1|	0	R	RHPDYSVSLLLR	L
3323	3	38	F	-0.51	3.252	0	0.945	gi|554561044|ref|XP_005874890.1|	0	R	RHPDYSVSLLLR	L
3826	3	38	F	-0.63	3.641	0	0.948	gi|554561044|ref|XP_005874890.1|	0	R	RHPDYSVSLLLR	L
3070	3	38	F	-0.68	4.062	0	0.949	gi|554561044|ref|XP_005874890.1|	0	R	RHPDYSVSLLLR	L
3074	3	38	F	-0.69	4.298	0	0.952	gi|554561044|ref|XP_005874890.1|	0	R	RHPDYSVSLLLR	L
3193	3	38	F	-0.28	4.295	0	0.954	gi|554561044|ref|XP_005874890.1|	0	R	RHPDYSVSLLLR	L
3440	3	38	F	-0.32	3.187	0	0.983	gi|554561044|ref|XP_005874890.1|	0	R	RHPDYSVSLLLR	L
942	2	38	F	-0.09	2.547	0.782	0.782	gi|5915682|sp|P07724.3|ALBU_MOUSE	13	K	TPVSEHVTK	C
3443	3	40	F	3.42	3.925	0.447	0.447	gi|432092344|gb|ELK24962.1|	88	K	DVFLGTFLYEYSR	R
3305	3	40	F	3.49	3.936	0.449	0.449	gi|432092344|gb|ELK24962.1|	88	K	DVFLGTFLYEYSR	R
3314	3	40	F	3.13	4.444	0.473	0.473	gi|432092344|gb|ELK24962.1|	88	K	DVFLGTFLYEYSR	R
3169	3	40	F	4.12	3.682	0.482	0.482	gi|432092344|gb|ELK24962.1|	88	K	DVFLGTFLYEYSR	R
3172	3	40	F	4.23	3.571	0.496	0.496	gi|432092344|gb|ELK24962.1|	88	K	DVFLGTFLYEYSR	R
4289	2	40	F	3.85	3.395	0.511	0.511	gi|432092344|gb|ELK24962.1|	88	K	DVFLGTFLYEYSR	R

Scan	z	Sample ID	Band	PPM	XCorr	Delta correlation	Unique delta correlation	Reference	Redundancy		Peptides	
3396	2	40	F	3.75	3.835	0.526	0.526	gi|432092344|gb|ELK24962.1|	88	K	DVFLGTFLYEYSR	R
3527	2	40	F	4.24	2.872	0.533	0.533	gi|432092344|gb|ELK24962.1|	88	K	DVFLGTFLYEYSR	R
3265	2	40	F	4.18	3.925	0.537	0.537	gi|432092344|gb|ELK24962.1|	88	K	DVFLGTFLYEYSR	R
3267	2	40	F	4.19	3.844	0.538	0.538	gi|432092344|gb|ELK24962.1|	88	K	DVFLGTFLYEYSR	R
3394	2	40	F	3.75	3.788	0.555	0.555	gi|432092344|gb|ELK24962.1|	88	K	DVFLGTFLYEYSR	R
3706	3	40	F	-0.37	3.076	0.91	0.91	gi|432092344|gb|ELK24962.1|	88	K	DVFLGTFLYEYSR	R
4035	2	40	F	0.36	2.931	0.911	0.911	gi|432092344|gb|ELK24962.1|	88	K	DVFLGTFLYEYSR	R
3906	2	40	F	0.25	3.6	0.912	0.912	gi|432092344|gb|ELK24962.1|	88	K	DVFLGTFLYEYSR	R
3658	2	40	F	0.16	3.9	0.914	0.914	gi|432092344|gb|ELK24962.1|	88	K	DVFLGTFLYEYSR	R
4286	2	40	F	0.55	3.691	0.914	0.914	gi|432092344|gb|ELK24962.1|	88	K	DVFLGTFLYEYSR	R
3580	3	40	F	-0.4	4.377	0.918	0.918	gi|432092344|gb|ELK24962.1|	88	K	DVFLGTFLYEYSR	R
4216	3	40	F	-0.46	3.46	0.918	0.918	gi|432092344|gb|ELK24962.1|	88	K	DVFLGTFLYEYSR	R
3787	2	40	F	0.24	4.028	0.922	0.922	gi|432092344|gb|ELK24962.1|	88	K	DVFLGTFLYEYSR	R
3654	2	40	F	0.14	4.851	0.923	0.923	gi|432092344|gb|ELK24962.1|	88	K	DVFLGTFLYEYSR	R
3909	2	40	F	0.34	3.771	0.923	0.923	gi|432092344|gb|ELK24962.1|	88	K	DVFLGTFLYEYSR	R
3784	2	40	F	0.22	4.562	0.924	0.924	gi|432092344|gb|ELK24962.1|	88	K	DVFLGTFLYEYSR	R
3959	3	40	F	-0.13	3.932	0.94	0.94	gi|432092344|gb|ELK24962.1|	88	K	DVFLGTFLYEYSR	R
4156	2	40	F	0.37	4.027	0.943	0.943	gi|432092344|gb|ELK24962.1|	88	K	DVFLGTFLYEYSR	R
3575	3	40	F	-0.19	4.302	0.944	0.944	gi|432092344|gb|ELK24962.1|	88	K	DVFLGTFLYEYSR	R
3447	3	40	F	2.35	3.434	0.947	0.947	gi|432092344|gb|ELK24962.1|	88	K	DVFLGTFLYEYSR	R
4214	3	40	F	-0.47	3.551	0.961	0.961	gi|432092344|gb|ELK24962.1|	88	K	DVFLGTFLYEYSR	R
3715	3	40	F	-0.54	3.199	0.963	0.963	gi|432092344|gb|ELK24962.1|	88	K	DVFLGTFLYEYSR	R
4160	2	40	F	0.29	2.846	0.996	0.996	gi|432092344|gb|ELK24962.1|	88	K	DVFLGTFLYEYSR	R
3150	3	40	F	3.02	3.955	0.423	0.423	gi|124028612|sp|P02770.2|ALBU_RAT	27	K	GLVLIAFSQYLQK	C
3357	3	40	F	3.64	4.125	0.435	0.435	gi|124028612|sp|P02770.2|ALBU_RAT	27	K	GLVLIAFSQYLQK	C
3846	3	40	F	-0.42	3.872	0.446	0.446	gi|124028612|sp|P02770.2|ALBU_RAT	27	K	GLVLIAFSQYLQK	C
4111	3	40	F	-0.57	3.32	0.458	0.458	gi|124028612|sp|P02770.2|ALBU_RAT	27	K	GLVLIAFSQYLQK	C
3976	3	40	F	-0.45	3.042	0.469	0.469	gi|124028612|sp|P02770.2|ALBU_RAT	27	K	GLVLIAFSQYLQK	C
3549	3	40	F	-1.38	3.924	0.475	0.475	gi|124028612|sp|P02770.2|ALBU_RAT	27	K	GLVLIAFSQYLQK	C
3351	3	40	F	3.49	4.572	0.48	0.48	gi|124028612|sp|P02770.2|ALBU_RAT	27	K	GLVLIAFSQYLQK	C
3693	3	40	F	-0.17	4.125	0.486	0.486	gi|124028612|sp|P02770.2|ALBU_RAT	27	K	GLVLIAFSQYLQK	C
3973	3	40	F	-0.45	3.711	0.488	0.488	gi|124028612|sp|P02770.2|ALBU_RAT	27	K	GLVLIAFSQYLQK	C
3716	3	40	F	-0.09	4.604	0.491	0.491	gi|124028612|sp|P02770.2|ALBU_RAT	27	K	GLVLIAFSQYLQK	C
3493	3	40	F	-1.05	4.171	0.494	0.494	gi|124028612|sp|P02770.2|ALBU_RAT	27	K	GLVLIAFSQYLQK	C
3854	3	40	F	-0.45	3.256	0.497	0.497	gi|124028612|sp|P02770.2|ALBU_RAT	27	K	GLVLIAFSQYLQK	C
4240	3	40	F	0.06	4.221	0.504	0.504	gi|124028612|sp|P02770.2|ALBU_RAT	27	K	GLVLIAFSQYLQK	C
4246	3	40	F	2.58	4.724	0.509	0.509	gi|124028612|sp|P02770.2|ALBU_RAT	27	K	GLVLIAFSQYLQK	C
3719	2	40	F	-1.65	3.73	0.543	0.543	gi|124028612|sp|P02770.2|ALBU_RAT	27	K	GLVLIAFSQYLQK	C

Scan	z	Sample ID	Band	PPM	XCorr	Delta correlation	Unique delta correlation	Reference	Redundancy		Peptides	
3559	3	40	F	-0.67	4.482	0.544	0.544	gi|124028612|sp|P02770.2|ALBU_RAT	27	K	GLVLIAFSQYLQK	C
3331	2	40	F	2.63	2.82	0.545	0.545	gi|124028612|sp|P02770.2|ALBU_RAT	27	K	GLVLIAFSQYLQK	C
3723	2	40	F	-1.62	4.408	0.561	0.561	gi|124028612|sp|P02770.2|ALBU_RAT	27	K	GLVLIAFSQYLQK	C
3966	2	40	F	-1.84	3.607	0.573	0.573	gi|124028612|sp|P02770.2|ALBU_RAT	27	K	GLVLIAFSQYLQK	C
3202	2	40	F	3.31	3.122	0.578	0.578	gi|124028612|sp|P02770.2|ALBU_RAT	27	K	GLVLIAFSQYLQK	C
3590	2	40	F	-0.18	3.994	0.584	0.584	gi|124028612|sp|P02770.2|ALBU_RAT	27	K	GLVLIAFSQYLQK	C
4098	2	40	F	-2.32	3.59	0.588	0.588	gi|124028612|sp|P02770.2|ALBU_RAT	27	K	GLVLIAFSQYLQK	C
4219	2	40	F	-1.13	3.84	0.592	0.592	gi|124028612|sp|P02770.2|ALBU_RAT	27	K	GLVLIAFSQYLQK	C
3593	2	40	F	-0.27	3.582	0.595	0.595	gi|124028612|sp|P02770.2|ALBU_RAT	27	K	GLVLIAFSQYLQK	C
3333	2	40	F	2.92	4.051	0.599	0.599	gi|124028612|sp|P02770.2|ALBU_RAT	27	K	GLVLIAFSQYLQK	C
4221	2	40	F	-1.26	3.793	0.6	0.6	gi|124028612|sp|P02770.2|ALBU_RAT	27	K	GLVLIAFSQYLQK	C
3970	2	40	F	-1.74	3.56	0.615	0.615	gi|124028612|sp|P02770.2|ALBU_RAT	27	K	GLVLIAFSQYLQK	C
3200	2	40	F	3.44	3.272	0.616	0.616	gi|124028612|sp|P02770.2|ALBU_RAT	27	K	GLVLIAFSQYLQK	C
3462	2	40	F	2.38	3.382	0.627	0.627	gi|124028612|sp|P02770.2|ALBU_RAT	27	K	GLVLIAFSQYLQK	C
4096	2	40	F	-2.24	3.324	0.627	0.627	gi|124028612|sp|P02770.2|ALBU_RAT	27	K	GLVLIAFSQYLQK	C
3460	2	40	F	2.95	3.382	0.69	0.69	gi|124028612|sp|P02770.2|ALBU_RAT	27	K	GLVLIAFSQYLQK	C
2414	2	40	F	4.76	3.141	0	0.788	gi|432092344|gb|ELK24962.1|	15	K	LGEYGFQNALLVR^	Y
2410	2	40	F	4.99	4.128	0	0.838	gi|432092344|gb|ELK24962.1|	15	K	LGEYGFQNALLVR^	Y
4342	2	40	F	2.03	2.68	0	0.848	gi|432092344|gb|ELK24962.1|	15	K	LGEYGFQNALLVR^	Y
2256	2	40	F	3.05	3.402	0	0.896	gi|432092344|gb|ELK24962.1|	15	K	LGEYGFQNALLVR^	Y
4348	2	40	F	2.01	3.309	0	0.903	gi|432092344|gb|ELK24962.1|	15	K	LGEYGFQNALLVR^	Y
2246	2	40	F	1.75	3.56	0	0.907	gi|432092344|gb|ELK24962.1|	15	K	LGEYGFQNALLVR^	Y
4101	2	40	F	-0.12	2.577	0.903	0.903	gi|5915682|sp|P07724.3|ALBU_MOUSE	12	R	LSQTFPNADFAEITK	L
3967	2	40	F	0.23	2.549	0.904	0.904	gi|5915682|sp|P07724.3|ALBU_MOUSE	12	R	LSQTFPNADFAEITK	L
2072	2	40	F	2.05	3.838	0.921	0.921	gi|5915682|sp|P07724.3|ALBU_MOUSE	12	R	LSQTFPNADFAEITK	L
3299	2	40	F	4.18	3.26	0.925	0.925	gi|5915682|sp|P07724.3|ALBU_MOUSE	12	R	LSQTFPNADFAEITK	L
3295	2	40	F	4.22	4.383	0.94	0.94	gi|5915682|sp|P07724.3|ALBU_MOUSE	12	R	LSQTFPNADFAEITK	L
1917	2	40	F	4.25	3.021	0	0.875	gi|554561044|ref|XP_005874890.1|	0	R	RHPDYSVSLLLR	L
4146	3	40	F	-0.15	3.506	0	0.902	gi|554561044|ref|XP_005874890.1|	0	R	RHPDYSVSLLLR	L
4013	3	40	F	-0.1	3.53	0	0.91	gi|554561044|ref|XP_005874890.1|	0	R	RHPDYSVSLLLR	L
4019	3	40	F	-0.08	3.687	0	0.91	gi|554561044|ref|XP_005874890.1|	0	R	RHPDYSVSLLLR	L
4151	3	40	F	-0.21	3.651	0	0.912	gi|554561044|ref|XP_005874890.1|	0	R	RHPDYSVSLLLR	L
3573	3	40	F	-0.13	3.859	0	0.919	gi|554561044|ref|XP_005874890.1|	0	R	RHPDYSVSLLLR	L
4302	3	40	F	-0.13	4.125	0	0.921	gi|554561044|ref|XP_005874890.1|	0	R	RHPDYSVSLLLR	L
2208	3	40	F	-0.7	4.023	0	0.922	gi|554561044|ref|XP_005874890.1|	0	R	RHPDYSVSLLLR	L
4296	3	40	F	-0.05	4.449	0	0.929	gi|554561044|ref|XP_005874890.1|	0	R	RHPDYSVSLLLR	L
3897	3	40	F	-0.29	3.652	0	0.935	gi|554561044|ref|XP_005874890.1|	0	R	RHPDYSVSLLLR	L
3874	3	40	F	-0.09	3.179	0	0.984	gi|554561044|ref|XP_005874890.1|	0	R	RHPDYSVSLLLR	L

Scan	z	Sample ID	Band	PPM	XCorr	Delta correlation	Unique delta correlation	Reference	Redundancy		Peptides	
3775	3	40	F	-1.03	4.114	0	0.988	gi|554561044|ref|XP_005874890.1|	0	R	RHPDYSVSLLLR	L
2207	3	41	F	0.25	4.398	0.645	0.645	gi|3121749|sp|O35090.1|ALBU_MERUN	26	K	APQVSTPTLVEAAR	S
2216	3	41	F	0.25	4.054	0.684	0.684	gi|3121749|sp|O35090.1|ALBU_MERUN	26	K	APQVSTPTLVEAAR	S
2196	2	41	F	1.93	3.687	0.89	0.89	gi|3121749|sp|O35090.1|ALBU_MERUN	26	K	APQVSTPTLVEAAR	S
4371	2	41	F	0.21	2.749	0.946	0.946	gi|3121749|sp|O35090.1|ALBU_MERUN	26	K	APQVSTPTLVEAAR	S
3156	3	41	F	2.94	4.69	0.447	0.447	gi|432092344|gb|ELK24962.1|	88	K	DVFLGTFLYEYSR	R
3290	3	41	F	3.11	3.57	0.45	0.45	gi|432092344|gb|ELK24962.1|	88	K	DVFLGTFLYEYSR	R
3153	3	41	F	3.59	3.578	0.488	0.488	gi|432092344|gb|ELK24962.1|	88	K	DVFLGTFLYEYSR	R
3419	3	41	F	3.25	4.75	0.492	0.492	gi|432092344|gb|ELK24962.1|	88	K	DVFLGTFLYEYSR	R
3376	2	41	F	2.7	3.648	0.528	0.528	gi|432092344|gb|ELK24962.1|	88	K	DVFLGTFLYEYSR	R
3381	2	41	F	3.73	4.446	0.571	0.571	gi|432092344|gb|ELK24962.1|	88	K	DVFLGTFLYEYSR	R
4168	3	41	F	0.01	3.353	0.893	0.893	gi|432092344|gb|ELK24962.1|	88	K	DVFLGTFLYEYSR	R
4565	2	41	F	0.19	4.047	0.9	0.9	gi|432092344|gb|ELK24962.1|	88	K	DVFLGTFLYEYSR	R
3799	3	41	F	-0.47	3.007	0.918	0.918	gi|432092344|gb|ELK24962.1|	88	K	DVFLGTFLYEYSR	R
4439	2	41	F	0.48	2.942	0.918	0.918	gi|432092344|gb|ELK24962.1|	88	K	DVFLGTFLYEYSR	R
4562	2	41	F	0.21	3.825	0.919	0.919	gi|432092344|gb|ELK24962.1|	88	K	DVFLGTFLYEYSR	R
4468	3	41	F	-0.39	3.277	0.92	0.92	gi|432092344|gb|ELK24962.1|	88	K	DVFLGTFLYEYSR	R
4600	3	41	F	-0.33	3.09	0.92	0.92	gi|432092344|gb|ELK24962.1|	88	K	DVFLGTFLYEYSR	R
4473	3	41	F	-0.4	3.754	0.922	0.922	gi|432092344|gb|ELK24962.1|	88	K	DVFLGTFLYEYSR	R
4279	3	41	F	-0.23	3.399	0.934	0.934	gi|432092344|gb|ELK24962.1|	88	K	DVFLGTFLYEYSR	R
3802	3	41	F	-0.37	3.012	0.937	0.937	gi|432092344|gb|ELK24962.1|	88	K	DVFLGTFLYEYSR	R
3550	3	41	F	-0.15	4.5	0.939	0.939	gi|432092344|gb|ELK24962.1|	88	K	DVFLGTFLYEYSR	R
4045	3	41	F	-0.33	4.177	0.94	0.94	gi|432092344|gb|ELK24962.1|	88	K	DVFLGTFLYEYSR	R
3675	3	41	F	-0.53	4.962	0.941	0.941	gi|432092344|gb|ELK24962.1|	88	K	DVFLGTFLYEYSR	R
3417	3	41	F	-0.61	4.529	0.943	0.943	gi|432092344|gb|ELK24962.1|	88	K	DVFLGTFLYEYSR	R
3544	3	41	F	-0.25	5.156	0.945	0.945	gi|432092344|gb|ELK24962.1|	88	K	DVFLGTFLYEYSR	R
4043	3	41	F	-0.4	4.274	0.945	0.945	gi|432092344|gb|ELK24962.1|	88	K	DVFLGTFLYEYSR	R
3677	3	41	F	-0.56	3.528	0.946	0.946	gi|432092344|gb|ELK24962.1|	88	K	DVFLGTFLYEYSR	R
3926	3	41	F	-0.52	3.506	0.947	0.947	gi|432092344|gb|ELK24962.1|	88	K	DVFLGTFLYEYSR	R
4436	2	41	F	0.47	3.411	0.97	0.97	gi|432092344|gb|ELK24962.1|	88	K	DVFLGTFLYEYSR	R
3920	3	41	F	-0.55	3.217	0.972	0.972	gi|432092344|gb|ELK24962.1|	88	K	DVFLGTFLYEYSR	R
3829	2	41	F	0.07	4.231	0.983	0.983	gi|432092344|gb|ELK24962.1|	88	K	DVFLGTFLYEYSR	R
2142	2	41	F	4.11	4.203	0.933	0.933	gi|5915682|sp|P07724.3|ALBU_MOUSE	16	K	ENPTTFM*GHYLHEVAR	R
2153	2	41	F	-0.09	4.316	0.947	0.947	gi|5915682|sp|P07724.3|ALBU_MOUSE	16	K	ENPTTFM*GHYLHEVAR	R
2422	3	41	F	1.28	3.138	0.856	0.856	gi|5915682|sp|P07724.3|ALBU_MOUSE	16	K	ENPTTFMGHYLHEVAR	R
2419	2	41	F	1.07	4.004	0.902	0.902	gi|5915682|sp|P07724.3|ALBU_MOUSE	16	K	ENPTTFMGHYLHEVAR	R
2423	2	41	F	0.89	4.178	0.933	0.933	gi|5915682|sp|P07724.3|ALBU_MOUSE	16	K	ENPTTFMGHYLHEVAR	R
4148	3	41	F	1.11	3.147	0.391	0.391	gi|124028612|sp|P02770.2|ALBU_RAT	27	K	GLVLIAFSQYLQK	C

Scan	z	Sample ID	Band	PPM	XCorr	Delta correlation	Unique delta correlation	Reference	Redundancy		Peptides	
3111	3	41	F	3.58	3.229	0.414	0.414	gi|124028612|sp|P02770.2|ALBU_RAT	27	K	GLVLIAFSQYLQK	C
4527	3	41	F	-1.2	3.989	0.436	0.436	gi|124028612|sp|P02770.2|ALBU_RAT	27	K	GLVLIAFSQYLQK	C
3104	3	41	F	2.96	3.685	0.451	0.451	gi|124028612|sp|P02770.2|ALBU_RAT	27	K	GLVLIAFSQYLQK	C
4137	3	41	F	-0.39	3.453	0.465	0.465	gi|124028612|sp|P02770.2|ALBU_RAT	27	K	GLVLIAFSQYLQK	C
4026	3	41	F	-0.91	4.091	0.47	0.47	gi|124028612|sp|P02770.2|ALBU_RAT	27	K	GLVLIAFSQYLQK	C
3652	3	41	F	-0.48	3.974	0.472	0.472	gi|124028612|sp|P02770.2|ALBU_RAT	27	K	GLVLIAFSQYLQK	C
3654	3	41	F	-0.37	3.704	0.502	0.502	gi|124028612|sp|P02770.2|ALBU_RAT	27	K	GLVLIAFSQYLQK	C
4530	3	41	F	-1.03	3.627	0.502	0.502	gi|124028612|sp|P02770.2|ALBU_RAT	27	K	GLVLIAFSQYLQK	C
3240	3	41	F	3.18	5.368	0.508	0.508	gi|124028612|sp|P02770.2|ALBU_RAT	27	K	GLVLIAFSQYLQK	C
3526	3	41	F	-0.73	5.388	0.508	0.508	gi|124028612|sp|P02770.2|ALBU_RAT	27	K	GLVLIAFSQYLQK	C
4022	3	41	F	-0.8	3.989	0.512	0.512	gi|124028612|sp|P02770.2|ALBU_RAT	27	K	GLVLIAFSQYLQK	C
3775	3	41	F	-0.65	5.285	0.516	0.516	gi|124028612|sp|P02770.2|ALBU_RAT	27	K	GLVLIAFSQYLQK	C
3902	3	41	F	-0.44	5.171	0.531	0.531	gi|124028612|sp|P02770.2|ALBU_RAT	27	K	GLVLIAFSQYLQK	C
3389	3	41	F	3.37	5.151	0.533	0.533	gi|124028612|sp|P02770.2|ALBU_RAT	27	K	GLVLIAFSQYLQK	C
3778	3	41	F	-0.68	5.297	0.533	0.533	gi|124028612|sp|P02770.2|ALBU_RAT	27	K	GLVLIAFSQYLQK	C
3057	2	41	F	3.66	3.09	0.534	0.534	gi|124028612|sp|P02770.2|ALBU_RAT	27	K	GLVLIAFSQYLQK	C
3248	3	41	F	3.05	4.694	0.542	0.542	gi|124028612|sp|P02770.2|ALBU_RAT	27	K	GLVLIAFSQYLQK	C
3400	3	41	F	2.49	5.09	0.551	0.551	gi|124028612|sp|P02770.2|ALBU_RAT	27	K	GLVLIAFSQYLQK	C
3440	2	41	F	2.56	2.888	0.553	0.553	gi|124028612|sp|P02770.2|ALBU_RAT	27	K	GLVLIAFSQYLQK	C
3529	3	41	F	-0.55	5.561	0.555	0.555	gi|124028612|sp|P02770.2|ALBU_RAT	27	K	GLVLIAFSQYLQK	C
3055	2	41	F	3.85	3.628	0.556	0.556	gi|124028612|sp|P02770.2|ALBU_RAT	27	K	GLVLIAFSQYLQK	C
3907	3	41	F	-0.38	4.124	0.563	0.563	gi|124028612|sp|P02770.2|ALBU_RAT	27	K	GLVLIAFSQYLQK	C
3938	2	41	F	-1.2	4.356	0.565	0.565	gi|124028612|sp|P02770.2|ALBU_RAT	27	K	GLVLIAFSQYLQK	C
3817	2	41	F	-1.01	3.707	0.571	0.571	gi|124028612|sp|P02770.2|ALBU_RAT	27	K	GLVLIAFSQYLQK	C
3695	2	41	F	-1.11	4.701	0.578	0.578	gi|124028612|sp|P02770.2|ALBU_RAT	27	K	GLVLIAFSQYLQK	C
4057	2	41	F	-1.07	4.099	0.58	0.58	gi|124028612|sp|P02770.2|ALBU_RAT	27	K	GLVLIAFSQYLQK	C
4053	2	41	F	-1.03	4.104	0.583	0.583	gi|124028612|sp|P02770.2|ALBU_RAT	27	K	GLVLIAFSQYLQK	C
3815	2	41	F	-1.05	3.894	0.585	0.585	gi|124028612|sp|P02770.2|ALBU_RAT	27	K	GLVLIAFSQYLQK	C
3185	2	41	F	2.41	3.898	0.59	0.59	gi|124028612|sp|P02770.2|ALBU_RAT	27	K	GLVLIAFSQYLQK	C
4177	2	41	F	0.05	4.309	0.59	0.59	gi|124028612|sp|P02770.2|ALBU_RAT	27	K	GLVLIAFSQYLQK	C
3934	2	41	F	-1.26	4.319	0.594	0.594	gi|124028612|sp|P02770.2|ALBU_RAT	27	K	GLVLIAFSQYLQK	C
3692	2	41	F	-1.02	3.556	0.602	0.602	gi|124028612|sp|P02770.2|ALBU_RAT	27	K	GLVLIAFSQYLQK	C
4546	2	41	F	-1.25	4.565	0.602	0.602	gi|124028612|sp|P02770.2|ALBU_RAT	27	K	GLVLIAFSQYLQK	C
4166	2	41	F	0.13	4.17	0.605	0.605	gi|124028612|sp|P02770.2|ALBU_RAT	27	K	GLVLIAFSQYLQK	C
4289	2	41	F	-1.53	3.8	0.606	0.606	gi|124028612|sp|P02770.2|ALBU_RAT	27	K	GLVLIAFSQYLQK	C
4417	2	41	F	-1.64	3.276	0.607	0.607	gi|124028612|sp|P02770.2|ALBU_RAT	27	K	GLVLIAFSQYLQK	C
3183	2	41	F	2.39	3.619	0.62	0.62	gi|124028612|sp|P02770.2|ALBU_RAT	27	K	GLVLIAFSQYLQK	C
4550	2	41	F	-1.26	4.588	0.622	0.622	gi|124028612|sp|P02770.2|ALBU_RAT	27	K	GLVLIAFSQYLQK	C

Scan	z	Sample ID	Band	PPM	XCorr	Delta correlation	Unique delta correlation	Reference	Redundancy		Peptides	
4299	2	41	F	-1.56	4.376	0.63	0.63	gi|124028612|sp|P02770.2|ALBU_RAT	27	K	GLVLIAFSQYLQK	C
3437	2	41	F	2.78	3.441	0.633	0.633	gi|124028612|sp|P02770.2|ALBU_RAT	27	K	GLVLIAFSQYLQK	C
3316	2	41	F	3.37	2.631	0.642	0.642	gi|124028612|sp|P02770.2|ALBU_RAT	27	K	GLVLIAFSQYLQK	C
4423	2	41	F	-1.74	3.378	0.657	0.657	gi|124028612|sp|P02770.2|ALBU_RAT	27	K	GLVLIAFSQYLQK	C
2414	3	41	F	3.41	3.362	0	0.768	gi|554561044|ref|XP_005874890.1|	0	R	HPDYSVSLLLR	L
2405	3	41	F	-0.3	4.143	0	0.795	gi|554561044|ref|XP_005874890.1|	0	R	HPDYSVSLLLR	L
2118	3	41	F	3.21	3.88	0.832	0.832	gi|731280814|ref|XP_010609182.1|	34	K	KQTALAELVK	H
2109	3	41	F	3.14	4.244	0.846	0.846	gi|731280814|ref|XP_010609182.1|	34	K	KQTALAELVK	H
2107	2	41	F	3.13	3.342	0.892	0.892	gi|731280814|ref|XP_010609182.1|	34	K	KQTALAELVK	H
2117	2	41	F	3.13	3.482	0.895	0.895	gi|731280814|ref|XP_010609182.1|	34	K	KQTALAELVK	H
1234	2	41	F	1.26	2.562	0.178	0.178	gi|3121749|sp|O35090.1|ALBU_MERUN	38	K	KYEATLEK	C
2533	3	41	F	2.32	3.209	0	0.78	gi|432092344|gb|ELK24962.1|	15	K	LGEYGFQNALLVR^	Y
2528	3	41	F	2.35	4.202	0	0.859	gi|432092344|gb|ELK24962.1|	15	K	LGEYGFQNALLVR^	Y
2634	2	41	F	3.27	3.553	0	0.916	gi|432092344|gb|ELK24962.1|	15	K	LGEYGFQNALLVR^	Y
2643	2	41	F	3.4	3.561	0	0.919	gi|432092344|gb|ELK24962.1|	15	K	LGEYGFQNALLVR^	Y
4231	2	41	F	0.17	2.646	0.908	0.908	gi|5915682|sp|P07724.3|ALBU_MOUSE	12	R	LSQTFPNADFAEITK	L
3581	2	41	F	3.44	3.058	0.922	0.922	gi|5915682|sp|P07724.3|ALBU_MOUSE	12	R	LSQTFPNADFAEITK	L
2466	2	41	F	3.76	5.099	0.932	0.932	gi|5915682|sp|P07724.3|ALBU_MOUSE	12	R	LSQTFPNADFAEITK	L
2205	2	41	F	1.57	3.009	0.335	0.335	gi|124028612|sp|P02770.2|ALBU_RAT	19	K	LVQEVTDFAK	T
4196	3	41	F	-0.15	3.182	0	0.897	gi|554561044|ref|XP_005874890.1|	0	R	RHPDYSVSLLLR	L
4563	3	41	F	-0.26	3.414	0	0.901	gi|554561044|ref|XP_005874890.1|	0	R	RHPDYSVSLLLR	L
2559	3	41	F	4.14	4.385	0	0.913	gi|554561044|ref|XP_005874890.1|	0	R	RHPDYSVSLLLR	L
2550	3	41	F	2.78	4.058	0	0.918	gi|554561044|ref|XP_005874890.1|	0	R	RHPDYSVSLLLR	L
2382	3	41	F	0.73	4.19	0	0.919	gi|554561044|ref|XP_005874890.1|	0	R	RHPDYSVSLLLR	L
2396	3	41	F	0.44	3.093	0	0.919	gi|554561044|ref|XP_005874890.1|	0	R	RHPDYSVSLLLR	L
3866	3	41	F	-0.32	4.117	0	0.92	gi|554561044|ref|XP_005874890.1|	0	R	RHPDYSVSLLLR	L
4003	3	41	F	-0.14	4.222	0	0.922	gi|554561044|ref|XP_005874890.1|	0	R	RHPDYSVSLLLR	L
2359	2	41	F	-0.71	2.758	0	0.931	gi|554561044|ref|XP_005874890.1|	0	R	RHPDYSVSLLLR	L
3871	3	41	F	-0.3	3.788	0	0.934	gi|554561044|ref|XP_005874890.1|	0	R	RHPDYSVSLLLR	L
3991	3	41	F	0.09	3.609	0	0.936	gi|554561044|ref|XP_005874890.1|	0	R	RHPDYSVSLLLR	L
3730	3	41	F	0.27	3.799	0	0.938	gi|554561044|ref|XP_005874890.1|	0	R	RHPDYSVSLLLR	L
4559	3	41	F	-0.11	4.037	0	0.941	gi|554561044|ref|XP_005874890.1|	0	R	RHPDYSVSLLLR	L
4181	3	41	F	-0.2	3.566	0	0.946	gi|554561044|ref|XP_005874890.1|	0	R	RHPDYSVSLLLR	L
3708	3	41	F	-0.47	4.131	0	0.95	gi|554561044|ref|XP_005874890.1|	0	R	RHPDYSVSLLLR	L
3569	3	41	F	-0.77	3.874	0	0.987	gi|554561044|ref|XP_005874890.1|	0	R	RHPDYSVSLLLR	L
2529	2	41	F	3.92	3.026	0.928	0.928	gi|124028612|sp|P02770.2|ALBU_RAT	18	R	RPCFSALTVDETYVPK	E
1393	2	41	F	-1.57	2.879	0.587	0.587	gi|5915682|sp|P07724.3|ALBU_MOUSE	13	K	TPVSEHVTK	C
1797	2	41	F	-0.45	2.686	0.64	0.64	gi|5915682|sp|P07724.3|ALBU_MOUSE	13	K	TPVSEHVTK	C

Scan	z	Sample ID	Band	PPM	XCorr	Delta correlation	Unique delta correlation	Reference	Redundancy		Peptides	
1272	2	41	F	-1.59	3.025	0.653	0.653	gi|5915682|sp|P07724.3|ALBU_MOUSE	13	K	TPVSEHVTK	C
1668	2	41	F	-0.76	2.53	0.676	0.676	gi|5915682|sp|P07724.3|ALBU_MOUSE	13	K	TPVSEHVTK	C
1278	2	41	F	-1.3	2.511	0.678	0.678	gi|5915682|sp|P07724.3|ALBU_MOUSE	13	K	TPVSEHVTK	C
1162	2	41	F	0.99	2.691	0.681	0.681	gi|5915682|sp|P07724.3|ALBU_MOUSE	13	K	TPVSEHVTK	C
1919	2	41	F	-0.53	2.603	0.681	0.681	gi|5915682|sp|P07724.3|ALBU_MOUSE	13	K	TPVSEHVTK	C
1157	2	41	F	0.98	2.962	0.725	0.725	gi|5915682|sp|P07724.3|ALBU_MOUSE	13	K	TPVSEHVTK	C
2018	2	41	F	-0.42	3.236	0.303	0.303	gi|124028612|sp|P02770.2|ALBU_RAT	18	K	YMC#ENQATISSK	L
2012	2	41	F	2.71	3.526	0.374	0.374	gi|124028612|sp|P02770.2|ALBU_RAT	18	K	YMC#ENQATISSK	L
1996	3	41	F	0.99	3.021	0.868	0.868	gi|5915682|sp|P07724.3|ALBU_MOUSE	15	R	YNDLGEQHFK	G
1997	2	41	F	0.23	3.728	0.877	0.877	gi|5915682|sp|P07724.3|ALBU_MOUSE	15	R	YNDLGEQHFK	G
2008	2	41	F	3.45	3.686	0.88	0.88	gi|5915682|sp|P07724.3|ALBU_MOUSE	15	R	YNDLGEQHFK	G
2279	3	42	F	0.4	4.533	0.648	0.648	gi|3121749|sp|O35090.1|ALBU_MERUN	26	K	APQVSTPTLVEAAR	S
2264	2	42	F	2.31	3.536	0.86	0.86	gi|3121749|sp|O35090.1|ALBU_MERUN	26	K	APQVSTPTLVEAAR	S
2946	3	42	F	2.9	5.064	0.477	0.477	gi|432092344|gb|ELK24962.1|	88	K	DVFLGTFLYEYSR	R
2933	2	42	F	2.96	3.764	0.513	0.513	gi|432092344|gb|ELK24962.1|	88	K	DVFLGTFLYEYSR	R
2929	2	42	F	2.7	3.826	0.557	0.557	gi|432092344|gb|ELK24962.1|	88	K	DVFLGTFLYEYSR	R
4434	2	42	F	0	2.836	0.887	0.887	gi|432092344|gb|ELK24962.1|	88	K	DVFLGTFLYEYSR	R
3468	3	42	F	-0.07	3.752	0.906	0.906	gi|432092344|gb|ELK24962.1|	88	K	DVFLGTFLYEYSR	R
4559	2	42	F	0.1	3.865	0.907	0.907	gi|432092344|gb|ELK24962.1|	88	K	DVFLGTFLYEYSR	R
4563	2	42	F	0.09	4.175	0.907	0.907	gi|432092344|gb|ELK24962.1|	88	K	DVFLGTFLYEYSR	R
4301	2	42	F	0.33	3.675	0.908	0.908	gi|432092344|gb|ELK24962.1|	88	K	DVFLGTFLYEYSR	R
4298	2	42	F	0.42	3.838	0.911	0.911	gi|432092344|gb|ELK24962.1|	88	K	DVFLGTFLYEYSR	R
4187	2	42	F	0.39	3.383	0.913	0.913	gi|432092344|gb|ELK24962.1|	88	K	DVFLGTFLYEYSR	R
3592	3	42	F	-0.68	3.328	0.919	0.919	gi|432092344|gb|ELK24962.1|	88	K	DVFLGTFLYEYSR	R
4429	2	42	F	0.07	3.2	0.919	0.919	gi|432092344|gb|ELK24962.1|	88	K	DVFLGTFLYEYSR	R
3465	3	42	F	-0.15	4.765	0.922	0.922	gi|432092344|gb|ELK24962.1|	88	K	DVFLGTFLYEYSR	R
4508	3	42	F	-0.05	3.406	0.923	0.923	gi|432092344|gb|ELK24962.1|	88	K	DVFLGTFLYEYSR	R
4502	3	42	F	-0.14	3.056	0.924	0.924	gi|432092344|gb|ELK24962.1|	88	K	DVFLGTFLYEYSR	R
4221	3	42	F	-0.36	3.719	0.926	0.926	gi|432092344|gb|ELK24962.1|	88	K	DVFLGTFLYEYSR	R
3957	3	42	F	-0.43	3.81	0.927	0.927	gi|432092344|gb|ELK24962.1|	88	K	DVFLGTFLYEYSR	R
3589	3	42	F	-0.7	4.99	0.931	0.931	gi|432092344|gb|ELK24962.1|	88	K	DVFLGTFLYEYSR	R
3840	3	42	F	-0.72	3.651	0.932	0.932	gi|432092344|gb|ELK24962.1|	88	K	DVFLGTFLYEYSR	R
3835	3	42	F	-0.69	3.816	0.934	0.934	gi|432092344|gb|ELK24962.1|	88	K	DVFLGTFLYEYSR	R
3333	3	42	F	-0.61	4.406	0.938	0.938	gi|432092344|gb|ELK24962.1|	88	K	DVFLGTFLYEYSR	R
4093	3	42	F	-0.3	4.107	0.94	0.94	gi|432092344|gb|ELK24962.1|	88	K	DVFLGTFLYEYSR	R
3074	3	42	F	-0.18	5.11	0.945	0.945	gi|432092344|gb|ELK24962.1|	88	K	DVFLGTFLYEYSR	R
3205	3	42	F	-0.46	4.778	0.945	0.945	gi|432092344|gb|ELK24962.1|	88	K	DVFLGTFLYEYSR	R
3348	3	42	F	-0.66	3.318	0.945	0.945	gi|432092344|gb|ELK24962.1|	88	K	DVFLGTFLYEYSR	R

Scan	z	Sample ID	Band	PPM	XCorr	Delta correlation	Unique delta correlation	Reference	Redundancy		Peptides	
2941	3	42	F	2.42	4.274	0.946	0.946	gi|432092344|gb|ELK24962.1|	88	K	DVFLGTFLYEYSR	R
3714	3	42	F	-0.53	4.601	0.948	0.948	gi|432092344|gb|ELK24962.1|	88	K	DVFLGTFLYEYSR	R
3965	3	42	F	-0.45	4.412	0.948	0.948	gi|432092344|gb|ELK24962.1|	88	K	DVFLGTFLYEYSR	R
3072	3	42	F	-0.21	4.158	0.949	0.949	gi|432092344|gb|ELK24962.1|	88	K	DVFLGTFLYEYSR	R
3210	3	42	F	-0.58	3.615	0.951	0.951	gi|432092344|gb|ELK24962.1|	88	K	DVFLGTFLYEYSR	R
3711	3	42	F	-0.5	3.782	0.956	0.956	gi|432092344|gb|ELK24962.1|	88	K	DVFLGTFLYEYSR	R
4211	3	42	F	-0.37	3.282	0.957	0.957	gi|432092344|gb|ELK24962.1|	88	K	DVFLGTFLYEYSR	R
2216	2	42	F	0.87	4.253	0.95	0.95	gi|5915682|sp|P07724.3|ALBU_MOUSE	16	K	ENPTTFM*GHYLHEVAR	R
2229	2	42	F	-0.34	4.169	0.982	0.982	gi|5915682|sp|P07724.3|ALBU_MOUSE	16	K	ENPTTFM*GHYLHEVAR	R
2505	2	42	F	0.67	3.663	0.918	0.918	gi|5915682|sp|P07724.3|ALBU_MOUSE	16	K	ENPTTFMGHYLHEVAR	R
2515	2	42	F	4.34	4.62	0.943	0.943	gi|5915682|sp|P07724.3|ALBU_MOUSE	16	K	ENPTTFMGHYLHEVAR	R
4076	3	42	F	-0.25	3.115	0.407	0.407	gi|124028612|sp|P02770.2|ALBU_RAT	27	K	GLVLIAFSQYLQK	C
4489	3	42	F	-0.47	3.366	0.416	0.416	gi|124028612|sp|P02770.2|ALBU_RAT	27	K	GLVLIAFSQYLQK	C
3836	3	42	F	-0.88	3.193	0.417	0.417	gi|124028612|sp|P02770.2|ALBU_RAT	27	K	GLVLIAFSQYLQK	C
3328	3	42	F	-0.86	3.409	0.422	0.422	gi|124028612|sp|P02770.2|ALBU_RAT	27	K	GLVLIAFSQYLQK	C
4492	3	42	F	-0.44	3.276	0.422	0.422	gi|124028612|sp|P02770.2|ALBU_RAT	27	K	GLVLIAFSQYLQK	C
4072	3	42	F	-0.32	3.62	0.436	0.436	gi|124028612|sp|P02770.2|ALBU_RAT	27	K	GLVLIAFSQYLQK	C
3192	3	42	F	-0.46	3.855	0.459	0.459	gi|124028612|sp|P02770.2|ALBU_RAT	27	K	GLVLIAFSQYLQK	C
3706	3	42	F	-0.76	3.62	0.459	0.459	gi|124028612|sp|P02770.2|ALBU_RAT	27	K	GLVLIAFSQYLQK	C
3463	3	42	F	-0.73	3.554	0.467	0.467	gi|124028612|sp|P02770.2|ALBU_RAT	27	K	GLVLIAFSQYLQK	C
3206	3	42	F	-0.49	4.076	0.478	0.478	gi|124028612|sp|P02770.2|ALBU_RAT	27	K	GLVLIAFSQYLQK	C
3958	3	42	F	-0.86	3.588	0.48	0.48	gi|124028612|sp|P02770.2|ALBU_RAT	27	K	GLVLIAFSQYLQK	C
3458	3	42	F	-0.66	4.234	0.482	0.482	gi|124028612|sp|P02770.2|ALBU_RAT	27	K	GLVLIAFSQYLQK	C
3953	3	42	F	-0.79	3.981	0.487	0.487	gi|124028612|sp|P02770.2|ALBU_RAT	27	K	GLVLIAFSQYLQK	C
3586	3	42	F	-0.63	3.905	0.49	0.49	gi|124028612|sp|P02770.2|ALBU_RAT	27	K	GLVLIAFSQYLQK	C
3828	3	42	F	-0.85	3.873	0.496	0.496	gi|124028612|sp|P02770.2|ALBU_RAT	27	K	GLVLIAFSQYLQK	C
3584	3	42	F	-0.64	4.711	0.499	0.499	gi|124028612|sp|P02770.2|ALBU_RAT	27	K	GLVLIAFSQYLQK	C
3059	3	42	F	-0.48	5.515	0.507	0.507	gi|124028612|sp|P02770.2|ALBU_RAT	27	K	GLVLIAFSQYLQK	C
3709	3	42	F	-0.72	4.468	0.507	0.507	gi|124028612|sp|P02770.2|ALBU_RAT	27	K	GLVLIAFSQYLQK	C
3342	3	42	F	-0.95	4.563	0.523	0.523	gi|124028612|sp|P02770.2|ALBU_RAT	27	K	GLVLIAFSQYLQK	C
2921	3	42	F	1.66	6.262	0.538	0.538	gi|124028612|sp|P02770.2|ALBU_RAT	27	K	GLVLIAFSQYLQK	C
2916	3	42	F	2.99	6.025	0.543	0.543	gi|124028612|sp|P02770.2|ALBU_RAT	27	K	GLVLIAFSQYLQK	C
3744	2	42	F	-1.54	4.186	0.558	0.558	gi|124028612|sp|P02770.2|ALBU_RAT	27	K	GLVLIAFSQYLQK	C
3248	2	42	F	-0.81	2.979	0.575	0.575	gi|124028612|sp|P02770.2|ALBU_RAT	27	K	GLVLIAFSQYLQK	C
3374	2	42	F	-1.11	2.624	0.575	0.575	gi|124028612|sp|P02770.2|ALBU_RAT	27	K	GLVLIAFSQYLQK	C
3054	3	42	F	-0.47	5.697	0.576	0.576	gi|124028612|sp|P02770.2|ALBU_RAT	27	K	GLVLIAFSQYLQK	C
3862	2	42	F	-1.62	4.748	0.576	0.576	gi|124028612|sp|P02770.2|ALBU_RAT	27	K	GLVLIAFSQYLQK	C
3116	2	42	F	-0.91	3.998	0.578	0.578	gi|124028612|sp|P02770.2|ALBU_RAT	27	K	GLVLIAFSQYLQK	C

Scan	z	Sample ID	Band	PPM	XCorr	Delta correlation	Unique delta correlation	Reference	Redundancy		Peptides	
3981	2	42	F	-1.33	4.552	0.581	0.581	gi|124028612|sp|P02770.2|ALBU_RAT	27	K	GLVLIAFSQYLQK	C
3987	2	42	F	-1.36	4.549	0.581	0.581	gi|124028612|sp|P02770.2|ALBU_RAT	27	K	GLVLIAFSQYLQK	C
3623	2	42	F	-1.6	4.41	0.586	0.586	gi|124028612|sp|P02770.2|ALBU_RAT	27	K	GLVLIAFSQYLQK	C
4491	2	42	F	-1.63	3.403	0.586	0.586	gi|124028612|sp|P02770.2|ALBU_RAT	27	K	GLVLIAFSQYLQK	C
3746	2	42	F	-1.62	4.469	0.59	0.59	gi|124028612|sp|P02770.2|ALBU_RAT	27	K	GLVLIAFSQYLQK	C
3499	2	42	F	-1.26	3.914	0.595	0.595	gi|124028612|sp|P02770.2|ALBU_RAT	27	K	GLVLIAFSQYLQK	C
4115	2	42	F	-1.43	3.446	0.596	0.596	gi|124028612|sp|P02770.2|ALBU_RAT	27	K	GLVLIAFSQYLQK	C
3252	2	42	F	-0.75	3.377	0.6	0.6	gi|124028612|sp|P02770.2|ALBU_RAT	27	K	GLVLIAFSQYLQK	C
3626	2	42	F	-1.54	4.372	0.603	0.603	gi|124028612|sp|P02770.2|ALBU_RAT	27	K	GLVLIAFSQYLQK	C
4231	2	42	F	-1.8	3.59	0.604	0.604	gi|124028612|sp|P02770.2|ALBU_RAT	27	K	GLVLIAFSQYLQK	C
3122	2	42	F	-0.95	3.466	0.607	0.607	gi|124028612|sp|P02770.2|ALBU_RAT	27	K	GLVLIAFSQYLQK	C
4356	2	42	F	-1.57	4.07	0.612	0.612	gi|124028612|sp|P02770.2|ALBU_RAT	27	K	GLVLIAFSQYLQK	C
3864	2	42	F	-1.6	4.694	0.616	0.616	gi|124028612|sp|P02770.2|ALBU_RAT	27	K	GLVLIAFSQYLQK	C
4220	2	42	F	-1.66	3.858	0.631	0.631	gi|124028612|sp|P02770.2|ALBU_RAT	27	K	GLVLIAFSQYLQK	C
4362	2	42	F	-1.51	3.519	0.631	0.631	gi|124028612|sp|P02770.2|ALBU_RAT	27	K	GLVLIAFSQYLQK	C
2985	2	42	F	3.13	2.71	0.643	0.643	gi|124028612|sp|P02770.2|ALBU_RAT	27	K	GLVLIAFSQYLQK	C
4105	2	42	F	-1.45	3.258	0.647	0.647	gi|124028612|sp|P02770.2|ALBU_RAT	27	K	GLVLIAFSQYLQK	C
3502	2	42	F	-1.3	3.72	0.648	0.648	gi|124028612|sp|P02770.2|ALBU_RAT	27	K	GLVLIAFSQYLQK	C
4496	2	42	F	-1.55	3.687	0.66	0.66	gi|124028612|sp|P02770.2|ALBU_RAT	27	K	GLVLIAFSQYLQK	C
2483	3	42	F	0.87	3.623	0	0.781	gi|554561044|ref|XP_005874890.1|	0	R	HPDYSVSLLLR	L
2473	3	42	F	0.97	3.936	0	0.814	gi|554561044|ref|XP_005874890.1|	0	R	HPDYSVSLLLR	L
2192	3	42	F	2.12	4.223	0.819	0.819	gi|731280814|ref|XP_010609182.1|	34	K	KQTALAELVK	H
2203	3	42	F	2.12	4.394	0.836	0.836	gi|731280814|ref|XP_010609182.1|	34	K	KQTALAELVK	H
2202	2	42	F	1.59	3.512	0.871	0.871	gi|731280814|ref|XP_010609182.1|	34	K	KQTALAELVK	H
2191	2	42	F	1.6	3.572	0.907	0.907	gi|731280814|ref|XP_010609182.1|	34	K	KQTALAELVK	H
2856	2	42	F	0.2	2.637	0	0.871	gi|432092344|gb|ELK24962.1|	15	K	LGEYGFQNALLVR^	Y
2592	3	42	F	-0.65	5.591	0	0.879	gi|432092344|gb|ELK24962.1|	15	K	LGEYGFQNALLVR^	Y
4622	2	42	F	2.22	2.759	0	0.887	gi|432092344|gb|ELK24962.1|	15	K	LGEYGFQNALLVR^	Y
2581	3	42	F	-0.64	5.509	0	0.892	gi|432092344|gb|ELK24962.1|	15	K	LGEYGFQNALLVR^	Y
2691	2	42	F	0.7	3.476	0	0.907	gi|432092344|gb|ELK24962.1|	15	K	LGEYGFQNALLVR^	Y
2701	2	42	F	3.48	3.742	0	0.914	gi|432092344|gb|ELK24962.1|	15	K	LGEYGFQNALLVR^	Y
2846	2	42	F	0.18	3.721	0	0.927	gi|432092344|gb|ELK24962.1|	15	K	LGEYGFQNALLVR^	Y
3033	3	42	F	1.65	3.532	0	0.86	gi|3121749|sp|O35090.1|ALBU_MERUN	3	R	LPC#VEDYLSAILNR	V
3028	2	42	F	0.08	4.392	0	0.898	gi|3121749|sp|O35090.1|ALBU_MERUN	3	R	LPC#VEDYLSAILNR	V
3031	2	42	F	1.41	5.066	0	0.93	gi|3121749|sp|O35090.1|ALBU_MERUN	3	R	LPC#VEDYLSAILNR	V
2526	2	42	F	1.96	4.269	0.92	0.92	gi|5915682|sp|P07724.3|ALBU_MOUSE	12	R	LSQTFPNADFAEITK	L
2268	2	42	F	1.5	2.969	0.322	0.322	gi|124028612|sp|P02770.2|ALBU_RAT	19	K	LVQEVTDFAK	T
2634	3	42	F	-0.34	3.011	0	0.864	gi|554561044|ref|XP_005874890.1|	0	R	RHPDYSVSLLLR	L

Scan	z	Sample ID	Band	PPM	XCorr	Delta correlation	Unique delta correlation	Reference	Redundancy		Peptides	
3672	3	42	F	1.28	4.011	0	0.912	gi|554561044|ref|XP_005874890.1|	0	R	RHPDYSVSLLLR	L
4048	3	42	F	-0.15	4.467	0	0.923	gi|554561044|ref|XP_005874890.1|	0	R	RHPDYSVSLLLR	L
2466	3	42	F	0.13	3.561	0	0.929	gi|554561044|ref|XP_005874890.1|	0	R	RHPDYSVSLLLR	L
2362	2	42	F	2.71	3.101	0	0.936	gi|554561044|ref|XP_005874890.1|	0	R	RHPDYSVSLLLR	L
3797	3	42	F	3.65	3.724	0	0.936	gi|554561044|ref|XP_005874890.1|	0	R	RHPDYSVSLLLR	L
4039	3	42	F	-0.21	3.888	0	0.939	gi|554561044|ref|XP_005874890.1|	0	R	RHPDYSVSLLLR	L
3669	3	42	F	-0.43	4.157	0	0.95	gi|554561044|ref|XP_005874890.1|	0	R	RHPDYSVSLLLR	L
3793	3	42	F	-0.39	3.977	0	0.962	gi|554561044|ref|XP_005874890.1|	0	R	RHPDYSVSLLLR	L
4556	3	42	F	-0.63	3.739	0	0.976	gi|554561044|ref|XP_005874890.1|	0	R	RHPDYSVSLLLR	L
2351	2	42	F	0.16	3.034	0	0.984	gi|554561044|ref|XP_005874890.1|	0	R	RHPDYSVSLLLR	L
4564	3	42	F	-0.46	3.934	0	0.986	gi|554561044|ref|XP_005874890.1|	0	R	RHPDYSVSLLLR	L
3916	3	42	F	-0.38	3.807	0	0.989	gi|554561044|ref|XP_005874890.1|	0	R	RHPDYSVSLLLR	L
3920	3	42	F	-0.41	3.802	0	0.993	gi|554561044|ref|XP_005874890.1|	0	R	RHPDYSVSLLLR	L
2672	3	42	F	-0.37	3.12	0.889	0.889	gi|5915682|sp|P07724.3|ALBU_MOUSE	19	K	SLHTLFGDKLCAIPNLR	E
1296	2	42	F	-1.1	3.11	0.644	0.644	gi|5915682|sp|P07724.3|ALBU_MOUSE	13	K	TPVSEHVTK	C
1554	2	42	F	-0.59	2.558	0.656	0.656	gi|5915682|sp|P07724.3|ALBU_MOUSE	13	K	TPVSEHVTK	C
1420	2	42	F	-1.22	2.878	0.665	0.665	gi|5915682|sp|P07724.3|ALBU_MOUSE	13	K	TPVSEHVTK	C
1179	2	42	F	-0.1	2.799	0.674	0.674	gi|5915682|sp|P07724.3|ALBU_MOUSE	13	K	TPVSEHVTK	C
1548	2	42	F	-0.57	2.636	0.68	0.68	gi|5915682|sp|P07724.3|ALBU_MOUSE	13	K	TPVSEHVTK	C
1175	2	42	F	-0.15	2.884	0.695	0.695	gi|5915682|sp|P07724.3|ALBU_MOUSE	13	K	TPVSEHVTK	C
1301	2	42	F	-1.07	2.995	0.695	0.695	gi|5915682|sp|P07724.3|ALBU_MOUSE	13	K	TPVSEHVTK	C
1415	2	42	F	-1.3	2.672	0.701	0.701	gi|5915682|sp|P07724.3|ALBU_MOUSE	13	K	TPVSEHVTK	C
2073	2	42	F	1.36	3.449	0.343	0.343	gi|124028612|sp|P02770.2|ALBU_RAT	18	K	YMC#ENQATISSK	L
2064	2	42	F	1.36	3.668	0.355	0.355	gi|124028612|sp|P02770.2|ALBU_RAT	18	K	YMC#ENQATISSK	L
2087	2	42	F	1.03	2.747	0.24	0.24	gi|124028612|sp|P02770.2|ALBU_RAT	18	K	YMCENQATISSK	L
2049	2	42	F	3.49	3.703	0.875	0.875	gi|5915682|sp|P07724.3|ALBU_MOUSE	15	R	YNDLGEQHFK	G
2330	2	43	F	-0.24	2.963	0.851	0.851	gi|3121749|sp|O35090.1|ALBU_MERUN	26	K	APQVSTPTLVEAAR	S
3056	2	43	F	0.19	3.541	0.911	0.911	gi|432092344|gb|ELK24962.1|	88	K	DVFLGTFLYEYSR	R
3051	2	43	F	0.13	3.603	0.922	0.922	gi|432092344|gb|ELK24962.1|	88	K	DVFLGTFLYEYSR	R
2574	2	43	F	2.15	3.689	0.883	0.883	gi|5915682|sp|P07724.3|ALBU_MOUSE	16	K	ENPTTFMGHYLHEVAR	R
2569	2	43	F	0.18	4.141	0.906	0.906	gi|5915682|sp|P07724.3|ALBU_MOUSE	16	K	ENPTTFMGHYLHEVAR	R
3043	3	43	F	-1.12	6.296	0.522	0.522	gi|124028612|sp|P02770.2|ALBU_RAT	27	K	GLVLIAFSQYLQK	C
3040	3	43	F	-1.17	6.147	0.523	0.523	gi|124028612|sp|P02770.2|ALBU_RAT	27	K	GLVLIAFSQYLQK	C
3035	2	43	F	-0.09	4.774	0.573	0.573	gi|124028612|sp|P02770.2|ALBU_RAT	27	K	GLVLIAFSQYLQK	C
3029	2	43	F	-0.11	2.709	0.689	0.689	gi|124028612|sp|P02770.2|ALBU_RAT	27	K	GLVLIAFSQYLQK	C
3186	2	43	F	2.49	2.694	0.726	0.726	gi|124028612|sp|P02770.2|ALBU_RAT	27	K	GLVLIAFSQYLQK	C
2211	2	43	F	-0.15	2.571	0.944	0.944	gi|731280814|ref|XP_010609182.1|	34	K	KQTALAELVK	H
2800	2	43	F	0.07	3.043	0	0.886	gi|432092344|gb|ELK24962.1|	15	K	LGEYGFQNALLVR^	Y

Scan	z	Sample ID	Band	PPM	XCorr	Delta correlation	Unique delta correlation	Reference	Redundancy		Peptides	
2649	2	43	F	0.05	4.165	0	0.922	gi|432092344|gb|ELK24962.1|	15	K	LGEYGFQNALLVR^	Y
2832	2	43	F	1.12	3.203	0	0.923	gi|432092344|gb|ELK24962.1|	15	K	LGEYGFQNALLVR^	Y
2605	2	43	F	0.54	4.135	0.924	0.924	gi|5915682|sp|P07724.3|ALBU_MOUSE	12	R	LSQTFPNADFAEITK	L
2432	2	43	F	-0.52	2.815	0	0.99	gi|554561044|ref|XP_005874890.1|	0	R	RHPDYSVSLLLR	L
2080	2	43	F	1.16	3.013	0.87	0.87	gi|5915682|sp|P07724.3|ALBU_MOUSE	15	R	YNDLGEQHFK	G
2527	3	44	F	-1.19	3.341	0.649	0.649	gi|3121749|sp|O35090.1|ALBU_MERUN	26	K	APQVSTPTLVEAAR	S
2503	2	44	F	-0.47	3.5	0.846	0.846	gi|3121749|sp|O35090.1|ALBU_MERUN	26	K	APQVSTPTLVEAAR	S
2514	2	44	F	-0.47	3.116	0.846	0.846	gi|3121749|sp|O35090.1|ALBU_MERUN	26	K	APQVSTPTLVEAAR	S
3156	2	44	F	0.29	3.398	0.907	0.907	gi|432092344|gb|ELK24962.1|	88	K	DVFLGTFLYEYSR	R
2758	2	44	F	-0.05	3.589	0.914	0.914	gi|5915682|sp|P07724.3|ALBU_MOUSE	16	K	ENPTTFMGHYLHEVAR	R
2772	2	44	F	-0.05	3.183	0.956	0.956	gi|5915682|sp|P07724.3|ALBU_MOUSE	16	K	ENPTTFMGHYLHEVAR	R
3157	3	44	F	-0.72	5.494	0.475	0.475	gi|124028612|sp|P02770.2|ALBU_RAT	27	K	GLVLIAFSQYLQK	C
3149	3	44	F	4.69	6.087	0.505	0.505	gi|124028612|sp|P02770.2|ALBU_RAT	27	K	GLVLIAFSQYLQK	C
3140	2	44	F	0.43	3.121	0.681	0.681	gi|124028612|sp|P02770.2|ALBU_RAT	27	K	GLVLIAFSQYLQK	C
2423	2	44	F	0.35	2.644	0.892	0.892	gi|731280814|ref|XP_010609182.1|	34	K	KQTALAELVK	H
2812	2	44	F	0.7	4.192	0	0.897	gi|432092344|gb|ELK24962.1|	15	K	LGEYGFQNALLVR^	Y
2768	2	44	F	0.88	2.973	0.917	0.917	gi|5915682|sp|P07724.3|ALBU_MOUSE	12	R	LSQTFPNADFAEITK	L
2778	2	44	F	0.88	4.027	0.942	0.942	gi|5915682|sp|P07724.3|ALBU_MOUSE	12	R	LSQTFPNADFAEITK	L
2635	3	44	F	0.63	5.133	0	0.931	gi|554561044|ref|XP_005874890.1|	0	R	RHPDYSVSLLLR	L
2640	2	44	F	0.11	2.62	0	0.989	gi|554561044|ref|XP_005874890.1|	0	R	RHPDYSVSLLLR	L
2290	2	44	F	-0.73	2.854	0.864	0.864	gi|5915682|sp|P07724.3|ALBU_MOUSE	15	R	YNDLGEQHFK	G
3981	3	45	F	3.17	4.031	0.412	0.412	gi|432092344|gb|ELK24962.1|	88	K	DVFLGTFLYEYSR	R
3986	3	45	F	3.23	4.777	0.413	0.413	gi|432092344|gb|ELK24962.1|	88	K	DVFLGTFLYEYSR	R
4130	3	45	F	4.22	4.524	0.485	0.485	gi|432092344|gb|ELK24962.1|	88	K	DVFLGTFLYEYSR	R
4124	3	45	F	4.49	4.389	0.512	0.512	gi|432092344|gb|ELK24962.1|	88	K	DVFLGTFLYEYSR	R
3969	2	45	F	4.6	3.464	0.559	0.559	gi|432092344|gb|ELK24962.1|	88	K	DVFLGTFLYEYSR	R
4224	2	45	F	3.11	3.643	0.561	0.561	gi|432092344|gb|ELK24962.1|	88	K	DVFLGTFLYEYSR	R
5391	2	45	F	0.11	3.532	0.896	0.896	gi|432092344|gb|ELK24962.1|	88	K	DVFLGTFLYEYSR	R
4773	3	45	F	-0.23	3.418	0.901	0.901	gi|432092344|gb|ELK24962.1|	88	K	DVFLGTFLYEYSR	R
4775	3	45	F	0.4	3.435	0.901	0.901	gi|432092344|gb|ELK24962.1|	88	K	DVFLGTFLYEYSR	R
5255	2	45	F	0.49	3.845	0.902	0.902	gi|432092344|gb|ELK24962.1|	88	K	DVFLGTFLYEYSR	R
5260	2	45	F	0.35	4.076	0.904	0.904	gi|432092344|gb|ELK24962.1|	88	K	DVFLGTFLYEYSR	R
5389	2	45	F	0.07	3.819	0.905	0.905	gi|432092344|gb|ELK24962.1|	88	K	DVFLGTFLYEYSR	R
4358	2	45	F	0.25	3.734	0.906	0.906	gi|432092344|gb|ELK24962.1|	88	K	DVFLGTFLYEYSR	R
5138	2	45	F	0.24	2.753	0.906	0.906	gi|432092344|gb|ELK24962.1|	88	K	DVFLGTFLYEYSR	R
4879	2	45	F	-0.26	3.65	0.913	0.913	gi|432092344|gb|ELK24962.1|	88	K	DVFLGTFLYEYSR	R
4881	2	45	F	-0.29	3.552	0.913	0.913	gi|432092344|gb|ELK24962.1|	88	K	DVFLGTFLYEYSR	R
4747	2	45	F	0.29	3.599	0.914	0.914	gi|432092344|gb|ELK24962.1|	88	K	DVFLGTFLYEYSR	R

Scan	z	Sample ID	Band	PPM	XCorr	Delta correlation	Unique delta correlation	Reference	Redundancy		Peptides	
4490	2	45	F	0.47	4.377	0.916	0.916	gi|432092344|gb|ELK24962.1|	88	K	DVFLGTFLYEYSR	R
4488	2	45	F	0.49	4.024	0.917	0.917	gi|432092344|gb|ELK24962.1|	88	K	DVFLGTFLYEYSR	R
4749	2	45	F	0.27	3.722	0.918	0.918	gi|432092344|gb|ELK24962.1|	88	K	DVFLGTFLYEYSR	R
4915	3	45	F	-0.96	3.288	0.918	0.918	gi|432092344|gb|ELK24962.1|	88	K	DVFLGTFLYEYSR	R
5128	2	45	F	0.24	3.399	0.92	0.92	gi|432092344|gb|ELK24962.1|	88	K	DVFLGTFLYEYSR	R
4614	2	45	F	0.18	3.743	0.921	0.921	gi|432092344|gb|ELK24962.1|	88	K	DVFLGTFLYEYSR	R
5042	3	45	F	-0.6	4.473	0.923	0.923	gi|432092344|gb|ELK24962.1|	88	K	DVFLGTFLYEYSR	R
4230	2	45	F	0.45	4.105	0.925	0.925	gi|432092344|gb|ELK24962.1|	88	K	DVFLGTFLYEYSR	R
4616	2	45	F	0.17	4.066	0.927	0.927	gi|432092344|gb|ELK24962.1|	88	K	DVFLGTFLYEYSR	R
4365	2	45	F	0.2	4.807	0.928	0.928	gi|432092344|gb|ELK24962.1|	88	K	DVFLGTFLYEYSR	R
4572	3	45	F	-0.39	3.94	0.935	0.935	gi|432092344|gb|ELK24962.1|	88	K	DVFLGTFLYEYSR	R
4606	3	45	F	-0.17	3.878	0.935	0.935	gi|432092344|gb|ELK24962.1|	88	K	DVFLGTFLYEYSR	R
4485	3	45	F	-1.02	3.484	0.937	0.937	gi|432092344|gb|ELK24962.1|	88	K	DVFLGTFLYEYSR	R
4910	3	45	F	-0.91	4.217	0.939	0.939	gi|432092344|gb|ELK24962.1|	88	K	DVFLGTFLYEYSR	R
4639	3	45	F	0.68	4.61	0.946	0.946	gi|432092344|gb|ELK24962.1|	88	K	DVFLGTFLYEYSR	R
5163	3	45	F	-0.34	3.214	0.951	0.951	gi|432092344|gb|ELK24962.1|	88	K	DVFLGTFLYEYSR	R
5159	3	45	F	-0.38	3.107	0.973	0.973	gi|432092344|gb|ELK24962.1|	88	K	DVFLGTFLYEYSR	R
3182	2	45	F	2.52	3.97	0.927	0.927	gi|5915682|sp|P07724.3|ALBU_MOUSE	16	K	ENPTTFM*GHYLHEVAR	R
3172	2	45	F	0.34	4.042	0.96	0.96	gi|5915682|sp|P07724.3|ALBU_MOUSE	16	K	ENPTTFM*GHYLHEVAR	R
5118	3	45	F	-0.66	3.965	0.418	0.418	gi|124028612|sp|P02770.2|ALBU_RAT	27	K	GLVLIAFSQYLQK	C
5358	3	45	F	-0.6	3.131	0.418	0.418	gi|124028612|sp|P02770.2|ALBU_RAT	27	K	GLVLIAFSQYLQK	C
4857	3	45	F	-1.18	3.134	0.446	0.446	gi|124028612|sp|P02770.2|ALBU_RAT	27	K	GLVLIAFSQYLQK	C
3953	3	45	F	2.54	6.325	0.468	0.468	gi|124028612|sp|P02770.2|ALBU_RAT	27	K	GLVLIAFSQYLQK	C
3960	3	45	F	2.32	5.859	0.476	0.476	gi|124028612|sp|P02770.2|ALBU_RAT	27	K	GLVLIAFSQYLQK	C
4930	3	45	F	-0.66	3.391	0.508	0.508	gi|124028612|sp|P02770.2|ALBU_RAT	27	K	GLVLIAFSQYLQK	C
4012	2	45	F	3.66	4.582	0.515	0.515	gi|124028612|sp|P02770.2|ALBU_RAT	27	K	GLVLIAFSQYLQK	C
5110	3	45	F	-0.51	3.055	0.519	0.519	gi|124028612|sp|P02770.2|ALBU_RAT	27	K	GLVLIAFSQYLQK	C
4781	2	45	F	-0.7	4.463	0.56	0.56	gi|124028612|sp|P02770.2|ALBU_RAT	27	K	GLVLIAFSQYLQK	C
4140	2	45	F	2.89	3.89	0.562	0.562	gi|124028612|sp|P02770.2|ALBU_RAT	27	K	GLVLIAFSQYLQK	C
5158	2	45	F	-2.25	3.332	0.564	0.564	gi|124028612|sp|P02770.2|ALBU_RAT	27	K	GLVLIAFSQYLQK	C
4267	2	45	F	-0.52	4.296	0.567	0.567	gi|124028612|sp|P02770.2|ALBU_RAT	27	K	GLVLIAFSQYLQK	C
4777	2	45	F	-0.99	4.803	0.573	0.573	gi|124028612|sp|P02770.2|ALBU_RAT	27	K	GLVLIAFSQYLQK	C
4518	2	45	F	-1.05	3.595	0.576	0.576	gi|124028612|sp|P02770.2|ALBU_RAT	27	K	GLVLIAFSQYLQK	C
4645	2	45	F	-1.26	3.812	0.577	0.577	gi|124028612|sp|P02770.2|ALBU_RAT	27	K	GLVLIAFSQYLQK	C
5162	2	45	F	-2.15	3.686	0.579	0.579	gi|124028612|sp|P02770.2|ALBU_RAT	27	K	GLVLIAFSQYLQK	C
4269	2	45	F	-0.53	3.498	0.581	0.581	gi|124028612|sp|P02770.2|ALBU_RAT	27	K	GLVLIAFSQYLQK	C
4647	2	45	F	-1.32	3.812	0.585	0.585	gi|124028612|sp|P02770.2|ALBU_RAT	27	K	GLVLIAFSQYLQK	C
5041	2	45	F	-1.18	3.864	0.585	0.585	gi|124028612|sp|P02770.2|ALBU_RAT	27	K	GLVLIAFSQYLQK	C

Scan	z	Sample ID	Band	PPM	XCorr	Delta correlation	Unique delta correlation	Reference	Redundancy		Peptides	
4398	2	45	F	0.48	3.453	0.586	0.586	gi|124028612|sp|P02770.2|ALBU_RAT	27	K	GLVLIAFSQYLQK	C
4912	2	45	F	-1.51	4.663	0.589	0.589	gi|124028612|sp|P02770.2|ALBU_RAT	27	K	GLVLIAFSQYLQK	C
5044	2	45	F	-1.32	4.702	0.593	0.593	gi|124028612|sp|P02770.2|ALBU_RAT	27	K	GLVLIAFSQYLQK	C
4142	2	45	F	2.86	3.841	0.595	0.595	gi|124028612|sp|P02770.2|ALBU_RAT	27	K	GLVLIAFSQYLQK	C
4914	2	45	F	-1.6	3.622	0.597	0.597	gi|124028612|sp|P02770.2|ALBU_RAT	27	K	GLVLIAFSQYLQK	C
5278	2	45	F	-2.11	4.569	0.6	0.6	gi|124028612|sp|P02770.2|ALBU_RAT	27	K	GLVLIAFSQYLQK	C
5283	2	45	F	-2.09	4.552	0.6	0.6	gi|124028612|sp|P02770.2|ALBU_RAT	27	K	GLVLIAFSQYLQK	C
4520	2	45	F	-0.99	4.089	0.602	0.602	gi|124028612|sp|P02770.2|ALBU_RAT	27	K	GLVLIAFSQYLQK	C
4392	2	45	F	0.25	3.503	0.605	0.605	gi|124028612|sp|P02770.2|ALBU_RAT	27	K	GLVLIAFSQYLQK	C
5415	2	45	F	-3.92	3.655	0.632	0.632	gi|124028612|sp|P02770.2|ALBU_RAT	27	K	GLVLIAFSQYLQK	C
5412	2	45	F	-3.2	3.371	0.656	0.656	gi|124028612|sp|P02770.2|ALBU_RAT	27	K	GLVLIAFSQYLQK	C
3505	3	45	F	-0.31	3.885	0	0.817	gi|554561044|ref|XP_005874890.1|	0	R	HPDYSVSLLLR	L
3199	3	45	F	-0.08	4.741	0.834	0.834	gi|731280814|ref|XP_010609182.1|	34	K	KQTALAELVK	H
3205	3	45	F	4.42	4.773	0.834	0.834	gi|731280814|ref|XP_010609182.1|	34	K	KQTALAELVK	H
3192	2	45	F	-0.41	3.157	0.896	0.896	gi|731280814|ref|XP_010609182.1|	34	K	KQTALAELVK	H
3703	2	45	F	0.46	2.762	0.879	0.879	gi|671001130|ref|XP_008691426.1|	2	K	LGEYAFQNALLVR	Y
3624	3	45	F	-0.7	6.056	0	0.871	gi|432092344|gb|ELK24962.1|	15	K	LGEYGFQNALLVR^	Y
3737	2	45	F	0.19	3.065	0	0.914	gi|432092344|gb|ELK24962.1|	15	K	LGEYGFQNALLVR^	Y
3898	2	45	F	3.62	3.172	0	0.918	gi|432092344|gb|ELK24962.1|	15	K	LGEYGFQNALLVR^	Y
3726	2	45	F	0.12	3.21	0	0.92	gi|432092344|gb|ELK24962.1|	15	K	LGEYGFQNALLVR^	Y
3866	2	45	F	3.77	3.733	0	0.924	gi|432092344|gb|ELK24962.1|	15	K	LGEYGFQNALLVR^	Y
4052	2	45	F	0.03	4.769	0	0.92	gi|3121749|sp|O35090.1|ALBU_MERUN	3	R	LPC#VEDYLSAILNR	V
4049	2	45	F	4.36	4.526	0	0.949	gi|3121749|sp|O35090.1|ALBU_MERUN	3	R	LPC#VEDYLSAILNR	V
3579	3	45	F	-0.36	5.238	0.831	0.831	gi|5915682|sp|P07724.3|ALBU_MOUSE	12	R	LSQTFPNADFAEITK	L
3554	2	45	F	1.84	4.976	0.922	0.922	gi|5915682|sp|P07724.3|ALBU_MOUSE	12	R	LSQTFPNADFAEITK	L
3565	2	45	F	1.84	4.985	0.924	0.924	gi|5915682|sp|P07724.3|ALBU_MOUSE	12	R	LSQTFPNADFAEITK	L
3198	2	45	F	-0.59	2.616	0.252	0.252	gi|124028612|sp|P02770.2|ALBU_RAT	19	K	LVQEVTDFAK	T
3204	2	45	F	3.44	2.529	0.257	0.257	gi|124028612|sp|P02770.2|ALBU_RAT	19	K	LVQEVTDFAK	T
5085	3	45	F	-1.46	3.252	0	0.961	gi|554561044|ref|XP_005874890.1|	0	R	RHPDYSVSLLLR	L
3477	2	45	F	-0.17	3.447	0.574	0.574	gi|124028612|sp|P02770.2|ALBU_RAT	18	R	RPC#FSALTVDETYVPK	E
2911	2	45	F	0.05	3.111	0.623	0.623	gi|5915682|sp|P07724.3|ALBU_MOUSE	13	K	TPVSEHVTK	C
2778	2	45	F	-0.35	2.828	0.664	0.664	gi|5915682|sp|P07724.3|ALBU_MOUSE	13	K	TPVSEHVTK	C
2775	2	45	F	-0.36	2.859	0.686	0.686	gi|5915682|sp|P07724.3|ALBU_MOUSE	13	K	TPVSEHVTK	C
2908	2	45	F	0.06	2.614	0.758	0.758	gi|5915682|sp|P07724.3|ALBU_MOUSE	13	K	TPVSEHVTK	C
2946	2	46	F	-0.31	3.169	0.843	0.843	gi|3121749|sp|O35090.1|ALBU_MERUN	26	K	APQVSTPTLVEAAR	S
2937	2	46	F	3.11	3.778	0.87	0.87	gi|3121749|sp|O35090.1|ALBU_MERUN	26	K	APQVSTPTLVEAAR	S
3618	2	46	F	3.27	3.155	0.592	0.592	gi|432092344|gb|ELK24962.1|	88	K	DVFLGTFLYEYSR	R
3628	2	46	F	-0.65	2.977	0.912	0.912	gi|432092344|gb|ELK24962.1|	88	K	DVFLGTFLYEYSR	R

Scan	z	Sample ID	Band	PPM	XCorr	Delta correlation	Unique delta correlation	Reference	Redundancy		Peptides	
3604	2	46	F	0.54	3.571	0.628	0.628	gi|124028612|sp|P02770.2|ALBU_RAT	27	K	GLVLIAFSQYLQK	C
3638	2	54	F	-1.37	2.777	0.934	0.934	gi|3121749|sp|O35090.1|ALBU_MERUN	26	K	APQVSTPTLVEAAR	S
4107	2	54	F	-1.2	2.988	0.608	0.608	gi|124028612|sp|P02770.2|ALBU_RAT	27	K	GLVLIAFSQYLQK	C
3876	2	54	F	-0.66	3.767	0	0.93	gi|432092344|gb|ELK24962.1|	15	K	LGEYGFQNALLVR^	Y

z: charge state; #: acrylamidation on C; *: oxidation on M; ^: manually adjusted sequence to LGEYGFQNAILVR-see Supplementary data I (Table I) for details.
